# Analysis of the human health threat caused by the red imported fire ant in mainland China

**DOI:** 10.1371/journal.pone.0350501

**Published:** 2026-06-16

**Authors:** Huimei Chen, Hongzhi Zhuang, Xiaojing Zhang, Yuling Liang, Mingrong Liang, Yongyue Lu, Lei Wang

**Affiliations:** 1 Red Imported Fire Ant Research Center, South China Agricultural University, Guangzhou, China; 2 Department of Emergency, Jinjiang Hospital of Traditional Chinese Medicine, Jinjiang, China; 3 School of Biological Sciences, The University of Hong Kong, Hong Kong SAR, China; PLOS: Public Library of Science, UNITED STATES OF AMERICA

## Abstract

In China, the spread of the red imported fire ant (*Solenopsis invicta* Buren, RIFA) exhibits a consistent pattern of expansion from the south to the north, with decreasing severity toward northern regions. The rapid spread of RIFA poses a substantial public health threat in China, particularly in regions where this invasive species has become established. To evaluate the health risks associated with RIFA stings in China, we analyzed the probability of developing various symptoms following RIFA stings using 8,749 representative cases with detailed symptom descriptions recorded between 2004 and 2024. Data were collected from literature reviews, Baidu (a Chinese search platform), and Jinjiang Hospital of Traditional Chinese Medicine. Meanwhile, the potential threat to public health was estimated based on the population density of affected regions in China. The recorded symptoms and their respective occurrence rates were as follows: itching and pain (77.54%), vesicles or pustules (36.12%), urticaria or papules (33.33%), systemic allergy (7.52%), fever (2.65%), dizziness or headache (1.97%), shock (1.17%), localized allergic lymphadenopathy (0.94%), speech impediment (0.75%), and death (0.03%). Based on the distribution, severity, and population density of RIFA in China, it is estimated that approximately 0.6992 million people are likely to be stung annually, including about 0.5422 million experiencing itching and pain, 0.2526 million developing vesicles or pustules, 0.2330 million with urticaria or papules, 526,000 with systemic allergy, 185,000 with fever, 138,000 with dizziness or headache, 82,000 with shock, 66,000 with localized allergic lymphadenopathy, 52,000 with speech impediments, and 209 deaths. RIFA stings can induce symptoms ranging from mild itching and swelling to severe systemic allergy, including anaphylaxis and death, constituting a serious public health concern. Currently, RIFA has spread to more than 700 counties and districts across 13 provinces, endangering the health of approximately 120 million people. Based on current data, an estimated 699,224 individuals may be affected annually, with 8,181 potentially experiencing anaphylactic shock and 209 deaths. There is an urgent need for enhanced protective measures and treatment options. Strengthening monitoring and prevention strategies is critical for mitigating the impact of this invasive species and safeguarding public health in affected regions.

## Introduction

The red imported fire ant (*Solenopsis invicta* Buren, RIFA) is a highly invasive species that poses a substantial threat to human health, ecosystems, agriculture, and public safety [1–3]. Due to its strong adaptability and high reproductive capacity, RIFA has caused significant economic losses and environmental degradation in its infested regions [4–7]. The combined costs of RIFA management and medical treatment in the United States alone are estimated at approximately USD 6 billion per year [8]. RIFA was first detected in mainland China in 2004 [9] and has now spread to 703 counties across 13 provinces in 2025 [10]. The rate of spread of RIFA in China is approximately 48.1 km per year [11], and recent studies indicate that the species remains in a rapid expansion phase, with an annual average of 41–52 newly infested counties [12].

RIFA exhibits strongly aggressive behavior, manifested through both interspecific invasion and territorial defense. When a RIFA nest is disturbed, the workers rapidly swarm out to attack the intruder using their stings and venom [13]. The venom of RIFA workers contains multiple allergenic proteins, including Sol i1, Sol i2, Sol i3, and Sol i4, which damage human tissues and the immune system, triggering inflammatory responses and inducing IgE-mediated allergic reactions [14–16]. The typical local reactions following RIFA stings are itching and burning pain, accompanied by redness and the development of raised wheals and white pustules at the sting site, followed by IgE-mediated urticarial and erythematous reactions [17]. Systemic allergic symptoms manifest as generalized itching, rashes, flushing, and swelling of the head, neck, and face, which may progress to the limbs and trunk, potentially leading to laryngeal edema, asthma, respiratory distress, or even anaphylactic shock [16].

RIFA stings can also lead to severe complications. For instance, they have been reported to cause cases of rhabdomyolysis and acute renal failure as secondary complications [18]. Furthermore, RIFA stings may induce neurological manifestations, including convulsions, altered consciousness, blurred vision, mental disturbances, drowsiness, and even epileptic seizures [19].

The main habitat of RIFA in China includes farmland and urban green spaces, which strongly overlap with areas of human activity [20,21]. Consequently, their pronounced aggressiveness and potent venom pose a significant threat to human health [22,23]. In the United States, the elderly, infants, and individuals with cognitive impairments are particularly susceptible to RIFA attacks [24,25]. During flood periods, RIFA can form floating “rafts”, posing potential dangers to people walking, wading, or boating nearby [26].

The probability of residents in RIFA-infested areas being stung may depend on the severity of infestation, human behavioral patterns (e.g., outdoor work and exposure risk), and environmental conditions influencing activity levels. Previous research indicates that 50–89% of residents in RIFA-infested areas are stung each year in the United States [23,27], while Pereira et al. [28] reported a more conservative estimate of 30–60%. In China, the annual probability of residents in infested areas being stung by RIFA is estimated to range from 27.80–37.70% [29–32]. Thus, a reasonable estimate is that approximately 30% of residents in such regions are stung each year [33,34].

In the United States, the probability of experiencing an allergic reaction due to RIFA stings is reported to be between 0.60–16% [35,36]. In Georgia, approximately 5% of those stung require medical treatment, of whom 16% experience systemic allergy [37]. The prevalence of allergic reactions to RIFA stings has also been investigated in China by Xu et al. [32] and Zhao and Xu [38]. The investigation results indicate all people who have been stung experience itchiness, and almost all experience flare and wheal, while nearly 10% experience fever. However, the number of infested counties increased from 245 to 703 between 2015 and 2025 [10,12], suggesting that a growing number of people have been exposed to RIFA stings in China.

This study collected cases of RIFA stings not only from online sources and published literature but also from hospital records with detailed symptom descriptions between 2004 and 2024. The aim was to confirm the prevalence of allergic reactions and to predict future trends in the health impacts of RIFA in China.

## Methods

### Data collection

Data on the distribution and intensity of RIFA infestations in mainland China in 2023 were obtained from the Plant Protection and Quarantine Information Management System [39]. National population data were derived from the Seventh National Population Census, and the total national land area was retrieved from the National Bureau of Statistics [40].

To identify documented cases of RIFA stings between September 2004 and May 2024, we systematically searched the China National Knowledge Infrastructure (CNKI), the Wanfang Medical Network (WMN), and Baidu (a Chinese search platform). Search terms included the Chinese equivalents of “RIFA” or “ant,” combined with “allergy,” “injury,” “bite,” or “sting,” along with additional keywords related to human attacks. The search results were carefully screened, and only confirmed RIFA sting cases with detailed symptom descriptions were included in this study. Conversely, sting cases lacking key information (e.g., unidentified ant species) were excluded from the analysis.

In total, 5,545 cases were extracted from 17 peer-reviewed research papers (Table 1) and 13 news reports (S1 Text). Additionally, 3,204 cases were obtained from the Emergency Department of Jinjiang Hospital of Traditional Chinese Medicine between 2020 and 2023 (Table 1), yielding a final dataset of 8,749 cases for analysis. All records were meticulously cross-checked during manuscript preparation to ensure there was no data overlap. Specifically, Jinjiang Hospital of Traditional Chinese Medicine is a county-level institution serving patients primarily from surrounding localities; the 17 research studies were conducted in regions outside Jinjiang County, and none of the news reports included cases from this area. Furthermore, cross-verification confirmed no overlap between cases derived from research articles and those from news sources.

**Table 1:** Statistics of symptoms after RIFA sting in different regions of China.

Area	Total number of stings (individual)	Number of persons with each type of symptom											
		Itching and pain	Vesicles or pustules	Urticaria or papules	Fever	Dizziness and headaches	Localized allergic lymphadeno-pathy	Systemic allergy	Speech impediment	Shock	Death	Particular year	Source
Jinjiang, Fujian	865	815	125	451	11	17	7	161	8	8	0	2020	Hospital*, 2020
Jinjiang, Fujian	947	876	143	427	7	5	5	174	10	10	0	2021	Hospital, 2021
Jinjiang, Fujian	1 021	941	101	511	4	9	11	146	8	7	0	2022	Hospital, 2022
Jinjiang, Fujian	371	302	36	147	1	2	8	34	3	3	0	2023	Hospital, 2023
Zhangzhou, Fujian	468	NA	NA	NA	NA	NA	0	23	23	23	0	2017.2-2020.3	(54)**
Wuchuan, Guangdong	970	970	902	931	50	24	43	4	0	0	0	1999-2004	(55)
Gaozhou, Guangdong	991	991	625	7	2	1	7	7	0	1	0	2005.6−8	(56)
Dongguan, Guangdong	78	75	25	43	1	0	0	1	0	1	1	2006.6	(57)
Dongguan, Guangdong	1	1	NA	1	0	NA	0	1	0	1	0	2006.6.20	(58)
Guangdong T-Village	72	72	24	39	1	1	0	0	0	0	0	2006	(59)
Jiangmen, Guangdong	63	55	55	55	11	15	0	27	5	9	0	2009.3-2011.6	(60)
Foshan, Guangdong	24	24	24	24	0	8	0	12	3	0	0	2011.11-2012.11	(61)
Yangjiang, Guangdong	127	127	127	107	13	5	0	7	0	0	0	2015.6-2016.12	(62)
Nansha, Guangdong	1 572	498	402	NA	77	77	0	0	0	0	0	1999-2013.3	(63)
A school in Guangdong	2	2	2	2	NA	1	0	0	0	0	0	2007	(64)
South China Sea islands and reefs, Hainan	110	110	71	99	NA	NA	0	11	0	11	0	2019.1-2020.1	(65)
Nansha Island, Guangdong	61	61	41	6	1	2	1	11	0	0	0	2019.3−6	(66)
An Island Reefs, Guangdong	3	3	3	3	0	2	0	3	3	3	0	2021.1−6	(67)
Banking School,Guangxi	66	50	50	50	NA	NA	0	16	0	2	0	2021.5−10	(68)
Liuzhou, Guangxi	30	30	7	10	NA	NA	0	0	2	0	0	2016-2020	(69)
A Ministry, Guangxi	881	755	386	NA	52	NA	0	15	0	15	0	2022.6-2023.6	(70)
Nanning, Guangxi	1	NA	NA	NA	NA	NA	0	0	0	1	1	2018	News (S1 Text)
Shaoguan, Guangdong	1	NA	NA	NA	NA	NA	0	0	0	1	0	2024	
Dongguan, Guangdong	1	NA	NA	NA	NA	NA	0	0	0	1	1	2006	
Yulin, Guangxi	1	NA	NA	NA	1	0	0	1	0	1	1	2021	
Ningde, Fujian	1	1	NA	1	NA	1	0	1	0	0	0	2023	
Xichang, Sichuan	3	3	NA	NA	NA	1	0	0	0	2	0	2020-2022	
Panzhihua, Sichuan	6	6	NA	NA	NA	NA	0	2	0	0	0	2020	
Luzhou, Sichuan	1	1	NA	1	NA	1	0	0	1	1	0	2018	
Hainan	6	6	6	NA	NA	NA	0	1	0	0	0	2015	
Jiangxi	1	1	1	1	0	0	0	0	0	1	0	2017	
Hubei	6	6	NA	NA	NA	0	1	0	0	0	0	2021	
Kunming, Yunnan	1	1	1	NA	NA	NA	0	0	0	0	0	2021	
Yunnan Honghe	2	2	2	NA	NA	NA	0	0	0	0	0	2014	
Add up the total	8 749	6 784	3 160	2 916	232	173	82	658	66	102	3		
Percentage of symptom occurrence (%)		77.54	36.12	33.33	2.65	1.98	0.94	7.52	0.75	1.17	0.03		

Note: Common and non-serious symptoms (e.g., itching and pain, vesicles or pustules, urticaria or papules, fever, and dizziness or headache) not mentioned in the literature or reports are uniformly denoted as ‘NA’. For the five serious symptoms (localized allergic lymphadenopathy, systemic allergy, speech impediment, shock, and death), unrecorded instances are assumed to be absent and recorded as 0 to avoid excessive bias.

Table 1 presents detailed geographical information for the 8,749 included RIFA sting cases. The majority occurred in Guangdong Province (*n* = 4,076) and Fujian Province (*n* = 3,673), followed by Guangxi (*n* = 979), Sichuan (*n* = 10), Hainan (*n* = 6), Yunnan (*n* = 3), and Jiangxi (*n* = 1).

### Dynamics of RIFA stings in different years in China

Statistical analyses were performed to determine the proportion of various clinical symptoms experienced by individuals following RIFA stings. To examine the temporal relationship between the number of sting cases and the progression of the RIFA invasion, the data were aggregated into four-year intervals. This approach was chosen to account for inconsistencies and temporal overlaps in sting reports across studies conducted in different years. All statistical analyses were performed using SPSS version 27.0 (IBM Corp., Armonk, NY, USA), and chi-square tests were employed to evaluate differences among groups.

### Prediction of the number of people with different symptoms after RIFA stings

Data on the distribution area of RIFA infestations across China (as of 2023) were obtained from the Plant Protection and Quarantine Information Management System, which is part of the Government Information System Management Platform of the Ministry of Agriculture and Rural Affairs of the People’s Republic of China. The total affected area nationwide (S) was calculated using Formula 1:


$$\begin{array}{c} \begin{array}{c} S=\sum_{i=\text{1}}^{\text{5}}\; \end{array} \end{array}$$ (1)

In the formula, S represents the total area of infected regions (in square kilometers), while Si represents the total infected area at level i (in square kilometers). Based on the calculated data, the total infected area is categorized into five levels according to its severity: a total infected area of 0.001–0.6 km² is classified as level 1, 0.7–3.3 km² as level 2, 3.4–6.6 km² as level 3, 6.7–33.3 km² as level 4, and areas larger than 33.3 km² are classified as level 5.

### Analyses of symptoms caused by RIFA stings in different regions of China

According to previous research and clinical guidelines [41], the symptoms following RIFA stings were categorized into ten types: itching and pain, urticaria or papules, vesicles or pustules, systemic allergy, fever, dizziness or headache, localized allergic reactions or lymphadenopathy, speech impediment, shock, and death (Table 1). The proportion (W) of each symptom observed among the collected cases was analyzed and calculated using Formula 2:


$$\begin{array}{c} \begin{array}{c} Wi=\frac{Ni}{N} \end{array} \end{array}$$ (2)

In the formula, Ni represents the total number of people exhibiting a specific symptom after been stung by RIFA, while N represents the total number of cases recorded in the study.

The national area data is sourced from the National Bureau of Statistics, and the national population data is acquired from the Seventh National Census. The population density ρ is calculated using Formula 3:


$$\begin{array}{c} \begin{array}{c} \rho =\frac{N}{M} \end{array} \end{array}$$ (3)

In the formula, N represents the total national population [in people], and M represents the total national land area (in square kilometers).

The total population in the areas infected by RIFA H is calculated using Formula 4:


$$\begin{array}{c} \begin{array}{c} H=\rho \times S \end{array} \end{array}$$ (4)

The total number of stung individuals in areas affected by RIFA K is calculated using Formula 5:


$$\begin{array}{c} \begin{array}{c} K=\sum_{i=\text{1}}^{\text{5}}Hi\times ci \end{array} \end{array}$$ (5)

In this formula, K represents the total number of stung individuals. The variable ci corresponds to the area level of RIFA infestation, which is determined based on statistical analysis of existing data. The values are defined as follows: c1 = 0.15, c2 = 0.30, c3 = 0.60, c4 = 0.80, and c5 = 0.90. These coefficients represent the proportion of the population at each infestation level that is likely to be harmed by RIFA.

## Results

### The occurrence of RIFA in China

The distribution of RIFA in China is illustrated in S1 Table. As of April 2023, RIFA had invaded 589 counties across 12 provinces in mainland China, including Chongqing, Fujian, Guangdong, Guangxi, Guizhou, Hainan, Hubei, Hunan, Jiangxi, Sichuan, Yunnan, and Zhejiang. The total area infested by RIFA was approximately 4,153.3 km². The area classified as level 1 infestation was 2,462.3 km², level 2 infestation 1,132.3 km², level 3 infestation 463.8 km², level 4 infestation 80.6 km², and level 5 infestation 14.2 km².

Guangdong Province had the largest infested area, covering 1,567 km², while Guangxi had the largest area classified as level 5 infestation, at 4.8 km². The infested areas in Hubei, Zhejiang, Chongqing, and Guizhou were relatively small, with total infestation areas of 4.8 km², 9.6 km², 10.1 km², and 14.1 km², respectively. Overall, RIFA infestation in mainland China exhibits a spatial trend of continuous expansion from south to north, accompanied by a gradual decrease in infestation severity.

### Dynamics of RIFA stings in different years in China

**Fig 1:**
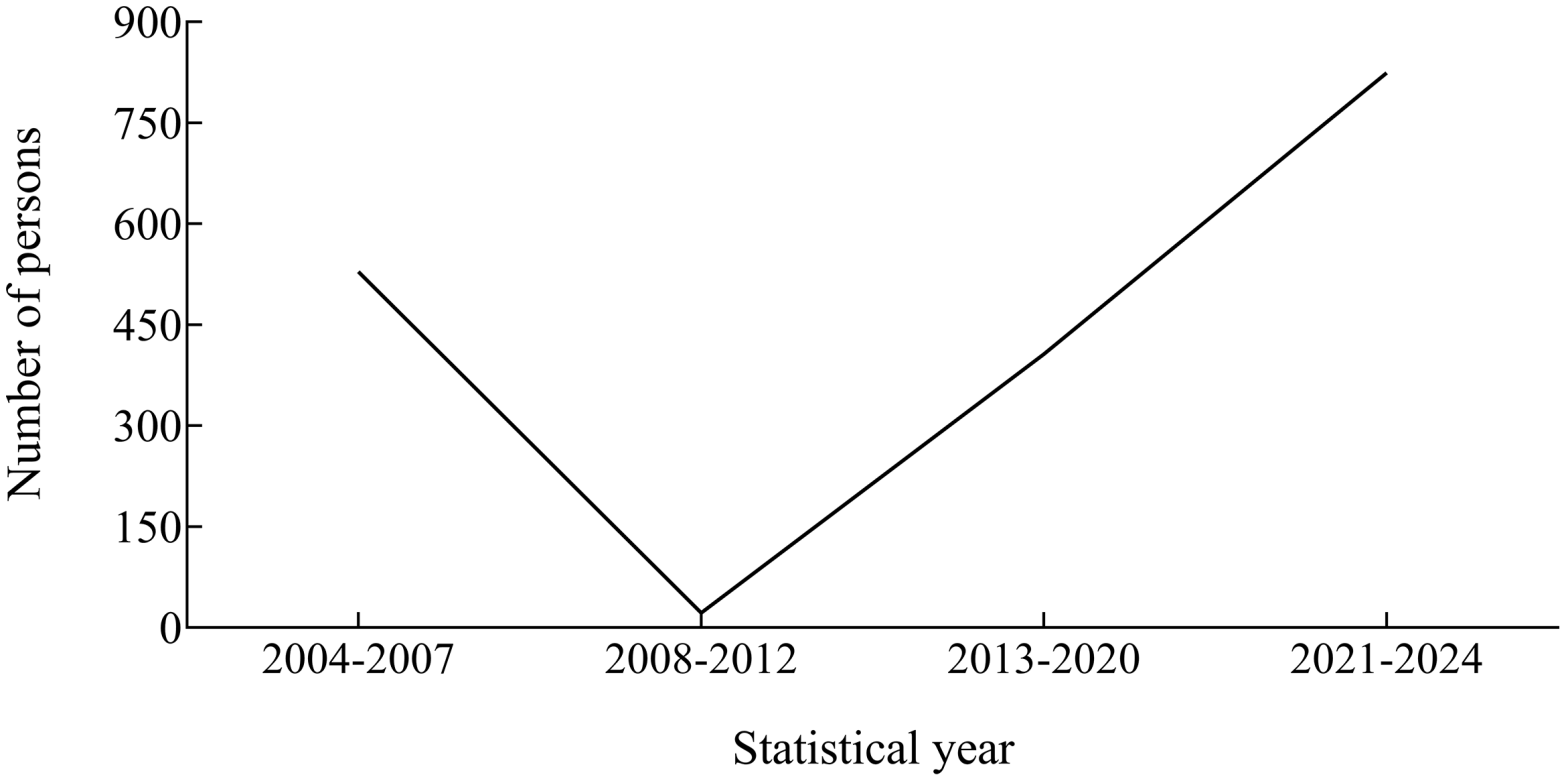
Annual average number of human sting cases caused by RIFA in different years after its invasion.

From 2004 to 2024, the overall trend in RIFA sting cases shows a fluctuating upward trajectory (Fig 1). During the period from 2004 to 2007, the average annual number of reported sting cases exceeded 500. However, between 2008 and 2012, the number of cases declined sharply to approximately 20 per year. Subsequently, during the period from 2013 to 2020, the average annual number of reported cases rebounded to around 400. Notably, from 2021 to 2024, the number of recorded sting cases increased significantly, with the average annual number of reported cases reaching 824.

### Analyses of symptoms caused by RIFA stings in different regions of China

Among the 8,749 collected cases, 6,784 (77.54%) experienced itching and pain, 3,160 (36.12%) developed vesicles or pustules, and 2,916 (33.33%) presented with urticaria or papules. Additionally, 658 cases (7.52%) developed systemic allergy, 232 (2.65%) had fever, 173 (1.98%) experienced dizziness or headache, 102 (1.17%) suffered shock, 82 (0.94%) exhibited localized allergic lymphadenopathy, 66 (0.75%) temporarily lost their voice (speech impediment), and 3 cases (0.03%) resulted in death (Table 1).

The proportions of symptoms experienced after stings varied among provinces, with the majority of cases concentrated in Guangdong, Fujian, and Guangxi. Among the three fatal cases, two occurred in Guangxi and one in Guangdong.

### Prediction of the number of people with different symptoms after RIFA stings

**Fig 2:**
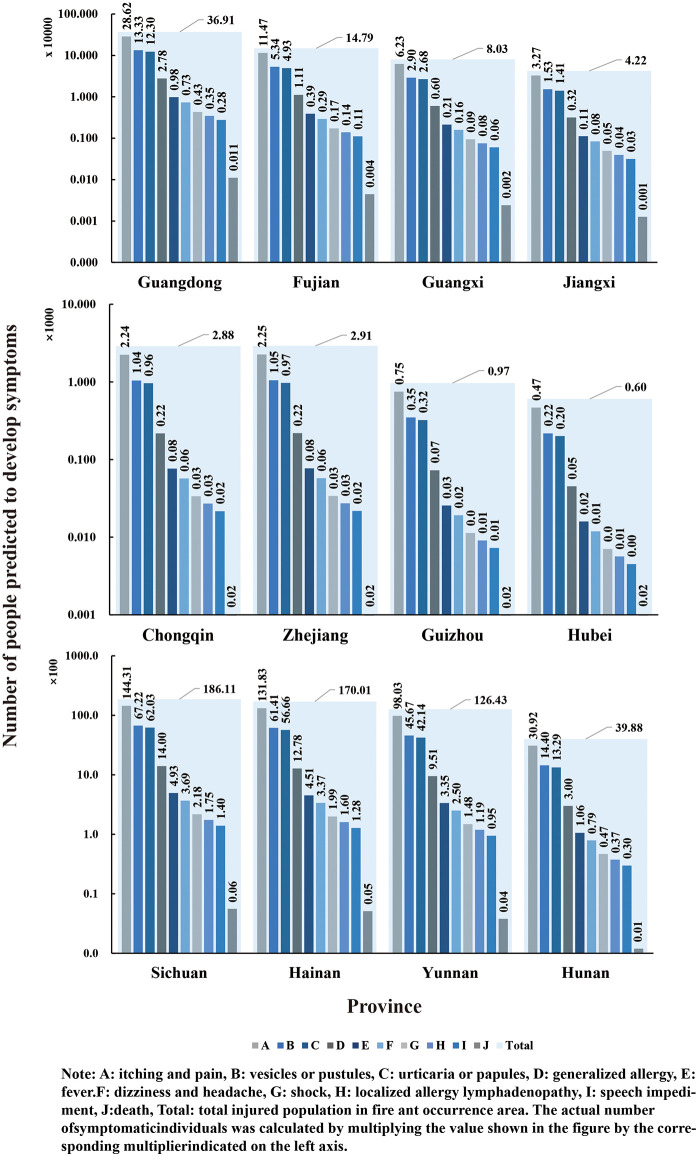
The number of stung cases by RIFA in different years after its invasion.

Based on the results above and national population density, predictions indicated that the total population in RIFA-infested regions across 12 provinces was 2.9917 million, with approximately 0.6992 million individuals stung by RIFA, representing about 23.37% of the population (Fig 2, Table 2). The predicted number of stung individuals was 0.3691 million in Guangdong Province, the highest among all infested provinces, accounting for 21.85% of the total population in Guangdong’s infested regions. Fujian Province followed with 0.1479 million individuals, accounting for 24.43% of the population in infested areas, while Guangxi Province ranked third, with 0.0803 million individuals, representing 27.57% of the population in its infested regions. Hubei Province was expected to have only about 600 affected individuals, accounting for 22.94% of the total population in the province.

**Table 2:** Prediction on the number of individuals stung by RIFA and their symptoms after RIFA stings in China.

Provinces (autonomous regions)	Total number of people in the area of occurrence (persons)	Number of individuals stung by RIFA in the area of occurrence (persons)	Number of people showing various types of symptoms after being stung (persons)									
			Itching and pain	Vesicles or pustules	Urticaria or papules	Systemic allergy	Fever	Dizziness or headache	Shock	Localized allergic lymphadenopathy	Speech impediment	Death
Chongqing	13 970.0	2 883.5	2 235.9	1 041.5	961.1	216.8	76.4	57.1	33.7	27.1	21.6	0.9
Fujian	605 458.3	147 924.8	114 700.9	53 430.4	49 303.3	11 123.9	3 920.0	2 928.9	1 730.7	1 390.5	1 109.4	44.4
Guangdong	1 689 581.0	369 139.7	286 230.9	133 333.3	123 034.3	27 759.3	9 782.2	7 309.0	4 318.9	3 469.9	2 768.5	110.7
Guangxi	291 271.0	80 320.9	62 280.8	29 011.9	26 771.0	6 040.1	2 128.5	1 590.4	939.8	755.0	602.4	24.1
Guizhou	3 782.5	966.6	749.5	349.1	322.2	72.7	25.6	19.1	11.3	9.1	7.2	0.3
Hainan	70 268.0	17 001.0	13 182.6	6 140.8	5 666.4	1 278.5	450.5	336.6	198.9	159.8	127.5	5.1
Hubei	2 625.5	602.2	466.9	217.5	200.7	45.3	16.0	11.9	7.0	5.7	4.5	0.2
Hunan	17 658.4	3 988.1	3 092.3	1 440.5	1 329.2	299.9	105.7	79.0	46.7	37.5	29.9	1.2
Jiangxi	170 811.4	42 236.0	32 749.8	15 255.7	14 077.3	3 176.2	1 119.3	836.3	494.2	397.0	316.8	12.7
Sichuan	70 765.7	18 611.3	14 431.2	6 722.4	6 203.1	1 399.6	493.2	368.5	217.8	174.9	139.6	5.6
Yunnan	45 469.3	12 643.1	9 803.5	4 566.7	4 214.0	950.8	335.0	250.3	147.9	118.8	94.8	3.8
Zhejiang	10 068.2	2 906.8	2 254.0	1 049.9	968.8	218.6	77.0	57.6	34.0	27.3	21.8	0.9
Add up the total	2 991 729.3	699 224.2	542 178.4	252 559.8	233 051.4	52 581.7	18 529.4	13 844.6	8 180.9	6 572.7	5 244.2	209.8
2×	5 983 458.5	1 398 448.3	1 084 356.8	505 119.5	466 102.8	105 163.3	37 058.9	27 689.3	16 361.8	13 145.4	10 488.4	419.5
3×	8 975 187.8	2 097 672.5	1 626 535.3	757 679.3	699 154.3	157 745.0	55 588.3	41 533.9	24 542.8	19 718.1	15 732.5	629.3

Note: For the five common and non-serious symptoms of itching and pain, vesicles or pustules, urticaria or papules, fever, and dizziness or headache, in statistics, and for the five serious symptoms of localized allergic lymph node enlargement, systemic allergy, speech impediment, shock, and death.

Based on the statistical results above, the estimated number of residents in RIFA-infested areas who are expected to exhibit corresponding symptoms after RIFA stings each year is summarized as follows. Common symptoms: itching and pain are expected to occur in approximately 0.5422 million individuals, urticaria or papules in 0.2330 million, and vesicles or pustules in 0.2526 million. Moderately severe symptoms: systemic allergy are predicted to occur in approximately 0.0526 million individuals, fever in 0.0185 million, and dizziness or headache in 0.0138 million. Rare symptoms: localized allergic lymphadenopathy is expected in approximately 6,600 individuals, speech impediment in 5,200, shock in 8,200, and death in about 209 individuals.

Among the affected regions, Guangdong and Fujian provinces are predicted to have the highest numbers of residents experiencing severe systemic allergy following RIFA stings. The projected annual number of anaphylactic shock cases is expected to reach approximately 1,700 in Fujian and 4,300 in Guangdong. The majority of fatal cases are also anticipated to be concentrated in these two provinces. In contrast, the likelihood of fatal incidents in provinces such as Hubei and Guizhou is anticipated to remain low, owing to the relatively mild infestation levels in these areas.

These projections underscore the urgent need for targeted health interventions and increased public awareness in the most severely affected regions–particularly in Guangdong and Fujian, where the risk of severe systemic allergy and fatalities is greatest. Furthermore, the findings highlight the importance of sustained monitoring and management of RIFA infestations to mitigate their potential impact on public health.

Assuming the total population in RIFA-infested regions doubles or triples from current levels, the number of individuals harmed by RIFA is projected to rise to approximately 1.3980 million and 2.0980 million, respectively. Correspondingly, the number of individuals exhibiting specific symptoms would increase substantially.

Itching and pain would affect approximately 1.0840 million and 1.6270 million individuals, vesicles or pustules 0.5050 million and 0.7580 million, urticaria or papules 0.4660 million and 0.6990 million, systemic allergy 0.1050 million and 0.1580 million, and fever 0.0370 million and 0.0560 million. Additionally, dizziness or headache would occur in 0.0280 million and 0.0420 million individuals, shock would affect 0.0160 million and 0.0250 million, localized allergic lymphadenopathy would develop in 0.0130 million and 0.0200 million, speech impairment would impact 0.0110 million and 0.0160 million, and deaths would increase to approximately 400 and 600, respectively (Table 2).

## Discussion

In this study, we conducted a statistical analysis of RIFA occurrence and associated sting cases in mainland China over the past two decades. Our findings indicate a continuous northward expansion of RIFA across the country. While the frequency of stings has exhibited an increasing trend, the severity of symptoms experienced by individuals appears to correlate with the degree of RIFA infestation. This suggests that RIFA is likely to pose an increasing threat to public health in China.

With the continuous growth in global mobility driven by international trade, the spread of RIFA has accelerated [8]. The present study indicates that the expansion of RIFA in China follows a south-to-north pattern, accompanied by a gradual reduction in infestation severity. This trend may be attributed to the initial detection of RIFA populations in Guangdong and the favorable climatic and ecological conditions supporting their survival and reproduction in southern China. The species remains in a rapid expansion phase in China [12].

A predictive analysis conducted by Yin et al. [42] using the Biomod2 ensemble model predicted that the potential suitable habitat for RIFA in China is primarily located south of 30°N latitude, encompassing an estimated area of approximately 152.34 × 10³ km². Consequently, the number of residents affected by RIFA stings in China is expected to continue rising, posing significant challenges for future control and prevention efforts.

Since RIFA were first detected in mainland China in 2004, the overall number of individuals stung has exhibited a fluctuating upward trend. From 2004 to 2007, over 2,000 individuals were reported to have been stung. This high number can be attributed to the early stage of the invasion, when residents in affected regions lacked adequate awareness of RIFA and effective control systems had not yet been established. The high severity of infestations during this period also contributed to the large number of sting incidents.

However, following several years of intensive prevention and control efforts, the total number of sting cases sharply declined to approximately 100 during 2009–2012. According to previous studies, China’s annual expenditure on RIFA control increased from USD 267,000 (2.03 million CNY at the 2007) to USD 1,735,000 (10.95 million CNY at the 2012) [43]. This substantial investment in control measures significantly reduced the prevalence and severity of infestations, thereby lowering the likelihood of residents in affected areas being stung.

Nevertheless, the total number of sting cases increased again between 2013 and 2024. This resurgence was likely driven by the accelerated spread of RIFA within China [44], resulting from a significant expansion of the infested area.

Our results suggest that approximately 23.37% of the population in RIFA-infested regions experience RIFA stings each year. Xu et al. [32] reported that approximately one-third of individuals had been stung in ant-infested areas, while a conservative estimate from the United States indicates that 30–60% of residents in RIFA-infested areas are stung annually [28]. These comparisons indicate that our estimates are generally consistent with previous studies.

Because the vast majority of individuals stung by RIFA do not seek medical attention unless concerned about serious complications or motivated by specific circumstances, the sting incidence rate may therefore be underestimated. Based on our calculations, approximately 209 deaths should occur annually, however, only three fatalities have been documented. This discrepancy appears to be substantial. We speculate that it arises because most individuals experiencing mild symptoms are underreported, as they neither seek medical care nor appear in news reports. As deaths are serious events that are almost always documented, the mortality rate associated with RIFA stings is likely overestimated.

The proportion of RIFA sting cases relative to the total population and the severity of allergic reactions vary among regions. The sting cases collected in this study are primarily concentrated in Guangdong, Fujian, and Guangxi. Our results are consistent with those of Zhao and Xu [38], who surveyed the prevalence of RIFA sting incidents using internet-based reports collected between 2003 and 2015.

This pattern can be attributed partly to the extensive affected areas and widespread infestations in these provinces. For example, Guangdong has the largest infested area, whereas Guangxi exhibits the highest RIFA density among infested regions. Additionally, these provinces lie within the subtropical climatic zone, characterized by hot summers and mild winters that favor the activity of RIFA. Higher RIFA activity increases the likelihood of human stings. For example, surveys have demonstrated that most RIFA sting incidents in both China and the United States occur during spring, summer, and autumn, particularly in the warmer months [17,38].

Moreover, RIFA have established dominance across diverse habitats in southern China, particularly in farmland and urban green spaces [20,21]. The frequent overlap between ant activity and human presence elevates the probability of sting incidents in these provinces. For instance, Zhao and Xu [38] found that most RIFA stings occurred in habitats such as greenbelts (41%), farmland (32%), and parks (16%). Furthermore, because RIFA have persisted in southern China for more than 20 years, the local population and relevant institutions are more familiar with and vigilant in monitoring and reporting this pest. As a result, diagnoses of ant stings tend to be more accurate, and symptom descriptions more detailed–helping to explain why the majority of recorded sting cases are concentrated in Guangdong, Guangxi, and Fujian.

Localized reactions-including itching, pain, and pustule formation–are commonly observed following RIFA stings, whereas systemic symptoms such as generalized allergic reactions, speech impairment, and shock occur much less frequently. This finding aligns with previous studies on RIFA sting symptoms [32,38,45]. For example, 56% of stung individuals experience noticeable localized reactions, whereas only 0.5–2% exhibit systemic allergy [45].

The severity of symptoms following RIFA stings varies significantly among individuals. On the one hand, differences in individual tolerance to allergenic components in the venom and the number of stings received play important roles. Repeated exposure to the allergen can lead to progressively more severe allergic reactions [46,47]. The venom of RIFA consists of alkaloids and proteinaceous components, which cause inflammation and anaphylactic responses [14]. However, the composition of antigens and alkaloids in worker venom may vary according to caste, geographic location, habitat type, and season [47–49]. Such variation may explain differences in the severity of RIFA stings. For instance, seasonal variation in sting severity has been observed in Georgia, United States, possibly due to changes in venom potency [17].

This study demonstrates that RIFA stings in infested regions represent a significant threat to public health in China. The current population in RIFA-infested areas is estimated to be 2.9917 million, with approximately 0.6992 million individuals stung annually-corresponding to a sting rate of 23.37%. Among those stung, thousands are predicted to experience severe symptoms–including systemic allergy, speech impairment, and shock–and a non-negligible number of fatalities is projected. In the United States, approximately 9.30 million people are stung by RIFA each year, with an average sting rate of 31.00%. Over half of the population in infested areas are expected to be stung at least once in their lifetime [50], which aligns with the findings of this study.

Lopez et al. [41] reported that 38.30% of children in infested areas were allergic to RIFA venom, with allergy rates increasing with age–from 35.70% among children aged 2–5 years to 57.50% among adolescents aged 11–20 years. Children and the elderly are particularly vulnerable due to limited mobility, insufficient awareness of RIFA, and weaker immune systems. As such, children, the elderly, and individuals with allergic tendencies constitute high-risk groups and require enhanced protective measures [51]. Previous studies have shown that many fatalities caused by RIFA stings result from delayed or inadequate treatment, leading to anaphylactic shock or secondary complications [52]. Although whole-body extract immunotherapy (RIFA WBE-IT) has proven effective in preventing systemic allergy caused by RIFA stings [53–70], there remains an urgent need for strengthened control and prevention strategies to mitigate the risks RIFA pose to human health. In this study, certain symptoms were recorded as “NA” (missing data) due to incomplete documentation in some case records. The lack of comprehensive statistical data may have resulted in an underestimation of the incidence of certain symptoms. Moreover, because of variations in data sources and the absence of a standardized emergency treatment protocol, the classification of symptoms by severity may have lacked standardized and consistent criteria. Therefore, there is an urgent need to establish a nationwide standard for the emergency management and clinical classification of RIFA stings. Such a protocol would ensure more accurate symptom recording and facilitate a more precise assessment of the health risks posed by RIFA.

## Conclusions

RIFA is a highly destructive invasive species of considerable international concern. Stings from RIFA can cause symptoms ranging from mild redness, swelling, pain, and itching at the sting site to severe systemic allergy, including anaphylactic shock and even death, posing a serious threat to public health.

In China, RIFA has exhibited a consistent south-to-north expansion pattern, with decreasing infestation severity toward the north. At present, RIFA has invaded more than 600 counties and districts across 12 provinces, threatening the health of approximately 120 million people. Based on current data, it is estimated that about 699,224 individuals may be affected by RIFA stings annually, with approximately 8,181 potentially experiencing anaphylactic shock and around 209 deaths each year.

Consequently, there is an urgent need for effective protective measures and therapeutic interventions against RIFA stings. Strengthening RIFA monitoring systems and implementing comprehensive preventive strategies are imperative to mitigate the impact of this invasive species and safeguard public health and safety in affected regions.

## Supporting information

S1 Table Table1 National raw data table on infestation severity and symptom prediction of red imported fire ant (RIFA). (DOCX)

S1 Text URL and content of news reports related to red imported fire ant stings. (DOCX)

## References

[1] Wojcik DP, Allen CR, Brenner RJ, Forys EA, Jouvenaz DP, Lutz RS. Red imported fire ant: impact on biodiversity. Am Entomol. 2001;47(1):16–23. doi: 10.1093/ae/47.1.16

[2] Gutrich JJ, VanGelder E, Loope L. Potential economic impact of introduction and spread of the red imported fire ant, Solenopsis invicta, in Hawaii. Environmental Science & Policy. 2007;10(7–8):685–96. doi: 10.1016/j.envsci.2007.03.007

[3] Dunham AE, Mikheyev AS. Influence of an invasive ant on grazing and detrital communities and nutrient fluxes in a tropical forest. Divers Distrib. 2010;16(1):33–42. doi: 10.1111/j.1472-4642.2009.00620.x

[4] Williams DF, Collins HL, Oi DH. Red imported fire ant (Hymenoptera: Formicidae): an historical perspective of treatment programs and the development of chemical baits for control. Am Entomol. 2001;47(3):146–59. doi: 10.1093/ae/47.3.146

[5] Ross KG, Shoemaker DD. Estimation of the number of founders of an invasive pest insect population: the fire ant Solenopsis invicta in the USA. Proc Biol Sci. 2008;275(1648):2231–40. doi: 10.1098/rspb.2008.0412 18577505 PMC2603238

[6] Wickings K, Ruberson J. Red imported fire ant, Solenopsis invicta, modifies predation at the soil surface and in cotton foliage. Ann Appl Biol. 2016;169(3):319–28. doi: 10.1111/aab.12303

[7] Bertelsmeier C, Ollier S, Liebhold A, Keller L. Recent human history governs global ant invasion dynamics. Nat Ecol Evol. 2017;1(7):0184. doi: 10.1038/s41559-017-0184 28685166 PMC5495171

[8] Ascunce MS, Yang C-C, Oakey J, Calcaterra L, Wu W-J, Shih C-J, et al. Global invasion history of the fire ant Solenopsis invicta. Science. 2011;331(6020):1066–8. doi: 10.1126/science.1198734 21350177

[9] Zeng L, Lu YY, He XF, Zhang WQ, Liang GW. Identification of red imported fire ant Solenopsis invicta to invade mainland China and infestation in Wuchuan, Guangdong. Chin J Appl Entomol. 2005;42(2):144–8. https://kns.cnki.net/kcms2/article/abstract?v=PAev8JwjQiv11r-GD2cvmjWO_Z5kl8arMIkzdnoOow6RyX6QcFGtQk4JPbvJE_Vf0HMvqQqaeHayHU0aQHuzOXCCrYttna56muY90b45lJfSZR7C15W4pE6PWgewC0cA2IMTYpYiFvt_uiVqtBZfmeK-VSK6ezbFBi37VF4nLBcsy2uBR0Ibdk6-iiHRZHxVrGUaXX0Vao=&uniplatform=NZKPT&language=CHS in Chinese.

[10] Ministry of Agriculture and Rural Affairs. List of national agricultural plant quarantine pests distribution administrative areas. Ministry of Agriculture and Rural Affairs. https://zzys.moa.gov.cn/tzgg/202507/t20250730_6476125.htm Accessed 2023 October 23. Feb 2026. in Chinese. 2026.

[11] Lu YY. Long-distance spreading speed and trend prediction of red imported fire ant, Solenopsis invicta Buren, in mainland China. Guangdong Agricultural Science. 2014;41(10):70–2. doi: 10.16768/j.issn.1004-874x.2014.10.018

[12] Wang L, Chen KW, Feng XD, Wang XL, Lu YY. Long-term prediction of red imported fire ant (Solenopsis invicta Buren) expansion in Chinese mainland. J Environ Entomol. 2022;44(2):339–44. https://link.cnki.net/urlid/44.1640.Q.20220519.1550.006 in Chinese.

[13] Vinson SB. Invasion of red imported fire ant (Hymenoptera: Formicidae): spread, biology, and impact. Am Entomol. 1997;43(1):23–39. https://academic.oup.com/aesa/article/81/6/913/9372?login=true

[14] Hoffman DR, Jacobson RS, Schmidt M, Smith AM. Allergens in Hymenoptera venoms. XXIII. Venom content of imported fire ant whole body extracts. Ann Allergy. 1991;66(1):29–31. https://pubmed.ncbi.nlm.nih.gov/1987866/1987866

[15] Caldwell ST, Schuman SH, Simpson WM Jr. Fire ants: a continuing community health threat in South Carolina. J S C Med Assoc. 1999;95(6):231–5. https://pubmed.ncbi.nlm.nih.gov/10389385/ 10389385

[16] Zamith-Miranda D, Fox EGP, Monteiro AP, Gama D, Poublan LE, de Araujo AF, et al. The allergic response mediated by fire ant venom proteins. Sci Rep. 2018;8(1):14427. doi: 10.1038/s41598-018-32327-z 30258210 PMC6158280

[17] Adams CT, Lofgren CS. Red imported fire ants (Hymenoptera: Formicidae): frequency of sting attacks on residents of Sumter County, Georgia. J Med Entomol. 1981;18(5):378–82. doi: 10.1093/jmedent/18.5.378 7299792

[18] Koya S, Crenshaw D, Agarwal A. Rhabdomyolysis and acute renal failure after fire ant bites. J Gen Intern Med. 2007;22(1):145–7. doi: 10.1007/s11606-006-0025-z 17351856 PMC1824724

[19] Candiotti KA, Lamas AM. Adverse neurologic reactions to the sting of the imported fire ant. Int Arch Allergy Immunol. 1993;102(4):417–20. doi: 10.1159/000236592 8241804

[20] Chan KH, Guénard B. Ecological and socio-economic impacts of the red import fire ant, Solenopsis invicta (Hymenoptera: Formicidae), on urban agricultural ecosystems. Urban Ecosyst. 2019;23(1):1–12. doi: 10.1007/s11252-019-00893-3

[21] Hong JX. Diversity and functional analysis of common ant viruses in Guangzhou. Thesis for Master Degree, South China Agricultural University, 2025. Guangzhou. in Chinese.

[22] Hoffman DR. Hymenoptera venom allergens. Clin Rev Allergy Immunol. 2006;30(2):109–28. doi: 10.1385/criai:30:2:109 16645223

[23] de Shazo JR, Williams D, Goddard J, Rockhold R, Kemp S. Stings of imported fire ants: Clinical manifestations, diagnosis, and treatment. Up to Date. https://sso.uptodate.com/contents/search 2023.

[24] deShazo RD, Banks WA. Medical consequences of multiple fire ant stings occurring indoors. J Allergy Clin Immunol. 1994;93(5):847–50. doi: 10.1016/0091-6749(94)90376-x 8182226

[25] Kemp SF, deShazo RD, Moffitt JE, Williams DF, Buhner WA 2nd. Expanding habitat of the imported fire ant (Solenopsis invicta): a public health concern. J Allergy Clin Immunol. 2000;105(4):683–91. doi: 10.1067/mai.2000.105707 10756216

[26] Goddard J, de Shazo RD. Envenomation From Flood-Related Fire Ant Rafting: A Cautionary Note. Am J Med. 2023;136(9):937–40. doi: 10.1016/j.amjmed.2023.06.002 37355195

[27] Wanandy T, Mulcahy E, Lau WY, Brown SGA, Wiese MD. Global View on Ant Venom Allergy: from Allergenic Components to Clinical Management. Clin Rev Allergy Immunol. 2022;62(1):123–44. doi: 10.1007/s12016-021-08858-1 34075569

[28] Pereira R, Williams D, Davis T, Qi D, Bolton H, Horton P. Imported fire ants and their management. Environ Sci. 2006. https://api.semanticscholar.org/CorpusID:13468437

[29] Wang L, Zeng L, Xu Y, Lu Y. Prevalence and management of Solenopsis invicta in China. NB. 2020;54:89–124. doi: 10.3897/neobiota.54.38584

[30] Liu Y-S, Huang S-A, Lin I-L, Lin C-C, Lai H-K, Yang C-H, et al. Establishment and Social Impacts of the Red Imported Fire Ant, Solenopsis invicta, (Hymenoptera: Formicidae) in Taiwan. Int J Environ Res Public Health. 2021;18(10):5055. doi: 10.3390/ijerph18105055 34064690 PMC8151706

[31] Wu NJ, Lu WC, Luo HM, He DZ, He JF, Liang KB, et al. A survey on human bitten by red imported fire ants in mainland for the first time. Chin J Vector Biol Control. 2005;16(5):14–6. https://kns.cnki.net/kcms2/article/abstract?v=PAev8JwjQit_jmvoHCeu6DCPgw_I5M9WsZSHZy2nQ4ZEMOh1DObYCZrkjINxXXlR-Eqt-HPSj4j3JGhxJXkji_elUIgelCNpzTjhG-BiwPCvkSrBZx_eF8KE00UUXcrTKxvqh7I0o3dZqL9JGIlli64NQCvfKiDd54AXKA-9wFMK6HMVZIYVRRapNzIQDV0&uniplatform=NZKPT&language=CHS. in Chinese.

[32] Xu Y, Huang J, Zhou A, Zeng L. Prevalence of Solenopsis invicta (Hymenoptera: Formicidae) venom allergic reactions in mainland China. Fla Entomol. 2012;95(4):961–5. doi: 10.1653/024.095.0421

[33] Stafford CT, Hutto LS, Rhoades RB, Thompson WO, Impson LK. Imported fire ant as a health hazard. South Med J. 1989;82(12):1515–9. doi: 10.1097/00007611-198912000-00014 2595421

[34] Hoffman DR. Fire ant venom allergy. Allergy. 1995;50(7):535–44. doi: 10.1111/j.1398-9995.1995.tb01196.x 8588684

[35] deShazo RD, Kemp SF, deShazo MD, Goddard J. Fire ant attacks on patients in nursing homes: an increasing problem. Am J Med. 2004;116(12):843–6. doi: 10.1016/j.amjmed.2004.02.026 15178500

[36] Solley GO, Vanderwoude C, Knight GK. Anaphylaxis due to Red Imported Fire Ant sting. Med J Aust. 2002;176(11):521–3. doi: 10.5694/j.1326-5377.2002.tb04548.x 12064982

[37] Triplett RF. The imported fire ant: health hazard or nuisance? South Med J. 1976;69(3):258–9. https://pubmed.ncbi.nlm.nih.gov/1257814 1257814

[38] Zhao JN, Xu YJ. Survey of the prevalence of fire ant sting accidents based on internet reports. Chin J Appl Entomol. 2015;52(6):1409–12. https://kns.cnki.net/kcms2/article/abstract?v=PAev8JwjQiv1MAQK0Bq0SdQW2oWuD25HMf_FW46PBsj4M_w7DcaM6fYLdCQ8HCHxixTASXoFx-ky_H6Q8BiB359U5D0XficoP9VOuI8HMW7MMO7zmaQJn8MEDPLCk3gFlhIIOp4o52nE2Mx1TWRZk0cE59kLNvdYXTJk2yOgHIr76eb_cuvbkqO1P-HLC6B0pmvkIz56sPM=&uniplatform=NZKPT&language=CHS in Chinese.

[39] Epidemic report, annual report, Plant Protection and Plant Inspection Information Management System. 2023. https://zbzj.agri.cn/pq/pq_index

[40] Seventh National Census Data. http://www.stats.gov.cn/tjsj/ndsj/ 2023.

[41] Lopez DJ, Winkel KD, Wanandy T, van Nunen S, Perrett KP, Lowe AJ. The Human Health Impacts of the Red Imported Fire Ant in the Western Pacific Region Context: A Narrative Review. Trop Med Infect Dis. 2024;9(4):69. doi: 10.3390/tropicalmed9040069 38668530 PMC11053531

[42] Yin CL, Gao HJ, Zhao XQ, Liu Y. Suitable area prediction of red imported fire ant in China based on current climate data. Plant Quarantine. 2024;38(1):72–8. doi: 10.19662/j.cnki.issn1005-2755.2024.01.010

[43] Fu QY, Song ZD, Zhao Y, Li SL, Xu YJ. Analysis on the control cost of Solenopsis invicta in China’s mainland. J Environ Entomol. 2022;44(2):345–51. https://link.cnki.net/urlid/44.1640.q.20210223.0815.002 in Chinese.

[44] Lu YY. Invasive expansion of red imported fire ant in China and countermeasures for prevention and control. Compend Annu Conf Anim Inj Prev Control. 2023;1:093871. doi: 10.26914/c.cnkihy.2023.093871

[45] Partridge ME, Blackwood W, Hamilton RG, Ford J, Young P, Ownby DR. Prevalence of allergic sensitization to imported fire ants in children living in an endemic region of the southeastern United States. Ann Allergy Asthma Immunol. 2008;100(1):54–8. doi: 10.1016/S1081-1206(10)60405-X 18254483

[46] Neaves BI, Coop CA. Imported fire ant immunotherapy. Ann Allergy Asthma Immunol. 2024;133(1):28–32. doi: 10.1016/j.anai.2024.01.014 38281676

[47] Hannan CJ Jr, Stafford CT, Rhoades RB, Wray BB, Baer H, Anderson MC. Seasonal variation in antigens of the imported fire ant Solenopsis invicta. J Allergy Clin Immunol. 1986;78(2):331–6. doi: 10.1016/s0091-6749(86)80085-9 3734284

[48] Guan D, Liao XL, Chen L. Comparative analysis of venomous alkaloids in workers of red imported fire ant, Solenopsis invicta (Hymenoptera. Formicidae). Acta Entomol Sin. 2013;56(4):365–71. doi: 10.16380/j.kcxb.2013.04.012

[49] Zhang HJ, Chen L, Wang WK. Comparison of venom alkaloids in different castes of individual ants of Solenopsis invicta Buren. J Environ Entomol. 2018;40(2):468–73. https://kns.cnki.net/kcms2/article/abstract?v=PAev8JwjQisCY8XPXMA9qGvfC-3deGZORPUAyPrjEhUNFZprgc_iry0ZMS6V6ZrRbxBl8LrbmQZIxX3X_1L11c2RyH42jbg3ZuztUhrI6IIirUurouRH65Cqtpf6HuQV5bbQeJN3moZUFpmPwMm6AzfAeDLYX0HAJpS3FkNt8eazXdZZHqEDkiargAwdt_Pyeqs-iUTJpQE=&uniplatform=NZKPT&language=CHS in Chinese.

[50] Antonicelli L, Bilò MB, Bonifazi F. Epidemiology of Hymenoptera allergy. Curr Opin Allergy Clin Immunol. 2002;2(4):341–6. doi: 10.1097/00130832-200208000-00008 12130949

[51] Chen XY, Ma P, Yu M, Li ZY, Huang ZH, Gui FR. Invasive risk analysis of Solenopsis invicta Buren using integrated multi-index evaluation system in Yunnan Province. J Biosaf. 2014;81–7. https://kns.cnki.net/kcms2/article/abstract?v=PAev8JwjQivPrsSpx_9IYkpoemvtG2TsyfpQGamODQibW19MEZIOmK0wmykX3IMqx0ORvw_O9CyQxSv8Tf41lP7ih6tZUW44AAuRiOFJEcfU3Xh8QuR8M_TgBQwYYbLj2o4-fZ5-5JPXSyacKF2oVqMrZRMH3N8engVZ6HEy1kbwhmU6O7STOMX5B6Tvw8zN&uniplatform=NZKPT&language=CHS in Chinese.

[52] Stafford CT. Hypersensitivity to fire ant venom. Ann Allergy Asthma Immunol. 1996;77(2):87–95; quiz 96–9. doi: 10.1016/S1081-1206(10)63493-X 8760773

[53] Wauters RH, Brooks DI, Schwartz DJ. Imported fire ant immunotherapy prescribing patterns in a large health care system during an 11-year period. Ann Allergy Asthma Immunol. 2020;125(5):577–80. doi: 10.1016/j.anai.2020.06.013 32544534

[54] Lin KC, Xie MZ, Yang XH, Li GH. Clinical observation on the treatment of red imported fire ant stings by internal and external application of Ji Desheng snake medicine tablets. Chin Naturopathy. 2021;29(18):86–8. doi: 10.19621/j.cnki.11-3555/r.2021.1832

[55] Xiao KS, Liang KB, Li Y, Zhu RX, Liang JH, Zhan YL, et al. Investigation report on red imported fire ant stings. Chin J Dermatol. 2006;7:415–6. https://kns.cnki.net/kcms2/article/abstract?v=PAev8JwjQiuIpisj1u8bI-Gnf9hKIO1xOAW-w-ewXkfW8LZJYVl22IkmfVuDs86nXCmkZfr_0x2cHfCe4y3WRyyz_VO3OPu65EQviVqgMPH_4SNF7hYLQYb1s_zOo1jz-_UDMy4MT2N68bn1KZDNqVyOuz5nojB3g_FSLExyAENIE2KXCUGZ0KFgDoXVX6f&uniplatform=NZKPT&language=CHS in Chinese.

[56] Xu GF, Sun LM, Wang HM. Investigation of a red imported fire ant injury incident. Occup Health. 2006;22(15):1190–1. https://kns.cnki.net/kcms2/article/abstract?v=PAev8JwjQithQhdqOWnOCA9Hg0uyh87V1STXR1fndfOgIvRo4BDV2LvogjhUcoLFYxMc9d4K7z9tleiJ0bfE6K9URxfcYS-Bs3qBie3IvtjCZZVHYxWNn1rmi84UHGpUybQLi72QJjlgcxltO3o2PBkR4JbtfQunI9kKdsd6bMq6dD2CpgZTIrd7PgJ5xAy0&uniplatform=NZKPT&language=CHS

[57] Zhang QL, Lin LF, Chen HT, Chen PH, Lu WC, Li YJ. An investigation on the first human death incident caused by the bite of red imported fire ant. Dis Surveill. 2006;21(12):654–6. https://kns.cnki.net/kcms2/article/abstract?v=PAev8JwjQitrDhAPmhQ7vcTiyZg7rfjr6xMLG-YxwhcorzQBUFlWQ9bGaWWp1Sd6EKZM-lxHvxWctTHreZsmzE7q8f9RuksFwkIqQMFgQqdro3-Rd8Ovah5Zd2Zt7BC77Z3ywIwCR0N0zBKAkTXiZ7pB1mrE2AoSncv7-W4UgL8CfY1S8egrIHZhWnC0VymK&uniplatform=NZKPT&language=CHS in Chinese.

[58] Lu WC, Han JY, Zhang QL, Chen HT, Liu WH, Lin LF, et al. An irritability shock case caused by red imported fire ant stinging. Chin J Vector Biol Control. 2007;18(2):105–6. https://kns.cnki.net/kcms2/article/abstract?v=PAev8JwjQivmBi_KCuF3lRR7bqspaijcSLMJ8eCYlPhfKFRoaJo45LZdzfHzTJfb1J4bUGo-DXM538zfsHLMrfOK0kCWItHru4aIqN9HXEqLw6-FJ2w3f506fg0V5Mz7ve0KGsPlLAK5X_m1SFn1I4nK8hmp3LcU-9SZ0eNmbCdZZjAU1twWHxa95niAXqFg&uniplatform=NZKPT&language=CHS in Chinese.

[59] Han JY, Lin LF, Lu WC, Yi JR, Zhang QL, Lu XP, et al. A survey on red imported fire ant bites in villagers and disease control in a village of Guangdong. Chin J Vector Biol Control. 2007;18(1):20–3. https://kns.cnki.net/kcms2/article/abstract?v=PAev8JwjQiugMUUcmfnc1yt1swZLteWoZ8F_x_a06yFx_yylnOBwoyJnAupzSWf2yUkAGVtCYX2ge5XH00ik4sGFZ0yzETnwca7KAYPR0FFcKMPuXC3hFKyJC0dYt-jvYw8CeDlqFj9kLnmeC-wlFLgNdzO48Wj6VwFmuWFBX9dCSvjx5kLxaRujWzHiZAPJ&uniplatform=NZKPT&language=CHS in Chinese.

[60] Yang XQ, Tan WL, Li YX. A clinical analysis of Solenopsis invicta Buren on 63 cases. Guide China Med. 2012;10(10):20–1. doi: 10.15912/j.cnki.gocm.2012.10.350

[61] Guo XP. The clinical of on RIFA stings clinical characteristics analysis and prevention. Guide China Med. 2015;13(7):135–6. doi: 10.15912/j.cnki.gocm.2015.07.097

[62] Li TX, Cai TT. New method for the treatment of skin damage caused by Solenopsis invicta Buren. J Clin Dermatol. 2019;48(4):210–2. doi: 10.16761/j.cnki.1000-4963.2019.04.006

[63] Wang C, Li SL, Lu YY. Discussion of management strategy and method for red imported fire ant based on cognitive level assessment. Guangdong Agric Sci. 2014;41(10):232–6. doi: 10.16768/j.issn.1004-874x.2014.10.022

[64] Mao RQ, Wu H, Huang H. Impacts of red imported fire ants on human health and preventive strategies. Occup Health Emerg Rescue. 2006;24(3):121–2.

[65] Zhang M, Hu F, Bi XL, Wang YL. Analysis of clinical data of 110 cases bited by fire ant on a reclaimed reef of South China Sea. China J Lepr Skin Dis. 2021;37(3):174–5. https://kns.cnki.net/kcms2/article/abstract?v=PAev8JwjQiuxr1KA_ciS29HCGEzTA0HadgqXp2bHl6Ham4e5w5IhlghtK2rTx-el7SW0gZqKvGlV0ngdw8MUHxMlCntz2HFBwzigfrUhkrJ3mBQnoo2S5Cvg7DKfiQudHIzhO6RSTDuS4cB_CJ4rZVaSWTlI3MM7oVTTy2psgv0btJuk8JD2-cyB8Rs7tRRX-GovcacHw=&uniplatform=NZKPT&language=CHS

[66] Wang YL, Yang LY, Fu XW, Wu JH. Clinical analysis of sting injury by red imported fire ant in 61 reef garrison officers and men. J Navy Med. 2022;43(2):129–31. https://kns.cnki.net/kcms2/article/abstract?v=PAev8JwjQisrvue2r7aDVyJms-JCxEMTrj5i9monzZhKbhdcJza68twMlYXJFHiQK_p0tgCjNw0FpsOhf2S3O-1As3tNCaH_-ig_wvdFV2g-JOEGMtPX_zNq76wcf7yIEghI8bIW1V19GiKxAvW8numnhFzNHKExLp4Md27F6f4v8ID4oQBx4wAvBGJE_7nGgWymkZGacKE=&uniplatform=NZKPT&language=CHS in Chinese.

[67] Qiao AH, Qu JL, Lv J, Sheng R, Fan JP, Xie J, et al. Emergency care of three cases of anaphylaxis due to red imported fire ant stings onan island reef. J Navy Med. 2022;43(5):472–474. https://kns.cnki.net/kcms2/article/abstract?v=PAev8JwjQiva2XtKzNkUUHPexTE8_dAeF8CkvQvHCm2yWoZ6Qf-xBd3Kt3ew3ZFfDewkzB_CkrpQy87H7V9CWlnQGHn4vaMGf6k5bgSZYmw36Nr-bA71TJ32nQmxMtLxNBQ4efsD-z9PcWaPPSve0gFXJqDwNOTVsXKIw9RWbodEHJNC8NqftYjiT7Hrgj1ObeTMbb7Z7Y=&uniplatform=NZKPT&language=CHS in Chinese.

[68] Tang LL. Clinical analysis of 66 cases of red imported fire ant stings. Chin J Rural Med Pharm. 2023;30(7):37–8. doi: 10.19542/j.cnki.1006-5180.007122

[69] Tang XL, Wu X, Luo Y, Ye L, Lv JJ, Xu LL. Study on the application of integrated nursing intervention of traditional Chinese and western medicine in patients with Solenopsis invicta Buren sting and bite. J Snake. 2022;34(2):259–61. https://kns.cnki.net/kcms2/article/abstract?v=PAev8JwjQitLLUwP-PuuHRnExpfWsDvqtN6ruFCuU0A4D1uev2HVmWxcWvacWzzg7U0izOt8gB6LGWxi9LJ5C3B93LQjVQnQX2k8iZM4QSJ9SS2qB6YdaPHbjtaRwO2h35Ly9kC0fm72f6j8yO7jmELSjml6wpKYAhVsHXaSLVRLcZoHefOL6VcfjCPscZsLFuXmuA2qE=&uniplatform=NZKPT&language=CHS in Chinese.

[70] Wang B, Hong SC, Hu QJ, Huang FD, Du J, Ma YJ. Med J Chin People’s Armed Police Forces. 2024;35(4):299–301. doi: 10.14010/j.cnki.wjyx.2024.04.008

