# Supplementary material for: Analysis of the human health threat caused by the red imported fire ant in mainland China
Source: PLoS One. 2026 Jun 16;21(6):e0350501. doi: 10.1371/journal.pone.0350501 (PMC13271487; doi:10.1371/journal.pone.0350501)
Supplement: S1 Table — (DOCX) [file pone.0350501.s001.docx]

**Table1 National raw data table on infestation severity and symptom prediction of red imported fire ant (RIFA).**

| Administrative division | 20-year census data | Area of RIFA infection zones by level (km^2^) | | | | | Total area of RIFA infection zones (km^2^) | Population density in RIFA infected areas(km^2^) | Prediction on the opulation of RIFA infection zones by level | | | | | Prediction on the total opulation of RIFA infection zones | Prediction on the RIFA-affected populatio-n | Prediction on the number of people with different symptoms after RIFA stings | | | | | | | | | |
| --- | --- | --- | --- | --- | --- | --- | --- | --- | --- | --- | --- | --- | --- | --- | --- | --- | --- | --- | --- | --- | --- | --- | --- | --- | --- |
|  |  |  |  |  |  |  |  |  |  |  |  |  |  |  |  | Itching and pain | Vesicles or pustules | Urticaria or papules | Fever | Dizziness and headache-s | Localized allergic lymphadeno-pathy | Systemi-c allergy | Speech impedimen-t | Shock | Death |
|  |  | Level 1 | Level 2 | Level 3 | Level 4 | Level 5 |  |  | Level 1 | Level 2 | Level 3 | Level 4 | Level 5 |  |  | 0.7754 | 0.3612 | 0.3333 | 0.0265 | 0.0198 | 0.0094 | 0.0752 | 0.0075 | 0.0117 | 0.0003 |
| **Zhejiang Province** | 64567588 | 6.9722 | 1.5496 | 0.3029 | 0.7752 | 0.0033 | 9.6032 | 618.04 | 4309.08 | 957.71 | 187.18 | 479.11 | 2.06 | 5935.14 | 1431.13 | 1109.70 | 516.92 | 476.99 | 37.92 | 28.34 | 13.45 | 107.62 | 10.73 | 16.74 | 0.43 |
| **Hangzhou City** | **11936010** | 0.0020 | 0.0314 | 0.0000 | 0.0000 | 0.0000 | 0.0334 | 742.69 | 1.49 | 23.32 | 0.00 | 0.00 | 0.00 | 24.81 | 7.22 | 5.60 | 2.61 | 2.41 | 0.19 | 0.14 | 0.07 | 0.54 | 0.05 | 0.08 | 0.00 |
| Shangcheng District | 1845444 | 0.0000 | 0.0000 | 0.0000 | 0.0000 | 0.0000 | 0.0000 | 15336.52 | 0.00 | 0.00 | 0.00 | 0.00 | 0.00 | 0.00 | 0.00 | 0.00 | 0.00 | 0.00 | 0.00 | 0.00 | 0.00 | 0.00 | 0.00 | 0.00 | 0.00 |
| Gongshu District | 1120985 | 0.0000 | 0.0000 | 0.0000 | 0.0000 | 0.0000 | 0.0000 | 11371.32 | 0.00 | 0.00 | 0.00 | 0.00 | 0.00 | 0.00 | 0.00 | 0.00 | 0.00 | 0.00 | 0.00 | 0.00 | 0.00 | 0.00 | 0.00 | 0.00 | 0.00 |
| Xihu District | 1112992 | 0.0000 | 0.0000 | 0.0000 | 0.0000 | 0.0000 | 0.0000 | 3595.98 | 0.00 | 0.00 | 0.00 | 0.00 | 0.00 | 0.00 | 0.00 | 0.00 | 0.00 | 0.00 | 0.00 | 0.00 | 0.00 | 0.00 | 0.00 | 0.00 | 0.00 |
| Binjiang District | 503859 | 0.0000 | 0.0000 | 0.0000 | 0.0000 | 0.0000 | 0.0000 | 7052.90 | 0.00 | 0.00 | 0.00 | 0.00 | 0.00 | 0.00 | 0.00 | 0.00 | 0.00 | 0.00 | 0.00 | 0.00 | 0.00 | 0.00 | 0.00 | 0.00 | 0.00 |
| Xiaoshan District | 2258872 | 0.0000 | 0.0314 | 0.0000 | 0.0000 | 0.0000 | 0.0314 | 2198.63 | 0.00 | 69.04 | 0.00 | 0.00 | 0.00 | 69.04 | 20.71 | 16.06 | 7.48 | 6.90 | 0.55 | 0.41 | 0.19 | 1.56 | 0.16 | 0.24 | 0.01 |
| Yuhang District | 2402514 | 0.0000 | 0.0000 | 0.0000 | 0.0000 | 0.0000 | 0.0000 | 2550.14 | 0.00 | 0.00 | 0.00 | 0.00 | 0.00 | 0.00 | 0.00 | 0.00 | 0.00 | 0.00 | 0.00 | 0.00 | 0.00 | 0.00 | 0.00 | 0.00 | 0.00 |
| Fuyang District | 832017 | 0.0000 | 0.0000 | 0.0000 | 0.0000 | 0.0000 | 0.0000 | 457.23 | 0.00 | 0.00 | 0.00 | 0.00 | 0.00 | 0.00 | 0.00 | 0.00 | 0.00 | 0.00 | 0.00 | 0.00 | 0.00 | 0.00 | 0.00 | 0.00 | 0.00 |
| Lin'an District | 634555 | 0.0000 | 0.0000 | 0.0000 | 0.0000 | 0.0000 | 0.0000 | 203.24 | 0.00 | 0.00 | 0.00 | 0.00 | 0.00 | 0.00 | 0.00 | 0.00 | 0.00 | 0.00 | 0.00 | 0.00 | 0.00 | 0.00 | 0.00 | 0.00 | 0.00 |
| Tonglu County | 453106 | 0.0000 | 0.0000 | 0.0000 | 0.0000 | 0.0000 | 0.0000 | 247.41 | 0.00 | 0.00 | 0.00 | 0.00 | 0.00 | 0.00 | 0.00 | 0.00 | 0.00 | 0.00 | 0.00 | 0.00 | 0.00 | 0.00 | 0.00 | 0.00 | 0.00 |
| Chun'an County | 328957 | 0.0020 | 0.0000 | 0.0000 | 0.0000 | 0.0000 | 0.0020 | 74.48 | 0.15 | 0.00 | 0.00 | 0.00 | 0.00 | 0.15 | 0.02 | 0.02 | 0.01 | 0.01 | 0.00 | 0.00 | 0.00 | 0.00 | 0.00 | 0.00 | 0.00 |
| Jiande City | 442709 | 0.0000 | 0.0000 | 0.0000 | 0.0000 | 0.0000 | 0.0000 | 191.50 | 0.00 | 0.00 | 0.00 | 0.00 | 0.00 | 0.00 | 0.00 | 0.00 | 0.00 | 0.00 | 0.00 | 0.00 | 0.00 | 0.00 | 0.00 | 0.00 | 0.00 |
| **Ningbo City** | **9404283** | 0.0000 | 0.1793 | 0.0000 | 0.0000 | 0.0000 | 0.1793 | 982.68 | 0.00 | 176.23 | 0.00 | 0.00 | 0.00 | 176.23 | 52.87 | 40.99 | 19.10 | 17.62 | 1.40 | 1.05 | 0.50 | 3.98 | 0.40 | 0.62 | 0.02 |
| Haishu District | 1041285 | 0.0000 | 0.1200 | 0.0000 | 0.0000 | 0.0000 | 0.1200 | 1733.60 | 0.00 | 208.03 | 0.00 | 0.00 | 0.00 | 208.03 | 62.41 | 48.39 | 22.54 | 20.80 | 1.65 | 1.24 | 0.59 | 4.69 | 0.47 | 0.73 | 0.02 |
| Jiangbei District | 488885 | 0.0000 | 0.0000 | 0.0000 | 0.0000 | 0.0000 | 0.0000 | 2402.74 | 0.00 | 0.00 | 0.00 | 0.00 | 0.00 | 0.00 | 0.00 | 0.00 | 0.00 | 0.00 | 0.00 | 0.00 | 0.00 | 0.00 | 0.00 | 0.00 | 0.00 |
| Beilun District | 829448 | 0.0000 | 0.0000 | 0.0000 | 0.0000 | 0.0000 | 0.0000 | 1371.31 | 0.00 | 0.00 | 0.00 | 0.00 | 0.00 | 0.00 | 0.00 | 0.00 | 0.00 | 0.00 | 0.00 | 0.00 | 0.00 | 0.00 | 0.00 | 0.00 | 0.00 |
| Zhenhai District | 531830 | 0.0000 | 0.0593 | 0.0000 | 0.0000 | 0.0000 | 0.0593 | 2172.86 | 0.00 | 128.92 | 0.00 | 0.00 | 0.00 | 128.92 | 38.68 | 29.99 | 13.97 | 12.89 | 1.02 | 0.77 | 0.36 | 2.91 | 0.29 | 0.45 | 0.01 |
| Yinzhou District | 1588187 | 0.0000 | 0.0000 | 0.0000 | 0.0000 | 0.0000 | 0.0000 | 1996.46 | 0.00 | 0.00 | 0.00 | 0.00 | 0.00 | 0.00 | 0.00 | 0.00 | 0.00 | 0.00 | 0.00 | 0.00 | 0.00 | 0.00 | 0.00 | 0.00 | 0.00 |
| Fenghua District | 577505 | 0.0000 | 0.0000 | 0.0000 | 0.0000 | 0.0000 | 0.0000 | 459.94 | 0.00 | 0.00 | 0.00 | 0.00 | 0.00 | 0.00 | 0.00 | 0.00 | 0.00 | 0.00 | 0.00 | 0.00 | 0.00 | 0.00 | 0.00 | 0.00 | 0.00 |
| Xiangshan County | 567665 | 0.0000 | 0.0000 | 0.0000 | 0.0000 | 0.0000 | 0.0000 | 462.46 | 0.00 | 0.00 | 0.00 | 0.00 | 0.00 | 0.00 | 0.00 | 0.00 | 0.00 | 0.00 | 0.00 | 0.00 | 0.00 | 0.00 | 0.00 | 0.00 | 0.00 |
| Ninghai County | 695958 | 0.0000 | 0.0000 | 0.0000 | 0.0000 | 0.0000 | 0.0000 | 406.59 | 0.00 | 0.00 | 0.00 | 0.00 | 0.00 | 0.00 | 0.00 | 0.00 | 0.00 | 0.00 | 0.00 | 0.00 | 0.00 | 0.00 | 0.00 | 0.00 | 0.00 |
| Yuyao City | 1254032 | 0.0000 | 0.0000 | 0.0000 | 0.0000 | 0.0000 | 0.0000 | 815.47 | 0.00 | 0.00 | 0.00 | 0.00 | 0.00 | 0.00 | 0.00 | 0.00 | 0.00 | 0.00 | 0.00 | 0.00 | 0.00 | 0.00 | 0.00 | 0.00 | 0.00 |
| Cixi City | 1829488 | 0.0000 | 0.0000 | 0.0000 | 0.0000 | 0.0000 | 0.0000 | 1317.89 | 0.00 | 0.00 | 0.00 | 0.00 | 0.00 | 0.00 | 0.00 | 0.00 | 0.00 | 0.00 | 0.00 | 0.00 | 0.00 | 0.00 | 0.00 | 0.00 | 0.00 |
| **Wenzhou City** | **9572903** | 1.1343 | 0.3618 | 0.0000 | 0.0000 | 0.0000 | 1.4961 | 819.43 | 929.51 | 296.47 | 0.00 | 0.00 | 0.00 | 1225.98 | 228.37 | 177.08 | 82.49 | 76.12 | 6.05 | 4.52 | 2.15 | 17.17 | 1.71 | 2.67 | 0.07 |
| Lucheng District | 1167165 | 0.0000 | 0.0000 | 0.0000 | 0.0000 | 0.0000 | 0.0000 | 3978.61 | 0.00 | 0.00 | 0.00 | 0.00 | 0.00 | 0.00 | 0.00 | 0.00 | 0.00 | 0.00 | 0.00 | 0.00 | 0.00 | 0.00 | 0.00 | 0.00 | 0.00 |
| Longwan District | 725036 | 0.5667 | 0.2307 | 0.0000 | 0.0000 | 0.0000 | 0.7973 | 2474.86 | 1402.42 | 570.87 | 0.00 | 0.00 | 0.00 | 1973.29 | 381.62 | 295.91 | 137.84 | 127.20 | 10.11 | 7.56 | 3.59 | 28.70 | 2.86 | 4.47 | 0.11 |
| Ouhai District | 963238 | 0.0457 | 0.0000 | 0.0000 | 0.0000 | 0.0000 | 0.0457 | 2065.26 | 94.45 | 0.00 | 0.00 | 0.00 | 0.00 | 94.45 | 14.17 | 10.99 | 5.12 | 4.72 | 0.38 | 0.28 | 0.13 | 1.07 | 0.11 | 0.17 | 0.00 |
| Dongtou District | 148807 | 0.1240 | 0.0000 | 0.0000 | 0.0000 | 0.0000 | 0.1240 | 848.24 | 105.18 | 0.00 | 0.00 | 0.00 | 0.00 | 105.18 | 15.78 | 12.23 | 5.70 | 5.26 | 0.42 | 0.31 | 0.15 | 1.19 | 0.12 | 0.18 | 0.00 |
| Yongjia County | 869624 | 0.0000 | 0.0000 | 0.0000 | 0.0000 | 0.0000 | 0.0000 | 324.77 | 0.00 | 0.00 | 0.00 | 0.00 | 0.00 | 0.00 | 0.00 | 0.00 | 0.00 | 0.00 | 0.00 | 0.00 | 0.00 | 0.00 | 0.00 | 0.00 | 0.00 |
| Pingyang County | 863088 | 0.1800 | 0.0000 | 0.0000 | 0.0000 | 0.0000 | 0.1800 | 894.26 | 160.97 | 0.00 | 0.00 | 0.00 | 0.00 | 160.97 | 24.15 | 18.72 | 8.72 | 8.05 | 0.64 | 0.48 | 0.23 | 1.82 | 0.18 | 0.28 | 0.01 |
| Cangnan County | 843962 | 0.0259 | 0.0643 | 0.0000 | 0.0000 | 0.0000 | 0.0902 | 820.02 | 21.27 | 52.70 | 0.00 | 0.00 | 0.00 | 73.97 | 19.00 | 14.73 | 6.86 | 6.33 | 0.50 | 0.38 | 0.18 | 1.43 | 0.14 | 0.22 | 0.01 |
| Wencheng County | 288178 | 0.0000 | 0.0000 | 0.0000 | 0.0000 | 0.0000 | 0.0000 | 222.15 | 0.00 | 0.00 | 0.00 | 0.00 | 0.00 | 0.00 | 0.00 | 0.00 | 0.00 | 0.00 | 0.00 | 0.00 | 0.00 | 0.00 | 0.00 | 0.00 | 0.00 |
| Taishun County | 265980 | 0.0000 | 0.0000 | 0.0000 | 0.0000 | 0.0000 | 0.0000 | 150.53 | 0.00 | 0.00 | 0.00 | 0.00 | 0.00 | 0.00 | 0.00 | 0.00 | 0.00 | 0.00 | 0.00 | 0.00 | 0.00 | 0.00 | 0.00 | 0.00 | 0.00 |
| Ruian City | 1520386 | 0.0000 | 0.0669 | 0.0000 | 0.0000 | 0.0000 | 0.0669 | 1196.12 | 0.00 | 79.98 | 0.00 | 0.00 | 0.00 | 79.98 | 23.99 | 18.61 | 8.67 | 8.00 | 0.64 | 0.48 | 0.23 | 1.80 | 0.18 | 0.28 | 0.01 |
| Yueqing City | 1452707 | 0.0000 | 0.0000 | 0.0000 | 0.0000 | 0.0000 | 0.0000 | 1126.83 | 0.00 | 0.00 | 0.00 | 0.00 | 0.00 | 0.00 | 0.00 | 0.00 | 0.00 | 0.00 | 0.00 | 0.00 | 0.00 | 0.00 | 0.00 | 0.00 | 0.00 |
| Longgang City | 464732 | 0.1920 | 0.0000 | 0.0000 | 0.0000 | 0.0000 | 0.1920 | 2946.00 | 565.63 | 0.00 | 0.00 | 0.00 | 0.00 | 565.63 | 84.84 | 65.79 | 30.65 | 28.28 | 2.25 | 1.68 | 0.80 | 6.38 | 0.64 | 0.99 | 0.03 |
| **Jiaxing City** | **5400868** | 0.0787 | 0.2012 | 0.0000 | 0.0000 | 0.0000 | 0.2799 | 1097.63 | 86.35 | 220.84 | 0.00 | 0.00 | 0.00 | 307.19 | 79.21 | 61.42 | 28.61 | 26.40 | 2.10 | 1.57 | 0.74 | 5.96 | 0.59 | 0.93 | 0.02 |
| Nanhu District | 839433 | 0.0000 | 0.1327 | 0.0000 | 0.0000 | 0.0000 | 0.1327 | 1911.41 | 0.00 | 253.58 | 0.00 | 0.00 | 0.00 | 253.58 | 76.07 | 58.99 | 27.48 | 25.36 | 2.02 | 1.51 | 0.72 | 5.72 | 0.57 | 0.89 | 0.02 |
| Xiuzhou District | 679221 | 0.0000 | 0.0000 | 0.0000 | 0.0000 | 0.0000 | 0.0000 | 1240.81 | 0.00 | 0.00 | 0.00 | 0.00 | 0.00 | 0.00 | 0.00 | 0.00 | 0.00 | 0.00 | 0.00 | 0.00 | 0.00 | 0.00 | 0.00 | 0.00 | 0.00 |
| Jiashan County | 648160 | 0.0787 | 0.0000 | 0.0000 | 0.0000 | 0.0000 | 0.0787 | 1278.55 | 100.58 | 0.00 | 0.00 | 0.00 | 0.00 | 100.58 | 15.09 | 11.70 | 5.45 | 5.03 | 0.40 | 0.30 | 0.14 | 1.13 | 0.11 | 0.18 | 0.00 |
| Haiyan County | 456775 | 0.0000 | 0.0000 | 0.0000 | 0.0000 | 0.0000 | 0.0000 | 419.41 | 0.00 | 0.00 | 0.00 | 0.00 | 0.00 | 0.00 | 0.00 | 0.00 | 0.00 | 0.00 | 0.00 | 0.00 | 0.00 | 0.00 | 0.00 | 0.00 | 0.00 |
| Haining City | 1076199 | 0.0000 | 0.0685 | 0.0000 | 0.0000 | 0.0000 | 0.0685 | 1244.20 | 0.00 | 85.27 | 0.00 | 0.00 | 0.00 | 85.27 | 25.58 | 19.84 | 9.24 | 8.53 | 0.68 | 0.51 | 0.24 | 1.92 | 0.19 | 0.30 | 0.01 |
| Pinghu City | 671326 | 0.0000 | 0.0000 | 0.0000 | 0.0000 | 0.0000 | 0.0000 | 901.01 | 0.00 | 0.00 | 0.00 | 0.00 | 0.00 | 0.00 | 0.00 | 0.00 | 0.00 | 0.00 | 0.00 | 0.00 | 0.00 | 0.00 | 0.00 | 0.00 | 0.00 |
| Tongxiang City | 1029754 | 0.0000 | 0.0000 | 0.0000 | 0.0000 | 0.0000 | 0.0000 | 1414.87 | 0.00 | 0.00 | 0.00 | 0.00 | 0.00 | 0.00 | 0.00 | 0.00 | 0.00 | 0.00 | 0.00 | 0.00 | 0.00 | 0.00 | 0.00 | 0.00 | 0.00 |
| **Huzhou City** | **3367579** | 0.0017 | 0.0000 | 0.0000 | 0.0000 | 0.0000 | 0.0017 | 579.05 | 0.97 | 0.00 | 0.00 | 0.00 | 0.00 | 0.97 | 0.14 | 0.11 | 0.05 | 0.05 | 0.00 | 0.00 | 0.00 | 0.01 | 0.00 | 0.00 | 0.00 |
| Wuxing District | 1015937 | 0.0000 | 0.0000 | 0.0000 | 0.0000 | 0.0000 | 0.0000 | 1178.77 | 0.00 | 0.00 | 0.00 | 0.00 | 0.00 | 0.00 | 0.00 | 0.00 | 0.00 | 0.00 | 0.00 | 0.00 | 0.00 | 0.00 | 0.00 | 0.00 | 0.00 |
| Nanxun District | 542889 | 0.0000 | 0.0000 | 0.0000 | 0.0000 | 0.0000 | 0.0000 | 774.13 | 0.00 | 0.00 | 0.00 | 0.00 | 0.00 | 0.00 | 0.00 | 0.00 | 0.00 | 0.00 | 0.00 | 0.00 | 0.00 | 0.00 | 0.00 | 0.00 | 0.00 |
| Deqing County | 548568 | 0.0017 | 0.0000 | 0.0000 | 0.0000 | 0.0000 | 0.0017 | 585.83 | 0.98 | 0.00 | 0.00 | 0.00 | 0.00 | 0.98 | 0.15 | 0.11 | 0.05 | 0.05 | 0.00 | 0.00 | 0.00 | 0.01 | 0.00 | 0.00 | 0.00 |
| Changxing County | 673776 | 0.0000 | 0.0000 | 0.0000 | 0.0000 | 0.0000 | 0.0000 | 471.30 | 0.00 | 0.00 | 0.00 | 0.00 | 0.00 | 0.00 | 0.00 | 0.00 | 0.00 | 0.00 | 0.00 | 0.00 | 0.00 | 0.00 | 0.00 | 0.00 | 0.00 |
| Anji County | 586409 | 0.0000 | 0.0000 | 0.0000 | 0.0000 | 0.0000 | 0.0000 | 310.84 | 0.00 | 0.00 | 0.00 | 0.00 | 0.00 | 0.00 | 0.00 | 0.00 | 0.00 | 0.00 | 0.00 | 0.00 | 0.00 | 0.00 | 0.00 | 0.00 | 0.00 |
| **Shaoxing City** | 5270977 | 0.0000 | 0.0000 | 0.0000 | 0.0000 | 0.0000 | 0.0000 | 636.97 | 0.00 | 0.00 | 0.00 | 0.00 | 0.00 | 0.00 | 0.00 | 0.00 | 0.00 | 0.00 | 0.00 | 0.00 | 0.00 | 0.00 | 0.00 | 0.00 | 0.00 |
| Yuecheng District | 1020037 | 0.0000 | 0.0000 | 0.0000 | 0.0000 | 0.0000 | 0.0000 | 1574.13 | 0.00 | 0.00 | 0.00 | 0.00 | 0.00 | 0.00 | 0.00 | 0.00 | 0.00 | 0.00 | 0.00 | 0.00 | 0.00 | 0.00 | 0.00 | 0.00 | 0.00 |
| Keqiao District | 1098859 | 0.0000 | 0.0000 | 0.0000 | 0.0000 | 0.0000 | 0.0000 | 1028.41 | 0.00 | 0.00 | 0.00 | 0.00 | 0.00 | 0.00 | 0.00 | 0.00 | 0.00 | 0.00 | 0.00 | 0.00 | 0.00 | 0.00 | 0.00 | 0.00 | 0.00 |
| Shangyu District | 839747 | 0.0000 | 0.0000 | 0.0000 | 0.0000 | 0.0000 | 0.0000 | 674.17 | 0.00 | 0.00 | 0.00 | 0.00 | 0.00 | 0.00 | 0.00 | 0.00 | 0.00 | 0.00 | 0.00 | 0.00 | 0.00 | 0.00 | 0.00 | 0.00 | 0.00 |
| Xinchang County | 419036 | 0.0000 | 0.0000 | 0.0000 | 0.0000 | 0.0000 | 0.0000 | 345.23 | 0.00 | 0.00 | 0.00 | 0.00 | 0.00 | 0.00 | 0.00 | 0.00 | 0.00 | 0.00 | 0.00 | 0.00 | 0.00 | 0.00 | 0.00 | 0.00 | 0.00 |
| Zhuji City | 1218072 | 0.0000 | 0.0000 | 0.0000 | 0.0000 | 0.0000 | 0.0000 | 526.94 | 0.00 | 0.00 | 0.00 | 0.00 | 0.00 | 0.00 | 0.00 | 0.00 | 0.00 | 0.00 | 0.00 | 0.00 | 0.00 | 0.00 | 0.00 | 0.00 | 0.00 |
| Shengzhou City | 675226 | 0.0000 | 0.0000 | 0.0000 | 0.0000 | 0.0000 | 0.0000 | 377.73 | 0.00 | 0.00 | 0.00 | 0.00 | 0.00 | 0.00 | 0.00 | 0.00 | 0.00 | 0.00 | 0.00 | 0.00 | 0.00 | 0.00 | 0.00 | 0.00 | 0.00 |
| **Jinhua City** | **7050683** | 4.3207 | 0.5239 | 0.1962 | 0.6379 | 0.0000 | 5.6787 | 644.13 | 2783.12 | 337.44 | 126.38 | 410.88 | 0.00 | 3657.81 | 923.23 | 715.87 | 333.47 | 307.71 | 24.47 | 18.28 | 8.68 | 69.43 | 6.92 | 10.80 | 0.28 |
| Wucheng District | 957055 | 2.9191 | 0.0000 | 0.0000 | 0.0000 | 0.0000 | 2.9191 | 688.43 | 2009.57 | 0.00 | 0.00 | 0.00 | 0.00 | 2009.57 | 301.44 | 233.73 | 108.88 | 100.47 | 7.99 | 5.97 | 2.83 | 22.67 | 2.26 | 3.53 | 0.09 |
| Jindong District | 506935 | 1.3690 | 0.3257 | 0.0000 | 0.0000 | 0.0000 | 1.6947 | 769.19 | 1053.02 | 250.55 | 0.00 | 0.00 | 0.00 | 1303.57 | 233.12 | 180.76 | 84.20 | 77.70 | 6.18 | 4.62 | 2.19 | 17.53 | 1.75 | 2.73 | 0.07 |
| Wuyi County | 462462 | 0.0000 | 0.0000 | 0.0000 | 0.0000 | 0.0000 | 0.0000 | 294.58 | 0.00 | 0.00 | 0.00 | 0.00 | 0.00 | 0.00 | 0.00 | 0.00 | 0.00 | 0.00 | 0.00 | 0.00 | 0.00 | 0.00 | 0.00 | 0.00 | 0.00 |
| Pujiang County | 460726 | 0.0000 | 0.0000 | 0.0000 | 0.0000 | 0.0000 | 0.0000 | 502.05 | 0.00 | 0.00 | 0.00 | 0.00 | 0.00 | 0.00 | 0.00 | 0.00 | 0.00 | 0.00 | 0.00 | 0.00 | 0.00 | 0.00 | 0.00 | 0.00 | 0.00 |
| Pan'an County | 177161 | 0.0000 | 0.0000 | 0.0000 | 0.0000 | 0.0000 | 0.0000 | 148.10 | 0.00 | 0.00 | 0.00 | 0.00 | 0.00 | 0.00 | 0.00 | 0.00 | 0.00 | 0.00 | 0.00 | 0.00 | 0.00 | 0.00 | 0.00 | 0.00 | 0.00 |
| Lanxi City | 574801 | 0.0000 | 0.0000 | 0.0000 | 0.0000 | 0.0000 | 0.0000 | 437.71 | 0.00 | 0.00 | 0.00 | 0.00 | 0.00 | 0.00 | 0.00 | 0.00 | 0.00 | 0.00 | 0.00 | 0.00 | 0.00 | 0.00 | 0.00 | 0.00 | 0.00 |
| Yiwu City | 1859390 | 0.0000 | 0.0000 | 0.0533 | 0.6304 | 0.0000 | 0.6837 | 1682.40 | 0.00 | 0.00 | 89.73 | 1060.61 | 0.00 | 1150.34 | 902.32 | 699.66 | 325.92 | 300.74 | 23.91 | 17.87 | 8.48 | 67.85 | 6.77 | 10.56 | 0.27 |
| Dongyang City | 1087950 | 0.0327 | 0.1981 | 0.1429 | 0.0075 | 0.0000 | 0.3811 | 622.75 | 20.34 | 123.39 | 88.97 | 4.65 | 0.00 | 237.35 | 97.17 | 75.35 | 35.10 | 32.39 | 2.58 | 1.92 | 0.91 | 7.31 | 0.73 | 1.14 | 0.03 |
| Yongkang City | 964203 | 0.0000 | 0.0000 | 0.0000 | 0.0000 | 0.0000 | 0.0000 | 920.39 | 0.00 | 0.00 | 0.00 | 0.00 | 0.00 | 0.00 | 0.00 | 0.00 | 0.00 | 0.00 | 0.00 | 0.00 | 0.00 | 0.00 | 0.00 | 0.00 | 0.00 |
| **Quzhou City** | **2276184** | 1.0493 | 0.0000 | 0.0000 | 0.0000 | 0.0000 | 1.0493 | 257.38 | 270.08 | 0.00 | 0.00 | 0.00 | 0.00 | 270.08 | 40.51 | 31.41 | 14.63 | 13.50 | 1.07 | 0.80 | 0.38 | 3.05 | 0.30 | 0.47 | 0.01 |
| Kecheng District | 528847 | 0.0000 | 0.0000 | 0.0000 | 0.0000 | 0.0000 | 0.0000 | 870.50 | 0.00 | 0.00 | 0.00 | 0.00 | 0.00 | 0.00 | 0.00 | 0.00 | 0.00 | 0.00 | 0.00 | 0.00 | 0.00 | 0.00 | 0.00 | 0.00 | 0.00 |
| Qujiang District | 373920 | 0.0000 | 0.0000 | 0.0000 | 0.0000 | 0.0000 | 0.0000 | 213.90 | 0.00 | 0.00 | 0.00 | 0.00 | 0.00 | 0.00 | 0.00 | 0.00 | 0.00 | 0.00 | 0.00 | 0.00 | 0.00 | 0.00 | 0.00 | 0.00 | 0.00 |
| Changshan County | 259966 | 0.0000 | 0.0000 | 0.0000 | 0.0000 | 0.0000 | 0.0000 | 237.02 | 0.00 | 0.00 | 0.00 | 0.00 | 0.00 | 0.00 | 0.00 | 0.00 | 0.00 | 0.00 | 0.00 | 0.00 | 0.00 | 0.00 | 0.00 | 0.00 | 0.00 |
| Kaihua County | 258810 | 0.0000 | 0.0000 | 0.0000 | 0.0000 | 0.0000 | 0.0000 | 116.00 | 0.00 | 0.00 | 0.00 | 0.00 | 0.00 | 0.00 | 0.00 | 0.00 | 0.00 | 0.00 | 0.00 | 0.00 | 0.00 | 0.00 | 0.00 | 0.00 | 0.00 |
| Longyou County | 360229 | 1.0493 | 0.0000 | 0.0000 | 0.0000 | 0.0000 | 1.0493 | 315.71 | 331.29 | 0.00 | 0.00 | 0.00 | 0.00 | 331.29 | 49.69 | 38.53 | 17.95 | 16.56 | 1.32 | 0.98 | 0.47 | 3.74 | 0.37 | 0.58 | 0.01 |
| Jiangshan City | 494412 | 0.0000 | 0.0000 | 0.0000 | 0.0000 | 0.0000 | 0.0000 | 244.86 | 0.00 | 0.00 | 0.00 | 0.00 | 0.00 | 0.00 | 0.00 | 0.00 | 0.00 | 0.00 | 0.00 | 0.00 | 0.00 | 0.00 | 0.00 | 0.00 | 0.00 |
| **Zhoushan City** | 1157817 | 0.0000 | 0.0000 | 0.0000 | 0.0000 | 0.0000 | 0.0000 | 839.67 | 0.00 | 0.00 | 0.00 | 0.00 | 0.00 | 0.00 | 0.00 | 0.00 | 0.00 | 0.00 | 0.00 | 0.00 | 0.00 | 0.00 | 0.00 | 0.00 | 0.00 |
| Dinghai District | 500030 | 0.0000 | 0.0000 | 0.0000 | 0.0000 | 0.0000 | 0.0000 | 893.64 | 0.00 | 0.00 | 0.00 | 0.00 | 0.00 | 0.00 | 0.00 | 0.00 | 0.00 | 0.00 | 0.00 | 0.00 | 0.00 | 0.00 | 0.00 | 0.00 | 0.00 |
| Putuo District | 382902 | 0.0000 | 0.0000 | 0.0000 | 0.0000 | 0.0000 | 0.0000 | 874.72 | 0.00 | 0.00 | 0.00 | 0.00 | 0.00 | 0.00 | 0.00 | 0.00 | 0.00 | 0.00 | 0.00 | 0.00 | 0.00 | 0.00 | 0.00 | 0.00 | 0.00 |
| Daishan County | 207982 | 0.0000 | 0.0000 | 0.0000 | 0.0000 | 0.0000 | 0.0000 | 695.89 | 0.00 | 0.00 | 0.00 | 0.00 | 0.00 | 0.00 | 0.00 | 0.00 | 0.00 | 0.00 | 0.00 | 0.00 | 0.00 | 0.00 | 0.00 | 0.00 | 0.00 |
| Shengsi County | 66903 | 0.0000 | 0.0000 | 0.0000 | 0.0000 | 0.0000 | 0.0000 | 808.59 | 0.00 | 0.00 | 0.00 | 0.00 | 0.00 | 0.00 | 0.00 | 0.00 | 0.00 | 0.00 | 0.00 | 0.00 | 0.00 | 0.00 | 0.00 | 0.00 | 0.00 |
| **Taizhou City** | **6622888** | 0.2287 | 0.2520 | 0.1067 | 0.1373 | 0.0033 | 0.7280 | 683.22 | 156.23 | 172.17 | 72.88 | 93.83 | 2.28 | 497.38 | 195.92 | 151.92 | 70.77 | 65.30 | 5.19 | 3.88 | 1.84 | 14.73 | 1.47 | 2.29 | 0.06 |
| Jiaojiang District | 826074 | 0.0000 | 0.0000 | 0.0000 | 0.0000 | 0.0000 | 0.0000 | 2496.22 | 0.00 | 0.00 | 0.00 | 0.00 | 0.00 | 0.00 | 0.00 | 0.00 | 0.00 | 0.00 | 0.00 | 0.00 | 0.00 | 0.00 | 0.00 | 0.00 | 0.00 |
| Huangyan District | 707453 | 0.0000 | 0.0167 | 0.0000 | 0.0000 | 0.0000 | 0.0167 | 715.54 | 0.00 | 11.93 | 0.00 | 0.00 | 0.00 | 11.93 | 3.58 | 2.77 | 1.29 | 1.19 | 0.09 | 0.07 | 0.03 | 0.27 | 0.03 | 0.04 | 0.00 |
| Luqiao District | 628934 | 0.0000 | 0.2353 | 0.1067 | 0.1373 | 0.0000 | 0.4793 | 2012.98 | 0.00 | 473.72 | 214.72 | 276.45 | 0.00 | 964.89 | 492.11 | 381.58 | 177.75 | 164.02 | 13.04 | 9.74 | 4.63 | 37.01 | 3.69 | 5.76 | 0.15 |
| Sanmen County | 379469 | 0.0000 | 0.0000 | 0.0000 | 0.0000 | 0.0000 | 0.0000 | 373.13 | 0.00 | 0.00 | 0.00 | 0.00 | 0.00 | 0.00 | 0.00 | 0.00 | 0.00 | 0.00 | 0.00 | 0.00 | 0.00 | 0.00 | 0.00 | 0.00 | 0.00 |
| Tiantai County | 474711 | 0.0000 | 0.0000 | 0.0000 | 0.0000 | 0.0000 | 0.0000 | 331.46 | 0.00 | 0.00 | 0.00 | 0.00 | 0.00 | 0.00 | 0.00 | 0.00 | 0.00 | 0.00 | 0.00 | 0.00 | 0.00 | 0.00 | 0.00 | 0.00 | 0.00 |
| Xianju County | 431888 | 0.0000 | 0.0000 | 0.0000 | 0.0000 | 0.0000 | 0.0000 | 215.95 | 0.00 | 0.00 | 0.00 | 0.00 | 0.00 | 0.00 | 0.00 | 0.00 | 0.00 | 0.00 | 0.00 | 0.00 | 0.00 | 0.00 | 0.00 | 0.00 | 0.00 |
| Wenling City | 1416199 | 0.0000 | 0.0000 | 0.0000 | 0.0000 | 0.0000 | 0.0000 | 1457.05 | 0.00 | 0.00 | 0.00 | 0.00 | 0.00 | 0.00 | 0.00 | 0.00 | 0.00 | 0.00 | 0.00 | 0.00 | 0.00 | 0.00 | 0.00 | 0.00 | 0.00 |
| Linhai City | 1114146 | 0.2287 | 0.0000 | 0.0000 | 0.0000 | 0.0033 | 0.2320 | 508.91 | 116.37 | 0.00 | 0.00 | 0.00 | 1.70 | 118.07 | 18.98 | 14.72 | 6.86 | 6.33 | 0.50 | 0.38 | 0.18 | 1.43 | 0.14 | 0.22 | 0.01 |
| Yuhuan City | 644014 | 0.0000 | 0.0000 | 0.0000 | 0.0000 | 0.0000 | 0.0000 | 1427.18 | 0.00 | 0.00 | 0.00 | 0.00 | 0.00 | 0.00 | 0.00 | 0.00 | 0.00 | 0.00 | 0.00 | 0.00 | 0.00 | 0.00 | 0.00 | 0.00 | 0.00 |
| **Lishui City** | **2507396** | 0.1568 | 0.0000 | 0.0000 | 0.0000 | 0.0000 | 0.1568 | 145.15 | 22.76 | 0.00 | 0.00 | 0.00 | 0.00 | 22.76 | 3.41 | 2.65 | 1.23 | 1.14 | 0.09 | 0.07 | 0.03 | 0.26 | 0.03 | 0.04 | 0.00 |
| Liandu District | 562116 | 0.1007 | 0.0000 | 0.0000 | 0.0000 | 0.0000 | 0.1007 | 376.73 | 37.92 | 0.00 | 0.00 | 0.00 | 0.00 | 37.92 | 5.69 | 4.41 | 2.05 | 1.90 | 0.15 | 0.11 | 0.05 | 0.43 | 0.04 | 0.07 | 0.00 |
| Qingtian County | 509053 | 0.0000 | 0.0000 | 0.0000 | 0.0000 | 0.0000 | 0.0000 | 205.53 | 0.00 | 0.00 | 0.00 | 0.00 | 0.00 | 0.00 | 0.00 | 0.00 | 0.00 | 0.00 | 0.00 | 0.00 | 0.00 | 0.00 | 0.00 | 0.00 | 0.00 |
| Jinyun County | 405318 | 0.0000 | 0.0000 | 0.0000 | 0.0000 | 0.0000 | 0.0000 | 271.42 | 0.00 | 0.00 | 0.00 | 0.00 | 0.00 | 0.00 | 0.00 | 0.00 | 0.00 | 0.00 | 0.00 | 0.00 | 0.00 | 0.00 | 0.00 | 0.00 | 0.00 |
| Suichang County | 194385 | 0.0000 | 0.0000 | 0.0000 | 0.0000 | 0.0000 | 0.0000 | 76.55 | 0.00 | 0.00 | 0.00 | 0.00 | 0.00 | 0.00 | 0.00 | 0.00 | 0.00 | 0.00 | 0.00 | 0.00 | 0.00 | 0.00 | 0.00 | 0.00 | 0.00 |
| Songyang County | 204880 | 0.0000 | 0.0000 | 0.0000 | 0.0000 | 0.0000 | 0.0000 | 146.46 | 0.00 | 0.00 | 0.00 | 0.00 | 0.00 | 0.00 | 0.00 | 0.00 | 0.00 | 0.00 | 0.00 | 0.00 | 0.00 | 0.00 | 0.00 | 0.00 | 0.00 |
| Yunhe County | 129216 | 0.0000 | 0.0000 | 0.0000 | 0.0000 | 0.0000 | 0.0000 | 130.41 | 0.00 | 0.00 | 0.00 | 0.00 | 0.00 | 0.00 | 0.00 | 0.00 | 0.00 | 0.00 | 0.00 | 0.00 | 0.00 | 0.00 | 0.00 | 0.00 | 0.00 |
| Qingyuan County | 142551 | 0.0000 | 0.0000 | 0.0000 | 0.0000 | 0.0000 | 0.0000 | 75.08 | 0.00 | 0.00 | 0.00 | 0.00 | 0.00 | 0.00 | 0.00 | 0.00 | 0.00 | 0.00 | 0.00 | 0.00 | 0.00 | 0.00 | 0.00 | 0.00 | 0.00 |
| Jingning She Autonomous County | 111011 | 0.0561 | 0.0000 | 0.0000 | 0.0000 | 0.0000 | 0.0561 | 57.22 | 3.21 | 0.00 | 0.00 | 0.00 | 0.00 | 3.21 | 0.48 | 0.37 | 0.17 | 0.16 | 0.01 | 0.01 | 0.00 | 0.04 | 0.00 | 0.01 | 0.00 |
| Longquan City | 248866 | 0.0000 | 0.0000 | 0.0000 | 0.0000 | 0.0000 | 0.0000 | 81.73 | 0.00 | 0.00 | 0.00 | 0.00 | 0.00 | 0.00 | 0.00 | 0.00 | 0.00 | 0.00 | 0.00 | 0.00 | 0.00 | 0.00 | 0.00 | 0.00 | 0.00 |
| **Fujian Province** | 41540086 | 371.5315 | 162.3445 | 50.0704 | 5.3843 | 0.3307 | 589.6614 | 293.88 | 109186.74 | 47710.28 | 14714.83 | 1582.36 | 97.18 | 173291.39 | 40873.34 | 31693.19 | 14763.45 | 13623.09 | 1083.14 | 809.29 | 384.21 | 3073.68 | 306.55 | 478.22 | 12.26 |
| **Fuzhou City** | **8291268** | 53.6453 | 37.7217 | 3.4460 | 0.1160 | 0.0000 | 94.9291 | 269.10 | 14436.21 | 10151.10 | 927.33 | 31.22 | 0.00 | 25545.86 | 5792.14 | 4491.22 | 2092.12 | 1930.52 | 153.49 | 114.68 | 54.45 | 435.57 | 43.44 | 67.77 | 1.74 |
| Gulou District | 669090 | 0.0000 | 0.0000 | 0.0000 | 0.0000 | 0.0000 | 0.0000 | 19013.64 | 0.00 | 0.00 | 0.00 | 0.00 | 0.00 | 0.00 | 0.00 | 0.00 | 0.00 | 0.00 | 0.00 | 0.00 | 0.00 | 0.00 | 0.00 | 0.00 | 0.00 |
| Taijiang District | 411819 | 0.0007 | 0.0000 | 0.0000 | 0.0000 | 0.0000 | 0.0007 | 23708.64 | 15.81 | 0.00 | 0.00 | 0.00 | 0.00 | 15.81 | 2.37 | 1.84 | 0.86 | 0.79 | 0.06 | 0.05 | 0.02 | 0.18 | 0.02 | 0.03 | 0.00 |
| Cangshan District | 1142991 | 0.0467 | 0.0367 | 0.0000 | 0.0000 | 0.0000 | 0.0833 | 7803.58 | 364.17 | 286.13 | 0.00 | 0.00 | 0.00 | 650.30 | 140.46 | 108.92 | 50.74 | 46.82 | 3.72 | 2.78 | 1.32 | 10.56 | 1.05 | 1.64 | 0.04 |
| Mawei District | 290554 | 0.2667 | 0.1733 | 0.0000 | 0.0000 | 0.0000 | 0.4400 | 1270.01 | 338.67 | 220.14 | 0.00 | 0.00 | 0.00 | 558.81 | 116.84 | 90.60 | 42.20 | 38.94 | 3.10 | 2.31 | 1.10 | 8.79 | 0.88 | 1.37 | 0.04 |
| Jin'an District | 789775 | 0.0513 | 0.0287 | 0.0000 | 0.0000 | 0.0000 | 0.0800 | 1400.11 | 71.87 | 40.14 | 0.00 | 0.00 | 0.00 | 112.01 | 22.82 | 17.70 | 8.24 | 7.61 | 0.60 | 0.45 | 0.21 | 1.72 | 0.17 | 0.27 | 0.01 |
| Changle District | 790262 | 4.9740 | 1.6200 | 0.0767 | 0.0000 | 0.0000 | 6.6707 | 1157.37 | 5756.75 | 1874.94 | 88.73 | 0.00 | 0.00 | 7720.41 | 1479.23 | 1147.00 | 534.30 | 493.03 | 39.20 | 29.29 | 13.90 | 111.24 | 11.09 | 17.31 | 0.44 |
| Minhou County | 988200 | 5.8413 | 0.7524 | 0.0947 | 0.0000 | 0.0000 | 6.6884 | 46.57 | 272.05 | 35.04 | 4.41 | 0.00 | 0.00 | 311.50 | 53.97 | 41.85 | 19.49 | 17.99 | 1.43 | 1.07 | 0.51 | 4.06 | 0.40 | 0.63 | 0.02 |
| Lianjiang County | 639498 | 6.2667 | 0.4133 | 0.0000 | 0.0000 | 0.0000 | 6.6800 | 561.95 | 3521.55 | 232.27 | 0.00 | 0.00 | 0.00 | 3753.82 | 597.91 | 463.62 | 215.97 | 199.28 | 15.84 | 11.84 | 5.62 | 44.96 | 4.48 | 7.00 | 0.18 |
| Luoyuan County | 255214 | 3.9333 | 2.4200 | 0.5333 | 0.0000 | 0.0000 | 6.8867 | 238.54 | 938.26 | 577.27 | 127.22 | 0.00 | 0.00 | 1642.75 | 390.25 | 302.60 | 140.96 | 130.07 | 10.34 | 7.73 | 3.67 | 29.35 | 2.93 | 4.57 | 0.12 |
| Minqing County | 256181 | 1.5053 | 0.4400 | 0.0133 | 0.0000 | 0.0000 | 1.9587 | 171.65 | 258.38 | 75.52 | 2.29 | 0.00 | 0.00 | 336.20 | 62.79 | 48.69 | 22.68 | 20.93 | 1.66 | 1.24 | 0.59 | 4.72 | 0.47 | 0.73 | 0.02 |
| Yongtai County | 281216 | 1.3800 | 0.7933 | 0.0880 | 0.0000 | 0.0000 | 2.2613 | 126.06 | 173.96 | 100.01 | 11.09 | 0.00 | 0.00 | 285.07 | 62.75 | 48.66 | 22.67 | 20.92 | 1.66 | 1.24 | 0.59 | 4.72 | 0.47 | 0.73 | 0.02 |
| Pingtan County | 385981 | 15.0613 | 10.5320 | 2.5580 | 0.1160 | 0.0000 | 28.2673 | 1170.85 | 17634.50 | 12331.35 | 2995.02 | 135.82 | 0.00 | 33096.69 | 8250.25 | 6397.24 | 2979.99 | 2749.81 | 218.63 | 163.35 | 77.55 | 620.42 | 61.88 | 96.53 | 2.48 |
| Fuqing City | 1390487 | 14.3180 | 20.5120 | 0.0820 | 0.0000 | 0.0000 | 34.9120 | 839.16 | 12015.08 | 17212.84 | 68.81 | 0.00 | 0.00 | 29296.73 | 7007.40 | 5433.54 | 2531.07 | 2335.57 | 185.70 | 138.75 | 65.87 | 526.96 | 52.56 | 81.99 | 2.10 |
| **Xiamen City** | **5163970** | 38.1227 | 10.1533 | 1.9333 | 0.0000 | 0.0000 | 50.2093 | 3250.19 | 123906.01 | 33000.28 | 6283.70 | 0.00 | 0.00 | 163189.99 | 32256.21 | 25011.46 | 11650.94 | 10750.99 | 854.79 | 638.67 | 303.21 | 2425.67 | 241.92 | 377.40 | 9.68 |
| Siming District | 1073315 | 0.0000 | 0.0000 | 0.0000 | 0.0000 | 0.0000 | 0.0000 | 14197.29 | 0.00 | 0.00 | 0.00 | 0.00 | 0.00 | 0.00 | 0.00 | 0.00 | 0.00 | 0.00 | 0.00 | 0.00 | 0.00 | 0.00 | 0.00 | 0.00 | 0.00 |
| Haicang District | 582519 | 0.9000 | 0.0667 | 0.0333 | 0.0000 | 0.0000 | 1.0000 | 3332.30 | 2999.07 | 222.15 | 111.08 | 0.00 | 0.00 | 3332.30 | 583.15 | 452.18 | 210.63 | 194.36 | 15.45 | 11.55 | 5.48 | 43.85 | 4.37 | 6.82 | 0.17 |
| Huli District | 1036974 | 0.0107 | 0.0000 | 0.0000 | 0.0000 | 0.0000 | 0.0107 | 14965.71 | 159.73 | 0.00 | 0.00 | 0.00 | 0.00 | 159.73 | 23.96 | 18.58 | 8.65 | 7.99 | 0.63 | 0.47 | 0.23 | 1.80 | 0.18 | 0.28 | 0.01 |
| Jimei District | 1036987 | 8.3753 | 6.5400 | 0.1567 | 0.0000 | 0.0000 | 15.0720 | 4089.87 | 34254.04 | 26747.76 | 640.75 | 0.00 | 0.00 | 61642.55 | 13546.88 | 10504.25 | 4893.13 | 4515.18 | 358.99 | 268.23 | 127.34 | 1018.73 | 101.60 | 158.50 | 4.06 |
| Tong'an District | 855920 | 18.8000 | 3.5333 | 1.7333 | 0.0000 | 0.0000 | 24.0667 | 1316.01 | 24741.00 | 4649.90 | 2281.08 | 0.00 | 0.00 | 31671.98 | 6474.77 | 5020.54 | 2338.69 | 2158.04 | 171.58 | 128.20 | 60.86 | 486.90 | 48.56 | 75.75 | 1.94 |
| Xiang'an District | 578255 | 10.0367 | 0.0133 | 0.0100 | 0.0000 | 0.0000 | 10.0600 | 1583.48 | 15892.85 | 21.11 | 15.83 | 0.00 | 0.00 | 15929.80 | 2399.76 | 1860.78 | 866.79 | 799.84 | 63.59 | 47.52 | 22.56 | 180.46 | 18.00 | 28.08 | 0.72 |
| **Putian City** | **3210714** | 47.3907 | 30.7369 | 17.4671 | 0.3150 | 0.0000 | 95.9097 | 816.64 | 38701.16 | 25101.04 | 14264.32 | 257.24 | 0.00 | 78323.77 | 22099.87 | 17136.24 | 7982.47 | 7365.89 | 585.65 | 437.58 | 207.74 | 1661.91 | 165.75 | 258.57 | 6.63 |
| Chengxiang District | 547422 | 4.3800 | 0.8000 | 0.0000 | 0.0000 | 0.0000 | 5.1800 | 1137.24 | 4981.11 | 909.79 | 0.00 | 0.00 | 0.00 | 5890.90 | 1020.10 | 790.99 | 368.46 | 340.00 | 27.03 | 20.20 | 9.59 | 76.71 | 7.65 | 11.94 | 0.31 |
| Hanjiang District | 479605 | 2.2360 | 0.3353 | 0.0000 | 0.0000 | 0.0000 | 2.5713 | 625.66 | 1398.97 | 209.80 | 0.00 | 0.00 | 0.00 | 1608.78 | 272.79 | 211.52 | 98.53 | 90.92 | 7.23 | 5.40 | 2.56 | 20.51 | 2.05 | 3.19 | 0.08 |
| Licheng District | 673935 | 3.6020 | 2.9823 | 2.3931 | 0.3150 | 0.0000 | 9.2923 | 2420.22 | 8717.64 | 7217.75 | 5791.75 | 762.37 | 0.00 | 22489.51 | 7557.92 | 5860.41 | 2729.92 | 2519.05 | 200.28 | 149.65 | 71.04 | 568.36 | 56.68 | 88.43 | 2.27 |
| Xiuyu District | 604684 | 8.4333 | 5.2107 | 1.6167 | 0.0000 | 0.0000 | 15.2607 | 1071.89 | 9039.59 | 5585.25 | 1732.89 | 0.00 | 0.00 | 16357.72 | 4071.24 | 3156.84 | 1470.53 | 1356.95 | 107.89 | 80.61 | 38.27 | 306.16 | 30.53 | 47.63 | 1.22 |
| Xianyou County | 905068 | 28.7393 | 21.4087 | 13.4573 | 0.0000 | 0.0000 | 63.6053 | 491.59 | 14127.99 | 10524.31 | 6615.50 | 0.00 | 0.00 | 31267.80 | 9245.79 | 7169.19 | 3339.58 | 3081.62 | 245.01 | 183.07 | 86.91 | 695.28 | 69.34 | 108.18 | 2.77 |
| **Sanming City** | **2486450** | 14.7097 | 9.1080 | 0.0000 | 0.0000 | 0.0000 | 23.8177 | 108.27 | 1592.66 | 986.15 | 0.00 | 0.00 | 0.00 | 2578.81 | 534.74 | 414.64 | 193.15 | 178.23 | 14.17 | 10.59 | 5.03 | 40.21 | 4.01 | 6.26 | 0.16 |
| Sanyuan District | 408051 | 1.8833 | 0.1333 | 0.0000 | 0.0000 | 0.0000 | 2.0167 | 354.15 | 666.98 | 47.22 | 0.00 | 0.00 | 0.00 | 714.20 | 114.21 | 88.56 | 41.25 | 38.07 | 3.03 | 2.26 | 1.07 | 8.59 | 0.86 | 1.34 | 0.03 |
| Mingxi County | 98930 | 1.6667 | 0.0000 | 0.0000 | 0.0000 | 0.0000 | 1.6667 | 57.17 | 95.28 | 0.00 | 0.00 | 0.00 | 0.00 | 95.28 | 14.29 | 11.08 | 5.16 | 4.76 | 0.38 | 0.28 | 0.13 | 1.07 | 0.11 | 0.17 | 0.00 |
| Qingliu County | 118029 | 1.7400 | 0.6000 | 0.0000 | 0.0000 | 0.0000 | 2.3400 | 65.26 | 113.55 | 39.16 | 0.00 | 0.00 | 0.00 | 152.71 | 28.78 | 22.32 | 10.40 | 9.59 | 0.76 | 0.57 | 0.27 | 2.16 | 0.22 | 0.34 | 0.01 |
| Ninghua County | 261579 | 0.3733 | 1.8400 | 0.0000 | 0.0000 | 0.0000 | 2.2133 | 108.67 | 40.57 | 199.96 | 0.00 | 0.00 | 0.00 | 240.53 | 66.07 | 51.23 | 23.87 | 22.02 | 1.75 | 1.31 | 0.62 | 4.97 | 0.50 | 0.77 | 0.02 |
| Datian County | 299513 | 1.6667 | 1.7000 | 0.0000 | 0.0000 | 0.0000 | 3.3667 | 134.31 | 223.85 | 228.33 | 0.00 | 0.00 | 0.00 | 452.18 | 102.08 | 79.15 | 36.87 | 34.02 | 2.71 | 2.02 | 0.96 | 7.68 | 0.77 | 1.19 | 0.03 |
| Youxi County | 341638 | 1.2880 | 0.8520 | 0.0000 | 0.0000 | 0.0000 | 2.1400 | 99.90 | 128.67 | 85.12 | 0.00 | 0.00 | 0.00 | 213.79 | 44.84 | 34.77 | 16.19 | 14.94 | 1.19 | 0.89 | 0.42 | 3.37 | 0.34 | 0.52 | 0.01 |
| Sha County | 250503 | 3.3333 | 1.8667 | 0.0000 | 0.0000 | 0.0000 | 5.2000 | 139.31 | 464.36 | 260.04 | 0.00 | 0.00 | 0.00 | 724.40 | 147.67 | 114.50 | 53.34 | 49.22 | 3.91 | 2.92 | 1.39 | 11.10 | 1.11 | 1.73 | 0.04 |
| Jiangle County | 144943 | 0.0004 | 0.0000 | 0.0000 | 0.0000 | 0.0000 | 0.0004 | 64.76 | 0.03 | 0.00 | 0.00 | 0.00 | 0.00 | 0.03 | 0.00 | 0.00 | 0.00 | 0.00 | 0.00 | 0.00 | 0.00 | 0.00 | 0.00 | 0.00 | 0.00 |
| Taining County | 104071 | 0.4133 | 0.1920 | 0.0000 | 0.0000 | 0.0000 | 0.6053 | 68.03 | 28.12 | 13.06 | 0.00 | 0.00 | 0.00 | 41.18 | 8.14 | 6.31 | 2.94 | 2.71 | 0.22 | 0.16 | 0.08 | 0.61 | 0.06 | 0.10 | 0.00 |
| Jianing County | 114400 | 0.0000 | 0.0000 | 0.0000 | 0.0000 | 0.0000 | 0.0000 | 66.62 | 0.00 | 0.00 | 0.00 | 0.00 | 0.00 | 0.00 | 0.00 | 0.00 | 0.00 | 0.00 | 0.00 | 0.00 | 0.00 | 0.00 | 0.00 | 0.00 | 0.00 |
| Yong'an City | 344793 | 2.3447 | 1.9240 | 0.0000 | 0.0000 | 0.0000 | 4.2687 | 117.55 | 275.61 | 226.16 | 0.00 | 0.00 | 0.00 | 501.77 | 109.19 | 84.67 | 39.44 | 36.39 | 2.89 | 2.16 | 1.03 | 8.21 | 0.82 | 1.28 | 0.03 |
| **Quanzhou City** | **8782285** | 59.9316 | 34.1407 | 11.7920 | 1.9707 | 0.0000 | 107.8349 | 795.35 | 47666.50 | 27153.73 | 9378.75 | 1567.37 | 0.00 | 85766.34 | 22177.24 | 17196.23 | 8010.42 | 7391.67 | 587.70 | 439.11 | 208.47 | 1667.73 | 166.33 | 259.47 | 6.65 |
| Licheng District | 428361 | 0.0000 | 0.0000 | 1.7533 | 0.0000 | 0.0000 | 1.7533 | 8325.77 | 0.00 | 0.00 | 14597.85 | 0.00 | 0.00 | 14597.85 | 8758.71 | 6791.51 | 3163.65 | 2919.28 | 232.11 | 173.42 | 82.33 | 658.66 | 65.69 | 102.48 | 2.63 |
| Fortress District | 698557 | 1.4143 | 0.0000 | 0.0000 | 0.0000 | 0.0000 | 1.4143 | 6153.06 | 8702.07 | 0.00 | 0.00 | 0.00 | 0.00 | 8702.07 | 1305.31 | 1012.14 | 471.48 | 435.06 | 34.59 | 25.85 | 12.27 | 98.16 | 9.79 | 15.27 | 0.39 |
| Luojiang District | 247172 | 3.0433 | 1.7200 | 1.3360 | 0.0000 | 0.0000 | 6.0993 | 666.79 | 2029.26 | 1146.88 | 890.83 | 0.00 | 0.00 | 4066.97 | 1182.95 | 917.26 | 427.28 | 394.28 | 31.35 | 23.42 | 11.12 | 88.96 | 8.87 | 13.84 | 0.35 |
| Quangang District | 354296 | 1.1733 | 1.5067 | 0.0000 | 0.0000 | 0.0000 | 2.6800 | 1201.86 | 1410.18 | 1810.80 | 0.00 | 0.00 | 0.00 | 3220.98 | 754.77 | 585.25 | 272.62 | 251.56 | 20.00 | 14.94 | 7.09 | 56.76 | 5.66 | 8.83 | 0.23 |
| Hui'an County | 1030626 | 16.0667 | 5.8667 | 4.6000 | 0.3333 | 0.0000 | 26.8667 | 1422.51 | 22855.07 | 8345.42 | 6543.57 | 474.17 | 0.00 | 38218.22 | 10237.36 | 7938.05 | 3697.74 | 3412.11 | 271.29 | 202.70 | 96.23 | 769.85 | 76.78 | 119.78 | 3.07 |
| Anxi County | 1003599 | 0.0000 | 6.0000 | 0.0000 | 0.0000 | 0.0000 | 6.0000 | 335.16 | 0.00 | 2010.95 | 0.00 | 0.00 | 0.00 | 2010.95 | 603.29 | 467.79 | 217.91 | 201.08 | 15.99 | 11.95 | 5.67 | 45.37 | 4.52 | 7.06 | 0.18 |
| Yongchun County | 422531 | 0.1333 | 0.0000 | 0.0000 | 0.0000 | 0.0000 | 0.1333 | 289.86 | 38.65 | 0.00 | 0.00 | 0.00 | 0.00 | 38.65 | 5.80 | 4.50 | 2.09 | 1.93 | 0.15 | 0.11 | 0.05 | 0.44 | 0.04 | 0.07 | 0.00 |
| Dehua County | 332148 | 0.2667 | 0.0000 | 0.0000 | 0.0000 | 0.0000 | 0.2667 | 150.79 | 40.21 | 0.00 | 0.00 | 0.00 | 0.00 | 40.21 | 6.03 | 4.68 | 2.18 | 2.01 | 0.16 | 0.12 | 0.06 | 0.45 | 0.05 | 0.07 | 0.00 |
| Shishi City | 685930 | 2.4187 | 0.5307 | 0.0000 | 0.0000 | 0.0000 | 2.9493 | 4251.19 | 10282.22 | 2255.97 | 0.00 | 0.00 | 0.00 | 12538.19 | 2219.12 | 1720.71 | 801.55 | 739.63 | 58.81 | 43.94 | 20.86 | 166.88 | 16.64 | 25.96 | 0.67 |
| Jinjiang City | 2061551 | 18.7487 | 10.8500 | 2.5027 | 1.3373 | 0.0000 | 33.4387 | 3098.49 | 58092.60 | 33618.64 | 7754.49 | 4143.72 | 0.00 | 103609.46 | 26767.15 | 20755.25 | 9668.30 | 8921.49 | 709.33 | 529.99 | 251.61 | 2012.89 | 200.75 | 313.18 | 8.03 |
| Nan'an City | 1517514 | 16.6667 | 7.6667 | 1.6000 | 0.3000 | 0.0000 | 26.2333 | 756.64 | 12610.64 | 5800.89 | 1210.62 | 226.99 | 0.00 | 19849.15 | 4539.83 | 3520.18 | 1639.79 | 1513.13 | 120.31 | 89.89 | 42.67 | 341.40 | 34.05 | 53.12 | 1.36 |
| **Zhangzhou City** | **5054328** | 128.8402 | 33.8853 | 12.6433 | 1.1867 | 0.3307 | 176.8862 | 400.04 | 51541.00 | 13555.43 | 5057.82 | 474.71 | 132.28 | 70761.24 | 15331.29 | 11887.88 | 5537.66 | 5109.92 | 406.28 | 303.56 | 144.11 | 1152.91 | 114.98 | 179.38 | 4.60 |
| Xiangcheng District | 638060 | 7.0353 | 4.9267 | 1.3533 | 0.1333 | 0.0333 | 13.4820 | 2543.79 | 17896.44 | 12532.43 | 3442.60 | 339.17 | 84.79 | 34295.44 | 8857.41 | 6868.03 | 3199.30 | 2952.17 | 234.72 | 175.38 | 83.26 | 666.08 | 66.43 | 103.63 | 2.66 |
| Longwen District | 301883 | 3.7602 | 0.2380 | 0.0000 | 0.0000 | 0.0000 | 3.9982 | 2378.34 | 8942.95 | 566.05 | 0.00 | 0.00 | 0.00 | 9508.99 | 1511.26 | 1171.83 | 545.87 | 503.70 | 40.05 | 29.92 | 14.21 | 113.65 | 11.33 | 17.68 | 0.45 |
| Yunxiao County | 411558 | 6.6200 | 2.8133 | 2.5000 | 0.5333 | 0.0333 | 12.5000 | 401.36 | 2657.03 | 1129.17 | 1003.41 | 214.06 | 13.38 | 5017.04 | 1522.64 | 1180.65 | 549.98 | 507.50 | 40.35 | 30.15 | 14.31 | 114.50 | 11.42 | 17.81 | 0.46 |
| Zhangpu County | 847535 | 23.9360 | 12.6360 | 7.0253 | 0.4533 | 0.2640 | 44.3147 | 418.47 | 10016.59 | 5287.84 | 2939.92 | 189.71 | 110.48 | 18544.53 | 5103.99 | 3957.63 | 1843.56 | 1701.16 | 135.26 | 101.06 | 47.98 | 383.82 | 38.28 | 59.72 | 1.53 |
| Zhaoan County | 560969 | 1.8300 | 1.5720 | 0.4360 | 0.0000 | 0.0000 | 3.8380 | 442.44 | 809.66 | 695.51 | 192.90 | 0.00 | 0.00 | 1698.08 | 445.85 | 345.71 | 161.04 | 148.60 | 11.81 | 8.83 | 4.19 | 33.53 | 3.34 | 5.22 | 0.13 |
| Changtai County | 228235 | 10.9520 | 6.8333 | 0.5020 | 0.0000 | 0.0000 | 18.2873 | 254.09 | 2782.81 | 1736.29 | 127.55 | 0.00 | 0.00 | 4646.65 | 1014.84 | 786.91 | 366.56 | 338.25 | 26.89 | 20.09 | 9.54 | 76.32 | 7.61 | 11.87 | 0.30 |
| Dongshan County | 219511 | 16.6667 | 0.0000 | 0.0000 | 0.0000 | 0.0000 | 16.6667 | 985.64 | 16427.27 | 0.00 | 0.00 | 0.00 | 0.00 | 16427.27 | 2464.09 | 1910.66 | 890.03 | 821.28 | 65.30 | 48.79 | 23.16 | 185.30 | 18.48 | 28.83 | 0.74 |
| Nanjing County | 305259 | 21.3333 | 0.2000 | 0.0333 | 0.0000 | 0.0000 | 21.5667 | 155.55 | 3318.48 | 31.11 | 5.19 | 0.00 | 0.00 | 3354.78 | 510.22 | 395.62 | 184.29 | 170.06 | 13.52 | 10.10 | 4.80 | 38.37 | 3.83 | 5.97 | 0.15 |
| Heping County | 455042 | 2.4333 | 1.0993 | 0.0000 | 0.0000 | 0.0000 | 3.5327 | 196.88 | 479.07 | 216.43 | 0.00 | 0.00 | 0.00 | 695.50 | 136.79 | 106.07 | 49.41 | 45.59 | 3.62 | 2.71 | 1.29 | 10.29 | 1.03 | 1.60 | 0.04 |
| Hua'an County | 134276 | 5.6000 | 0.9333 | 0.4000 | 0.0000 | 0.0000 | 6.9333 | 105.09 | 588.51 | 98.09 | 42.04 | 0.00 | 0.00 | 728.64 | 142.93 | 110.82 | 51.62 | 47.64 | 3.79 | 2.83 | 1.34 | 10.75 | 1.07 | 1.67 | 0.04 |
| Longhai City | 952000 | 28.6733 | 2.6333 | 0.3933 | 0.0667 | 0.0000 | 31.7667 | 752.03 | 21563.33 | 1980.36 | 295.80 | 50.14 | 0.00 | 23889.62 | 4046.19 | 3137.42 | 1461.49 | 1348.60 | 107.22 | 80.11 | 38.03 | 304.27 | 30.35 | 47.34 | 1.21 |
| **Nanping City** | **2680645** | 5.9067 | 0.9852 | 0.0000 | 0.0000 | 0.0000 | 6.8919 | 101.96 | 602.26 | 100.45 | 0.00 | 0.00 | 0.00 | 702.71 | 120.47 | 93.42 | 43.52 | 40.15 | 3.19 | 2.39 | 1.13 | 9.06 | 0.90 | 1.41 | 0.04 |
| Yanping District | 454605 | 1.3660 | 0.3740 | 0.0000 | 0.0000 | 0.0000 | 1.7400 | 171.13 | 233.76 | 64.00 | 0.00 | 0.00 | 0.00 | 297.76 | 54.27 | 42.08 | 19.60 | 18.09 | 1.44 | 1.07 | 0.51 | 4.08 | 0.41 | 0.63 | 0.02 |
| Jianyang District | 340843 | 4.0433 | 0.2740 | 0.0000 | 0.0000 | 0.0000 | 4.3173 | 100.62 | 406.84 | 27.57 | 0.00 | 0.00 | 0.00 | 434.41 | 69.30 | 53.73 | 25.03 | 23.10 | 1.84 | 1.37 | 0.65 | 5.21 | 0.52 | 0.81 | 0.02 |
| Shunchang County | 179064 | 0.2333 | 0.0667 | 0.0000 | 0.0000 | 0.0000 | 0.3000 | 90.35 | 21.08 | 6.02 | 0.00 | 0.00 | 0.00 | 27.10 | 4.97 | 3.85 | 1.79 | 1.66 | 0.13 | 0.10 | 0.05 | 0.37 | 0.04 | 0.06 | 0.00 |
| Pucheng County | 297719 | 0.0000 | 0.0000 | 0.0000 | 0.0000 | 0.0000 | 0.0000 | 88.17 | 0.00 | 0.00 | 0.00 | 0.00 | 0.00 | 0.00 | 0.00 | 0.00 | 0.00 | 0.00 | 0.00 | 0.00 | 0.00 | 0.00 | 0.00 | 0.00 | 0.00 |
| Guangze County | 130294 | 0.0000 | 0.0000 | 0.0000 | 0.0000 | 0.0000 | 0.0000 | 58.18 | 0.00 | 0.00 | 0.00 | 0.00 | 0.00 | 0.00 | 0.00 | 0.00 | 0.00 | 0.00 | 0.00 | 0.00 | 0.00 | 0.00 | 0.00 | 0.00 | 0.00 |
| Songxi County | 130867 | 0.2007 | 0.0533 | 0.0000 | 0.0000 | 0.0000 | 0.2540 | 125.63 | 25.21 | 6.70 | 0.00 | 0.00 | 0.00 | 31.91 | 5.79 | 4.49 | 2.09 | 1.93 | 0.15 | 0.11 | 0.05 | 0.44 | 0.04 | 0.07 | 0.00 |
| Zhenghe County | 179413 | 0.0000 | 0.0000 | 0.0000 | 0.0000 | 0.0000 | 0.0000 | 102.77 | 0.00 | 0.00 | 0.00 | 0.00 | 0.00 | 0.00 | 0.00 | 0.00 | 0.00 | 0.00 | 0.00 | 0.00 | 0.00 | 0.00 | 0.00 | 0.00 | 0.00 |
| Shaowu City | 273721 | 0.0000 | 0.1505 | 0.0000 | 0.0000 | 0.0000 | 0.1505 | 95.64 | 0.00 | 14.40 | 0.00 | 0.00 | 0.00 | 14.40 | 4.32 | 3.35 | 1.56 | 1.44 | 0.11 | 0.09 | 0.04 | 0.32 | 0.03 | 0.05 | 0.00 |
| Wuyishan City | 259668 | 0.0400 | 0.0600 | 0.0000 | 0.0000 | 0.0000 | 0.1000 | 92.62 | 3.70 | 5.56 | 0.00 | 0.00 | 0.00 | 9.26 | 2.22 | 1.72 | 0.80 | 0.74 | 0.06 | 0.04 | 0.02 | 0.17 | 0.02 | 0.03 | 0.00 |
| Jian'ou City | 434451 | 0.0233 | 0.0067 | 0.0000 | 0.0000 | 0.0000 | 0.0300 | 103.55 | 2.42 | 0.69 | 0.00 | 0.00 | 0.00 | 3.11 | 0.57 | 0.44 | 0.21 | 0.19 | 0.02 | 0.01 | 0.01 | 0.04 | 0.00 | 0.01 | 0.00 |
| **Longyan City** | **2723637** | 15.4860 | 4.4133 | 2.5733 | 1.7853 | 0.0000 | 24.2580 | 143.15 | 2216.80 | 631.76 | 368.37 | 255.57 | 0.00 | 3472.51 | 947.53 | 734.71 | 342.25 | 315.81 | 25.11 | 18.76 | 8.91 | 71.25 | 7.11 | 11.09 | 0.28 |
| Silla District | 841745 | 7.5193 | 0.9267 | 0.2333 | 0.0000 | 0.0000 | 8.6793 | 314.93 | 2368.06 | 291.84 | 73.48 | 0.00 | 0.00 | 2733.38 | 486.85 | 377.50 | 175.85 | 162.27 | 12.90 | 9.64 | 4.58 | 36.61 | 3.65 | 5.70 | 0.15 |
| Yongding District | 325880 | 1.7333 | 1.6133 | 0.4667 | 0.0000 | 0.0000 | 3.8133 | 146.43 | 253.81 | 236.24 | 68.33 | 0.00 | 0.00 | 558.39 | 149.94 | 116.27 | 54.16 | 49.98 | 3.97 | 2.97 | 1.41 | 11.28 | 1.12 | 1.75 | 0.04 |
| Changting County | 397470 | 0.2000 | 0.0000 | 0.0000 | 0.0000 | 0.0000 | 0.2000 | 128.08 | 25.62 | 0.00 | 0.00 | 0.00 | 0.00 | 25.62 | 3.84 | 2.98 | 1.39 | 1.28 | 0.10 | 0.08 | 0.04 | 0.29 | 0.03 | 0.04 | 0.00 |
| Shanghang County | 376392 | 1.0000 | 0.0267 | 0.0000 | 0.0000 | 0.0000 | 1.0267 | 131.94 | 131.94 | 3.52 | 0.00 | 0.00 | 0.00 | 135.46 | 20.85 | 16.16 | 7.53 | 6.95 | 0.55 | 0.41 | 0.20 | 1.57 | 0.16 | 0.24 | 0.01 |
| Wuping County | 278238 | 1.6533 | 1.4133 | 1.8267 | 1.7853 | 0.0000 | 6.6787 | 105.53 | 174.48 | 149.15 | 192.77 | 188.41 | 0.00 | 704.82 | 337.31 | 261.55 | 121.84 | 112.43 | 8.94 | 6.68 | 3.17 | 25.37 | 2.53 | 3.95 | 0.10 |
| Liancheng County | 250518 | 1.0000 | 0.2000 | 0.0133 | 0.0000 | 0.0000 | 1.2133 | 97.11 | 97.11 | 19.42 | 1.29 | 0.00 | 0.00 | 117.82 | 21.17 | 16.41 | 7.65 | 7.06 | 0.56 | 0.42 | 0.20 | 1.59 | 0.16 | 0.25 | 0.01 |
| Zhangping City | 253394 | 2.3800 | 0.2333 | 0.0333 | 0.0000 | 0.0000 | 2.6467 | 85.72 | 204.02 | 20.00 | 2.86 | 0.00 | 0.00 | 226.88 | 38.32 | 29.71 | 13.84 | 12.77 | 1.02 | 0.76 | 0.36 | 2.88 | 0.29 | 0.45 | 0.01 |
| **Ningde City** | **3146789** | 7.4987 | 1.2000 | 0.2153 | 0.0107 | 0.0000 | 8.9247 | 240.96 | 1806.85 | 289.15 | 51.89 | 2.57 | 0.00 | 2150.46 | 390.96 | 303.15 | 141.21 | 130.31 | 10.36 | 7.74 | 3.68 | 29.40 | 2.93 | 4.57 | 0.12 |
| Jiaocheng District | 623840 | 2.7333 | 0.2000 | 0.0000 | 0.0000 | 0.0000 | 2.9333 | 439.91 | 1202.43 | 87.98 | 0.00 | 0.00 | 0.00 | 1290.41 | 206.76 | 160.32 | 74.68 | 68.91 | 5.48 | 4.09 | 1.94 | 15.55 | 1.55 | 2.42 | 0.06 |
| Xiapu County | 475936 | 3.2667 | 0.0233 | 0.0000 | 0.0000 | 0.0000 | 3.2900 | 313.18 | 1023.05 | 7.31 | 0.00 | 0.00 | 0.00 | 1030.35 | 155.65 | 120.69 | 56.22 | 51.88 | 4.12 | 3.08 | 1.46 | 11.70 | 1.17 | 1.82 | 0.05 |
| Gutian County | 323771 | 0.0700 | 0.0433 | 0.0153 | 0.0107 | 0.0000 | 0.1393 | 136.51 | 9.56 | 5.92 | 2.09 | 1.46 | 0.00 | 19.02 | 5.63 | 4.36 | 2.03 | 1.88 | 0.15 | 0.11 | 0.05 | 0.42 | 0.04 | 0.07 | 0.00 |
| Pingnan County | 139815 | 0.0733 | 0.0000 | 0.0000 | 0.0000 | 0.0000 | 0.0733 | 94.23 | 6.91 | 0.00 | 0.00 | 0.00 | 0.00 | 6.91 | 1.04 | 0.80 | 0.37 | 0.35 | 0.03 | 0.02 | 0.01 | 0.08 | 0.01 | 0.01 | 0.00 |
| Shouning County | 177960 | 0.0000 | 0.0000 | 0.0000 | 0.0000 | 0.0000 | 0.0000 | 124.49 | 0.00 | 0.00 | 0.00 | 0.00 | 0.00 | 0.00 | 0.00 | 0.00 | 0.00 | 0.00 | 0.00 | 0.00 | 0.00 | 0.00 | 0.00 | 0.00 | 0.00 |
| Zhouning County | 149567 | 0.0887 | 0.0667 | 0.0000 | 0.0000 | 0.0000 | 0.1553 | 144.05 | 12.77 | 9.60 | 0.00 | 0.00 | 0.00 | 22.38 | 4.80 | 3.72 | 1.73 | 1.60 | 0.13 | 0.09 | 0.05 | 0.36 | 0.04 | 0.06 | 0.00 |
| Zherong County | 92989 | 0.0000 | 0.0000 | 0.0000 | 0.0000 | 0.0000 | 0.0000 | 172.59 | 0.00 | 0.00 | 0.00 | 0.00 | 0.00 | 0.00 | 0.00 | 0.00 | 0.00 | 0.00 | 0.00 | 0.00 | 0.00 | 0.00 | 0.00 | 0.00 | 0.00 |
| Fu'an City | 609779 | 1.2667 | 0.8667 | 0.2000 | 0.0000 | 0.0000 | 2.3333 | 343.89 | 435.59 | 298.03 | 68.78 | 0.00 | 0.00 | 802.40 | 196.02 | 151.99 | 70.80 | 65.33 | 5.19 | 3.88 | 1.84 | 14.74 | 1.47 | 2.29 | 0.06 |
| Fuding City | 553132 | 0.0000 | 0.0000 | 0.0000 | 0.0000 | 0.0000 | 0.0000 | 372.10 | 0.00 | 0.00 | 0.00 | 0.00 | 0.00 | 0.00 | 0.00 | 0.00 | 0.00 | 0.00 | 0.00 | 0.00 | 0.00 | 0.00 | 0.00 | 0.00 | 0.00 |
| **Jiangxi Province** | 45188635 | 279.0019 | 88.0680 | 49.5840 | 1.0540 | 0.2573 | 417.9653 | 270.67 | 75517.76 | 23837.46 | 13420.96 | 285.29 | 69.65 | 113131.12 | 26822.39 | 20798.08 | 9688.25 | 8939.90 | 710.79 | 531.08 | 252.13 | 2017.04 | 201.17 | 313.82 | 8.05 |
| **Nanchang city** | 6255007 | 0.0133 | 0.0060 | 0.0000 | 0.0000 | 0.0000 | 0.0193 | 870.08 | 11.60 | 5.22 | 0.00 | 0.00 | 0.00 | 16.82 | 3.31 | 2.56 | 1.19 | 1.10 | 0.09 | 0.07 | 0.03 | 0.25 | 0.02 | 0.04 | 0.00 |
| East hu District | 421690 | 0.0000 | 0.0000 | 0.0000 | 0.0000 | 0.0000 | 0.0000 | 7559.88 | 0.00 | 0.00 | 0.00 | 0.00 | 0.00 | 0.00 | 0.00 | 0.00 | 0.00 | 0.00 | 0.00 | 0.00 | 0.00 | 0.00 | 0.00 | 0.00 | 0.00 |
| West hu District | 485161 | 0.0000 | 0.0000 | 0.0000 | 0.0000 | 0.0000 | 0.0000 | 13921.41 | 0.00 | 0.00 | 0.00 | 0.00 | 0.00 | 0.00 | 0.00 | 0.00 | 0.00 | 0.00 | 0.00 | 0.00 | 0.00 | 0.00 | 0.00 | 0.00 | 0.00 |
| Qingyunpu District | 349074 | 0.0000 | 0.0000 | 0.0000 | 0.0000 | 0.0000 | 0.0000 | 9393.81 | 0.00 | 0.00 | 0.00 | 0.00 | 0.00 | 0.00 | 0.00 | 0.00 | 0.00 | 0.00 | 0.00 | 0.00 | 0.00 | 0.00 | 0.00 | 0.00 | 0.00 |
| Qingshan hu District | 1307366 | 0.0000 | 0.0000 | 0.0000 | 0.0000 | 0.0000 | 0.0000 | 5855.28 | 0.00 | 0.00 | 0.00 | 0.00 | 0.00 | 0.00 | 0.00 | 0.00 | 0.00 | 0.00 | 0.00 | 0.00 | 0.00 | 0.00 | 0.00 | 0.00 | 0.00 |
| Xinjian District | 810614 | 0.0000 | 0.0000 | 0.0000 | 0.0000 | 0.0000 | 0.0000 | 384.01 | 0.00 | 0.00 | 0.00 | 0.00 | 0.00 | 0.00 | 0.00 | 0.00 | 0.00 | 0.00 | 0.00 | 0.00 | 0.00 | 0.00 | 0.00 | 0.00 | 0.00 |
| Honggutan District | 555755 | 0.0000 | 0.0000 | 0.0000 | 0.0000 | 0.0000 | 0.0000 | 1758.94 | 0.00 | 0.00 | 0.00 | 0.00 | 0.00 | 0.00 | 0.00 | 0.00 | 0.00 | 0.00 | 0.00 | 0.00 | 0.00 | 0.00 | 0.00 | 0.00 | 0.00 |
| Nanchang County | 1452502 | 0.0133 | 0.0060 | 0.0000 | 0.0000 | 0.0000 | 0.0193 | 800.36 | 10.67 | 4.80 | 0.00 | 0.00 | 0.00 | 15.47 | 3.04 | 2.36 | 1.10 | 1.01 | 0.08 | 0.06 | 0.03 | 0.23 | 0.02 | 0.04 | 0.00 |
| Anyi County | 252591 | 0.0000 | 0.0000 | 0.0000 | 0.0000 | 0.0000 | 0.0000 | 382.50 | 0.00 | 0.00 | 0.00 | 0.00 | 0.00 | 0.00 | 0.00 | 0.00 | 0.00 | 0.00 | 0.00 | 0.00 | 0.00 | 0.00 | 0.00 | 0.00 | 0.00 |
| Jinxian County | 620254 | 0.0000 | 0.0000 | 0.0000 | 0.0000 | 0.0000 | 0.0000 | 320.40 | 0.00 | 0.00 | 0.00 | 0.00 | 0.00 | 0.00 | 0.00 | 0.00 | 0.00 | 0.00 | 0.00 | 0.00 | 0.00 | 0.00 | 0.00 | 0.00 | 0.00 |
| **Jingdezhen City** | 1618979 | 0.9967 | 0.0000 | 0.0000 | 0.0000 | 0.0000 | 0.9967 | 307.76 | 306.73 | 0.00 | 0.00 | 0.00 | 0.00 | 306.73 | 46.01 | 35.68 | 16.62 | 15.34 | 1.22 | 0.91 | 0.43 | 3.46 | 0.35 | 0.54 | 0.01 |
| Changjiang District | 201363 | 0.0000 | 0.0000 | 0.0000 | 0.0000 | 0.0000 | 0.0000 | 639.55 | 0.00 | 0.00 | 0.00 | 0.00 | 0.00 | 0.00 | 0.00 | 0.00 | 0.00 | 0.00 | 0.00 | 0.00 | 0.00 | 0.00 | 0.00 | 0.00 | 0.00 |
| Zhushan District | 383453 | 0.0000 | 0.0000 | 0.0000 | 0.0000 | 0.0000 | 0.0000 | 3443.36 | 0.00 | 0.00 | 0.00 | 0.00 | 0.00 | 0.00 | 0.00 | 0.00 | 0.00 | 0.00 | 0.00 | 0.00 | 0.00 | 0.00 | 0.00 | 0.00 | 0.00 |
| Fuliang County | 280376 | 0.0000 | 0.0000 | 0.0000 | 0.0000 | 0.0000 | 0.0000 | 98.34 | 0.00 | 0.00 | 0.00 | 0.00 | 0.00 | 0.00 | 0.00 | 0.00 | 0.00 | 0.00 | 0.00 | 0.00 | 0.00 | 0.00 | 0.00 | 0.00 | 0.00 |
| Leping City | 753787 | 0.9967 | 0.0000 | 0.0000 | 0.0000 | 0.0000 | 0.9967 | 380.09 | 378.82 | 0.00 | 0.00 | 0.00 | 0.00 | 378.82 | 56.82 | 44.06 | 20.52 | 18.94 | 1.51 | 1.13 | 0.53 | 4.27 | 0.43 | 0.66 | 0.02 |
| **Pingxiang City** | 1804805 | 0.0051 | 0.0000 | 0.0000 | 0.0000 | 0.0000 | 0.0051 | 470.90 | 2.39 | 0.00 | 0.00 | 0.00 | 0.00 | 2.39 | 0.36 | 0.28 | 0.13 | 0.12 | 0.01 | 0.01 | 0.00 | 0.03 | 0.00 | 0.00 | 0.00 |
| Anyuan District | 553293 | 0.0000 | 0.0000 | 0.0000 | 0.0000 | 0.0000 | 0.0000 | 2613.45 | 0.00 | 0.00 | 0.00 | 0.00 | 0.00 | 0.00 | 0.00 | 0.00 | 0.00 | 0.00 | 0.00 | 0.00 | 0.00 | 0.00 | 0.00 | 0.00 | 0.00 |
| Xiangdong District | 304423 | 0.0000 | 0.0000 | 0.0000 | 0.0000 | 0.0000 | 0.0000 | 355.29 | 0.00 | 0.00 | 0.00 | 0.00 | 0.00 | 0.00 | 0.00 | 0.00 | 0.00 | 0.00 | 0.00 | 0.00 | 0.00 | 0.00 | 0.00 | 0.00 | 0.00 |
| Lianhua County | 216938 | 0.0000 | 0.0000 | 0.0000 | 0.0000 | 0.0000 | 0.0000 | 202.20 | 0.00 | 0.00 | 0.00 | 0.00 | 0.00 | 0.00 | 0.00 | 0.00 | 0.00 | 0.00 | 0.00 | 0.00 | 0.00 | 0.00 | 0.00 | 0.00 | 0.00 |
| Shangli County | 469957 | 0.0000 | 0.0000 | 0.0000 | 0.0000 | 0.0000 | 0.0000 | 644.99 | 0.00 | 0.00 | 0.00 | 0.00 | 0.00 | 0.00 | 0.00 | 0.00 | 0.00 | 0.00 | 0.00 | 0.00 | 0.00 | 0.00 | 0.00 | 0.00 | 0.00 |
| Luxi County | 260194 | 0.0051 | 0.0000 | 0.0000 | 0.0000 | 0.0000 | 0.0051 | 270.30 | 1.37 | 0.00 | 0.00 | 0.00 | 0.00 | 1.37 | 0.21 | 0.16 | 0.07 | 0.07 | 0.01 | 0.00 | 0.00 | 0.02 | 0.00 | 0.00 | 0.00 |
| **Jiujiang City** | 4600276 | 1.0733 | 0.2700 | 0.0000 | 0.0000 | 0.0000 | 1.3433 | 241.03 | 258.70 | 65.08 | 0.00 | 0.00 | 0.00 | 323.78 | 58.33 | 45.23 | 21.07 | 19.44 | 1.55 | 1.15 | 0.55 | 4.39 | 0.44 | 0.68 | 0.02 |
| Lianxi District | 418339 | 0.0000 | 0.0033 | 0.0000 | 0.0000 | 0.0000 | 0.0033 | 830.78 | 0.00 | 2.77 | 0.00 | 0.00 | 0.00 | 2.77 | 0.83 | 0.64 | 0.30 | 0.28 | 0.02 | 0.02 | 0.01 | 0.06 | 0.01 | 0.01 | 0.00 |
| Xunyang District | 433122 | 0.6733 | 0.0000 | 0.0000 | 0.0000 | 0.0000 | 0.6733 | 8919.32 | 6005.67 | 0.00 | 0.00 | 0.00 | 0.00 | 6005.67 | 900.85 | 698.52 | 325.39 | 300.25 | 23.87 | 17.84 | 8.47 | 67.74 | 6.76 | 10.54 | 0.27 |
| Chaisang District (formerly known as Jiujiang County) | 312807 | 0.4000 | 0.2667 | 0.0000 | 0.0000 | 0.0000 | 0.6667 | 341.31 | 136.52 | 91.02 | 0.00 | 0.00 | 0.00 | 227.54 | 47.78 | 37.05 | 17.26 | 15.93 | 1.27 | 0.95 | 0.45 | 3.59 | 0.36 | 0.56 | 0.01 |
| Wuning County | 322161 | 0.0000 | 0.0000 | 0.0000 | 0.0000 | 0.0000 | 0.0000 | 91.95 | 0.00 | 0.00 | 0.00 | 0.00 | 0.00 | 0.00 | 0.00 | 0.00 | 0.00 | 0.00 | 0.00 | 0.00 | 0.00 | 0.00 | 0.00 | 0.00 | 0.00 |
| Xiushui County | 710620 | 0.0000 | 0.0000 | 0.0000 | 0.0000 | 0.0000 | 0.0000 | 157.83 | 0.00 | 0.00 | 0.00 | 0.00 | 0.00 | 0.00 | 0.00 | 0.00 | 0.00 | 0.00 | 0.00 | 0.00 | 0.00 | 0.00 | 0.00 | 0.00 | 0.00 |
| Yongxiu County | 316365 | 0.0000 | 0.0000 | 0.0000 | 0.0000 | 0.0000 | 0.0000 | 162.70 | 0.00 | 0.00 | 0.00 | 0.00 | 0.00 | 0.00 | 0.00 | 0.00 | 0.00 | 0.00 | 0.00 | 0.00 | 0.00 | 0.00 | 0.00 | 0.00 | 0.00 |
| De'an County | 167090 | 0.0000 | 0.0000 | 0.0000 | 0.0000 | 0.0000 | 0.0000 | 194.61 | 0.00 | 0.00 | 0.00 | 0.00 | 0.00 | 0.00 | 0.00 | 0.00 | 0.00 | 0.00 | 0.00 | 0.00 | 0.00 | 0.00 | 0.00 | 0.00 | 0.00 |
| Duchang County | 577684 | 0.0000 | 0.0000 | 0.0000 | 0.0000 | 0.0000 | 0.0000 | 259.25 | 0.00 | 0.00 | 0.00 | 0.00 | 0.00 | 0.00 | 0.00 | 0.00 | 0.00 | 0.00 | 0.00 | 0.00 | 0.00 | 0.00 | 0.00 | 0.00 | 0.00 |
| Hukou County | 227462 | 0.0000 | 0.0000 | 0.0000 | 0.0000 | 0.0000 | 0.0000 | 338.81 | 0.00 | 0.00 | 0.00 | 0.00 | 0.00 | 0.00 | 0.00 | 0.00 | 0.00 | 0.00 | 0.00 | 0.00 | 0.00 | 0.00 | 0.00 | 0.00 | 0.00 |
| Pengze County | 284757 | 0.0000 | 0.0000 | 0.0000 | 0.0000 | 0.0000 | 0.0000 | 184.80 | 0.00 | 0.00 | 0.00 | 0.00 | 0.00 | 0.00 | 0.00 | 0.00 | 0.00 | 0.00 | 0.00 | 0.00 | 0.00 | 0.00 | 0.00 | 0.00 | 0.00 |
| Ruichang City | 403655 | 0.0000 | 0.0000 | 0.0000 | 0.0000 | 0.0000 | 0.0000 | 284.04 | 0.00 | 0.00 | 0.00 | 0.00 | 0.00 | 0.00 | 0.00 | 0.00 | 0.00 | 0.00 | 0.00 | 0.00 | 0.00 | 0.00 | 0.00 | 0.00 | 0.00 |
| Gongqingcheng City | 194689 | 0.0000 | 0.0000 | 0.0000 | 0.0000 | 0.0000 | 0.0000 | 688.90 | 0.00 | 0.00 | 0.00 | 0.00 | 0.00 | 0.00 | 0.00 | 0.00 | 0.00 | 0.00 | 0.00 | 0.00 | 0.00 | 0.00 | 0.00 | 0.00 | 0.00 |
| Lushan City | 231525 | 0.0000 | 0.0000 | 0.0000 | 0.0000 | 0.0000 | 0.0000 | 348.55 | 0.00 | 0.00 | 0.00 | 0.00 | 0.00 | 0.00 | 0.00 | 0.00 | 0.00 | 0.00 | 0.00 | 0.00 | 0.00 | 0.00 | 0.00 | 0.00 | 0.00 |
| **Xinyu City** | 1202499 | 2.1067 | 18.5267 | 0.0400 | 0.0000 | 0.0000 | 20.6733 | 380.63 | 801.87 | 7051.88 | 15.23 | 0.00 | 0.00 | 7868.97 | 2244.98 | 1740.76 | 810.89 | 748.25 | 59.49 | 44.45 | 21.10 | 168.82 | 16.84 | 26.27 | 0.67 |
| Yushui District | 927950 | 0.0000 | 18.2467 | 0.0000 | 0.0000 | 0.0000 | 18.2467 | 521.14 | 0.00 | 9509.15 | 0.00 | 0.00 | 0.00 | 9509.15 | 2852.75 | 2212.02 | 1030.41 | 950.82 | 75.60 | 56.48 | 26.82 | 214.53 | 21.40 | 33.38 | 0.86 |
| Fenyi County | 274549 | 2.1067 | 0.2800 | 0.0400 | 0.0000 | 0.0000 | 2.4267 | 199.15 | 419.54 | 55.76 | 7.97 | 0.00 | 0.00 | 483.27 | 84.44 | 65.47 | 30.50 | 28.14 | 2.24 | 1.67 | 0.79 | 6.35 | 0.63 | 0.99 | 0.03 |
| **Yingtan City** | 1154223 | 0.3000 | 0.0000 | 0.0000 | 0.0000 | 0.0000 | 0.3000 | 324.41 | 97.32 | 0.00 | 0.00 | 0.00 | 0.00 | 97.32 | 14.60 | 11.32 | 5.27 | 4.87 | 0.39 | 0.29 | 0.14 | 1.10 | 0.11 | 0.17 | 0.00 |
| Yuehu District | 287489 | 0.0000 | 0.0000 | 0.0000 | 0.0000 | 0.0000 | 0.0000 | 2097.39 | 0.00 | 0.00 | 0.00 | 0.00 | 0.00 | 0.00 | 0.00 | 0.00 | 0.00 | 0.00 | 0.00 | 0.00 | 0.00 | 0.00 | 0.00 | 0.00 | 0.00 |
| Yujiang District | 326162 | 0.0000 | 0.0000 | 0.0000 | 0.0000 | 0.0000 | 0.0000 | 351.32 | 0.00 | 0.00 | 0.00 | 0.00 | 0.00 | 0.00 | 0.00 | 0.00 | 0.00 | 0.00 | 0.00 | 0.00 | 0.00 | 0.00 | 0.00 | 0.00 | 0.00 |
| Guixi City | 540572 | 0.3000 | 0.0000 | 0.0000 | 0.0000 | 0.0000 | 0.3000 | 216.89 | 65.07 | 0.00 | 0.00 | 0.00 | 0.00 | 65.07 | 9.76 | 7.57 | 3.53 | 3.25 | 0.26 | 0.19 | 0.09 | 0.73 | 0.07 | 0.11 | 0.00 |
| **Ganzhou City** | 8970014 | 219.5500 | 51.0453 | 48.1067 | 0.7907 | 0.1773 | 319.6700 | 227.87 | 50028.19 | 11631.54 | 10961.92 | 180.17 | 40.41 | 72842.22 | 17751.34 | 13764.39 | 6411.79 | 5916.52 | 470.41 | 351.48 | 166.86 | 1334.90 | 133.14 | 207.69 | 5.33 |
| Zhanggong District | 1123276 | 10.0000 | 12.3667 | 3.2333 | 0.6533 | 0.0933 | 26.3467 | 1899.54 | 18995.43 | 23491.02 | 6141.86 | 1241.04 | 177.29 | 50046.64 | 14734.13 | 11424.84 | 5321.97 | 4910.88 | 390.45 | 291.74 | 138.50 | 1108.01 | 110.51 | 172.39 | 4.42 |
| Nankang District | 888474 | 0.0000 | 0.2000 | 8.0800 | 0.0000 | 0.0000 | 8.2800 | 509.97 | 0.00 | 101.99 | 4120.58 | 0.00 | 0.00 | 4222.57 | 2502.94 | 1940.78 | 904.06 | 834.23 | 66.33 | 49.56 | 23.53 | 188.22 | 18.77 | 29.28 | 0.75 |
| Ganxian District | 576310 | 6.9153 | 1.4627 | 0.1427 | 0.0000 | 0.0000 | 8.5207 | 192.76 | 1332.99 | 281.94 | 27.50 | 0.00 | 0.00 | 1642.43 | 301.03 | 233.42 | 108.73 | 100.33 | 7.98 | 5.96 | 2.83 | 22.64 | 2.26 | 3.52 | 0.09 |
| Xinfeng County | 673763 | 50.0000 | 6.0000 | 0.6667 | 0.0000 | 0.0000 | 56.6667 | 235.06 | 11752.77 | 1410.33 | 156.70 | 0.00 | 0.00 | 13319.81 | 2280.04 | 1767.94 | 823.55 | 759.94 | 60.42 | 45.14 | 21.43 | 171.46 | 17.10 | 26.68 | 0.68 |
| Dayu County | 264995 | 9.5667 | 5.0000 | 0.0000 | 0.0000 | 0.0000 | 14.5667 | 195.44 | 1869.69 | 977.19 | 0.00 | 0.00 | 0.00 | 2846.89 | 573.61 | 444.78 | 207.19 | 191.18 | 15.20 | 11.36 | 5.39 | 43.14 | 4.30 | 6.71 | 0.17 |
| Shangyou County | 268557 | 0.5600 | 0.7667 | 0.1267 | 0.0000 | 0.0000 | 1.4533 | 174.22 | 97.56 | 133.57 | 22.07 | 0.00 | 0.00 | 253.20 | 67.95 | 52.68 | 24.54 | 22.65 | 1.80 | 1.35 | 0.64 | 5.11 | 0.51 | 0.79 | 0.02 |
| Chongyi County | 177831 | 0.5567 | 0.2007 | 0.0000 | 0.0000 | 0.0000 | 0.7573 | 80.93 | 45.05 | 16.24 | 0.00 | 0.00 | 0.00 | 61.29 | 11.63 | 9.02 | 4.20 | 3.88 | 0.31 | 0.23 | 0.11 | 0.87 | 0.09 | 0.14 | 0.00 |
| Anyuan County | 346435 | 4.0000 | 5.9400 | 32.4867 | 0.0000 | 0.0000 | 42.4267 | 147.50 | 590.00 | 876.15 | 4791.81 | 0.00 | 0.00 | 6257.96 | 3226.43 | 2501.77 | 1165.39 | 1075.37 | 85.50 | 63.88 | 30.33 | 242.63 | 24.20 | 37.75 | 0.97 |
| Dingnan County | 209914 | 8.8313 | 0.0000 | 0.0000 | 0.0000 | 0.0000 | 8.8313 | 158.70 | 1401.54 | 0.00 | 0.00 | 0.00 | 0.00 | 1401.54 | 210.23 | 163.01 | 75.94 | 70.07 | 5.57 | 4.16 | 1.98 | 15.81 | 1.58 | 2.46 | 0.06 |
| Quannan County | 169503 | 19.1333 | 0.2000 | 0.0000 | 0.0000 | 0.0000 | 19.3333 | 110.48 | 2113.77 | 22.10 | 0.00 | 0.00 | 0.00 | 2135.87 | 323.69 | 250.99 | 116.92 | 107.89 | 8.58 | 6.41 | 3.04 | 24.34 | 2.43 | 3.79 | 0.10 |
| Ningdu County | 702394 | 0.3333 | 0.0000 | 0.0000 | 0.0000 | 0.0000 | 0.3333 | 173.55 | 57.85 | 0.00 | 0.00 | 0.00 | 0.00 | 57.85 | 8.68 | 6.73 | 3.13 | 2.89 | 0.23 | 0.17 | 0.08 | 0.65 | 0.07 | 0.10 | 0.00 |
| Yudu County | 905439 | 64.9267 | 6.5600 | 0.8667 | 0.0000 | 0.0000 | 72.3533 | 312.84 | 20311.35 | 2052.20 | 271.12 | 0.00 | 0.00 | 22634.67 | 3825.04 | 2965.93 | 1381.60 | 1274.88 | 101.36 | 75.74 | 35.96 | 287.64 | 28.69 | 44.75 | 1.15 |
| Xingguo County | 715149 | 8.9753 | 3.5140 | 1.4767 | 0.0000 | 0.0000 | 13.9660 | 222.45 | 1996.55 | 781.68 | 328.48 | 0.00 | 0.00 | 3106.71 | 731.08 | 566.88 | 264.06 | 243.67 | 19.37 | 14.48 | 6.87 | 54.98 | 5.48 | 8.55 | 0.22 |
| Huichang County | 451513 | 6.8000 | 0.7600 | 0.1600 | 0.0000 | 0.0000 | 7.7200 | 166.55 | 1132.57 | 126.58 | 26.65 | 0.00 | 0.00 | 1285.80 | 223.85 | 173.57 | 80.85 | 74.61 | 5.93 | 4.43 | 2.10 | 16.83 | 1.68 | 2.62 | 0.07 |
| Xunwu County | 280219 | 2.4847 | 4.4747 | 0.1473 | 0.0440 | 0.0840 | 7.2347 | 119.11 | 295.95 | 532.98 | 17.55 | 5.24 | 10.01 | 861.72 | 228.01 | 176.80 | 82.36 | 76.00 | 6.04 | 4.51 | 2.14 | 17.15 | 1.71 | 2.67 | 0.07 |
| Shicheng County | 283182 | 1.0000 | 1.0667 | 0.4000 | 0.0000 | 0.0000 | 2.4667 | 180.66 | 180.66 | 192.70 | 72.26 | 0.00 | 0.00 | 445.62 | 128.27 | 99.46 | 46.33 | 42.75 | 3.40 | 2.54 | 1.21 | 9.65 | 0.96 | 1.50 | 0.04 |
| Ruijin City | 613894 | 8.1333 | 1.5333 | 0.0000 | 0.0000 | 0.0000 | 9.6667 | 251.47 | 2045.31 | 385.59 | 0.00 | 0.00 | 0.00 | 2430.90 | 422.47 | 327.59 | 152.60 | 140.81 | 11.20 | 8.36 | 3.97 | 31.77 | 3.17 | 4.94 | 0.13 |
| Longnan City | 319166 | 17.3333 | 1.0000 | 0.3200 | 0.0933 | 0.0000 | 18.7467 | 193.88 | 3360.59 | 193.88 | 62.04 | 18.10 | 0.00 | 3634.61 | 613.95 | 476.06 | 221.76 | 204.63 | 16.27 | 12.16 | 5.77 | 46.17 | 4.60 | 7.18 | 0.18 |
| **Ji'an City** | 4469176 | 50.8033 | 16.6320 | 1.1840 | 0.2500 | 0.0800 | 68.9493 | 176.75 | 8979.71 | 2939.78 | 209.28 | 44.19 | 14.14 | 12187.10 | 2402.53 | 1862.93 | 867.80 | 800.76 | 63.67 | 47.57 | 22.58 | 180.67 | 18.02 | 28.11 | 0.72 |
| Jizhou District | 402669 | 17.4200 | 9.3667 | 0.8667 | 0.1733 | 0.0800 | 27.9067 | 947.48 | 16505.08 | 8874.72 | 821.15 | 164.23 | 75.80 | 26440.97 | 5830.47 | 4520.95 | 2105.97 | 1943.30 | 154.51 | 115.44 | 54.81 | 438.45 | 43.73 | 68.22 | 1.75 |
| Qingyuan District | 240730 | 2.1533 | 1.3800 | 0.1200 | 0.0733 | 0.0000 | 3.7267 | 262.21 | 564.63 | 361.85 | 31.47 | 19.23 | 0.00 | 977.18 | 227.51 | 176.41 | 82.18 | 75.83 | 6.03 | 4.50 | 2.14 | 17.11 | 1.71 | 2.66 | 0.07 |
| Ji'an County | 469513 | 16.4000 | 3.6667 | 0.0000 | 0.0000 | 0.0000 | 20.0667 | 221.15 | 3626.78 | 810.87 | 0.00 | 0.00 | 0.00 | 4437.64 | 787.28 | 610.45 | 284.36 | 262.40 | 20.86 | 15.59 | 7.40 | 59.20 | 5.90 | 9.21 | 0.24 |
| Jishui County | 421849 | 4.3333 | 0.0000 | 0.0000 | 0.0000 | 0.0000 | 4.3333 | 168.30 | 729.31 | 0.00 | 0.00 | 0.00 | 0.00 | 729.31 | 109.40 | 84.83 | 39.51 | 36.46 | 2.90 | 2.17 | 1.03 | 8.23 | 0.82 | 1.28 | 0.03 |
| Xiajiang County | 149954 | 0.4333 | 0.0000 | 0.0000 | 0.0000 | 0.0000 | 0.4333 | 115.49 | 50.05 | 0.00 | 0.00 | 0.00 | 0.00 | 50.05 | 7.51 | 5.82 | 2.71 | 2.50 | 0.20 | 0.15 | 0.07 | 0.56 | 0.06 | 0.09 | 0.00 |
| Xingan County | 286077 | 0.0000 | 0.0000 | 0.0000 | 0.0000 | 0.0000 | 0.0000 | 230.04 | 0.00 | 0.00 | 0.00 | 0.00 | 0.00 | 0.00 | 0.00 | 0.00 | 0.00 | 0.00 | 0.00 | 0.00 | 0.00 | 0.00 | 0.00 | 0.00 | 0.00 |
| Yongfeng County | 388464 | 0.0000 | 0.0000 | 0.0000 | 0.0000 | 0.0000 | 0.0000 | 143.40 | 0.00 | 0.00 | 0.00 | 0.00 | 0.00 | 0.00 | 0.00 | 0.00 | 0.00 | 0.00 | 0.00 | 0.00 | 0.00 | 0.00 | 0.00 | 0.00 | 0.00 |
| Taihe County | 467807 | 7.4900 | 0.3787 | 0.1973 | 0.0033 | 0.0000 | 8.0693 | 187.36 | 1403.35 | 70.95 | 36.97 | 0.62 | 0.00 | 1511.89 | 254.47 | 197.32 | 91.91 | 84.81 | 6.74 | 5.04 | 2.39 | 19.14 | 1.91 | 2.98 | 0.08 |
| Suichuan County | 512109 | 0.0000 | 0.0000 | 0.0000 | 0.0000 | 0.0000 | 0.0000 | 165.18 | 0.00 | 0.00 | 0.00 | 0.00 | 0.00 | 0.00 | 0.00 | 0.00 | 0.00 | 0.00 | 0.00 | 0.00 | 0.00 | 0.00 | 0.00 | 0.00 | 0.00 |
| Wan'an County | 250991 | 2.3600 | 1.6733 | 0.0000 | 0.0000 | 0.0000 | 4.0333 | 123.25 | 290.88 | 206.24 | 0.00 | 0.00 | 0.00 | 497.12 | 105.50 | 81.81 | 38.11 | 35.16 | 2.80 | 2.09 | 0.99 | 7.93 | 0.79 | 1.23 | 0.03 |
| Anfu County | 329053 | 0.2133 | 0.1667 | 0.0000 | 0.0000 | 0.0000 | 0.3800 | 117.78 | 25.13 | 19.63 | 0.00 | 0.00 | 0.00 | 44.75 | 9.66 | 7.49 | 3.49 | 3.22 | 0.26 | 0.19 | 0.09 | 0.73 | 0.07 | 0.11 | 0.00 |
| Yongxin County | 393984 | 0.0000 | 0.0000 | 0.0000 | 0.0000 | 0.0000 | 0.0000 | 180.63 | 0.00 | 0.00 | 0.00 | 0.00 | 0.00 | 0.00 | 0.00 | 0.00 | 0.00 | 0.00 | 0.00 | 0.00 | 0.00 | 0.00 | 0.00 | 0.00 | 0.00 |
| Jinggangshan City | 155976 | 0.0000 | 0.0000 | 0.0000 | 0.0000 | 0.0000 | 0.0000 | 107.39 | 0.00 | 0.00 | 0.00 | 0.00 | 0.00 | 0.00 | 0.00 | 0.00 | 0.00 | 0.00 | 0.00 | 0.00 | 0.00 | 0.00 | 0.00 | 0.00 | 0.00 |
| **Yichun City** | 5007702 | 3.1273 | 1.2813 | 0.1867 | 0.0133 | 0.0000 | 4.6087 | 268.23 | 838.85 | 343.69 | 50.07 | 3.58 | 0.00 | 1236.18 | 261.84 | 203.03 | 94.58 | 87.27 | 6.94 | 5.18 | 2.46 | 19.69 | 1.96 | 3.06 | 0.08 |
| Yuanzhou District | 1121707 | 0.0000 | 0.0000 | 0.0000 | 0.0000 | 0.0000 | 0.0000 | 442.07 | 0.00 | 0.00 | 0.00 | 0.00 | 0.00 | 0.00 | 0.00 | 0.00 | 0.00 | 0.00 | 0.00 | 0.00 | 0.00 | 0.00 | 0.00 | 0.00 | 0.00 |
| Fengxin County | 268617 | 0.0000 | 0.0000 | 0.0000 | 0.0000 | 0.0000 | 0.0000 | 162.82 | 0.00 | 0.00 | 0.00 | 0.00 | 0.00 | 0.00 | 0.00 | 0.00 | 0.00 | 0.00 | 0.00 | 0.00 | 0.00 | 0.00 | 0.00 | 0.00 | 0.00 |
| Wanzai County | 486435 | 0.0000 | 0.0000 | 0.0000 | 0.0000 | 0.0000 | 0.0000 | 283.45 | 0.00 | 0.00 | 0.00 | 0.00 | 0.00 | 0.00 | 0.00 | 0.00 | 0.00 | 0.00 | 0.00 | 0.00 | 0.00 | 0.00 | 0.00 | 0.00 | 0.00 |
| Shanggao County | 343767 | 1.6667 | 0.8667 | 0.0000 | 0.0000 | 0.0000 | 2.5333 | 255.34 | 425.57 | 221.30 | 0.00 | 0.00 | 0.00 | 646.87 | 130.22 | 100.98 | 47.04 | 43.40 | 3.45 | 2.58 | 1.22 | 9.79 | 0.98 | 1.52 | 0.04 |
| Yifeng County | 252974 | 0.0000 | 0.0000 | 0.0000 | 0.0000 | 0.0000 | 0.0000 | 130.58 | 0.00 | 0.00 | 0.00 | 0.00 | 0.00 | 0.00 | 0.00 | 0.00 | 0.00 | 0.00 | 0.00 | 0.00 | 0.00 | 0.00 | 0.00 | 0.00 | 0.00 |
| Jing'an County | 121800 | 0.0000 | 0.0000 | 0.0000 | 0.0000 | 0.0000 | 0.0000 | 88.40 | 0.00 | 0.00 | 0.00 | 0.00 | 0.00 | 0.00 | 0.00 | 0.00 | 0.00 | 0.00 | 0.00 | 0.00 | 0.00 | 0.00 | 0.00 | 0.00 | 0.00 |
| Tonggu County | 116418 | 0.0000 | 0.0000 | 0.0000 | 0.0000 | 0.0000 | 0.0000 | 74.98 | 0.00 | 0.00 | 0.00 | 0.00 | 0.00 | 0.00 | 0.00 | 0.00 | 0.00 | 0.00 | 0.00 | 0.00 | 0.00 | 0.00 | 0.00 | 0.00 | 0.00 |
| Fengcheng City | 1065641 | 0.4633 | 0.4133 | 0.1867 | 0.0133 | 0.0000 | 1.0767 | 375.70 | 174.08 | 155.29 | 70.13 | 5.01 | 0.00 | 404.51 | 118.78 | 92.11 | 42.90 | 39.59 | 3.15 | 2.35 | 1.12 | 8.93 | 0.89 | 1.39 | 0.04 |
| Zhangshu City | 485649 | 0.5640 | 0.0013 | 0.0000 | 0.0000 | 0.0000 | 0.5653 | 376.79 | 212.51 | 0.50 | 0.00 | 0.00 | 0.00 | 213.01 | 32.03 | 24.83 | 11.57 | 10.67 | 0.85 | 0.63 | 0.30 | 2.41 | 0.24 | 0.37 | 0.01 |
| Gao'an City | 744694 | 0.4333 | 0.0000 | 0.0000 | 0.0000 | 0.0000 | 0.4333 | 306.86 | 132.97 | 0.00 | 0.00 | 0.00 | 0.00 | 132.97 | 19.95 | 15.47 | 7.20 | 6.65 | 0.53 | 0.39 | 0.19 | 1.50 | 0.15 | 0.23 | 0.01 |
| **Fuzhou City** | 3614866 | 0.0029 | 0.0000 | 0.0000 | 0.0000 | 0.0000 | 0.0029 | 192.26 | 0.55 | 0.00 | 0.00 | 0.00 | 0.00 | 0.55 | 0.08 | 0.06 | 0.03 | 0.03 | 0.00 | 0.00 | 0.00 | 0.01 | 0.00 | 0.00 | 0.00 |
| Linchuan District | 1102421 | 0.0000 | 0.0000 | 0.0000 | 0.0000 | 0.0000 | 0.0000 | 518.18 | 0.00 | 0.00 | 0.00 | 0.00 | 0.00 | 0.00 | 0.00 | 0.00 | 0.00 | 0.00 | 0.00 | 0.00 | 0.00 | 0.00 | 0.00 | 0.00 | 0.00 |
| Dongxiang District | 383846 | 0.0000 | 0.0000 | 0.0000 | 0.0000 | 0.0000 | 0.0000 | 302.77 | 0.00 | 0.00 | 0.00 | 0.00 | 0.00 | 0.00 | 0.00 | 0.00 | 0.00 | 0.00 | 0.00 | 0.00 | 0.00 | 0.00 | 0.00 | 0.00 | 0.00 |
| Nancheng County | 286260 | 0.0000 | 0.0000 | 0.0000 | 0.0000 | 0.0000 | 0.0000 | 167.17 | 0.00 | 0.00 | 0.00 | 0.00 | 0.00 | 0.00 | 0.00 | 0.00 | 0.00 | 0.00 | 0.00 | 0.00 | 0.00 | 0.00 | 0.00 | 0.00 | 0.00 |
| Lichuan County | 205205 | 0.0000 | 0.0000 | 0.0000 | 0.0000 | 0.0000 | 0.0000 | 120.14 | 0.00 | 0.00 | 0.00 | 0.00 | 0.00 | 0.00 | 0.00 | 0.00 | 0.00 | 0.00 | 0.00 | 0.00 | 0.00 | 0.00 | 0.00 | 0.00 | 0.00 |
| Nanfeng County | 271888 | 0.0029 | 0.0000 | 0.0000 | 0.0000 | 0.0000 | 0.0029 | 142.21 | 0.41 | 0.00 | 0.00 | 0.00 | 0.00 | 0.41 | 0.06 | 0.05 | 0.02 | 0.02 | 0.00 | 0.00 | 0.00 | 0.00 | 0.00 | 0.00 | 0.00 |
| Chongren County | 302181 | 0.0000 | 0.0000 | 0.0000 | 0.0000 | 0.0000 | 0.0000 | 198.80 | 0.00 | 0.00 | 0.00 | 0.00 | 0.00 | 0.00 | 0.00 | 0.00 | 0.00 | 0.00 | 0.00 | 0.00 | 0.00 | 0.00 | 0.00 | 0.00 | 0.00 |
| Le'an County | 307909 | 0.0000 | 0.0000 | 0.0000 | 0.0000 | 0.0000 | 0.0000 | 127.73 | 0.00 | 0.00 | 0.00 | 0.00 | 0.00 | 0.00 | 0.00 | 0.00 | 0.00 | 0.00 | 0.00 | 0.00 | 0.00 | 0.00 | 0.00 | 0.00 | 0.00 |
| Yihuang County | 201127 | 0.0000 | 0.0000 | 0.0000 | 0.0000 | 0.0000 | 0.0000 | 103.73 | 0.00 | 0.00 | 0.00 | 0.00 | 0.00 | 0.00 | 0.00 | 0.00 | 0.00 | 0.00 | 0.00 | 0.00 | 0.00 | 0.00 | 0.00 | 0.00 | 0.00 |
| Jinxi County | 253828 | 0.0000 | 0.0000 | 0.0000 | 0.0000 | 0.0000 | 0.0000 | 187.66 | 0.00 | 0.00 | 0.00 | 0.00 | 0.00 | 0.00 | 0.00 | 0.00 | 0.00 | 0.00 | 0.00 | 0.00 | 0.00 | 0.00 | 0.00 | 0.00 | 0.00 |
| Zixi County | 95826 | 0.0000 | 0.0000 | 0.0000 | 0.0000 | 0.0000 | 0.0000 | 76.75 | 0.00 | 0.00 | 0.00 | 0.00 | 0.00 | 0.00 | 0.00 | 0.00 | 0.00 | 0.00 | 0.00 | 0.00 | 0.00 | 0.00 | 0.00 | 0.00 | 0.00 |
| Guangchang County | 204375 | 0.0000 | 0.0000 | 0.0000 | 0.0000 | 0.0000 | 0.0000 | 127.48 | 0.00 | 0.00 | 0.00 | 0.00 | 0.00 | 0.00 | 0.00 | 0.00 | 0.00 | 0.00 | 0.00 | 0.00 | 0.00 | 0.00 | 0.00 | 0.00 | 0.00 |
| **Shangrao City** | 6491088 | 1.0233 | 0.3067 | 0.0667 | 0.0000 | 0.0000 | 1.3967 | 285.39 | 292.05 | 87.52 | 19.03 | 0.00 | 0.00 | 398.60 | 81.48 | 63.18 | 29.43 | 27.16 | 2.16 | 1.61 | 0.77 | 6.13 | 0.61 | 0.95 | 0.02 |
| Xinshu District | 545134 | 0.6667 | 0.0000 | 0.0000 | 0.0000 | 0.0000 | 0.6667 | 1724.67 | 1149.78 | 0.00 | 0.00 | 0.00 | 0.00 | 1149.78 | 172.47 | 133.73 | 62.30 | 57.48 | 4.57 | 3.41 | 1.62 | 12.97 | 1.29 | 2.02 | 0.05 |
| Guangfeng District | 775364 | 0.0000 | 0.0000 | 0.0000 | 0.0000 | 0.0000 | 0.0000 | 563.86 | 0.00 | 0.00 | 0.00 | 0.00 | 0.00 | 0.00 | 0.00 | 0.00 | 0.00 | 0.00 | 0.00 | 0.00 | 0.00 | 0.00 | 0.00 | 0.00 | 0.00 |
| Guangxin District | 748265 | 0.3333 | 0.2667 | 0.0667 | 0.0000 | 0.0000 | 0.6667 | 335.36 | 111.79 | 89.43 | 22.36 | 0.00 | 0.00 | 223.58 | 57.01 | 44.21 | 20.59 | 19.00 | 1.51 | 1.13 | 0.54 | 4.29 | 0.43 | 0.67 | 0.02 |
| Yushan County | 519357 | 0.0000 | 0.0400 | 0.0000 | 0.0000 | 0.0000 | 0.0400 | 300.00 | 0.00 | 12.00 | 0.00 | 0.00 | 0.00 | 12.00 | 3.60 | 2.79 | 1.30 | 1.20 | 0.10 | 0.07 | 0.03 | 0.27 | 0.03 | 0.04 | 0.00 |
| Qianshan County | 386200 | 0.0233 | 0.0000 | 0.0000 | 0.0000 | 0.0000 | 0.0233 | 177.21 | 4.13 | 0.00 | 0.00 | 0.00 | 0.00 | 4.13 | 0.62 | 0.48 | 0.22 | 0.21 | 0.02 | 0.01 | 0.01 | 0.05 | 0.00 | 0.01 | 0.00 |
| Hengfeng County | 187326 | 0.0000 | 0.0000 | 0.0000 | 0.0000 | 0.0000 | 0.0000 | 286.25 | 0.00 | 0.00 | 0.00 | 0.00 | 0.00 | 0.00 | 0.00 | 0.00 | 0.00 | 0.00 | 0.00 | 0.00 | 0.00 | 0.00 | 0.00 | 0.00 | 0.00 |
| Yiyang County | 338817 | 0.0000 | 0.0000 | 0.0000 | 0.0000 | 0.0000 | 0.0000 | 215.33 | 0.00 | 0.00 | 0.00 | 0.00 | 0.00 | 0.00 | 0.00 | 0.00 | 0.00 | 0.00 | 0.00 | 0.00 | 0.00 | 0.00 | 0.00 | 0.00 | 0.00 |
| Yugan County | 840498 | 0.0000 | 0.0000 | 0.0000 | 0.0000 | 0.0000 | 0.0000 | 356.69 | 0.00 | 0.00 | 0.00 | 0.00 | 0.00 | 0.00 | 0.00 | 0.00 | 0.00 | 0.00 | 0.00 | 0.00 | 0.00 | 0.00 | 0.00 | 0.00 | 0.00 |
| Poyang County | 1184097 | 0.0000 | 0.0000 | 0.0000 | 0.0000 | 0.0000 | 0.0000 | 287.01 | 0.00 | 0.00 | 0.00 | 0.00 | 0.00 | 0.00 | 0.00 | 0.00 | 0.00 | 0.00 | 0.00 | 0.00 | 0.00 | 0.00 | 0.00 | 0.00 | 0.00 |
| Wannian County | 357449 | 0.0000 | 0.0000 | 0.0000 | 0.0000 | 0.0000 | 0.0000 | 310.34 | 0.00 | 0.00 | 0.00 | 0.00 | 0.00 | 0.00 | 0.00 | 0.00 | 0.00 | 0.00 | 0.00 | 0.00 | 0.00 | 0.00 | 0.00 | 0.00 | 0.00 |
| Wuyuan County | 314985 | 0.0000 | 0.0000 | 0.0000 | 0.0000 | 0.0000 | 0.0000 | 106.09 | 0.00 | 0.00 | 0.00 | 0.00 | 0.00 | 0.00 | 0.00 | 0.00 | 0.00 | 0.00 | 0.00 | 0.00 | 0.00 | 0.00 | 0.00 | 0.00 | 0.00 |
| Dexing City | 293596 | 0.0000 | 0.0000 | 0.0000 | 0.0000 | 0.0000 | 0.0000 | 141.11 | 0.00 | 0.00 | 0.00 | 0.00 | 0.00 | 0.00 | 0.00 | 0.00 | 0.00 | 0.00 | 0.00 | 0.00 | 0.00 | 0.00 | 0.00 | 0.00 | 0.00 |
| **Hubei Province** | 57752557 | 3.2833 | 1.0833 | 0.2976 | 0.1620 | 0.0000 | 4.8262 | 316.01 | 1037.57 | 342.35 | 94.04 | 51.19 | 0.00 | 1525.15 | 355.72 | 275.82 | 128.48 | 118.56 | 9.43 | 7.04 | 3.34 | 26.75 | 2.67 | 4.16 | 0.11 |
| **Wuhan City** | 12447718 | 0.0333 | 0.0000 | 0.0000 | 0.0000 | 0.0000 | 0.0333 | 1451.17 | 48.37 | 0.00 | 0.00 | 0.00 | 0.00 | 48.37 | 7.26 | 5.63 | 2.62 | 2.42 | 0.19 | 0.14 | 0.07 | 0.55 | 0.05 | 0.08 | 0.00 |
| Jiang'an District | 965260 | 0.0000 | 0.0000 | 0.0000 | 0.0000 | 0.0000 | 0.0000 | 12091.44 | 0.00 | 0.00 | 0.00 | 0.00 | 0.00 | 0.00 | 0.00 | 0.00 | 0.00 | 0.00 | 0.00 | 0.00 | 0.00 | 0.00 | 0.00 | 0.00 | 0.00 |
| Jianghan District | 647932 | 0.0000 | 0.0000 | 0.0000 | 0.0000 | 0.0000 | 0.0000 | 22678.75 | 0.00 | 0.00 | 0.00 | 0.00 | 0.00 | 0.00 | 0.00 | 0.00 | 0.00 | 0.00 | 0.00 | 0.00 | 0.00 | 0.00 | 0.00 | 0.00 | 0.00 |
| Qiaokou District | 666661 | 0.0000 | 0.0000 | 0.0000 | 0.0000 | 0.0000 | 0.0000 | 15801.40 | 0.00 | 0.00 | 0.00 | 0.00 | 0.00 | 0.00 | 0.00 | 0.00 | 0.00 | 0.00 | 0.00 | 0.00 | 0.00 | 0.00 | 0.00 | 0.00 | 0.00 |
| Hanyang District | 837263 | 0.0000 | 0.0000 | 0.0000 | 0.0000 | 0.0000 | 0.0000 | 7389.79 | 0.00 | 0.00 | 0.00 | 0.00 | 0.00 | 0.00 | 0.00 | 0.00 | 0.00 | 0.00 | 0.00 | 0.00 | 0.00 | 0.00 | 0.00 | 0.00 | 0.00 |
| Wuchang District | 1102188 | 0.0000 | 0.0000 | 0.0000 | 0.0000 | 0.0000 | 0.0000 | 12308.07 | 0.00 | 0.00 | 0.00 | 0.00 | 0.00 | 0.00 | 0.00 | 0.00 | 0.00 | 0.00 | 0.00 | 0.00 | 0.00 | 0.00 | 0.00 | 0.00 | 0.00 |
| Qingshan District | 431818 | 0.0000 | 0.0000 | 0.0000 | 0.0000 | 0.0000 | 0.0000 | 9024.41 | 0.00 | 0.00 | 0.00 | 0.00 | 0.00 | 0.00 | 0.00 | 0.00 | 0.00 | 0.00 | 0.00 | 0.00 | 0.00 | 0.00 | 0.00 | 0.00 | 0.00 |
| Hongshan District | 2554403 | 0.0000 | 0.0000 | 0.0000 | 0.0000 | 0.0000 | 0.0000 | 4511.64 | 0.00 | 0.00 | 0.00 | 0.00 | 0.00 | 0.00 | 0.00 | 0.00 | 0.00 | 0.00 | 0.00 | 0.00 | 0.00 | 0.00 | 0.00 | 0.00 | 0.00 |
| Dongxihu District | 845782 | 0.0000 | 0.0000 | 0.0000 | 0.0000 | 0.0000 | 0.0000 | 1737.97 | 0.00 | 0.00 | 0.00 | 0.00 | 0.00 | 0.00 | 0.00 | 0.00 | 0.00 | 0.00 | 0.00 | 0.00 | 0.00 | 0.00 | 0.00 | 0.00 | 0.00 |
| Hannan District | 145103 | 0.0000 | 0.0000 | 0.0000 | 0.0000 | 0.0000 | 0.0000 | 504.06 | 0.00 | 0.00 | 0.00 | 0.00 | 0.00 | 0.00 | 0.00 | 0.00 | 0.00 | 0.00 | 0.00 | 0.00 | 0.00 | 0.00 | 0.00 | 0.00 | 0.00 |
| Caidian District | 930818 | 0.0333 | 0.0000 | 0.0000 | 0.0000 | 0.0000 | 0.0333 | 850.22 | 28.34 | 0.00 | 0.00 | 0.00 | 0.00 | 28.34 | 4.25 | 3.30 | 1.54 | 1.42 | 0.11 | 0.08 | 0.04 | 0.32 | 0.03 | 0.05 | 0.00 |
| Jiangxia District | 1308469 | 0.0000 | 0.0000 | 0.0000 | 0.0000 | 0.0000 | 0.0000 | 648.43 | 0.00 | 0.00 | 0.00 | 0.00 | 0.00 | 0.00 | 0.00 | 0.00 | 0.00 | 0.00 | 0.00 | 0.00 | 0.00 | 0.00 | 0.00 | 0.00 | 0.00 |
| Huangpi District | 1151644 | 0.0000 | 0.0000 | 0.0000 | 0.0000 | 0.0000 | 0.0000 | 512.50 | 0.00 | 0.00 | 0.00 | 0.00 | 0.00 | 0.00 | 0.00 | 0.00 | 0.00 | 0.00 | 0.00 | 0.00 | 0.00 | 0.00 | 0.00 | 0.00 | 0.00 |
| Xinzhou District | 860377 | 0.0000 | 0.0000 | 0.0000 | 0.0000 | 0.0000 | 0.0000 | 582.95 | 0.00 | 0.00 | 0.00 | 0.00 | 0.00 | 0.00 | 0.00 | 0.00 | 0.00 | 0.00 | 0.00 | 0.00 | 0.00 | 0.00 | 0.00 | 0.00 | 0.00 |
| **Huangshi City** | 2469079 | 0.0000 | 0.0000 | 0.0000 | 0.0000 | 0.0000 | 0.0000 | 539.67 | 0.00 | 0.00 | 0.00 | 0.00 | 0.00 | 0.00 | 0.00 | 0.00 | 0.00 | 0.00 | 0.00 | 0.00 | 0.00 | 0.00 | 0.00 | 0.00 | 0.00 |
| Huangshigang District | 241589 | 0.0000 | 0.0000 | 0.0000 | 0.0000 | 0.0000 | 0.0000 | 8606.66 | 0.00 | 0.00 | 0.00 | 0.00 | 0.00 | 0.00 | 0.00 | 0.00 | 0.00 | 0.00 | 0.00 | 0.00 | 0.00 | 0.00 | 0.00 | 0.00 | 0.00 |
| Xisaishan District | 197217 | 0.0000 | 0.0000 | 0.0000 | 0.0000 | 0.0000 | 0.0000 | 1715.38 | 0.00 | 0.00 | 0.00 | 0.00 | 0.00 | 0.00 | 0.00 | 0.00 | 0.00 | 0.00 | 0.00 | 0.00 | 0.00 | 0.00 | 0.00 | 0.00 | 0.00 |
| Xialu District | 215181 | 0.0000 | 0.0000 | 0.0000 | 0.0000 | 0.0000 | 0.0000 | 3471.78 | 0.00 | 0.00 | 0.00 | 0.00 | 0.00 | 0.00 | 0.00 | 0.00 | 0.00 | 0.00 | 0.00 | 0.00 | 0.00 | 0.00 | 0.00 | 0.00 | 0.00 |
| Tieshan District | 41907 | 0.0000 | 0.0000 | 0.0000 | 0.0000 | 0.0000 | 0.0000 | 1446.07 | 0.00 | 0.00 | 0.00 | 0.00 | 0.00 | 0.00 | 0.00 | 0.00 | 0.00 | 0.00 | 0.00 | 0.00 | 0.00 | 0.00 | 0.00 | 0.00 | 0.00 |
| Yangxin County | 901971 | 0.0000 | 0.0000 | 0.0000 | 0.0000 | 0.0000 | 0.0000 | 324.22 | 0.00 | 0.00 | 0.00 | 0.00 | 0.00 | 0.00 | 0.00 | 0.00 | 0.00 | 0.00 | 0.00 | 0.00 | 0.00 | 0.00 | 0.00 | 0.00 | 0.00 |
| Daye City | 871214 | 0.0000 | 0.0000 | 0.0000 | 0.0000 | 0.0000 | 0.0000 | 558.76 | 0.00 | 0.00 | 0.00 | 0.00 | 0.00 | 0.00 | 0.00 | 0.00 | 0.00 | 0.00 | 0.00 | 0.00 | 0.00 | 0.00 | 0.00 | 0.00 | 0.00 |
| **Shiyan City** | 3209004 | 0.0000 | 0.0000 | 0.0000 | 0.0000 | 0.0000 | 0.0000 | 135.48 | 0.00 | 0.00 | 0.00 | 0.00 | 0.00 | 0.00 | 0.00 | 0.00 | 0.00 | 0.00 | 0.00 | 0.00 | 0.00 | 0.00 | 0.00 | 0.00 | 0.00 |
| Maojian District | 601548 | 0.0000 | 0.0000 | 0.0000 | 0.0000 | 0.0000 | 0.0000 | 1122.98 | 0.00 | 0.00 | 0.00 | 0.00 | 0.00 | 0.00 | 0.00 | 0.00 | 0.00 | 0.00 | 0.00 | 0.00 | 0.00 | 0.00 | 0.00 | 0.00 | 0.00 |
| Zhangwan District | 431859 | 0.0000 | 0.0000 | 0.0000 | 0.0000 | 0.0000 | 0.0000 | 656.24 | 0.00 | 0.00 | 0.00 | 0.00 | 0.00 | 0.00 | 0.00 | 0.00 | 0.00 | 0.00 | 0.00 | 0.00 | 0.00 | 0.00 | 0.00 | 0.00 | 0.00 |
| Yunyang District | 395222 | 0.0000 | 0.0000 | 0.0000 | 0.0000 | 0.0000 | 0.0000 | 103.13 | 0.00 | 0.00 | 0.00 | 0.00 | 0.00 | 0.00 | 0.00 | 0.00 | 0.00 | 0.00 | 0.00 | 0.00 | 0.00 | 0.00 | 0.00 | 0.00 | 0.00 |
| Yunxi County | 371012 | 0.0000 | 0.0000 | 0.0000 | 0.0000 | 0.0000 | 0.0000 | 105.77 | 0.00 | 0.00 | 0.00 | 0.00 | 0.00 | 0.00 | 0.00 | 0.00 | 0.00 | 0.00 | 0.00 | 0.00 | 0.00 | 0.00 | 0.00 | 0.00 | 0.00 |
| Zhushan County | 346069 | 0.0000 | 0.0000 | 0.0000 | 0.0000 | 0.0000 | 0.0000 | 96.42 | 0.00 | 0.00 | 0.00 | 0.00 | 0.00 | 0.00 | 0.00 | 0.00 | 0.00 | 0.00 | 0.00 | 0.00 | 0.00 | 0.00 | 0.00 | 0.00 | 0.00 |
| Zhuxi County | 283578 | 0.0000 | 0.0000 | 0.0000 | 0.0000 | 0.0000 | 0.0000 | 85.79 | 0.00 | 0.00 | 0.00 | 0.00 | 0.00 | 0.00 | 0.00 | 0.00 | 0.00 | 0.00 | 0.00 | 0.00 | 0.00 | 0.00 | 0.00 | 0.00 | 0.00 |
| Fang County | 369776 | 0.0000 | 0.0000 | 0.0000 | 0.0000 | 0.0000 | 0.0000 | 72.14 | 0.00 | 0.00 | 0.00 | 0.00 | 0.00 | 0.00 | 0.00 | 0.00 | 0.00 | 0.00 | 0.00 | 0.00 | 0.00 | 0.00 | 0.00 | 0.00 | 0.00 |
| Danjiangkou City | 409940 | 0.0000 | 0.0000 | 0.0000 | 0.0000 | 0.0000 | 0.0000 | 130.92 | 0.00 | 0.00 | 0.00 | 0.00 | 0.00 | 0.00 | 0.00 | 0.00 | 0.00 | 0.00 | 0.00 | 0.00 | 0.00 | 0.00 | 0.00 | 0.00 | 0.00 |
| **Yichang City** | 3762407 | 0.0233 | 0.0033 | 0.0000 | 0.0000 | 0.0000 | 0.0267 | 177.35 | 4.14 | 0.59 | 0.00 | 0.00 | 0.00 | 4.73 | 0.80 | 0.62 | 0.29 | 0.27 | 0.02 | 0.02 | 0.01 | 0.06 | 0.01 | 0.01 | 0.00 |
| Xiling District | 506520 | 0.0000 | 0.0033 | 0.0000 | 0.0000 | 0.0000 | 0.0033 | 6296.87 | 0.00 | 20.99 | 0.00 | 0.00 | 0.00 | 20.99 | 6.30 | 4.88 | 2.27 | 2.10 | 0.17 | 0.12 | 0.06 | 0.47 | 0.05 | 0.07 | 0.00 |
| Wujiagang District | 336294 | 0.0233 | 0.0000 | 0.0000 | 0.0000 | 0.0000 | 0.0233 | 4050.76 | 94.52 | 0.00 | 0.00 | 0.00 | 0.00 | 94.52 | 14.18 | 10.99 | 5.12 | 4.73 | 0.38 | 0.28 | 0.13 | 1.07 | 0.11 | 0.17 | 0.00 |
| Dianjun District | 101649 | 0.0000 | 0.0000 | 0.0000 | 0.0000 | 0.0000 | 0.0000 | 189.59 | 0.00 | 0.00 | 0.00 | 0.00 | 0.00 | 0.00 | 0.00 | 0.00 | 0.00 | 0.00 | 0.00 | 0.00 | 0.00 | 0.00 | 0.00 | 0.00 | 0.00 |
| Xiaoting District | 68728 | 0.0000 | 0.0000 | 0.0000 | 0.0000 | 0.0000 | 0.0000 | 570.17 | 0.00 | 0.00 | 0.00 | 0.00 | 0.00 | 0.00 | 0.00 | 0.00 | 0.00 | 0.00 | 0.00 | 0.00 | 0.00 | 0.00 | 0.00 | 0.00 | 0.00 |
| Yiling District | 534408 | 0.0000 | 0.0000 | 0.0000 | 0.0000 | 0.0000 | 0.0000 | 156.87 | 0.00 | 0.00 | 0.00 | 0.00 | 0.00 | 0.00 | 0.00 | 0.00 | 0.00 | 0.00 | 0.00 | 0.00 | 0.00 | 0.00 | 0.00 | 0.00 | 0.00 |
| Yuan'an County | 166174 | 0.0000 | 0.0000 | 0.0000 | 0.0000 | 0.0000 | 0.0000 | 95.35 | 0.00 | 0.00 | 0.00 | 0.00 | 0.00 | 0.00 | 0.00 | 0.00 | 0.00 | 0.00 | 0.00 | 0.00 | 0.00 | 0.00 | 0.00 | 0.00 | 0.00 |
| Xingshan County | 133886 | 0.0000 | 0.0000 | 0.0000 | 0.0000 | 0.0000 | 0.0000 | 57.94 | 0.00 | 0.00 | 0.00 | 0.00 | 0.00 | 0.00 | 0.00 | 0.00 | 0.00 | 0.00 | 0.00 | 0.00 | 0.00 | 0.00 | 0.00 | 0.00 | 0.00 |
| Zigui County | 299642 | 0.0000 | 0.0000 | 0.0000 | 0.0000 | 0.0000 | 0.0000 | 131.76 | 0.00 | 0.00 | 0.00 | 0.00 | 0.00 | 0.00 | 0.00 | 0.00 | 0.00 | 0.00 | 0.00 | 0.00 | 0.00 | 0.00 | 0.00 | 0.00 | 0.00 |
| Changyang Tujia Autonomous County | 305740 | 0.0000 | 0.0000 | 0.0000 | 0.0000 | 0.0000 | 0.0000 | 89.46 | 0.00 | 0.00 | 0.00 | 0.00 | 0.00 | 0.00 | 0.00 | 0.00 | 0.00 | 0.00 | 0.00 | 0.00 | 0.00 | 0.00 | 0.00 | 0.00 | 0.00 |
| Wufeng Tujia Autonomous County | 156026 | 0.0000 | 0.0000 | 0.0000 | 0.0000 | 0.0000 | 0.0000 | 65.94 | 0.00 | 0.00 | 0.00 | 0.00 | 0.00 | 0.00 | 0.00 | 0.00 | 0.00 | 0.00 | 0.00 | 0.00 | 0.00 | 0.00 | 0.00 | 0.00 | 0.00 |
| Yidu City | 347388 | 0.0000 | 0.0000 | 0.0000 | 0.0000 | 0.0000 | 0.0000 | 257.11 | 0.00 | 0.00 | 0.00 | 0.00 | 0.00 | 0.00 | 0.00 | 0.00 | 0.00 | 0.00 | 0.00 | 0.00 | 0.00 | 0.00 | 0.00 | 0.00 | 0.00 |
| Dangyang City | 397465 | 0.0000 | 0.0000 | 0.0000 | 0.0000 | 0.0000 | 0.0000 | 184.90 | 0.00 | 0.00 | 0.00 | 0.00 | 0.00 | 0.00 | 0.00 | 0.00 | 0.00 | 0.00 | 0.00 | 0.00 | 0.00 | 0.00 | 0.00 | 0.00 | 0.00 |
| Zhijiang City | 408487 | 0.0000 | 0.0000 | 0.0000 | 0.0000 | 0.0000 | 0.0000 | 296.89 | 0.00 | 0.00 | 0.00 | 0.00 | 0.00 | 0.00 | 0.00 | 0.00 | 0.00 | 0.00 | 0.00 | 0.00 | 0.00 | 0.00 | 0.00 | 0.00 | 0.00 |
| **Xiangyang City** | 5260951 | 0.0000 | 0.0000 | 0.0000 | 0.0000 | 0.0000 | 0.0000 | 266.66 | 0.00 | 0.00 | 0.00 | 0.00 | 0.00 | 0.00 | 0.00 | 0.00 | 0.00 | 0.00 | 0.00 | 0.00 | 0.00 | 0.00 | 0.00 | 0.00 | 0.00 |
| Xiangcheng District | 475611 | 0.0000 | 0.0000 | 0.0000 | 0.0000 | 0.0000 | 0.0000 | 723.29 | 0.00 | 0.00 | 0.00 | 0.00 | 0.00 | 0.00 | 0.00 | 0.00 | 0.00 | 0.00 | 0.00 | 0.00 | 0.00 | 0.00 | 0.00 | 0.00 | 0.00 |
| Fancheng District | 920794 | 0.0000 | 0.0000 | 0.0000 | 0.0000 | 0.0000 | 0.0000 | 1873.63 | 0.00 | 0.00 | 0.00 | 0.00 | 0.00 | 0.00 | 0.00 | 0.00 | 0.00 | 0.00 | 0.00 | 0.00 | 0.00 | 0.00 | 0.00 | 0.00 | 0.00 |
| Xiangzhou District | 923235 | 0.0000 | 0.0000 | 0.0000 | 0.0000 | 0.0000 | 0.0000 | 366.32 | 0.00 | 0.00 | 0.00 | 0.00 | 0.00 | 0.00 | 0.00 | 0.00 | 0.00 | 0.00 | 0.00 | 0.00 | 0.00 | 0.00 | 0.00 | 0.00 | 0.00 |
| Nanzhang County | 455690 | 0.0000 | 0.0000 | 0.0000 | 0.0000 | 0.0000 | 0.0000 | 118.18 | 0.00 | 0.00 | 0.00 | 0.00 | 0.00 | 0.00 | 0.00 | 0.00 | 0.00 | 0.00 | 0.00 | 0.00 | 0.00 | 0.00 | 0.00 | 0.00 | 0.00 |
| Gucheng County | 483293 | 0.0000 | 0.0000 | 0.0000 | 0.0000 | 0.0000 | 0.0000 | 189.95 | 0.00 | 0.00 | 0.00 | 0.00 | 0.00 | 0.00 | 0.00 | 0.00 | 0.00 | 0.00 | 0.00 | 0.00 | 0.00 | 0.00 | 0.00 | 0.00 | 0.00 |
| Baokang County | 223622 | 0.0000 | 0.0000 | 0.0000 | 0.0000 | 0.0000 | 0.0000 | 69.45 | 0.00 | 0.00 | 0.00 | 0.00 | 0.00 | 0.00 | 0.00 | 0.00 | 0.00 | 0.00 | 0.00 | 0.00 | 0.00 | 0.00 | 0.00 | 0.00 | 0.00 |
| Laohekou City | 420495 | 0.0000 | 0.0000 | 0.0000 | 0.0000 | 0.0000 | 0.0000 | 402.73 | 0.00 | 0.00 | 0.00 | 0.00 | 0.00 | 0.00 | 0.00 | 0.00 | 0.00 | 0.00 | 0.00 | 0.00 | 0.00 | 0.00 | 0.00 | 0.00 | 0.00 |
| Zaoyang City | 888794 | 0.0000 | 0.0000 | 0.0000 | 0.0000 | 0.0000 | 0.0000 | 270.83 | 0.00 | 0.00 | 0.00 | 0.00 | 0.00 | 0.00 | 0.00 | 0.00 | 0.00 | 0.00 | 0.00 | 0.00 | 0.00 | 0.00 | 0.00 | 0.00 | 0.00 |
| Yicheng City | 469417 | 0.0000 | 0.0000 | 0.0000 | 0.0000 | 0.0000 | 0.0000 | 222.05 | 0.00 | 0.00 | 0.00 | 0.00 | 0.00 | 0.00 | 0.00 | 0.00 | 0.00 | 0.00 | 0.00 | 0.00 | 0.00 | 0.00 | 0.00 | 0.00 | 0.00 |
| **Ezhou City** | 1079353 | 3.2267 | 1.0800 | 0.2667 | 0.1620 | 0.0000 | 4.7353 | 682.41 | 2201.92 | 737.01 | 181.98 | 110.55 | 0.00 | 3231.46 | 749.02 | 580.79 | 270.54 | 249.65 | 19.85 | 14.83 | 7.04 | 56.33 | 5.62 | 8.76 | 0.22 |
| Liangzihu District | 126822 | 0.0000 | 0.0000 | 0.0000 | 0.0000 | 0.0000 | 0.0000 | 260.82 | 0.00 | 0.00 | 0.00 | 0.00 | 0.00 | 0.00 | 0.00 | 0.00 | 0.00 | 0.00 | 0.00 | 0.00 | 0.00 | 0.00 | 0.00 | 0.00 | 0.00 |
| Huarong District | 256834 | 3.2267 | 1.0800 | 0.2667 | 0.1620 | 0.0000 | 4.7353 | 522.80 | 1686.89 | 564.62 | 139.41 | 84.69 | 0.00 | 2475.61 | 573.82 | 444.94 | 207.26 | 191.25 | 15.21 | 11.36 | 5.39 | 43.15 | 4.30 | 6.71 | 0.17 |
| Echeng District | 695697 | 0.0000 | 0.0000 | 0.0000 | 0.0000 | 0.0000 | 0.0000 | 1151.53 | 0.00 | 0.00 | 0.00 | 0.00 | 0.00 | 0.00 | 0.00 | 0.00 | 0.00 | 0.00 | 0.00 | 0.00 | 0.00 | 0.00 | 0.00 | 0.00 | 0.00 |
| **Jingmen City** | 2596927 | 0.0000 | 0.0000 | 0.0309 | 0.0000 | 0.0000 | 0.0309 | 210.41 | 0.00 | 0.00 | 6.50 | 0.00 | 0.00 | 6.50 | 3.90 | 3.03 | 1.41 | 1.30 | 0.10 | 0.08 | 0.04 | 0.29 | 0.03 | 0.05 | 0.00 |
| Dongbao District | 356938 | 0.0000 | 0.0000 | 0.0000 | 0.0000 | 0.0000 | 0.0000 | 213.61 | 0.00 | 0.00 | 0.00 | 0.00 | 0.00 | 0.00 | 0.00 | 0.00 | 0.00 | 0.00 | 0.00 | 0.00 | 0.00 | 0.00 | 0.00 | 0.00 | 0.00 |
| Duodao District | 430532 | 0.0000 | 0.0000 | 0.0000 | 0.0000 | 0.0000 | 0.0000 | 785.33 | 0.00 | 0.00 | 0.00 | 0.00 | 0.00 | 0.00 | 0.00 | 0.00 | 0.00 | 0.00 | 0.00 | 0.00 | 0.00 | 0.00 | 0.00 | 0.00 | 0.00 |
| Shayang County | 395717 | 0.0000 | 0.0000 | 0.0000 | 0.0000 | 0.0000 | 0.0000 | 180.95 | 0.00 | 0.00 | 0.00 | 0.00 | 0.00 | 0.00 | 0.00 | 0.00 | 0.00 | 0.00 | 0.00 | 0.00 | 0.00 | 0.00 | 0.00 | 0.00 | 0.00 |
| Zhongxiang City | 868897 | 0.0000 | 0.0000 | 0.0309 | 0.0000 | 0.0000 | 0.0309 | 196.80 | 0.00 | 0.00 | 6.08 | 0.00 | 0.00 | 6.08 | 3.65 | 2.83 | 1.32 | 1.22 | 0.10 | 0.07 | 0.03 | 0.27 | 0.03 | 0.04 | 0.00 |
| Jingshan City | 544843 | 0.0000 | 0.0000 | 0.0000 | 0.0000 | 0.0000 | 0.0000 | 154.75 | 0.00 | 0.00 | 0.00 | 0.00 | 0.00 | 0.00 | 0.00 | 0.00 | 0.00 | 0.00 | 0.00 | 0.00 | 0.00 | 0.00 | 0.00 | 0.00 | 0.00 |
| **Xiaogan City** | 4270371 | 0.0000 | 0.0000 | 0.0000 | 0.0000 | 0.0000 | 0.0000 | 479.07 | 0.00 | 0.00 | 0.00 | 0.00 | 0.00 | 0.00 | 0.00 | 0.00 | 0.00 | 0.00 | 0.00 | 0.00 | 0.00 | 0.00 | 0.00 | 0.00 | 0.00 |
| Xiaonan District | 988479 | 0.0000 | 0.0000 | 0.0000 | 0.0000 | 0.0000 | 0.0000 | 961.27 | 0.00 | 0.00 | 0.00 | 0.00 | 0.00 | 0.00 | 0.00 | 0.00 | 0.00 | 0.00 | 0.00 | 0.00 | 0.00 | 0.00 | 0.00 | 0.00 | 0.00 |
| Xiaochang County | 483367 | 0.0000 | 0.0000 | 0.0000 | 0.0000 | 0.0000 | 0.0000 | 406.40 | 0.00 | 0.00 | 0.00 | 0.00 | 0.00 | 0.00 | 0.00 | 0.00 | 0.00 | 0.00 | 0.00 | 0.00 | 0.00 | 0.00 | 0.00 | 0.00 | 0.00 |
| Dawu County | 486153 | 0.0000 | 0.0000 | 0.0000 | 0.0000 | 0.0000 | 0.0000 | 245.31 | 0.00 | 0.00 | 0.00 | 0.00 | 0.00 | 0.00 | 0.00 | 0.00 | 0.00 | 0.00 | 0.00 | 0.00 | 0.00 | 0.00 | 0.00 | 0.00 | 0.00 |
| Yunmeng County | 434124 | 0.0000 | 0.0000 | 0.0000 | 0.0000 | 0.0000 | 0.0000 | 715.67 | 0.00 | 0.00 | 0.00 | 0.00 | 0.00 | 0.00 | 0.00 | 0.00 | 0.00 | 0.00 | 0.00 | 0.00 | 0.00 | 0.00 | 0.00 | 0.00 | 0.00 |
| Ying City | 476596 | 0.0000 | 0.0000 | 0.0000 | 0.0000 | 0.0000 | 0.0000 | 434.57 | 0.00 | 0.00 | 0.00 | 0.00 | 0.00 | 0.00 | 0.00 | 0.00 | 0.00 | 0.00 | 0.00 | 0.00 | 0.00 | 0.00 | 0.00 | 0.00 | 0.00 |
| Anlu City | 498356 | 0.0000 | 0.0000 | 0.0000 | 0.0000 | 0.0000 | 0.0000 | 367.87 | 0.00 | 0.00 | 0.00 | 0.00 | 0.00 | 0.00 | 0.00 | 0.00 | 0.00 | 0.00 | 0.00 | 0.00 | 0.00 | 0.00 | 0.00 | 0.00 | 0.00 |
| Hanchuan City | 903296 | 0.0000 | 0.0000 | 0.0000 | 0.0000 | 0.0000 | 0.0000 | 545.37 | 0.00 | 0.00 | 0.00 | 0.00 | 0.00 | 0.00 | 0.00 | 0.00 | 0.00 | 0.00 | 0.00 | 0.00 | 0.00 | 0.00 | 0.00 | 0.00 | 0.00 |
| **Jingzhou City** | 5231180 | 0.0000 | 0.0000 | 0.0000 | 0.0000 | 0.0000 | 0.0000 | 480.46 | 0.00 | 0.00 | 0.00 | 0.00 | 0.00 | 0.00 | 0.00 | 0.00 | 0.00 | 0.00 | 0.00 | 0.00 | 0.00 | 0.00 | 0.00 | 0.00 | 0.00 |
| Shashi City | 694977 | 0.0000 | 0.0000 | 0.0000 | 0.0000 | 0.0000 | 0.0000 | 1344.35 | 0.00 | 0.00 | 0.00 | 0.00 | 0.00 | 0.00 | 0.00 | 0.00 | 0.00 | 0.00 | 0.00 | 0.00 | 0.00 | 0.00 | 0.00 | 0.00 | 0.00 |
| Jinzhou District | 563398 | 0.0000 | 0.0000 | 0.0000 | 0.0000 | 0.0000 | 0.0000 | 541.10 | 0.00 | 0.00 | 0.00 | 0.00 | 0.00 | 0.00 | 0.00 | 0.00 | 0.00 | 0.00 | 0.00 | 0.00 | 0.00 | 0.00 | 0.00 | 0.00 | 0.00 |
| Gong'an County | 747134 | 0.0000 | 0.0000 | 0.0000 | 0.0000 | 0.0000 | 0.0000 | 330.66 | 0.00 | 0.00 | 0.00 | 0.00 | 0.00 | 0.00 | 0.00 | 0.00 | 0.00 | 0.00 | 0.00 | 0.00 | 0.00 | 0.00 | 0.00 | 0.00 | 0.00 |
| Jianli County | 1120822 | 0.0000 | 0.0000 | 0.0000 | 0.0000 | 0.0000 | 0.0000 | #DIV/0! | #DIV/0! | #DIV/0! | #DIV/0! | #DIV/0! | #DIV/0! | #DIV/0! | #DIV/0! | #DIV/0! | #DIV/0! | #DIV/0! | #DIV/0! | #DIV/0! | #DIV/0! | #DIV/0! | #DIV/0! | #DIV/0! | #DIV/0! |
| Jiangling County | 278192 | 0.0000 | 0.0000 | 0.0000 | 0.0000 | 0.0000 | 0.0000 | 265.78 | 0.00 | 0.00 | 0.00 | 0.00 | 0.00 | 0.00 | 0.00 | 0.00 | 0.00 | 0.00 | 0.00 | 0.00 | 0.00 | 0.00 | 0.00 | 0.00 | 0.00 |
| Shishou City | 473707 | 0.0000 | 0.0000 | 0.0000 | 0.0000 | 0.0000 | 0.0000 | 335.91 | 0.00 | 0.00 | 0.00 | 0.00 | 0.00 | 0.00 | 0.00 | 0.00 | 0.00 | 0.00 | 0.00 | 0.00 | 0.00 | 0.00 | 0.00 | 0.00 | 0.00 |
| Honghu City | 698188 | 0.0000 | 0.0000 | 0.0000 | 0.0000 | 0.0000 | 0.0000 | 286.44 | 0.00 | 0.00 | 0.00 | 0.00 | 0.00 | 0.00 | 0.00 | 0.00 | 0.00 | 0.00 | 0.00 | 0.00 | 0.00 | 0.00 | 0.00 | 0.00 | 0.00 |
| Songzi City | 654762 | 0.0000 | 0.0000 | 0.0000 | 0.0000 | 0.0000 | 0.0000 | 300.93 | 0.00 | 0.00 | 0.00 | 0.00 | 0.00 | 0.00 | 0.00 | 0.00 | 0.00 | 0.00 | 0.00 | 0.00 | 0.00 | 0.00 | 0.00 | 0.00 | 0.00 |
| **Huanggang City** | 5882719 | 0.0000 | 0.0000 | 0.0000 | 0.0000 | 0.0000 | 0.0000 | 337.18 | 0.00 | 0.00 | 0.00 | 0.00 | 0.00 | 0.00 | 0.00 | 0.00 | 0.00 | 0.00 | 0.00 | 0.00 | 0.00 | 0.00 | 0.00 | 0.00 | 0.00 |
| Huangzhou District | 456862 | 0.0000 | 0.0000 | 0.0000 | 0.0000 | 0.0000 | 0.0000 | 1261.56 | 0.00 | 0.00 | 0.00 | 0.00 | 0.00 | 0.00 | 0.00 | 0.00 | 0.00 | 0.00 | 0.00 | 0.00 | 0.00 | 0.00 | 0.00 | 0.00 | 0.00 |
| Tuanfeng County | 266218 | 0.0000 | 0.0000 | 0.0000 | 0.0000 | 0.0000 | 0.0000 | 322.97 | 0.00 | 0.00 | 0.00 | 0.00 | 0.00 | 0.00 | 0.00 | 0.00 | 0.00 | 0.00 | 0.00 | 0.00 | 0.00 | 0.00 | 0.00 | 0.00 | 0.00 |
| Hong'an County | 510189 | 0.0000 | 0.0000 | 0.0000 | 0.0000 | 0.0000 | 0.0000 | 284.62 | 0.00 | 0.00 | 0.00 | 0.00 | 0.00 | 0.00 | 0.00 | 0.00 | 0.00 | 0.00 | 0.00 | 0.00 | 0.00 | 0.00 | 0.00 | 0.00 | 0.00 |
| Luotian County | 473195 | 0.0000 | 0.0000 | 0.0000 | 0.0000 | 0.0000 | 0.0000 | 221.46 | 0.00 | 0.00 | 0.00 | 0.00 | 0.00 | 0.00 | 0.00 | 0.00 | 0.00 | 0.00 | 0.00 | 0.00 | 0.00 | 0.00 | 0.00 | 0.00 | 0.00 |
| Yingshan County | 310180 | 0.0000 | 0.0000 | 0.0000 | 0.0000 | 0.0000 | 0.0000 | 214.97 | 0.00 | 0.00 | 0.00 | 0.00 | 0.00 | 0.00 | 0.00 | 0.00 | 0.00 | 0.00 | 0.00 | 0.00 | 0.00 | 0.00 | 0.00 | 0.00 | 0.00 |
| Xishui County | 716273 | 0.0000 | 0.0000 | 0.0000 | 0.0000 | 0.0000 | 0.0000 | 365.63 | 0.00 | 0.00 | 0.00 | 0.00 | 0.00 | 0.00 | 0.00 | 0.00 | 0.00 | 0.00 | 0.00 | 0.00 | 0.00 | 0.00 | 0.00 | 0.00 | 0.00 |
| Qichun County | 792101 | 0.0000 | 0.0000 | 0.0000 | 0.0000 | 0.0000 | 0.0000 | 331.28 | 0.00 | 0.00 | 0.00 | 0.00 | 0.00 | 0.00 | 0.00 | 0.00 | 0.00 | 0.00 | 0.00 | 0.00 | 0.00 | 0.00 | 0.00 | 0.00 | 0.00 |
| Huangmei County | 787783 | 0.0000 | 0.0000 | 0.0000 | 0.0000 | 0.0000 | 0.0000 | 461.64 | 0.00 | 0.00 | 0.00 | 0.00 | 0.00 | 0.00 | 0.00 | 0.00 | 0.00 | 0.00 | 0.00 | 0.00 | 0.00 | 0.00 | 0.00 | 0.00 | 0.00 |
| Macheng City | 893654 | 0.0000 | 0.0000 | 0.0000 | 0.0000 | 0.0000 | 0.0000 | 248.53 | 0.00 | 0.00 | 0.00 | 0.00 | 0.00 | 0.00 | 0.00 | 0.00 | 0.00 | 0.00 | 0.00 | 0.00 | 0.00 | 0.00 | 0.00 | 0.00 | 0.00 |
| Wuxue City | 676264 | 0.0000 | 0.0000 | 0.0000 | 0.0000 | 0.0000 | 0.0000 | 547.18 | 0.00 | 0.00 | 0.00 | 0.00 | 0.00 | 0.00 | 0.00 | 0.00 | 0.00 | 0.00 | 0.00 | 0.00 | 0.00 | 0.00 | 0.00 | 0.00 | 0.00 |
| **Xianning City** | 2658316 | 0.0000 | 0.0000 | 0.0000 | 0.0000 | 0.0000 | 0.0000 | 272.40 | 0.00 | 0.00 | 0.00 | 0.00 | 0.00 | 0.00 | 0.00 | 0.00 | 0.00 | 0.00 | 0.00 | 0.00 | 0.00 | 0.00 | 0.00 | 0.00 | 0.00 |
| Xian'an District | 657590 | 0.0000 | 0.0000 | 0.0000 | 0.0000 | 0.0000 | 0.0000 | 437.58 | 0.00 | 0.00 | 0.00 | 0.00 | 0.00 | 0.00 | 0.00 | 0.00 | 0.00 | 0.00 | 0.00 | 0.00 | 0.00 | 0.00 | 0.00 | 0.00 | 0.00 |
| Jiayu County | 285642 | 0.0000 | 0.0000 | 0.0000 | 0.0000 | 0.0000 | 0.0000 | 277.83 | 0.00 | 0.00 | 0.00 | 0.00 | 0.00 | 0.00 | 0.00 | 0.00 | 0.00 | 0.00 | 0.00 | 0.00 | 0.00 | 0.00 | 0.00 | 0.00 | 0.00 |
| Tongcheng County | 426288 | 0.0000 | 0.0000 | 0.0000 | 0.0000 | 0.0000 | 0.0000 | 379.94 | 0.00 | 0.00 | 0.00 | 0.00 | 0.00 | 0.00 | 0.00 | 0.00 | 0.00 | 0.00 | 0.00 | 0.00 | 0.00 | 0.00 | 0.00 | 0.00 | 0.00 |
| Chongyang County | 427130 | 0.0000 | 0.0000 | 0.0000 | 0.0000 | 0.0000 | 0.0000 | 216.26 | 0.00 | 0.00 | 0.00 | 0.00 | 0.00 | 0.00 | 0.00 | 0.00 | 0.00 | 0.00 | 0.00 | 0.00 | 0.00 | 0.00 | 0.00 | 0.00 | 0.00 |
| Tongshan County | 391311 | 0.0000 | 0.0000 | 0.0000 | 0.0000 | 0.0000 | 0.0000 | 161.51 | 0.00 | 0.00 | 0.00 | 0.00 | 0.00 | 0.00 | 0.00 | 0.00 | 0.00 | 0.00 | 0.00 | 0.00 | 0.00 | 0.00 | 0.00 | 0.00 | 0.00 |
| Chibi City | 470355 | 0.0000 | 0.0000 | 0.0000 | 0.0000 | 0.0000 | 0.0000 | 275.37 | 0.00 | 0.00 | 0.00 | 0.00 | 0.00 | 0.00 | 0.00 | 0.00 | 0.00 | 0.00 | 0.00 | 0.00 | 0.00 | 0.00 | 0.00 | 0.00 | 0.00 |
| **Suizhou City** | 2047923 | 0.0000 | 0.0000 | 0.0000 | 0.0000 | 0.0000 | 0.0000 | 213.04 | 0.00 | 0.00 | 0.00 | 0.00 | 0.00 | 0.00 | 0.00 | 0.00 | 0.00 | 0.00 | 0.00 | 0.00 | 0.00 | 0.00 | 0.00 | 0.00 | 0.00 |
| Zengdu District | 699475 | 0.0000 | 0.0000 | 0.0000 | 0.0000 | 0.0000 | 0.0000 | 488.05 | 0.00 | 0.00 | 0.00 | 0.00 | 0.00 | 0.00 | 0.00 | 0.00 | 0.00 | 0.00 | 0.00 | 0.00 | 0.00 | 0.00 | 0.00 | 0.00 | 0.00 |
| Sui County | 637548 | 0.0000 | 0.0000 | 0.0000 | 0.0000 | 0.0000 | 0.0000 | 115.17 | 0.00 | 0.00 | 0.00 | 0.00 | 0.00 | 0.00 | 0.00 | 0.00 | 0.00 | 0.00 | 0.00 | 0.00 | 0.00 | 0.00 | 0.00 | 0.00 | 0.00 |
| Guangshui City | 710900 | 0.0000 | 0.0000 | 0.0000 | 0.0000 | 0.0000 | 0.0000 | 268.89 | 0.00 | 0.00 | 0.00 | 0.00 | 0.00 | 0.00 | 0.00 | 0.00 | 0.00 | 0.00 | 0.00 | 0.00 | 0.00 | 0.00 | 0.00 | 0.00 | 0.00 |
| **Enshi Tujia and Miao Autonomous Prefecture** | 3456136 | 0.0000 | 0.0000 | 0.0000 | 0.0000 | 0.0000 | 0.0000 | 143.63 | 0.00 | 0.00 | 0.00 | 0.00 | 0.00 | 0.00 | 0.00 | 0.00 | 0.00 | 0.00 | 0.00 | 0.00 | 0.00 | 0.00 | 0.00 | 0.00 | 0.00 |
| Enshi City | 836781 | 0.0000 | 0.0000 | 0.0000 | 0.0000 | 0.0000 | 0.0000 | 211.23 | 0.00 | 0.00 | 0.00 | 0.00 | 0.00 | 0.00 | 0.00 | 0.00 | 0.00 | 0.00 | 0.00 | 0.00 | 0.00 | 0.00 | 0.00 | 0.00 | 0.00 |
| Lichuan City | 750670 | 0.0000 | 0.0000 | 0.0000 | 0.0000 | 0.0000 | 0.0000 | 162.97 | 0.00 | 0.00 | 0.00 | 0.00 | 0.00 | 0.00 | 0.00 | 0.00 | 0.00 | 0.00 | 0.00 | 0.00 | 0.00 | 0.00 | 0.00 | 0.00 | 0.00 |
| Jianshi County | 411622 | 0.0000 | 0.0000 | 0.0000 | 0.0000 | 0.0000 | 0.0000 | 154.22 | 0.00 | 0.00 | 0.00 | 0.00 | 0.00 | 0.00 | 0.00 | 0.00 | 0.00 | 0.00 | 0.00 | 0.00 | 0.00 | 0.00 | 0.00 | 0.00 | 0.00 |
| Badong County | 395376 | 0.0000 | 0.0000 | 0.0000 | 0.0000 | 0.0000 | 0.0000 | 117.93 | 0.00 | 0.00 | 0.00 | 0.00 | 0.00 | 0.00 | 0.00 | 0.00 | 0.00 | 0.00 | 0.00 | 0.00 | 0.00 | 0.00 | 0.00 | 0.00 | 0.00 |
| Xuan'en County | 284955 | 0.0000 | 0.0000 | 0.0000 | 0.0000 | 0.0000 | 0.0000 | 104.11 | 0.00 | 0.00 | 0.00 | 0.00 | 0.00 | 0.00 | 0.00 | 0.00 | 0.00 | 0.00 | 0.00 | 0.00 | 0.00 | 0.00 | 0.00 | 0.00 | 0.00 |
| Xianfeng County | 318827 | 0.0000 | 0.0000 | 0.0000 | 0.0000 | 0.0000 | 0.0000 | 126.17 | 0.00 | 0.00 | 0.00 | 0.00 | 0.00 | 0.00 | 0.00 | 0.00 | 0.00 | 0.00 | 0.00 | 0.00 | 0.00 | 0.00 | 0.00 | 0.00 | 0.00 |
| Laifeng County | 283255 | 0.0000 | 0.0000 | 0.0000 | 0.0000 | 0.0000 | 0.0000 | 211.24 | 0.00 | 0.00 | 0.00 | 0.00 | 0.00 | 0.00 | 0.00 | 0.00 | 0.00 | 0.00 | 0.00 | 0.00 | 0.00 | 0.00 | 0.00 | 0.00 | 0.00 |
| Hefeng County | 174650 | 0.0000 | 0.0000 | 0.0000 | 0.0000 | 0.0000 | 0.0000 | 60.90 | 0.00 | 0.00 | 0.00 | 0.00 | 0.00 | 0.00 | 0.00 | 0.00 | 0.00 | 0.00 | 0.00 | 0.00 | 0.00 | 0.00 | 0.00 | 0.00 | 0.00 |
| **Administrative divisions at the county level directly under the provincial government** | 3380473 | 0.0000 | 0.0000 | 0.0000 | 0.0000 | 0.0000 | 0.0000 | 326.13 | 0.00 | 0.00 | 0.00 | 0.00 | 0.00 | 0.00 | 0.00 | 0.00 | 0.00 | 0.00 | 0.00 | 0.00 | 0.00 | 0.00 | 0.00 | 0.00 | 0.00 |
| Xiantao City | 1268715 | 0.0000 | 0.0000 | 0.0000 | 0.0000 | 0.0000 | 0.0000 | 503.52 | 0.00 | 0.00 | 0.00 | 0.00 | 0.00 | 0.00 | 0.00 | 0.00 | 0.00 | 0.00 | 0.00 | 0.00 | 0.00 | 0.00 | 0.00 | 0.00 | 0.00 |
| Qianjiang City | 886547 | 0.0000 | 0.0000 | 0.0000 | 0.0000 | 0.0000 | 0.0000 | 444.03 | 0.00 | 0.00 | 0.00 | 0.00 | 0.00 | 0.00 | 0.00 | 0.00 | 0.00 | 0.00 | 0.00 | 0.00 | 0.00 | 0.00 | 0.00 | 0.00 | 0.00 |
| Tianmen City | 1158640 | 0.0000 | 0.0000 | 0.0000 | 0.0000 | 0.0000 | 0.0000 | 443.18 | 0.00 | 0.00 | 0.00 | 0.00 | 0.00 | 0.00 | 0.00 | 0.00 | 0.00 | 0.00 | 0.00 | 0.00 | 0.00 | 0.00 | 0.00 | 0.00 | 0.00 |
| Shennongjialin District | 66571 | 0.0000 | 0.0000 | 0.0000 | 0.0000 | 0.0000 | 0.0000 | 20.58 | 0.00 | 0.00 | 0.00 | 0.00 | 0.00 | 0.00 | 0.00 | 0.00 | 0.00 | 0.00 | 0.00 | 0.00 | 0.00 | 0.00 | 0.00 | 0.00 | 0.00 |
| **Hunan Province** | 66444864 | 40.7690 | 6.1883 | 2.6767 | 0.8527 | 0.1205 | 50.5871 | 315.60 | 12866.76 | 1953.05 | 844.76 | 269.10 | 38.02 | 15965.37 | 3272.28 | 2537.33 | 1181.95 | 1090.65 | 86.72 | 64.79 | 30.76 | 246.08 | 24.54 | 38.29 | 0.98 |
| **Changsha city** | 10047914 | 3.3547 | 1.4233 | 0.2200 | 0.5260 | 0.0138 | 5.5178 | 850.28 | 2852.41 | 1210.23 | 187.06 | 447.25 | 11.73 | 4691.68 | 1271.53 | 985.94 | 459.28 | 423.80 | 33.70 | 25.18 | 11.95 | 95.62 | 9.54 | 14.88 | 0.38 |
| Furong District | 642010 | 0.0000 | 0.0000 | 0.0000 | 0.0000 | 0.0000 | 0.0000 | 15060.05 | 0.00 | 0.00 | 0.00 | 0.00 | 0.00 | 0.00 | 0.00 | 0.00 | 0.00 | 0.00 | 0.00 | 0.00 | 0.00 | 0.00 | 0.00 | 0.00 | 0.00 |
| Tianxin District | 836157 | 0.0000 | 0.0000 | 0.0000 | 0.0000 | 0.0000 | 0.0000 | 6109.58 | 0.00 | 0.00 | 0.00 | 0.00 | 0.00 | 0.00 | 0.00 | 0.00 | 0.00 | 0.00 | 0.00 | 0.00 | 0.00 | 0.00 | 0.00 | 0.00 | 0.00 |
| Yuelu District | 1526641 | 0.0000 | 0.0000 | 0.0000 | 0.0000 | 0.0000 | 0.0000 | 2826.54 | 0.00 | 0.00 | 0.00 | 0.00 | 0.00 | 0.00 | 0.00 | 0.00 | 0.00 | 0.00 | 0.00 | 0.00 | 0.00 | 0.00 | 0.00 | 0.00 | 0.00 |
| Kaifu District | 820790 | 0.0000 | 0.0000 | 0.0000 | 0.0000 | 0.0000 | 0.0000 | 4340.05 | 0.00 | 0.00 | 0.00 | 0.00 | 0.00 | 0.00 | 0.00 | 0.00 | 0.00 | 0.00 | 0.00 | 0.00 | 0.00 | 0.00 | 0.00 | 0.00 | 0.00 |
| Yuhua District | 1264895 | 0.0000 | 0.0000 | 0.0000 | 0.0000 | 0.0000 | 0.0000 | 4314.25 | 0.00 | 0.00 | 0.00 | 0.00 | 0.00 | 0.00 | 0.00 | 0.00 | 0.00 | 0.00 | 0.00 | 0.00 | 0.00 | 0.00 | 0.00 | 0.00 | 0.00 |
| Wangcheng District | 890214 | 0.0000 | 0.0000 | 0.0000 | 0.0000 | 0.0000 | 0.0000 | 936.52 | 0.00 | 0.00 | 0.00 | 0.00 | 0.00 | 0.00 | 0.00 | 0.00 | 0.00 | 0.00 | 0.00 | 0.00 | 0.00 | 0.00 | 0.00 | 0.00 | 0.00 |
| Changsha County | 1374491 | 3.3413 | 1.4033 | 0.2000 | 0.5260 | 0.0138 | 5.4845 | 782.34 | 2614.05 | 1097.88 | 156.47 | 411.51 | 10.80 | 4290.71 | 1154.28 | 895.03 | 416.93 | 384.72 | 30.59 | 22.85 | 10.85 | 86.80 | 8.66 | 13.51 | 0.35 |
| Liuyang City | 1429384 | 0.0133 | 0.0200 | 0.0200 | 0.0000 | 0.0000 | 0.0333 | 286.03 | 3.81 | 5.72 | 5.72 | 0.00 | 0.00 | 9.53 | 5.72 | 4.44 | 2.07 | 1.91 | 0.15 | 0.11 | 0.05 | 0.43 | 0.04 | 0.07 | 0.00 |
| Ningxiang City | 1263332 | 0.0000 | 0.0000 | 0.0000 | 0.0000 | 0.0000 | 0.0000 | 434.06 | 0.00 | 0.00 | 0.00 | 0.00 | 0.00 | 0.00 | 0.00 | 0.00 | 0.00 | 0.00 | 0.00 | 0.00 | 0.00 | 0.00 | 0.00 | 0.00 | 0.00 |
| **Zhuzhou City** | 3902738 | 0.0067 | 0.0000 | 0.2013 | 0.0000 | 0.0000 | 0.2080 | 347.02 | 2.31 | 0.00 | 69.87 | 0.00 | 0.00 | 72.18 | 42.27 | 32.77 | 15.27 | 14.09 | 1.12 | 0.84 | 0.40 | 3.18 | 0.32 | 0.49 | 0.01 |
| Hetang District | 348894 | 0.0000 | 0.0000 | 0.0000 | 0.0000 | 0.0000 | 0.0000 | 2322.55 | 0.00 | 0.00 | 0.00 | 0.00 | 0.00 | 0.00 | 0.00 | 0.00 | 0.00 | 0.00 | 0.00 | 0.00 | 0.00 | 0.00 | 0.00 | 0.00 | 0.00 |
| Lusong District | 307012 | 0.0000 | 0.0000 | 0.0000 | 0.0000 | 0.0000 | 0.0000 | 1395.07 | 0.00 | 0.00 | 0.00 | 0.00 | 0.00 | 0.00 | 0.00 | 0.00 | 0.00 | 0.00 | 0.00 | 0.00 | 0.00 | 0.00 | 0.00 | 0.00 | 0.00 |
| Shifeng District | 339452 | 0.0000 | 0.0000 | 0.2013 | 0.0000 | 0.0000 | 0.2013 | 2050.20 | 0.00 | 0.00 | 412.77 | 0.00 | 0.00 | 412.77 | 247.66 | 192.04 | 89.46 | 82.55 | 6.56 | 4.90 | 2.33 | 18.62 | 1.86 | 2.90 | 0.07 |
| Tengen District | 478309 | 0.0000 | 0.0000 | 0.0000 | 0.0000 | 0.0000 | 0.0000 | 1469.78 | 0.00 | 0.00 | 0.00 | 0.00 | 0.00 | 0.00 | 0.00 | 0.00 | 0.00 | 0.00 | 0.00 | 0.00 | 0.00 | 0.00 | 0.00 | 0.00 | 0.00 |
| Lukou District | 260534 | 0.0000 | 0.0000 | 0.0000 | 0.0000 | 0.0000 | 0.0000 | 247.02 | 0.00 | 0.00 | 0.00 | 0.00 | 0.00 | 0.00 | 0.00 | 0.00 | 0.00 | 0.00 | 0.00 | 0.00 | 0.00 | 0.00 | 0.00 | 0.00 | 0.00 |
| You County | 630427 | 0.0000 | 0.0000 | 0.0000 | 0.0000 | 0.0000 | 0.0000 | 238.08 | 0.00 | 0.00 | 0.00 | 0.00 | 0.00 | 0.00 | 0.00 | 0.00 | 0.00 | 0.00 | 0.00 | 0.00 | 0.00 | 0.00 | 0.00 | 0.00 | 0.00 |
| Chaling County | 491849 | 0.0067 | 0.0000 | 0.0000 | 0.0000 | 0.0000 | 0.0067 | 197.24 | 1.31 | 0.00 | 0.00 | 0.00 | 0.00 | 1.31 | 0.20 | 0.15 | 0.07 | 0.07 | 0.01 | 0.00 | 0.00 | 0.01 | 0.00 | 0.00 | 0.00 |
| Yanling County | 160274 | 0.0000 | 0.0000 | 0.0000 | 0.0000 | 0.0000 | 0.0000 | 78.94 | 0.00 | 0.00 | 0.00 | 0.00 | 0.00 | 0.00 | 0.00 | 0.00 | 0.00 | 0.00 | 0.00 | 0.00 | 0.00 | 0.00 | 0.00 | 0.00 | 0.00 |
| Liling City | 885987 | 0.0000 | 0.0000 | 0.0000 | 0.0000 | 0.0000 | 0.0000 | 410.48 | 0.00 | 0.00 | 0.00 | 0.00 | 0.00 | 0.00 | 0.00 | 0.00 | 0.00 | 0.00 | 0.00 | 0.00 | 0.00 | 0.00 | 0.00 | 0.00 | 0.00 |
| **Xiangtan City** | 2726181 | 2.0000 | 0.0000 | 0.0000 | 0.0000 | 0.0000 | 2.0000 | 544.59 | 1089.19 | 0.00 | 0.00 | 0.00 | 0.00 | 1089.19 | 163.38 | 126.68 | 59.01 | 54.45 | 4.33 | 3.23 | 1.54 | 12.29 | 1.23 | 1.91 | 0.05 |
| Yuhu District | 616130 | 1.1333 | 0.0000 | 0.0000 | 0.0000 | 0.0000 | 1.1333 | 1385.40 | 1570.12 | 0.00 | 0.00 | 0.00 | 0.00 | 1570.12 | 235.52 | 182.62 | 85.07 | 78.50 | 6.24 | 4.66 | 2.21 | 17.71 | 1.77 | 2.76 | 0.07 |
| Yuetang District | 483762 | 0.8667 | 0.0000 | 0.0000 | 0.0000 | 0.0000 | 0.8667 | 2339.16 | 2027.27 | 0.00 | 0.00 | 0.00 | 0.00 | 2027.27 | 304.09 | 235.79 | 109.84 | 101.35 | 8.06 | 6.02 | 2.86 | 22.87 | 2.28 | 3.56 | 0.09 |
| Xiangtan County | 792829 | 0.0000 | 0.0000 | 0.0000 | 0.0000 | 0.0000 | 0.0000 | 370.69 | 0.00 | 0.00 | 0.00 | 0.00 | 0.00 | 0.00 | 0.00 | 0.00 | 0.00 | 0.00 | 0.00 | 0.00 | 0.00 | 0.00 | 0.00 | 0.00 | 0.00 |
| Xiangxiang City | 730103 | 0.0000 | 0.0000 | 0.0000 | 0.0000 | 0.0000 | 0.0000 | 363.92 | 0.00 | 0.00 | 0.00 | 0.00 | 0.00 | 0.00 | 0.00 | 0.00 | 0.00 | 0.00 | 0.00 | 0.00 | 0.00 | 0.00 | 0.00 | 0.00 | 0.00 |
| Shaoshan City | 103357 | 0.0000 | 0.0000 | 0.0000 | 0.0000 | 0.0000 | 0.0000 | 493.68 | 0.00 | 0.00 | 0.00 | 0.00 | 0.00 | 0.00 | 0.00 | 0.00 | 0.00 | 0.00 | 0.00 | 0.00 | 0.00 | 0.00 | 0.00 | 0.00 | 0.00 |
| **Hengyang city** | 6645243 | 0.0000 | 0.6603 | 0.0000 | 0.0000 | 0.0000 | 0.6603 | 434.40 | 0.00 | 286.85 | 0.00 | 0.00 | 0.00 | 286.85 | 86.05 | 66.73 | 31.08 | 28.68 | 2.28 | 1.70 | 0.81 | 6.47 | 0.65 | 1.01 | 0.03 |
| Zhuhui District | 337337 | 0.0000 | 0.2047 | 0.0000 | 0.0000 | 0.0000 | 0.2047 | 1538.53 | 0.00 | 314.88 | 0.00 | 0.00 | 0.00 | 314.88 | 94.47 | 73.25 | 34.12 | 31.49 | 2.50 | 1.87 | 0.89 | 7.10 | 0.71 | 1.11 | 0.03 |
| Yanfeng District | 247791 | 0.0000 | 0.1226 | 0.0000 | 0.0000 | 0.0000 | 0.1226 | 2999.16 | 0.00 | 367.62 | 0.00 | 0.00 | 0.00 | 367.62 | 110.29 | 85.52 | 39.84 | 36.76 | 2.92 | 2.18 | 1.04 | 8.29 | 0.83 | 1.29 | 0.03 |
| Shigu District | 227515 | 0.0000 | 0.2787 | 0.0000 | 0.0000 | 0.0000 | 0.2787 | 2172.40 | 0.00 | 605.37 | 0.00 | 0.00 | 0.00 | 605.37 | 181.61 | 140.82 | 65.60 | 60.53 | 4.81 | 3.60 | 1.71 | 13.66 | 1.36 | 2.12 | 0.05 |
| Zhengxiang District | 478072 | 0.0000 | 0.0544 | 0.0000 | 0.0000 | 0.0000 | 0.0544 | 4307.34 | 0.00 | 234.43 | 0.00 | 0.00 | 0.00 | 234.43 | 70.33 | 54.53 | 25.40 | 23.44 | 1.86 | 1.39 | 0.66 | 5.29 | 0.53 | 0.82 | 0.02 |
| Nanyue District | 70370 | 0.0000 | 0.0000 | 0.0000 | 0.0000 | 0.0000 | 0.0000 | 390.23 | 0.00 | 0.00 | 0.00 | 0.00 | 0.00 | 0.00 | 0.00 | 0.00 | 0.00 | 0.00 | 0.00 | 0.00 | 0.00 | 0.00 | 0.00 | 0.00 | 0.00 |
| Hengyang County | 888433 | 0.0000 | 0.0000 | 0.0000 | 0.0000 | 0.0000 | 0.0000 | 346.95 | 0.00 | 0.00 | 0.00 | 0.00 | 0.00 | 0.00 | 0.00 | 0.00 | 0.00 | 0.00 | 0.00 | 0.00 | 0.00 | 0.00 | 0.00 | 0.00 | 0.00 |
| Hengnan County | 796327 | 0.0000 | 0.0000 | 0.0000 | 0.0000 | 0.0000 | 0.0000 | 304.65 | 0.00 | 0.00 | 0.00 | 0.00 | 0.00 | 0.00 | 0.00 | 0.00 | 0.00 | 0.00 | 0.00 | 0.00 | 0.00 | 0.00 | 0.00 | 0.00 | 0.00 |
| Hengshan County | 335704 | 0.0000 | 0.0000 | 0.0000 | 0.0000 | 0.0000 | 0.0000 | 359.64 | 0.00 | 0.00 | 0.00 | 0.00 | 0.00 | 0.00 | 0.00 | 0.00 | 0.00 | 0.00 | 0.00 | 0.00 | 0.00 | 0.00 | 0.00 | 0.00 | 0.00 |
| Hengdong County | 565423 | 0.0000 | 0.0000 | 0.0000 | 0.0000 | 0.0000 | 0.0000 | 293.31 | 0.00 | 0.00 | 0.00 | 0.00 | 0.00 | 0.00 | 0.00 | 0.00 | 0.00 | 0.00 | 0.00 | 0.00 | 0.00 | 0.00 | 0.00 | 0.00 | 0.00 |
| Qidong County | 766920 | 0.0000 | 0.0000 | 0.0000 | 0.0000 | 0.0000 | 0.0000 | 410.80 | 0.00 | 0.00 | 0.00 | 0.00 | 0.00 | 0.00 | 0.00 | 0.00 | 0.00 | 0.00 | 0.00 | 0.00 | 0.00 | 0.00 | 0.00 | 0.00 | 0.00 |
| Leiyang City | 1140675 | 0.0000 | 0.0000 | 0.0000 | 0.0000 | 0.0000 | 0.0000 | 430.52 | 0.00 | 0.00 | 0.00 | 0.00 | 0.00 | 0.00 | 0.00 | 0.00 | 0.00 | 0.00 | 0.00 | 0.00 | 0.00 | 0.00 | 0.00 | 0.00 | 0.00 |
| Changning City | 790676 | 0.0000 | 0.0000 | 0.0000 | 0.0000 | 0.0000 | 0.0000 | 386.15 | 0.00 | 0.00 | 0.00 | 0.00 | 0.00 | 0.00 | 0.00 | 0.00 | 0.00 | 0.00 | 0.00 | 0.00 | 0.00 | 0.00 | 0.00 | 0.00 | 0.00 |
| **Shaoyang City** | 6563520 | 0.0000 | 0.0000 | 0.0000 | 0.0000 | 0.0000 | 0.0000 | 315.24 | 0.00 | 0.00 | 0.00 | 0.00 | 0.00 | 0.00 | 0.00 | 0.00 | 0.00 | 0.00 | 0.00 | 0.00 | 0.00 | 0.00 | 0.00 | 0.00 | 0.00 |
| Shuangqing District | 317283 | 0.0000 | 0.0000 | 0.0000 | 0.0000 | 0.0000 | 0.0000 | 2340.54 | 0.00 | 0.00 | 0.00 | 0.00 | 0.00 | 0.00 | 0.00 | 0.00 | 0.00 | 0.00 | 0.00 | 0.00 | 0.00 | 0.00 | 0.00 | 0.00 | 0.00 |
| Daxiang District | 362289 | 0.0000 | 0.0000 | 0.0000 | 0.0000 | 0.0000 | 0.0000 | 1687.11 | 0.00 | 0.00 | 0.00 | 0.00 | 0.00 | 0.00 | 0.00 | 0.00 | 0.00 | 0.00 | 0.00 | 0.00 | 0.00 | 0.00 | 0.00 | 0.00 | 0.00 |
| Beita District | 122658 | 0.0000 | 0.0000 | 0.0000 | 0.0000 | 0.0000 | 0.0000 | 1438.30 | 0.00 | 0.00 | 0.00 | 0.00 | 0.00 | 0.00 | 0.00 | 0.00 | 0.00 | 0.00 | 0.00 | 0.00 | 0.00 | 0.00 | 0.00 | 0.00 | 0.00 |
| Xinshao County | 612943 | 0.0000 | 0.0000 | 0.0000 | 0.0000 | 0.0000 | 0.0000 | 347.32 | 0.00 | 0.00 | 0.00 | 0.00 | 0.00 | 0.00 | 0.00 | 0.00 | 0.00 | 0.00 | 0.00 | 0.00 | 0.00 | 0.00 | 0.00 | 0.00 | 0.00 |
| Shaoyang County | 752125 | 0.0000 | 0.0000 | 0.0000 | 0.0000 | 0.0000 | 0.0000 | 376.16 | 0.00 | 0.00 | 0.00 | 0.00 | 0.00 | 0.00 | 0.00 | 0.00 | 0.00 | 0.00 | 0.00 | 0.00 | 0.00 | 0.00 | 0.00 | 0.00 | 0.00 |
| Longhui County | 1009778 | 0.0000 | 0.0000 | 0.0000 | 0.0000 | 0.0000 | 0.0000 | 352.55 | 0.00 | 0.00 | 0.00 | 0.00 | 0.00 | 0.00 | 0.00 | 0.00 | 0.00 | 0.00 | 0.00 | 0.00 | 0.00 | 0.00 | 0.00 | 0.00 | 0.00 |
| Dongkou County | 675495 | 0.0000 | 0.0000 | 0.0000 | 0.0000 | 0.0000 | 0.0000 | 310.82 | 0.00 | 0.00 | 0.00 | 0.00 | 0.00 | 0.00 | 0.00 | 0.00 | 0.00 | 0.00 | 0.00 | 0.00 | 0.00 | 0.00 | 0.00 | 0.00 | 0.00 |
| Suining County | 290664 | 0.0000 | 0.0000 | 0.0000 | 0.0000 | 0.0000 | 0.0000 | 99.54 | 0.00 | 0.00 | 0.00 | 0.00 | 0.00 | 0.00 | 0.00 | 0.00 | 0.00 | 0.00 | 0.00 | 0.00 | 0.00 | 0.00 | 0.00 | 0.00 | 0.00 |
| Xinning County | 513777 | 0.0000 | 0.0000 | 0.0000 | 0.0000 | 0.0000 | 0.0000 | 186.40 | 0.00 | 0.00 | 0.00 | 0.00 | 0.00 | 0.00 | 0.00 | 0.00 | 0.00 | 0.00 | 0.00 | 0.00 | 0.00 | 0.00 | 0.00 | 0.00 | 0.00 |
| Chengbu Miao Autonomous County | 227911 | 0.0000 | 0.0000 | 0.0000 | 0.0000 | 0.0000 | 0.0000 | 88.02 | 0.00 | 0.00 | 0.00 | 0.00 | 0.00 | 0.00 | 0.00 | 0.00 | 0.00 | 0.00 | 0.00 | 0.00 | 0.00 | 0.00 | 0.00 | 0.00 | 0.00 |
| Wugang City | 640181 | 0.0000 | 0.0000 | 0.0000 | 0.0000 | 0.0000 | 0.0000 | 415.54 | 0.00 | 0.00 | 0.00 | 0.00 | 0.00 | 0.00 | 0.00 | 0.00 | 0.00 | 0.00 | 0.00 | 0.00 | 0.00 | 0.00 | 0.00 | 0.00 | 0.00 |
| Shaodong City | 1038416 | 0.0000 | 0.0000 | 0.0000 | 0.0000 | 0.0000 | 0.0000 | 584.43 | 0.00 | 0.00 | 0.00 | 0.00 | 0.00 | 0.00 | 0.00 | 0.00 | 0.00 | 0.00 | 0.00 | 0.00 | 0.00 | 0.00 | 0.00 | 0.00 | 0.00 |
| **Yueyang City** | 5051922 | 0.1200 | 0.0000 | 0.0000 | 0.0000 | 0.0000 | 0.1200 | 339.80 | 40.78 | 0.00 | 0.00 | 0.00 | 0.00 | 40.78 | 6.12 | 4.74 | 2.21 | 2.04 | 0.16 | 0.12 | 0.06 | 0.46 | 0.05 | 0.07 | 0.00 |
| Yueyang Building District | 980401 | 0.0000 | 0.0000 | 0.0000 | 0.0000 | 0.0000 | 0.0000 | 2412.29 | 0.00 | 0.00 | 0.00 | 0.00 | 0.00 | 0.00 | 0.00 | 0.00 | 0.00 | 0.00 | 0.00 | 0.00 | 0.00 | 0.00 | 0.00 | 0.00 | 0.00 |
| Yunxi District | 153657 | 0.0000 | 0.0000 | 0.0000 | 0.0000 | 0.0000 | 0.0000 | 406.93 | 0.00 | 0.00 | 0.00 | 0.00 | 0.00 | 0.00 | 0.00 | 0.00 | 0.00 | 0.00 | 0.00 | 0.00 | 0.00 | 0.00 | 0.00 | 0.00 | 0.00 |
| Junshan District | 201634 | 0.0000 | 0.0000 | 0.0000 | 0.0000 | 0.0000 | 0.0000 | 323.23 | 0.00 | 0.00 | 0.00 | 0.00 | 0.00 | 0.00 | 0.00 | 0.00 | 0.00 | 0.00 | 0.00 | 0.00 | 0.00 | 0.00 | 0.00 | 0.00 | 0.00 |
| Yueyang County | 561888 | 0.0000 | 0.0000 | 0.0000 | 0.0000 | 0.0000 | 0.0000 | 199.78 | 0.00 | 0.00 | 0.00 | 0.00 | 0.00 | 0.00 | 0.00 | 0.00 | 0.00 | 0.00 | 0.00 | 0.00 | 0.00 | 0.00 | 0.00 | 0.00 | 0.00 |
| Huarong County | 553800 | 0.0000 | 0.0000 | 0.0000 | 0.0000 | 0.0000 | 0.0000 | 345.65 | 0.00 | 0.00 | 0.00 | 0.00 | 0.00 | 0.00 | 0.00 | 0.00 | 0.00 | 0.00 | 0.00 | 0.00 | 0.00 | 0.00 | 0.00 | 0.00 | 0.00 |
| Xiangyin County | 583984 | 0.0000 | 0.0000 | 0.0000 | 0.0000 | 0.0000 | 0.0000 | 378.77 | 0.00 | 0.00 | 0.00 | 0.00 | 0.00 | 0.00 | 0.00 | 0.00 | 0.00 | 0.00 | 0.00 | 0.00 | 0.00 | 0.00 | 0.00 | 0.00 | 0.00 |
| Pingjiang County | 951112 | 0.0000 | 0.0000 | 0.0000 | 0.0000 | 0.0000 | 0.0000 | 231.18 | 0.00 | 0.00 | 0.00 | 0.00 | 0.00 | 0.00 | 0.00 | 0.00 | 0.00 | 0.00 | 0.00 | 0.00 | 0.00 | 0.00 | 0.00 | 0.00 | 0.00 |
| Miluo City | 632246 | 0.1200 | 0.0000 | 0.0000 | 0.0000 | 0.0000 | 0.1200 | 378.98 | 45.48 | 0.00 | 0.00 | 0.00 | 0.00 | 45.48 | 6.82 | 5.29 | 2.46 | 2.27 | 0.18 | 0.14 | 0.06 | 0.51 | 0.05 | 0.08 | 0.00 |
| Linxiang City | 433200 | 0.0000 | 0.0000 | 0.0000 | 0.0000 | 0.0000 | 0.0000 | 251.80 | 0.00 | 0.00 | 0.00 | 0.00 | 0.00 | 0.00 | 0.00 | 0.00 | 0.00 | 0.00 | 0.00 | 0.00 | 0.00 | 0.00 | 0.00 | 0.00 | 0.00 |
| **Changde City** | 5279102 | 0.0000 | 0.0000 | 0.0000 | 0.0000 | 0.0000 | 0.0000 | 290.42 | 0.00 | 0.00 | 0.00 | 0.00 | 0.00 | 0.00 | 0.00 | 0.00 | 0.00 | 0.00 | 0.00 | 0.00 | 0.00 | 0.00 | 0.00 | 0.00 | 0.00 |
| Wuling District | 730970 | 0.0000 | 0.0000 | 0.0000 | 0.0000 | 0.0000 | 0.0000 | 1774.24 | 0.00 | 0.00 | 0.00 | 0.00 | 0.00 | 0.00 | 0.00 | 0.00 | 0.00 | 0.00 | 0.00 | 0.00 | 0.00 | 0.00 | 0.00 | 0.00 | 0.00 |
| Dingcheng District | 738085 | 0.0000 | 0.0000 | 0.0000 | 0.0000 | 0.0000 | 0.0000 | 315.46 | 0.00 | 0.00 | 0.00 | 0.00 | 0.00 | 0.00 | 0.00 | 0.00 | 0.00 | 0.00 | 0.00 | 0.00 | 0.00 | 0.00 | 0.00 | 0.00 | 0.00 |
| Anxiang County | 427412 | 0.0000 | 0.0000 | 0.0000 | 0.0000 | 0.0000 | 0.0000 | 393.06 | 0.00 | 0.00 | 0.00 | 0.00 | 0.00 | 0.00 | 0.00 | 0.00 | 0.00 | 0.00 | 0.00 | 0.00 | 0.00 | 0.00 | 0.00 | 0.00 | 0.00 |
| Hanshou County | 706249 | 0.0000 | 0.0000 | 0.0000 | 0.0000 | 0.0000 | 0.0000 | 337.56 | 0.00 | 0.00 | 0.00 | 0.00 | 0.00 | 0.00 | 0.00 | 0.00 | 0.00 | 0.00 | 0.00 | 0.00 | 0.00 | 0.00 | 0.00 | 0.00 | 0.00 |
| Li County | 721927 | 0.0000 | 0.0000 | 0.0000 | 0.0000 | 0.0000 | 0.0000 | 347.97 | 0.00 | 0.00 | 0.00 | 0.00 | 0.00 | 0.00 | 0.00 | 0.00 | 0.00 | 0.00 | 0.00 | 0.00 | 0.00 | 0.00 | 0.00 | 0.00 | 0.00 |
| Linli County | 373043 | 0.0000 | 0.0000 | 0.0000 | 0.0000 | 0.0000 | 0.0000 | 310.07 | 0.00 | 0.00 | 0.00 | 0.00 | 0.00 | 0.00 | 0.00 | 0.00 | 0.00 | 0.00 | 0.00 | 0.00 | 0.00 | 0.00 | 0.00 | 0.00 | 0.00 |
| Taoyuan County | 809220 | 0.0000 | 0.0000 | 0.0000 | 0.0000 | 0.0000 | 0.0000 | 182.21 | 0.00 | 0.00 | 0.00 | 0.00 | 0.00 | 0.00 | 0.00 | 0.00 | 0.00 | 0.00 | 0.00 | 0.00 | 0.00 | 0.00 | 0.00 | 0.00 | 0.00 |
| Shimen County | 559457 | 0.0000 | 0.0000 | 0.0000 | 0.0000 | 0.0000 | 0.0000 | 140.86 | 0.00 | 0.00 | 0.00 | 0.00 | 0.00 | 0.00 | 0.00 | 0.00 | 0.00 | 0.00 | 0.00 | 0.00 | 0.00 | 0.00 | 0.00 | 0.00 | 0.00 |
| Jingshi City | 212739 | 0.0000 | 0.0000 | 0.0000 | 0.0000 | 0.0000 | 0.0000 | 382.71 | 0.00 | 0.00 | 0.00 | 0.00 | 0.00 | 0.00 | 0.00 | 0.00 | 0.00 | 0.00 | 0.00 | 0.00 | 0.00 | 0.00 | 0.00 | 0.00 | 0.00 |
| **Zhangjiajie City** | 1517027 | 0.0000 | 0.0000 | 0.0000 | 0.0000 | 0.0000 | 0.0000 | 159.09 | 0.00 | 0.00 | 0.00 | 0.00 | 0.00 | 0.00 | 0.00 | 0.00 | 0.00 | 0.00 | 0.00 | 0.00 | 0.00 | 0.00 | 0.00 | 0.00 | 0.00 |
| Yongding District | 517595 | 0.0000 | 0.0000 | 0.0000 | 0.0000 | 0.0000 | 0.0000 | 238.40 | 0.00 | 0.00 | 0.00 | 0.00 | 0.00 | 0.00 | 0.00 | 0.00 | 0.00 | 0.00 | 0.00 | 0.00 | 0.00 | 0.00 | 0.00 | 0.00 | 0.00 |
| Wulingyuan District | 60857 | 0.0000 | 0.0000 | 0.0000 | 0.0000 | 0.0000 | 0.0000 | 152.88 | 0.00 | 0.00 | 0.00 | 0.00 | 0.00 | 0.00 | 0.00 | 0.00 | 0.00 | 0.00 | 0.00 | 0.00 | 0.00 | 0.00 | 0.00 | 0.00 | 0.00 |
| Cili County | 562493 | 0.0000 | 0.0000 | 0.0000 | 0.0000 | 0.0000 | 0.0000 | 161.07 | 0.00 | 0.00 | 0.00 | 0.00 | 0.00 | 0.00 | 0.00 | 0.00 | 0.00 | 0.00 | 0.00 | 0.00 | 0.00 | 0.00 | 0.00 | 0.00 | 0.00 |
| Sangzhi County | 376082 | 0.0000 | 0.0000 | 0.0000 | 0.0000 | 0.0000 | 0.0000 | 108.25 | 0.00 | 0.00 | 0.00 | 0.00 | 0.00 | 0.00 | 0.00 | 0.00 | 0.00 | 0.00 | 0.00 | 0.00 | 0.00 | 0.00 | 0.00 | 0.00 | 0.00 |
| **Yiyang City** | 3851564 | 0.0000 | 0.0000 | 0.0000 | 0.0000 | 0.0000 | 0.0000 | 312.57 | 0.00 | 0.00 | 0.00 | 0.00 | 0.00 | 0.00 | 0.00 | 0.00 | 0.00 | 0.00 | 0.00 | 0.00 | 0.00 | 0.00 | 0.00 | 0.00 | 0.00 |
| Ziyang District | 356405 | 0.0000 | 0.0000 | 0.0000 | 0.0000 | 0.0000 | 0.0000 | 621.14 | 0.00 | 0.00 | 0.00 | 0.00 | 0.00 | 0.00 | 0.00 | 0.00 | 0.00 | 0.00 | 0.00 | 0.00 | 0.00 | 0.00 | 0.00 | 0.00 | 0.00 |
| Heshan District | 729880 | 0.0000 | 0.0000 | 0.0000 | 0.0000 | 0.0000 | 0.0000 | 570.80 | 0.00 | 0.00 | 0.00 | 0.00 | 0.00 | 0.00 | 0.00 | 0.00 | 0.00 | 0.00 | 0.00 | 0.00 | 0.00 | 0.00 | 0.00 | 0.00 | 0.00 |
| Nan County | 572367 | 0.0000 | 0.0000 | 0.0000 | 0.0000 | 0.0000 | 0.0000 | 431.39 | 0.00 | 0.00 | 0.00 | 0.00 | 0.00 | 0.00 | 0.00 | 0.00 | 0.00 | 0.00 | 0.00 | 0.00 | 0.00 | 0.00 | 0.00 | 0.00 | 0.00 |
| Taojiang County | 685596 | 0.0000 | 0.0000 | 0.0000 | 0.0000 | 0.0000 | 0.0000 | 331.41 | 0.00 | 0.00 | 0.00 | 0.00 | 0.00 | 0.00 | 0.00 | 0.00 | 0.00 | 0.00 | 0.00 | 0.00 | 0.00 | 0.00 | 0.00 | 0.00 | 0.00 |
| Anhua County | 780969 | 0.0000 | 0.0000 | 0.0000 | 0.0000 | 0.0000 | 0.0000 | 157.89 | 0.00 | 0.00 | 0.00 | 0.00 | 0.00 | 0.00 | 0.00 | 0.00 | 0.00 | 0.00 | 0.00 | 0.00 | 0.00 | 0.00 | 0.00 | 0.00 | 0.00 |
| Yuanjiang City | 726347 | 0.0000 | 0.0000 | 0.0000 | 0.0000 | 0.0000 | 0.0000 | 341.31 | 0.00 | 0.00 | 0.00 | 0.00 | 0.00 | 0.00 | 0.00 | 0.00 | 0.00 | 0.00 | 0.00 | 0.00 | 0.00 | 0.00 | 0.00 | 0.00 | 0.00 |
| **Chenzhou City** | 4667134 | 9.3977 | 0.8733 | 0.5153 | 0.3267 | 0.1067 | 11.2197 | 241.27 | 2267.35 | 210.71 | 124.33 | 78.81 | 25.74 | 2706.94 | 564.13 | 437.42 | 203.76 | 188.02 | 14.95 | 11.17 | 5.30 | 42.42 | 4.23 | 6.60 | 0.17 |
| Beihu District | 568778 | 0.1200 | 0.1533 | 0.0400 | 0.0000 | 0.0000 | 0.3133 | 696.98 | 83.64 | 106.87 | 27.88 | 0.00 | 0.00 | 218.39 | 61.33 | 47.56 | 22.15 | 20.44 | 1.63 | 1.21 | 0.58 | 4.61 | 0.46 | 0.72 | 0.02 |
| Suxian District | 435836 | 0.6067 | 0.2333 | 0.3467 | 0.1467 | 0.0733 | 1.4067 | 323.90 | 196.50 | 75.58 | 112.28 | 47.50 | 23.75 | 455.62 | 178.90 | 138.72 | 64.62 | 59.63 | 4.74 | 3.54 | 1.68 | 13.45 | 1.34 | 2.09 | 0.05 |
| Guiyang County | 709372 | 0.0000 | 0.0000 | 0.0000 | 0.0000 | 0.0000 | 0.0000 | 239.56 | 0.00 | 0.00 | 0.00 | 0.00 | 0.00 | 0.00 | 0.00 | 0.00 | 0.00 | 0.00 | 0.00 | 0.00 | 0.00 | 0.00 | 0.00 | 0.00 | 0.00 |
| Yizhang County | 567970 | 8.2840 | 0.1467 | 0.0267 | 0.1467 | 0.0000 | 8.6040 | 267.76 | 2218.11 | 39.27 | 7.14 | 39.27 | 0.00 | 2303.80 | 380.20 | 294.81 | 137.33 | 126.72 | 10.08 | 7.53 | 3.57 | 28.59 | 2.85 | 4.45 | 0.11 |
| Yongxing County | 538532 | 0.0000 | 0.0000 | 0.0000 | 0.0000 | 0.0000 | 0.0000 | 271.92 | 0.00 | 0.00 | 0.00 | 0.00 | 0.00 | 0.00 | 0.00 | 0.00 | 0.00 | 0.00 | 0.00 | 0.00 | 0.00 | 0.00 | 0.00 | 0.00 | 0.00 |
| Jiahe County | 343169 | 0.1333 | 0.1667 | 0.1000 | 0.0333 | 0.0333 | 0.4667 | 491.45 | 65.53 | 81.91 | 49.14 | 16.38 | 16.38 | 229.34 | 91.74 | 71.13 | 33.14 | 30.58 | 2.43 | 1.82 | 0.86 | 6.90 | 0.69 | 1.07 | 0.03 |
| Linwu County | 322987 | 0.0000 | 0.0000 | 0.0000 | 0.0000 | 0.0000 | 0.0000 | 233.90 | 0.00 | 0.00 | 0.00 | 0.00 | 0.00 | 0.00 | 0.00 | 0.00 | 0.00 | 0.00 | 0.00 | 0.00 | 0.00 | 0.00 | 0.00 | 0.00 | 0.00 |
| Rucheng County | 344617 | 0.1467 | 0.0267 | 0.0000 | 0.0000 | 0.0000 | 0.1733 | 143.39 | 21.03 | 3.82 | 0.00 | 0.00 | 0.00 | 24.85 | 4.30 | 3.34 | 1.55 | 1.43 | 0.11 | 0.09 | 0.04 | 0.32 | 0.03 | 0.05 | 0.00 |
| Guidong County | 160956 | 0.0000 | 0.0000 | 0.0000 | 0.0000 | 0.0000 | 0.0000 | 111.06 | 0.00 | 0.00 | 0.00 | 0.00 | 0.00 | 0.00 | 0.00 | 0.00 | 0.00 | 0.00 | 0.00 | 0.00 | 0.00 | 0.00 | 0.00 | 0.00 | 0.00 |
| Anren County | 351927 | 0.0000 | 0.0000 | 0.0020 | 0.0000 | 0.0000 | 0.0020 | 240.31 | 0.00 | 0.00 | 0.48 | 0.00 | 0.00 | 0.48 | 0.29 | 0.22 | 0.10 | 0.10 | 0.01 | 0.01 | 0.00 | 0.02 | 0.00 | 0.00 | 0.00 |
| Zixing City | 322990 | 0.1070 | 0.1467 | 0.0000 | 0.0000 | 0.0000 | 0.2537 | 118.60 | 12.69 | 17.40 | 0.00 | 0.00 | 0.00 | 30.09 | 7.12 | 5.52 | 2.57 | 2.37 | 0.19 | 0.14 | 0.07 | 0.54 | 0.05 | 0.08 | 0.00 |
| **Yongzhou City** | 5289824 | 25.3700 | 3.2313 | 1.7400 | 0.0000 | 0.0000 | 30.3413 | 252.54 | 6406.90 | 816.04 | 439.42 | 0.00 | 0.00 | 7662.35 | 1469.50 | 1139.45 | 530.78 | 489.78 | 38.94 | 29.10 | 13.81 | 110.51 | 11.02 | 17.19 | 0.44 |
| Lingling District | 563556 | 0.5667 | 0.0000 | 0.0000 | 0.0000 | 0.0000 | 0.5667 | 286.81 | 162.53 | 0.00 | 0.00 | 0.00 | 0.00 | 162.53 | 24.38 | 18.90 | 8.81 | 8.13 | 0.65 | 0.48 | 0.23 | 1.83 | 0.18 | 0.29 | 0.01 |
| Lengshuitan area | 583136 | 0.0000 | 0.0000 | 0.0000 | 0.0000 | 0.0000 | 0.0000 | 478.25 | 0.00 | 0.00 | 0.00 | 0.00 | 0.00 | 0.00 | 0.00 | 0.00 | 0.00 | 0.00 | 0.00 | 0.00 | 0.00 | 0.00 | 0.00 | 0.00 | 0.00 |
| Qiyang County | 832813 | 0.0000 | 0.0000 | 0.0000 | 0.0000 | 0.0000 | 0.0000 | 683.03 | 0.00 | 0.00 | 0.00 | 0.00 | 0.00 | 0.00 | 0.00 | 0.00 | 0.00 | 0.00 | 0.00 | 0.00 | 0.00 | 0.00 | 0.00 | 0.00 | 0.00 |
| Dong'an County | 490385 | 0.0000 | 0.0000 | 0.0000 | 0.0000 | 0.0000 | 0.0000 | 222.25 | 0.00 | 0.00 | 0.00 | 0.00 | 0.00 | 0.00 | 0.00 | 0.00 | 0.00 | 0.00 | 0.00 | 0.00 | 0.00 | 0.00 | 0.00 | 0.00 | 0.00 |
| Shuangpai County | 157140 | 0.0000 | 0.0000 | 0.0000 | 0.0000 | 0.0000 | 0.0000 | 90.88 | 0.00 | 0.00 | 0.00 | 0.00 | 0.00 | 0.00 | 0.00 | 0.00 | 0.00 | 0.00 | 0.00 | 0.00 | 0.00 | 0.00 | 0.00 | 0.00 | 0.00 |
| Dao County | 621275 | 0.0000 | 0.0000 | 0.0000 | 0.0000 | 0.0000 | 0.0000 | 254.00 | 0.00 | 0.00 | 0.00 | 0.00 | 0.00 | 0.00 | 0.00 | 0.00 | 0.00 | 0.00 | 0.00 | 0.00 | 0.00 | 0.00 | 0.00 | 0.00 | 0.00 |
| Jiangyong County | 235699 | 0.0133 | 0.0000 | 0.0000 | 0.0000 | 0.0000 | 0.0133 | 144.59 | 1.93 | 0.00 | 0.00 | 0.00 | 0.00 | 1.93 | 0.29 | 0.22 | 0.10 | 0.10 | 0.01 | 0.01 | 0.00 | 0.02 | 0.00 | 0.00 | 0.00 |
| Ningyuan County | 684121 | 0.0000 | 0.0000 | 0.0000 | 0.0000 | 0.0000 | 0.0000 | 273.48 | 0.00 | 0.00 | 0.00 | 0.00 | 0.00 | 0.00 | 0.00 | 0.00 | 0.00 | 0.00 | 0.00 | 0.00 | 0.00 | 0.00 | 0.00 | 0.00 | 0.00 |
| Lanshan County | 329909 | 0.0680 | 0.0053 | 0.0000 | 0.0000 | 0.0000 | 0.0733 | 183.47 | 12.48 | 0.98 | 0.00 | 0.00 | 0.00 | 13.45 | 2.16 | 1.68 | 0.78 | 0.72 | 0.06 | 0.04 | 0.02 | 0.16 | 0.02 | 0.03 | 0.00 |
| Xintian County | 343595 | 0.0000 | 0.0000 | 0.0000 | 0.0000 | 0.0000 | 0.0000 | 344.14 | 0.00 | 0.00 | 0.00 | 0.00 | 0.00 | 0.00 | 0.00 | 0.00 | 0.00 | 0.00 | 0.00 | 0.00 | 0.00 | 0.00 | 0.00 | 0.00 | 0.00 |
| Jianghua Yao Autonomous County | 448195 | 24.7220 | 3.2260 | 1.7400 | 0.0000 | 0.0000 | 29.6880 | 138.61 | 3426.82 | 447.17 | 241.19 | 0.00 | 0.00 | 4115.18 | 792.89 | 614.80 | 286.39 | 264.27 | 21.01 | 15.70 | 7.45 | 59.63 | 5.95 | 9.28 | 0.24 |
| **Huaihua City** | 4587594 | 0.0000 | 0.0000 | 0.0000 | 0.0000 | 0.0000 | 0.0000 | 166.39 | 0.00 | 0.00 | 0.00 | 0.00 | 0.00 | 0.00 | 0.00 | 0.00 | 0.00 | 0.00 | 0.00 | 0.00 | 0.00 | 0.00 | 0.00 | 0.00 | 0.00 |
| Hecheng District | 712584 | 0.0000 | 0.0000 | 0.0000 | 0.0000 | 0.0000 | 0.0000 | 1058.47 | 0.00 | 0.00 | 0.00 | 0.00 | 0.00 | 0.00 | 0.00 | 0.00 | 0.00 | 0.00 | 0.00 | 0.00 | 0.00 | 0.00 | 0.00 | 0.00 | 0.00 |
| Zhongfang County | 233378 | 0.0000 | 0.0000 | 0.0000 | 0.0000 | 0.0000 | 0.0000 | 153.84 | 0.00 | 0.00 | 0.00 | 0.00 | 0.00 | 0.00 | 0.00 | 0.00 | 0.00 | 0.00 | 0.00 | 0.00 | 0.00 | 0.00 | 0.00 | 0.00 | 0.00 |
| Yuanling County | 510054 | 0.0000 | 0.0000 | 0.0000 | 0.0000 | 0.0000 | 0.0000 | 87.44 | 0.00 | 0.00 | 0.00 | 0.00 | 0.00 | 0.00 | 0.00 | 0.00 | 0.00 | 0.00 | 0.00 | 0.00 | 0.00 | 0.00 | 0.00 | 0.00 | 0.00 |
| Chenxi County | 407578 | 0.0000 | 0.0000 | 0.0000 | 0.0000 | 0.0000 | 0.0000 | 205.11 | 0.00 | 0.00 | 0.00 | 0.00 | 0.00 | 0.00 | 0.00 | 0.00 | 0.00 | 0.00 | 0.00 | 0.00 | 0.00 | 0.00 | 0.00 | 0.00 | 0.00 |
| Xupu County | 757797 | 0.0000 | 0.0000 | 0.0000 | 0.0000 | 0.0000 | 0.0000 | 220.96 | 0.00 | 0.00 | 0.00 | 0.00 | 0.00 | 0.00 | 0.00 | 0.00 | 0.00 | 0.00 | 0.00 | 0.00 | 0.00 | 0.00 | 0.00 | 0.00 | 0.00 |
| Huitong County | 291067 | 0.0000 | 0.0000 | 0.0000 | 0.0000 | 0.0000 | 0.0000 | 129.60 | 0.00 | 0.00 | 0.00 | 0.00 | 0.00 | 0.00 | 0.00 | 0.00 | 0.00 | 0.00 | 0.00 | 0.00 | 0.00 | 0.00 | 0.00 | 0.00 | 0.00 |
| Mayang Miao Autonomous County | 313305 | 0.0000 | 0.0000 | 0.0000 | 0.0000 | 0.0000 | 0.0000 | 200.22 | 0.00 | 0.00 | 0.00 | 0.00 | 0.00 | 0.00 | 0.00 | 0.00 | 0.00 | 0.00 | 0.00 | 0.00 | 0.00 | 0.00 | 0.00 | 0.00 | 0.00 |
| Xinhuang Dong Autonomous County | 220775 | 0.0000 | 0.0000 | 0.0000 | 0.0000 | 0.0000 | 0.0000 | 146.99 | 0.00 | 0.00 | 0.00 | 0.00 | 0.00 | 0.00 | 0.00 | 0.00 | 0.00 | 0.00 | 0.00 | 0.00 | 0.00 | 0.00 | 0.00 | 0.00 | 0.00 |
| Zhijiang Dong Autonomous County | 307661 | 0.0000 | 0.0000 | 0.0000 | 0.0000 | 0.0000 | 0.0000 | 147.04 | 0.00 | 0.00 | 0.00 | 0.00 | 0.00 | 0.00 | 0.00 | 0.00 | 0.00 | 0.00 | 0.00 | 0.00 | 0.00 | 0.00 | 0.00 | 0.00 | 0.00 |
| Jingzhou Miao and Dong Autonomous County | 233638 | 0.0000 | 0.0000 | 0.0000 | 0.0000 | 0.0000 | 0.0000 | 105.63 | 0.00 | 0.00 | 0.00 | 0.00 | 0.00 | 0.00 | 0.00 | 0.00 | 0.00 | 0.00 | 0.00 | 0.00 | 0.00 | 0.00 | 0.00 | 0.00 | 0.00 |
| Tongdao Dong Autonomous County | 201047 | 0.0000 | 0.0000 | 0.0000 | 0.0000 | 0.0000 | 0.0000 | 90.35 | 0.00 | 0.00 | 0.00 | 0.00 | 0.00 | 0.00 | 0.00 | 0.00 | 0.00 | 0.00 | 0.00 | 0.00 | 0.00 | 0.00 | 0.00 | 0.00 | 0.00 |
| Hongjiang City | 398710 | 0.0000 | 0.0000 | 0.0000 | 0.0000 | 0.0000 | 0.0000 | 174.20 | 0.00 | 0.00 | 0.00 | 0.00 | 0.00 | 0.00 | 0.00 | 0.00 | 0.00 | 0.00 | 0.00 | 0.00 | 0.00 | 0.00 | 0.00 | 0.00 | 0.00 |
| **Loudi City** | 3826996 | 0.5200 | 0.0000 | 0.0000 | 0.0000 | 0.0000 | 0.5200 | 471.98 | 245.43 | 0.00 | 0.00 | 0.00 | 0.00 | 245.43 | 36.81 | 28.55 | 13.30 | 12.27 | 0.98 | 0.73 | 0.35 | 2.77 | 0.28 | 0.43 | 0.01 |
| Lou Xing District | 752530 | 0.0000 | 0.0000 | 0.0000 | 0.0000 | 0.0000 | 0.0000 | 1210.63 | 0.00 | 0.00 | 0.00 | 0.00 | 0.00 | 0.00 | 0.00 | 0.00 | 0.00 | 0.00 | 0.00 | 0.00 | 0.00 | 0.00 | 0.00 | 0.00 | 0.00 |
| Shuangfeng County | 685917 | 0.5200 | 0.0000 | 0.0000 | 0.0000 | 0.0000 | 0.5200 | 429.21 | 223.19 | 0.00 | 0.00 | 0.00 | 0.00 | 223.19 | 33.48 | 25.96 | 12.09 | 11.16 | 0.89 | 0.66 | 0.31 | 2.52 | 0.25 | 0.39 | 0.01 |
| Xinhua County | 1196538 | 0.0000 | 0.0000 | 0.0000 | 0.0000 | 0.0000 | 0.0000 | 330.86 | 0.00 | 0.00 | 0.00 | 0.00 | 0.00 | 0.00 | 0.00 | 0.00 | 0.00 | 0.00 | 0.00 | 0.00 | 0.00 | 0.00 | 0.00 | 0.00 | 0.00 |
| Lengshuijiang City | 329912 | 0.0000 | 0.0000 | 0.0000 | 0.0000 | 0.0000 | 0.0000 | 752.66 | 0.00 | 0.00 | 0.00 | 0.00 | 0.00 | 0.00 | 0.00 | 0.00 | 0.00 | 0.00 | 0.00 | 0.00 | 0.00 | 0.00 | 0.00 | 0.00 | 0.00 |
| Lianyuan City | 862099 | 0.0000 | 0.0000 | 0.0000 | 0.0000 | 0.0000 | 0.0000 | 470.09 | 0.00 | 0.00 | 0.00 | 0.00 | 0.00 | 0.00 | 0.00 | 0.00 | 0.00 | 0.00 | 0.00 | 0.00 | 0.00 | 0.00 | 0.00 | 0.00 | 0.00 |
| **Xiangxi Tujia and Miao Autonomous Prefecture** | 2488105 | 0.0000 | 0.0000 | 0.0000 | 0.0000 | 0.0000 | 0.0000 | 160.80 | 0.00 | 0.00 | 0.00 | 0.00 | 0.00 | 0.00 | 0.00 | 0.00 | 0.00 | 0.00 | 0.00 | 0.00 | 0.00 | 0.00 | 0.00 | 0.00 | 0.00 |
| Jishou City | 408812 | 0.0000 | 0.0000 | 0.0000 | 0.0000 | 0.0000 | 0.0000 | 379.06 | 0.00 | 0.00 | 0.00 | 0.00 | 0.00 | 0.00 | 0.00 | 0.00 | 0.00 | 0.00 | 0.00 | 0.00 | 0.00 | 0.00 | 0.00 | 0.00 | 0.00 |
| Luxi County | 240937 | 0.0000 | 0.0000 | 0.0000 | 0.0000 | 0.0000 | 0.0000 | 153.76 | 0.00 | 0.00 | 0.00 | 0.00 | 0.00 | 0.00 | 0.00 | 0.00 | 0.00 | 0.00 | 0.00 | 0.00 | 0.00 | 0.00 | 0.00 | 0.00 | 0.00 |
| Phoenix County | 351619 | 0.0000 | 0.0000 | 0.0000 | 0.0000 | 0.0000 | 0.0000 | 202.60 | 0.00 | 0.00 | 0.00 | 0.00 | 0.00 | 0.00 | 0.00 | 0.00 | 0.00 | 0.00 | 0.00 | 0.00 | 0.00 | 0.00 | 0.00 | 0.00 | 0.00 |
| Huayuan County | 249238 | 0.0000 | 0.0000 | 0.0000 | 0.0000 | 0.0000 | 0.0000 | 224.66 | 0.00 | 0.00 | 0.00 | 0.00 | 0.00 | 0.00 | 0.00 | 0.00 | 0.00 | 0.00 | 0.00 | 0.00 | 0.00 | 0.00 | 0.00 | 0.00 | 0.00 |
| Baojing County | 238792 | 0.0000 | 0.0000 | 0.0000 | 0.0000 | 0.0000 | 0.0000 | 136.14 | 0.00 | 0.00 | 0.00 | 0.00 | 0.00 | 0.00 | 0.00 | 0.00 | 0.00 | 0.00 | 0.00 | 0.00 | 0.00 | 0.00 | 0.00 | 0.00 | 0.00 |
| Guzhang County | 108798 | 0.0000 | 0.0000 | 0.0000 | 0.0000 | 0.0000 | 0.0000 | 84.66 | 0.00 | 0.00 | 0.00 | 0.00 | 0.00 | 0.00 | 0.00 | 0.00 | 0.00 | 0.00 | 0.00 | 0.00 | 0.00 | 0.00 | 0.00 | 0.00 | 0.00 |
| Yongshun County | 413470 | 0.0000 | 0.0000 | 0.0000 | 0.0000 | 0.0000 | 0.0000 | 108.44 | 0.00 | 0.00 | 0.00 | 0.00 | 0.00 | 0.00 | 0.00 | 0.00 | 0.00 | 0.00 | 0.00 | 0.00 | 0.00 | 0.00 | 0.00 | 0.00 | 0.00 |
| Longshan County | 476439 | 0.0000 | 0.0000 | 0.0000 | 0.0000 | 0.0000 | 0.0000 | 152.20 | 0.00 | 0.00 | 0.00 | 0.00 | 0.00 | 0.00 | 0.00 | 0.00 | 0.00 | 0.00 | 0.00 | 0.00 | 0.00 | 0.00 | 0.00 | 0.00 | 0.00 |
| **Guangdong Province** | 126012510 | 912.9800 | 438.4903 | 193.7017 | 18.2118 | 3.6133 | 1566.9969 | 708.07 | 646456.21 | 310483.00 | 137154.88 | 12895.25 | 2558.45 | 1109547.79 | 285025.06 | 221008.43 | 102951.05 | 94998.85 | 7553.16 | 5643.50 | 2679.24 | 21433.88 | 2137.69 | 3334.79 | 85.51 |
| **Guangzhou City** | 18676605 | 33.0832 | 17.3249 | 0.6772 | 0.0495 | 0.0013 | 51.1361 | 2588.59 | 85639.07 | 44847.02 | 1753.08 | 128.26 | 3.24 | 132370.67 | 27457.34 | 21290.42 | 9917.59 | 9151.53 | 727.62 | 543.66 | 258.10 | 2064.79 | 205.93 | 321.25 | 8.24 |
| Liwan District | 1238305 | 0.0030 | 0.0000 | 0.0000 | 0.0000 | 0.0000 | 0.0030 | 19844.63 | 58.87 | 0.00 | 0.00 | 0.00 | 0.00 | 58.87 | 8.83 | 6.85 | 3.19 | 2.94 | 0.23 | 0.17 | 0.08 | 0.66 | 0.07 | 0.10 | 0.00 |
| Yuexiu District | 1038643 | 0.0000 | 0.0000 | 0.0000 | 0.0000 | 0.0000 | 0.0000 | 30847.73 | 0.00 | 0.00 | 0.00 | 0.00 | 0.00 | 0.00 | 0.00 | 0.00 | 0.00 | 0.00 | 0.00 | 0.00 | 0.00 | 0.00 | 0.00 | 0.00 | 0.00 |
| Haizhu District | 1819037 | 0.0062 | 0.0000 | 0.0000 | 0.0000 | 0.0000 | 0.0062 | 19765.70 | 122.55 | 0.00 | 0.00 | 0.00 | 0.00 | 122.55 | 18.38 | 14.25 | 6.64 | 6.13 | 0.49 | 0.36 | 0.17 | 1.38 | 0.14 | 0.22 | 0.01 |
| Tianhe District | 2241826 | 0.0348 | 0.0000 | 0.0000 | 0.0000 | 0.0000 | 0.0348 | 16464.64 | 572.97 | 0.00 | 0.00 | 0.00 | 0.00 | 572.97 | 85.95 | 66.64 | 31.04 | 28.65 | 2.28 | 1.70 | 0.81 | 6.46 | 0.64 | 1.01 | 0.03 |
| Baiyun District | 3742991 | 1.3406 | 1.4935 | 0.0546 | 0.0095 | 0.0013 | 2.8995 | 5613.87 | 7525.95 | 8384.43 | 306.33 | 53.59 | 7.04 | 16277.34 | 3877.23 | 3006.40 | 1400.45 | 1292.28 | 102.75 | 76.77 | 36.45 | 291.57 | 29.08 | 45.36 | 1.16 |
| Huangpu District | 1264447 | 2.0773 | 0.2127 | 0.0367 | 0.0000 | 0.0000 | 2.3267 | 2624.48 | 5451.91 | 558.14 | 96.23 | 0.00 | 0.00 | 6106.28 | 1042.97 | 808.72 | 376.72 | 347.62 | 27.64 | 20.65 | 9.80 | 78.43 | 7.82 | 12.20 | 0.31 |
| Panyu District | 2658397 | 2.9093 | 1.6367 | 0.0000 | 0.0000 | 0.0000 | 4.5460 | 5152.23 | 14989.56 | 8432.49 | 0.00 | 0.00 | 0.00 | 23422.05 | 4778.18 | 3705.00 | 1725.88 | 1592.57 | 126.62 | 94.61 | 44.91 | 359.32 | 35.84 | 55.90 | 1.43 |
| Huadu District | 1642360 | 5.2300 | 7.0420 | 0.1033 | 0.0000 | 0.0000 | 12.3753 | 1699.29 | 8887.27 | 11966.37 | 175.59 | 0.00 | 0.00 | 21029.23 | 5028.36 | 3898.99 | 1816.24 | 1675.95 | 133.25 | 99.56 | 47.27 | 378.13 | 37.71 | 58.83 | 1.51 |
| Nansha District | 846584 | 5.7727 | 2.6227 | 0.2427 | 0.0000 | 0.0000 | 8.6380 | 1281.34 | 7396.77 | 3360.54 | 310.94 | 0.00 | 0.00 | 11068.25 | 2304.24 | 1786.71 | 832.29 | 768.00 | 61.06 | 45.62 | 21.66 | 173.28 | 17.28 | 26.96 | 0.69 |
| Conghua District | 717684 | 3.3667 | 3.3200 | 0.2400 | 0.0400 | 0.0000 | 6.9667 | 361.55 | 1217.23 | 1200.36 | 86.77 | 14.46 | 0.00 | 2518.82 | 606.33 | 470.14 | 219.00 | 202.09 | 16.07 | 12.01 | 5.70 | 45.60 | 4.55 | 7.09 | 0.18 |
| Zengcheng District | 1466331 | 12.3427 | 0.9973 | 0.0000 | 0.0000 | 0.0000 | 13.3400 | 908.51 | 11213.40 | 906.08 | 0.00 | 0.00 | 0.00 | 12119.49 | 1953.84 | 1515.00 | 705.73 | 651.21 | 51.78 | 38.69 | 18.37 | 146.93 | 14.65 | 22.86 | 0.59 |
| **Shaoguan City** | 2855131 | 90.4173 | 33.6733 | 2.0167 | 0.0667 | 0.0000 | 126.1740 | 155.09 | 14023.11 | 5222.50 | 312.77 | 10.34 | 0.00 | 19568.72 | 3866.15 | 2997.81 | 1396.45 | 1288.59 | 102.45 | 76.55 | 36.34 | 290.73 | 29.00 | 45.23 | 1.16 |
| Wujiang District | 373686 | 0.5267 | 0.0667 | 0.0000 | 0.0000 | 0.0000 | 0.5933 | 553.04 | 291.27 | 36.87 | 0.00 | 0.00 | 0.00 | 328.13 | 54.75 | 42.45 | 19.78 | 18.25 | 1.45 | 1.08 | 0.51 | 4.12 | 0.41 | 0.64 | 0.02 |
| Zhenjiang District | 364319 | 1.8667 | 0.6667 | 0.1333 | 0.0000 | 0.0000 | 2.6667 | 639.78 | 1194.26 | 426.52 | 85.30 | 0.00 | 0.00 | 1706.09 | 358.28 | 277.81 | 129.41 | 119.41 | 9.49 | 7.09 | 3.37 | 26.94 | 2.69 | 4.19 | 0.11 |
| Qujiang District | 290455 | 19.8333 | 1.2000 | 0.0000 | 0.0000 | 0.0000 | 21.0333 | 179.05 | 3551.16 | 214.86 | 0.00 | 0.00 | 0.00 | 3766.02 | 597.13 | 463.02 | 215.68 | 199.02 | 15.82 | 11.82 | 5.61 | 44.90 | 4.48 | 6.99 | 0.18 |
| Shixing County | 198060 | 8.6667 | 8.0000 | 0.0000 | 0.0000 | 0.0000 | 16.6667 | 92.81 | 804.33 | 742.46 | 0.00 | 0.00 | 0.00 | 1546.79 | 343.39 | 266.26 | 124.03 | 114.45 | 9.10 | 6.80 | 3.23 | 25.82 | 2.58 | 4.02 | 0.10 |
| Renhua County | 186009 | 6.6733 | 4.4667 | 0.7400 | 0.0000 | 0.0000 | 11.8800 | 83.75 | 558.89 | 374.08 | 61.98 | 0.00 | 0.00 | 994.95 | 233.24 | 180.86 | 84.25 | 77.74 | 6.18 | 4.62 | 2.19 | 17.54 | 1.75 | 2.73 | 0.07 |
| Wengyuan County | 322482 | 9.2507 | 4.8713 | 0.9233 | 0.0000 | 0.0000 | 15.0453 | 148.27 | 1371.57 | 722.26 | 136.90 | 0.00 | 0.00 | 2230.74 | 504.55 | 391.23 | 182.25 | 168.17 | 13.37 | 9.99 | 4.74 | 37.94 | 3.78 | 5.90 | 0.15 |
| Ruyuan Yao Autonomous County | 187276 | 4.1200 | 0.7333 | 0.0800 | 0.0000 | 0.0000 | 4.9333 | 81.64 | 336.35 | 59.87 | 6.53 | 0.00 | 0.00 | 402.74 | 72.33 | 56.09 | 26.13 | 24.11 | 1.92 | 1.43 | 0.68 | 5.44 | 0.54 | 0.85 | 0.02 |
| Xinfeng County | 195430 | 8.8000 | 1.7000 | 0.1000 | 0.0667 | 0.0000 | 10.6667 | 99.29 | 873.74 | 168.79 | 9.93 | 6.62 | 0.00 | 1059.08 | 192.95 | 149.61 | 69.69 | 64.31 | 5.11 | 3.82 | 1.81 | 14.51 | 1.45 | 2.26 | 0.06 |
| Lechang City | 383498 | 5.1533 | 0.3133 | 0.0400 | 0.0000 | 0.0000 | 5.5067 | 158.29 | 815.74 | 49.60 | 6.33 | 0.00 | 0.00 | 871.67 | 141.04 | 109.36 | 50.94 | 47.01 | 3.74 | 2.79 | 1.33 | 10.61 | 1.06 | 1.65 | 0.04 |
| Nanxiong City | 353916 | 25.5267 | 11.6553 | 0.0000 | 0.0000 | 0.0000 | 37.1820 | 152.11 | 3882.88 | 1772.90 | 0.00 | 0.00 | 0.00 | 5655.78 | 1114.30 | 864.03 | 402.49 | 371.40 | 29.53 | 22.06 | 10.47 | 83.80 | 8.36 | 13.04 | 0.33 |
| **Shenzhen** | 17494398 | 46.6627 | 1.8100 | 0.1858 | 0.0082 | 0.0000 | 48.6667 | 8981.34 | 419094.05 | 16256.23 | 1668.31 | 73.41 | 0.00 | 437092.01 | 68800.69 | 53348.06 | 24850.81 | 22931.27 | 1823.22 | 1362.25 | 646.73 | 5173.81 | 516.01 | 804.97 | 20.64 |
| Luohu District | 1143801 | 2.6365 | 0.0259 | 0.0043 | 0.0000 | 0.0000 | 2.6667 | 14385.62 | 37927.51 | 371.92 | 62.24 | 0.00 | 0.00 | 38361.67 | 5838.05 | 4526.82 | 2108.70 | 1945.82 | 154.71 | 115.59 | 54.88 | 439.02 | 43.79 | 68.31 | 1.75 |
| Futian District | 1553225 | 1.0252 | 0.0081 | 0.0000 | 0.0000 | 0.0000 | 1.0333 | 21247.95 | 21783.11 | 173.10 | 0.00 | 0.00 | 0.00 | 21956.21 | 3319.40 | 2573.86 | 1198.97 | 1106.35 | 87.96 | 65.72 | 31.20 | 249.62 | 24.90 | 38.84 | 1.00 |
| Nanshan District | 1795826 | 2.8260 | 0.0348 | 0.0012 | 0.0047 | 0.0000 | 2.8667 | 10416.02 | 29435.60 | 362.27 | 12.92 | 48.47 | 0.00 | 29859.26 | 4570.55 | 3544.00 | 1650.88 | 1523.36 | 121.12 | 90.50 | 42.96 | 343.71 | 34.28 | 53.48 | 1.37 |
| Bao'an District | 4476554 | 7.6000 | 0.4000 | 0.0000 | 0.0000 | 0.0000 | 8.0000 | 11939.71 | 90741.77 | 4775.88 | 0.00 | 0.00 | 0.00 | 95517.65 | 15044.03 | 11665.14 | 5433.90 | 5014.18 | 398.67 | 297.87 | 141.41 | 1131.31 | 112.83 | 176.02 | 4.51 |
| Longgang District (+Dapeng) | 4135273 | 12.6678 | 0.1322 | 0.0000 | 0.0000 | 0.0000 | 12.8000 | 6106.43 | 77355.25 | 807.03 | 0.00 | 0.00 | 0.00 | 78162.28 | 11845.40 | 9184.92 | 4278.56 | 3948.07 | 313.90 | 234.54 | 111.35 | 890.77 | 88.84 | 138.59 | 3.55 |
| Yantian District | 214225 | 2.1359 | 0.0237 | 0.0035 | 0.0035 | 0.0000 | 2.1667 | 2921.38 | 6239.78 | 69.31 | 10.28 | 10.28 | 0.00 | 6329.66 | 971.16 | 753.04 | 350.78 | 323.69 | 25.74 | 19.23 | 9.13 | 73.03 | 7.28 | 11.36 | 0.29 |
| Longhua District | 2528872 | 5.2500 | 0.2800 | 0.0700 | 0.0000 | 0.0000 | 5.6000 | 14399.68 | 75598.33 | 4031.91 | 1007.98 | 0.00 | 0.00 | 80638.21 | 13154.11 | 10199.70 | 4751.26 | 4384.26 | 348.58 | 260.45 | 123.65 | 989.19 | 98.66 | 153.90 | 3.95 |
| Pingshan District | 551333 | 4.9480 | 0.0520 | 0.0000 | 0.0000 | 0.0000 | 5.0000 | 3318.08 | 16417.88 | 172.54 | 0.00 | 0.00 | 0.00 | 16590.42 | 2514.44 | 1949.70 | 908.22 | 838.06 | 66.63 | 49.79 | 23.64 | 189.09 | 18.86 | 29.42 | 0.75 |
| Bright District | 1095289 | 7.5733 | 0.8533 | 0.1067 | 0.0000 | 0.0000 | 8.5333 | 7039.13 | 53309.70 | 6006.73 | 750.84 | 0.00 | 0.00 | 60067.26 | 10248.98 | 7947.06 | 3701.93 | 3415.98 | 271.60 | 202.93 | 96.34 | 770.72 | 76.87 | 119.91 | 3.07 |
| **Zhuhai City** | 2439585 | 39.5100 | 15.5667 | 2.8393 | 0.1200 | 0.0000 | 58.0360 | 1538.40 | 60782.33 | 23947.82 | 4368.04 | 184.61 | 0.00 | 89282.79 | 19070.20 | 14787.04 | 6888.16 | 6356.10 | 505.36 | 377.59 | 179.26 | 1434.08 | 143.03 | 223.12 | 5.72 |
| Xiangzhou District | 1384317 | 5.7333 | 5.1793 | 0.8140 | 0.1200 | 0.0000 | 11.8467 | 2841.14 | 16289.20 | 14715.21 | 2312.69 | 340.94 | 0.00 | 33658.04 | 8518.31 | 6605.09 | 3076.81 | 2839.15 | 225.74 | 168.66 | 80.07 | 640.58 | 63.89 | 99.66 | 2.56 |
| Doumen District | 608899 | 26.5247 | 7.5980 | 0.9093 | 0.0000 | 0.0000 | 35.0320 | 1007.24 | 26716.81 | 7653.04 | 915.92 | 0.00 | 0.00 | 35285.76 | 6852.98 | 5313.80 | 2475.30 | 2284.10 | 181.60 | 135.69 | 64.42 | 515.34 | 51.40 | 80.18 | 2.06 |
| Jinwan District | 446369 | 7.2520 | 2.7893 | 1.1160 | 0.0000 | 0.0000 | 11.1573 | 903.53 | 6552.37 | 2520.24 | 1008.34 | 0.00 | 0.00 | 10080.94 | 2343.93 | 1817.48 | 846.63 | 781.23 | 62.11 | 46.41 | 22.03 | 176.26 | 17.58 | 27.42 | 0.70 |
| **Shantou City** | 5502031 | 14.1632 | 0.8094 | 0.2523 | 0.1433 | 0.0000 | 15.3682 | 2527.68 | 35800.07 | 2045.91 | 637.65 | 362.30 | 0.00 | 38845.92 | 6656.21 | 5161.23 | 2404.22 | 2218.52 | 176.39 | 131.79 | 62.57 | 500.55 | 49.92 | 77.88 | 2.00 |
| Longhu District | 630749 | 2.5000 | 0.0000 | 0.1167 | 0.1433 | 0.0000 | 2.7600 | 4803.14 | 12007.86 | 0.00 | 560.37 | 688.45 | 0.00 | 13256.68 | 2688.16 | 2084.40 | 970.96 | 895.96 | 71.24 | 53.23 | 25.27 | 202.15 | 20.16 | 31.45 | 0.81 |
| Jinping District | 777024 | 0.4000 | 0.0000 | 0.0533 | 0.0000 | 0.0000 | 0.4533 | 5194.01 | 2077.60 | 0.00 | 277.01 | 0.00 | 0.00 | 2354.62 | 477.85 | 370.52 | 172.60 | 159.27 | 12.66 | 9.46 | 4.49 | 35.93 | 3.58 | 5.59 | 0.14 |
| Haojiang District | 269471 | 0.1333 | 0.0667 | 0.0667 | 0.0000 | 0.0000 | 0.2667 | 1582.05 | 210.94 | 105.47 | 105.47 | 0.00 | 0.00 | 421.88 | 126.56 | 98.14 | 45.71 | 42.18 | 3.35 | 2.51 | 1.19 | 9.52 | 0.95 | 1.48 | 0.04 |
| Chaoyang District | 1654276 | 3.5900 | 0.1520 | 0.0067 | 0.0000 | 0.0000 | 3.7487 | 2484.31 | 8918.67 | 377.61 | 16.56 | 0.00 | 0.00 | 9312.84 | 1461.02 | 1132.88 | 527.72 | 486.96 | 38.72 | 28.93 | 13.73 | 109.87 | 10.96 | 17.09 | 0.44 |
| Chaonan District | 1231638 | 2.6767 | 0.0000 | 0.0000 | 0.0000 | 0.0000 | 2.6767 | 2053.00 | 5495.21 | 0.00 | 0.00 | 0.00 | 0.00 | 5495.21 | 824.28 | 639.15 | 297.73 | 274.73 | 21.84 | 16.32 | 7.75 | 61.99 | 6.18 | 9.64 | 0.25 |
| Chenghai District | 874444 | 4.8000 | 0.5667 | 0.0000 | 0.0000 | 0.0000 | 5.3667 | 2482.17 | 11914.42 | 1406.56 | 0.00 | 0.00 | 0.00 | 13320.98 | 2209.13 | 1712.96 | 797.94 | 736.30 | 58.54 | 43.74 | 20.77 | 166.13 | 16.57 | 25.85 | 0.66 |
| Nan'ao County | 64429 | 0.0632 | 0.0241 | 0.0089 | 0.0000 | 0.0000 | 0.0962 | 600.12 | 37.93 | 14.44 | 5.36 | 0.00 | 0.00 | 57.73 | 13.24 | 10.27 | 4.78 | 4.41 | 0.35 | 0.26 | 0.12 | 1.00 | 0.10 | 0.15 | 0.00 |
| **Foshan city** | 9498863 | 20.7573 | 22.6260 | 0.6867 | 0.3133 | 0.0567 | 44.4400 | 2502.47 | 51944.67 | 56620.96 | 1718.36 | 784.11 | 141.81 | 111209.91 | 26563.92 | 20597.66 | 9594.89 | 8853.75 | 703.94 | 525.97 | 249.70 | 1997.61 | 199.23 | 310.80 | 7.97 |
| Chancheng District | 1330262 | 0.0000 | 0.4347 | 0.0000 | 0.0000 | 0.0000 | 0.4347 | 8677.51 | 0.00 | 3771.82 | 0.00 | 0.00 | 0.00 | 3771.82 | 1131.55 | 877.40 | 408.71 | 377.14 | 29.99 | 22.40 | 10.64 | 85.09 | 8.49 | 13.24 | 0.34 |
| Nanhai District | 3667247 | 3.3333 | 11.3333 | 0.3000 | 0.2000 | 0.0333 | 15.2000 | 3423.17 | 11410.58 | 38795.98 | 1026.95 | 684.63 | 114.11 | 52032.25 | 14616.96 | 11333.99 | 5279.64 | 4871.83 | 387.35 | 289.42 | 137.40 | 1099.20 | 109.63 | 171.02 | 4.39 |
| Shunde District | 3229090 | 0.8667 | 7.1513 | 0.3200 | 0.1133 | 0.0233 | 8.4747 | 4002.69 | 3469.00 | 28624.57 | 1280.86 | 453.64 | 93.40 | 33921.46 | 10323.20 | 8004.61 | 3728.74 | 3440.72 | 273.56 | 204.40 | 97.04 | 776.30 | 77.42 | 120.78 | 3.10 |
| Sanshui District | 803226 | 8.0907 | 2.4600 | 0.0667 | 0.0000 | 0.0000 | 10.6173 | 971.43 | 7859.51 | 2389.72 | 64.76 | 0.00 | 0.00 | 10313.98 | 1934.70 | 1500.16 | 698.81 | 644.83 | 51.27 | 38.31 | 18.19 | 145.49 | 14.51 | 22.64 | 0.58 |
| Gaoming District | 469038 | 8.4667 | 1.2467 | 0.0000 | 0.0000 | 0.0000 | 9.7133 | 500.25 | 4235.44 | 623.64 | 0.00 | 0.00 | 0.00 | 4859.08 | 822.41 | 637.70 | 297.05 | 274.11 | 21.79 | 16.28 | 7.73 | 61.85 | 6.17 | 9.62 | 0.25 |
| **Jiangmen City** | 4798090 | 28.2988 | 12.8751 | 4.2015 | 0.1312 | 0.0040 | 45.5105 | 512.01 | 14489.27 | 6592.17 | 2151.19 | 67.16 | 2.05 | 23301.85 | 5497.33 | 4262.63 | 1985.64 | 1832.26 | 145.68 | 108.85 | 51.67 | 413.40 | 41.23 | 64.32 | 1.65 |
| Pengjiang District | 853007 | 1.0000 | 0.3333 | 0.3333 | 0.0000 | 0.0000 | 1.6667 | 2660.74 | 2660.74 | 886.91 | 886.91 | 0.00 | 0.00 | 4434.57 | 1197.33 | 928.41 | 432.48 | 399.07 | 31.73 | 23.71 | 11.25 | 90.04 | 8.98 | 14.01 | 0.36 |
| Jianghai District | 364694 | 0.9000 | 0.0000 | 0.0000 | 0.0000 | 0.0000 | 0.9000 | 3348.89 | 3014.00 | 0.00 | 0.00 | 0.00 | 0.00 | 3014.00 | 452.10 | 350.56 | 163.30 | 150.68 | 11.98 | 8.95 | 4.25 | 34.00 | 3.39 | 5.29 | 0.14 |
| Xinhui District | 909277 | 1.1388 | 0.7244 | 0.0188 | 0.0025 | 0.0000 | 1.8845 | 675.94 | 769.77 | 489.67 | 12.71 | 1.69 | 0.00 | 1273.84 | 271.35 | 210.40 | 98.01 | 90.44 | 7.19 | 5.37 | 2.55 | 20.41 | 2.04 | 3.17 | 0.08 |
| Taishan City | 907744 | 9.3333 | 1.0000 | 0.3333 | 0.0000 | 0.0000 | 10.6667 | 286.77 | 2676.53 | 286.77 | 95.59 | 0.00 | 0.00 | 3058.89 | 544.86 | 422.49 | 196.81 | 181.60 | 14.44 | 10.79 | 5.12 | 40.97 | 4.09 | 6.37 | 0.16 |
| Kaiping City | 748777 | 4.7333 | 4.0000 | 0.8667 | 0.0000 | 0.0000 | 9.6000 | 452.11 | 2139.97 | 1808.42 | 391.82 | 0.00 | 0.00 | 4340.21 | 1098.62 | 851.87 | 396.82 | 366.17 | 29.11 | 21.75 | 10.33 | 82.62 | 8.24 | 12.85 | 0.33 |
| Heshan City | 530684 | 4.5680 | 0.9767 | 0.2007 | 0.0367 | 0.0000 | 5.7820 | 489.79 | 2237.35 | 478.36 | 98.28 | 17.96 | 0.00 | 2831.95 | 552.45 | 428.37 | 199.54 | 184.13 | 14.64 | 10.94 | 5.19 | 41.54 | 4.14 | 6.46 | 0.17 |
| Enping City | 483907 | 6.6253 | 5.8407 | 2.4487 | 0.0920 | 0.0040 | 15.0107 | 286.12 | 1895.61 | 1671.10 | 700.60 | 26.32 | 1.14 | 4294.78 | 1228.12 | 952.29 | 443.60 | 409.33 | 32.55 | 24.32 | 11.54 | 92.35 | 9.21 | 14.37 | 0.37 |
| **Zhanjiang City** | 6981236 | 169.0640 | 106.3947 | 78.4680 | 10.6773 | 2.7647 | 367.3687 | 564.92 | 95507.56 | 60104.43 | 44328.11 | 6031.83 | 1561.81 | 207533.73 | 65185.42 | 50544.78 | 23544.98 | 21726.30 | 1727.41 | 1290.67 | 612.74 | 4901.94 | 488.89 | 762.67 | 19.56 |
| Chikan District (+Jingkai District) | 414539 | 1.5293 | 1.0050 | 0.0000 | 0.0000 | 0.0000 | 2.5343 | 6494.42 | 9932.14 | 6526.89 | 0.00 | 0.00 | 0.00 | 16459.03 | 3447.89 | 2673.49 | 1245.38 | 1149.18 | 91.37 | 68.27 | 32.41 | 259.28 | 25.86 | 40.34 | 1.03 |
| Xiashan District (+Jingkai District) | 653403 | 1.6593 | 1.1337 | 0.0753 | 0.0160 | 0.0040 | 2.8883 | 6508.65 | 10800.01 | 7378.64 | 490.32 | 104.14 | 26.03 | 18799.14 | 4234.53 | 3283.45 | 1529.51 | 1411.37 | 112.21 | 83.84 | 39.80 | 318.44 | 31.76 | 49.54 | 1.27 |
| Potou District | 337723 | 3.5533 | 1.8653 | 0.9567 | 0.0000 | 0.0000 | 6.3753 | 671.35 | 2385.53 | 1252.29 | 642.26 | 0.00 | 0.00 | 4280.08 | 1118.87 | 867.57 | 404.14 | 372.92 | 29.65 | 22.15 | 10.52 | 84.14 | 8.39 | 13.09 | 0.34 |
| Mazhang District | 525790 | 3.1473 | 4.1433 | 3.5427 | 0.5573 | 0.2973 | 11.6880 | 716.05 | 2253.66 | 2966.84 | 2536.73 | 399.08 | 212.91 | 8369.22 | 3261.02 | 2528.60 | 1177.88 | 1086.90 | 86.42 | 64.57 | 30.65 | 245.23 | 24.46 | 38.15 | 0.98 |
| Suixi County | 824608 | 35.4667 | 10.2000 | 4.1333 | 2.0667 | 0.2667 | 52.1333 | 409.72 | 14531.50 | 4179.17 | 1693.52 | 846.76 | 109.26 | 21360.21 | 5225.33 | 4051.72 | 1887.39 | 1741.60 | 138.47 | 103.46 | 49.12 | 392.94 | 39.19 | 61.14 | 1.57 |
| Xuwen County | 633258 | 19.3933 | 28.2093 | 24.1333 | 1.3333 | 0.6560 | 73.7253 | 351.38 | 6814.44 | 9912.21 | 8479.98 | 468.51 | 230.51 | 25905.65 | 9666.08 | 7495.08 | 3491.39 | 3221.70 | 256.15 | 191.39 | 90.86 | 726.89 | 72.50 | 113.09 | 2.90 |
| Lianjiang City | 1363470 | 24.5947 | 27.2380 | 27.7880 | 3.5440 | 0.9340 | 84.0987 | 489.63 | 12042.26 | 13336.52 | 13605.81 | 1735.25 | 457.31 | 41177.15 | 15770.56 | 12228.49 | 5696.33 | 5256.33 | 417.92 | 312.26 | 148.24 | 1185.95 | 118.28 | 184.52 | 4.73 |
| Leizhou City | 1321091 | 46.3867 | 16.2000 | 5.8387 | 2.1400 | 0.2067 | 70.7720 | 376.99 | 17487.37 | 6107.26 | 2201.13 | 806.76 | 77.91 | 26680.44 | 6491.49 | 5033.50 | 2344.73 | 2163.61 | 172.02 | 128.53 | 61.02 | 488.16 | 48.69 | 75.95 | 1.95 |
| Wuchuan City | 907354 | 33.3333 | 16.4000 | 12.0000 | 1.0200 | 0.4000 | 63.1533 | 1064.26 | 35475.25 | 17453.82 | 12771.09 | 1085.54 | 425.70 | 67211.41 | 19471.65 | 15098.32 | 7033.16 | 6489.90 | 516.00 | 385.54 | 183.03 | 1464.27 | 146.04 | 227.82 | 5.84 |
| **Maoming City** | 6174050 | 63.6907 | 43.7867 | 12.8753 | 1.7293 | 0.1280 | 122.2100 | 544.21 | 34661.15 | 23829.18 | 7006.90 | 941.12 | 69.66 | 66508.00 | 17367.65 | 13466.88 | 6273.20 | 5788.64 | 460.24 | 343.88 | 163.26 | 1306.05 | 130.26 | 203.20 | 5.21 |
| Maonan District | 1035411 | 12.3067 | 8.3333 | 2.8033 | 0.4433 | 0.0000 | 23.8867 | 1761.02 | 21672.32 | 14675.19 | 4936.73 | 780.72 | 0.00 | 42064.97 | 11240.02 | 8715.51 | 4059.90 | 3746.30 | 297.86 | 222.55 | 105.66 | 845.25 | 84.30 | 131.51 | 3.37 |
| Dianbai District | 1503737 | 7.4467 | 4.5533 | 1.6467 | 0.1733 | 0.0600 | 13.8800 | 735.07 | 5473.84 | 3347.03 | 1210.42 | 127.41 | 44.10 | 10202.80 | 2693.06 | 2088.20 | 972.73 | 897.60 | 71.37 | 53.32 | 25.31 | 202.52 | 20.20 | 31.51 | 0.81 |
| Gaozhou City | 1328657 | 16.4113 | 11.4533 | 1.9107 | 0.5567 | 0.0680 | 30.4000 | 406.02 | 6663.32 | 4650.27 | 775.77 | 226.02 | 27.61 | 12342.98 | 3065.70 | 2377.15 | 1107.33 | 1021.80 | 81.24 | 60.70 | 28.82 | 230.54 | 22.99 | 35.87 | 0.92 |
| Huazhou City | 1291668 | 20.4667 | 17.8067 | 5.3533 | 0.4600 | 0.0000 | 44.0867 | 548.15 | 11218.87 | 9760.78 | 2934.45 | 252.15 | 0.00 | 24166.24 | 6573.45 | 5097.05 | 2374.33 | 2190.93 | 174.20 | 130.15 | 61.79 | 494.32 | 49.30 | 76.91 | 1.97 |
| Xinyi City | 1014577 | 7.0593 | 1.6400 | 1.1613 | 0.0960 | 0.0000 | 9.9567 | 329.14 | 2323.52 | 539.79 | 382.24 | 31.60 | 0.00 | 3277.15 | 765.09 | 593.25 | 276.35 | 255.00 | 20.27 | 15.15 | 7.19 | 57.53 | 5.74 | 8.95 | 0.23 |
| **Zhaoqing City** | 4113594 | 9.4839 | 7.5558 | 4.5421 | 0.0000 | 0.0000 | 21.5818 | 276.09 | 2618.40 | 2086.09 | 1254.03 | 0.00 | 0.00 | 5958.52 | 1771.00 | 1373.24 | 639.69 | 590.28 | 46.93 | 35.07 | 16.65 | 133.18 | 13.28 | 20.72 | 0.53 |
| Duanzhou District | 602402 | 0.0800 | 0.0300 | 0.0000 | 0.0000 | 0.0000 | 0.1100 | 3928.54 | 314.28 | 117.86 | 0.00 | 0.00 | 0.00 | 432.14 | 82.50 | 63.97 | 29.80 | 27.50 | 2.19 | 1.63 | 0.78 | 6.20 | 0.62 | 0.97 | 0.02 |
| Dinghu District | 209116 | 0.2800 | 0.0967 | 0.0233 | 0.0000 | 0.0000 | 0.4000 | 378.12 | 105.87 | 36.55 | 8.82 | 0.00 | 0.00 | 151.25 | 32.14 | 24.92 | 11.61 | 10.71 | 0.85 | 0.64 | 0.30 | 2.42 | 0.24 | 0.38 | 0.01 |
| Gao yao District | 741591 | 1.1927 | 0.9193 | 0.1945 | 0.0000 | 0.0000 | 2.3064 | 339.12 | 404.46 | 311.74 | 65.95 | 0.00 | 0.00 | 782.15 | 193.76 | 150.24 | 69.99 | 64.58 | 5.13 | 3.84 | 1.82 | 14.57 | 1.45 | 2.27 | 0.06 |
| Guangning County | 408112 | 0.8647 | 0.2580 | 0.0000 | 0.0000 | 0.0000 | 1.1227 | 166.18 | 143.69 | 42.88 | 0.00 | 0.00 | 0.00 | 186.57 | 34.42 | 26.69 | 12.43 | 11.47 | 0.91 | 0.68 | 0.32 | 2.59 | 0.26 | 0.40 | 0.01 |
| Huaiji County | 805177 | 3.2179 | 5.5759 | 1.6423 | 0.0000 | 0.0000 | 10.4361 | 226.36 | 728.39 | 1262.15 | 371.75 | 0.00 | 0.00 | 2362.29 | 710.95 | 551.27 | 256.80 | 236.96 | 18.84 | 14.08 | 6.68 | 53.46 | 5.33 | 8.32 | 0.21 |
| Fengkai County | 374848 | 2.5953 | 0.5040 | 0.1807 | 0.0000 | 0.0000 | 3.2800 | 137.54 | 356.97 | 69.32 | 24.85 | 0.00 | 0.00 | 451.14 | 89.25 | 69.21 | 32.24 | 29.75 | 2.37 | 1.77 | 0.84 | 6.71 | 0.67 | 1.04 | 0.03 |
| Deqing County | 331438 | 0.0000 | 0.0000 | 2.4267 | 0.0000 | 0.0000 | 2.4267 | 165.36 | 0.00 | 0.00 | 401.26 | 0.00 | 0.00 | 401.26 | 240.76 | 186.68 | 86.96 | 80.24 | 6.38 | 4.77 | 2.26 | 18.10 | 1.81 | 2.82 | 0.07 |
| Sihui City (+Gaoxin District ) | 640910 | 1.2533 | 0.1720 | 0.0747 | 0.0000 | 0.0000 | 1.5000 | 507.17 | 635.65 | 87.23 | 37.87 | 0.00 | 0.00 | 760.75 | 144.24 | 111.84 | 52.10 | 48.07 | 3.82 | 2.86 | 1.36 | 10.85 | 1.08 | 1.69 | 0.04 |
| **Huizhou City** | 6042852 | 24.9127 | 14.3640 | 9.3613 | 0.9447 | 0.0147 | 49.5973 | 533.76 | 13297.37 | 7666.92 | 4996.70 | 504.22 | 7.83 | 26473.05 | 7703.13 | 5973.01 | 2782.37 | 2567.45 | 204.13 | 152.52 | 72.41 | 579.28 | 57.77 | 90.13 | 2.31 |
| Huicheng District (+Gaoxin District) | 2090578 | 1.6093 | 1.4100 | 0.4873 | 0.0420 | 0.0080 | 3.5567 | 1403.92 | 2259.38 | 1979.53 | 684.18 | 58.96 | 11.23 | 4993.28 | 1400.55 | 1085.99 | 505.88 | 466.80 | 37.11 | 27.73 | 13.17 | 105.32 | 10.50 | 16.39 | 0.42 |
| Huiyang District (+Dayawan) | 1404137 | 3.7240 | 1.4740 | 0.1500 | 0.0000 | 0.0000 | 5.3480 | 1170.21 | 4357.87 | 1724.89 | 175.53 | 0.00 | 0.00 | 6258.29 | 1276.47 | 989.77 | 461.06 | 425.45 | 33.83 | 25.27 | 12.00 | 95.99 | 9.57 | 14.93 | 0.38 |
| Boluo County | 1210878 | 5.5760 | 6.8500 | 7.5873 | 0.8907 | 0.0000 | 20.9040 | 423.58 | 2361.86 | 2901.50 | 3213.82 | 377.27 | 0.00 | 8854.44 | 3454.83 | 2678.88 | 1247.88 | 1151.50 | 91.55 | 68.41 | 32.48 | 259.80 | 25.91 | 40.42 | 1.04 |
| Huidong County | 1018076 | 10.1960 | 3.0367 | 0.7487 | 0.0000 | 0.0000 | 13.9813 | 290.32 | 2960.13 | 881.61 | 217.36 | 0.00 | 0.00 | 4059.10 | 838.92 | 650.50 | 303.02 | 279.61 | 22.23 | 16.61 | 7.89 | 63.09 | 6.29 | 9.82 | 0.25 |
| Longmen County | 319183 | 3.8073 | 1.5933 | 0.3880 | 0.0120 | 0.0067 | 5.8073 | 140.80 | 536.08 | 224.34 | 54.63 | 1.69 | 0.94 | 817.68 | 182.69 | 141.66 | 65.99 | 60.89 | 4.84 | 3.62 | 1.72 | 13.74 | 1.37 | 2.14 | 0.05 |
| **Meizhou City** | 3873239 | 110.6409 | 10.8934 | 1.1893 | 0.4664 | 0.0140 | 123.2040 | 244.14 | 27011.62 | 2659.48 | 290.36 | 113.87 | 3.42 | 30078.74 | 5117.97 | 3968.48 | 1848.61 | 1705.82 | 135.63 | 101.34 | 48.11 | 384.87 | 38.38 | 59.88 | 1.54 |
| Meijiang District | 435616 | 1.5067 | 0.6800 | 0.0000 | 0.0000 | 0.0000 | 2.1867 | 764.21 | 1151.41 | 519.66 | 0.00 | 0.00 | 0.00 | 1671.08 | 328.61 | 254.81 | 118.69 | 109.53 | 8.71 | 6.51 | 3.09 | 24.71 | 2.46 | 3.84 | 0.10 |
| Meixian District | 556735 | 16.7000 | 0.1106 | 0.0000 | 0.0000 | 0.0000 | 16.8106 | 224.92 | 3756.10 | 24.88 | 0.00 | 0.00 | 0.00 | 3780.98 | 570.88 | 442.66 | 206.20 | 190.27 | 15.13 | 11.30 | 5.37 | 42.93 | 4.28 | 6.68 | 0.17 |
| Taipo County | 330948 | 9.3887 | 2.5060 | 0.5540 | 0.0573 | 0.0140 | 12.5200 | 134.47 | 1262.51 | 336.99 | 74.50 | 7.71 | 1.88 | 1683.58 | 343.03 | 265.99 | 123.90 | 114.33 | 9.09 | 6.79 | 3.22 | 25.80 | 2.57 | 4.01 | 0.10 |
| Fengshun County | 478731 | 0.0000 | 0.3680 | 0.0000 | 0.0000 | 0.0000 | 0.3680 | 176.66 | 0.00 | 65.01 | 0.00 | 0.00 | 0.00 | 65.01 | 19.50 | 15.12 | 7.04 | 6.50 | 0.52 | 0.39 | 0.18 | 1.47 | 0.15 | 0.23 | 0.01 |
| Wuhua County | 916961 | 4.8467 | 4.2933 | 0.2767 | 0.0000 | 0.0000 | 9.4167 | 283.25 | 1372.81 | 1216.08 | 78.37 | 0.00 | 0.00 | 2667.26 | 617.77 | 479.02 | 223.14 | 205.90 | 16.37 | 12.23 | 5.81 | 46.46 | 4.63 | 7.23 | 0.19 |
| Pingyuan County | 190482 | 72.2733 | 0.1267 | 0.0000 | 0.3653 | 0.0000 | 72.7653 | 138.70 | 10024.59 | 17.57 | 0.00 | 50.67 | 0.00 | 10092.83 | 1549.50 | 1201.48 | 559.68 | 516.45 | 41.06 | 30.68 | 14.57 | 116.52 | 11.62 | 18.13 | 0.46 |
| Jiaoling County | 184355 | 2.5333 | 1.6000 | 0.0000 | 0.0000 | 0.0000 | 4.1333 | 191.65 | 485.50 | 306.63 | 0.00 | 0.00 | 0.00 | 792.13 | 164.81 | 127.80 | 59.53 | 54.93 | 4.37 | 3.26 | 1.55 | 12.39 | 1.24 | 1.93 | 0.05 |
| Xingning City | 779411 | 3.3923 | 1.2087 | 0.3587 | 0.0437 | 0.0000 | 5.0034 | 375.42 | 1273.53 | 453.78 | 134.65 | 16.42 | 0.00 | 1878.38 | 421.09 | 326.51 | 152.10 | 140.35 | 11.16 | 8.34 | 3.96 | 31.67 | 3.16 | 4.93 | 0.13 |
| **Shanwei City** | 2738482 | 32.3600 | 28.1787 | 18.2107 | 0.9367 | 0.0733 | 79.7593 | 568.22 | 18387.46 | 16011.56 | 10347.59 | 532.23 | 41.67 | 45320.51 | 14233.43 | 11036.60 | 5141.11 | 4744.00 | 377.19 | 281.82 | 133.79 | 1070.35 | 106.75 | 166.53 | 4.27 |
| Cheng District | 450959 | 4.4267 | 1.1653 | 0.3440 | 0.0033 | 0.0000 | 5.9393 | 1194.25 | 5286.53 | 1391.69 | 410.82 | 3.98 | 0.00 | 7093.02 | 1460.16 | 1132.21 | 527.41 | 486.67 | 38.69 | 28.91 | 13.73 | 109.80 | 10.95 | 17.08 | 0.44 |
| Haifeng County | 802454 | 7.0733 | 13.5067 | 8.7933 | 0.5067 | 0.0733 | 29.9533 | 454.91 | 3217.70 | 6144.26 | 4000.14 | 230.49 | 33.36 | 13625.95 | 4940.43 | 3830.81 | 1784.48 | 1646.65 | 130.92 | 97.82 | 46.44 | 371.52 | 37.05 | 57.80 | 1.48 |
| Luhe County | 249242 | 9.3867 | 5.3933 | 2.7667 | 0.3467 | 0.0000 | 17.8933 | 253.18 | 2376.55 | 1365.51 | 700.48 | 87.77 | 0.00 | 4530.31 | 1256.64 | 974.40 | 453.90 | 418.84 | 33.30 | 24.88 | 11.81 | 94.50 | 9.42 | 14.70 | 0.38 |
| Lufeng City | 1235827 | 11.4733 | 8.1133 | 6.3067 | 0.0800 | 0.0000 | 25.9733 | 729.79 | 8373.13 | 5921.03 | 4602.54 | 58.38 | 0.00 | 18955.09 | 5840.51 | 4528.73 | 2109.59 | 1946.64 | 154.77 | 115.64 | 54.90 | 439.21 | 43.80 | 68.33 | 1.75 |
| **Heyuan City** | 2837686 | 55.1187 | 27.9613 | 12.9907 | 0.8040 | 0.2233 | 97.0980 | 181.25 | 9990.51 | 5068.12 | 2354.62 | 145.73 | 40.48 | 17599.45 | 4584.80 | 3555.05 | 1656.03 | 1528.11 | 121.50 | 90.78 | 43.10 | 344.78 | 34.39 | 53.64 | 1.38 |
| Yuancheng District | 703607 | 8.5800 | 9.4933 | 0.5800 | 0.0000 | 0.0000 | 18.6533 | 1946.84 | 16703.88 | 18481.99 | 1129.17 | 0.00 | 0.00 | 36315.03 | 8727.68 | 6767.44 | 3152.44 | 2908.94 | 231.28 | 172.81 | 82.04 | 656.32 | 65.46 | 102.11 | 2.62 |
| Zijin County | 551095 | 2.5433 | 3.6000 | 3.4000 | 0.0933 | 0.0300 | 9.6667 | 151.48 | 385.26 | 545.32 | 515.03 | 14.14 | 4.54 | 1464.30 | 545.80 | 423.22 | 197.14 | 181.92 | 14.46 | 10.81 | 5.13 | 41.04 | 4.09 | 6.39 | 0.16 |
| Longchuan County | 595471 | 13.3333 | 3.3333 | 1.6000 | 0.4000 | 0.0000 | 18.6667 | 193.22 | 2576.29 | 644.07 | 309.15 | 77.29 | 0.00 | 3606.81 | 826.99 | 641.25 | 298.71 | 275.64 | 21.92 | 16.37 | 7.77 | 62.19 | 6.20 | 9.68 | 0.25 |
| Lianping County | 285224 | 10.6807 | 0.2613 | 0.0733 | 0.0000 | 0.0000 | 11.0153 | 125.42 | 1339.60 | 32.78 | 9.20 | 0.00 | 0.00 | 1381.57 | 216.29 | 167.71 | 78.12 | 72.09 | 5.73 | 4.28 | 2.03 | 16.27 | 1.62 | 2.53 | 0.06 |
| Heping County | 353903 | 13.1667 | 4.5533 | 1.1160 | 0.0000 | 0.0000 | 18.8360 | 154.43 | 2033.39 | 703.19 | 172.35 | 0.00 | 0.00 | 2908.94 | 619.38 | 480.26 | 223.72 | 206.44 | 16.41 | 12.26 | 5.82 | 46.58 | 4.65 | 7.25 | 0.19 |
| Dongyuan County | 348386 | 6.8147 | 6.7200 | 6.2213 | 0.3107 | 0.1933 | 20.2600 | 86.91 | 592.23 | 584.00 | 540.67 | 27.00 | 16.80 | 1760.70 | 625.16 | 484.75 | 225.81 | 208.36 | 16.57 | 12.38 | 5.88 | 47.01 | 4.69 | 7.31 | 0.19 |
| **Yangjiang City** | 2602959 | 24.5353 | 26.5540 | 28.1653 | 1.1713 | 0.0600 | 80.4860 | 333.01 | 8170.51 | 8842.75 | 9379.34 | 390.07 | 19.98 | 26802.64 | 9836.04 | 7626.86 | 3552.78 | 3278.35 | 260.65 | 194.75 | 92.46 | 739.67 | 73.77 | 115.08 | 2.95 |
| Jiangcheng District | 814688 | 2.8000 | 3.2000 | 2.3800 | 0.1267 | 0.0267 | 8.5333 | 1147.03 | 3211.68 | 3670.49 | 2729.93 | 145.29 | 30.59 | 9787.97 | 3364.62 | 2608.92 | 1215.30 | 1121.43 | 89.16 | 66.62 | 31.63 | 253.02 | 25.23 | 39.37 | 1.01 |
| Yangdong District | 478299 | 8.2133 | 5.9067 | 1.5533 | 0.0987 | 0.0333 | 15.8053 | 283.12 | 2325.34 | 1672.28 | 439.78 | 27.93 | 9.44 | 4474.77 | 1145.19 | 887.98 | 413.64 | 381.69 | 30.35 | 22.67 | 10.76 | 86.12 | 8.59 | 13.40 | 0.34 |
| Yangxi County | 434076 | 5.3027 | 2.4140 | 0.1387 | 0.0180 | 0.0000 | 7.8733 | 314.91 | 1669.88 | 760.20 | 43.67 | 5.67 | 0.00 | 2479.41 | 509.28 | 394.89 | 183.95 | 169.74 | 13.50 | 10.08 | 4.79 | 38.30 | 3.82 | 5.96 | 0.15 |
| Yangchun City | 875896 | 8.2193 | 15.0333 | 24.0933 | 0.9280 | 0.0000 | 48.2740 | 216.89 | 1782.71 | 3260.61 | 5225.65 | 201.28 | 0.00 | 10470.24 | 4542.00 | 3521.86 | 1640.57 | 1513.85 | 120.36 | 89.93 | 42.69 | 341.56 | 34.06 | 53.14 | 1.36 |
| **Qingyuan City** | 3969473 | 26.7193 | 20.3260 | 8.1663 | 0.1693 | 0.0680 | 55.4490 | 208.54 | 5572.16 | 4238.87 | 1703.04 | 35.31 | 14.18 | 11563.57 | 3170.32 | 2458.27 | 1145.12 | 1056.67 | 84.01 | 62.77 | 29.80 | 238.41 | 23.78 | 37.09 | 0.95 |
| Qingcheng District | 1119901 | 3.3533 | 0.8333 | 0.6333 | 0.1333 | 0.0667 | 5.0200 | 863.32 | 2895.01 | 719.43 | 546.77 | 115.11 | 57.55 | 4333.88 | 1122.03 | 870.02 | 405.28 | 373.97 | 29.73 | 22.22 | 10.55 | 84.38 | 8.42 | 13.13 | 0.34 |
| Qingxin District | 618523 | 5.1427 | 5.6267 | 2.6120 | 0.0000 | 0.0000 | 13.3813 | 263.01 | 1352.58 | 1479.88 | 686.98 | 0.00 | 0.00 | 3519.44 | 1059.04 | 821.18 | 382.53 | 352.98 | 28.06 | 20.97 | 9.95 | 79.64 | 7.94 | 12.39 | 0.32 |
| Fogang County | 315502 | 0.9227 | 1.1153 | 1.8080 | 0.0000 | 0.0000 | 3.8460 | 243.80 | 224.95 | 271.92 | 440.79 | 0.00 | 0.00 | 937.66 | 379.79 | 294.49 | 137.18 | 126.58 | 10.06 | 7.52 | 3.57 | 28.56 | 2.85 | 4.44 | 0.11 |
| Yangshan County | 367175 | 1.4093 | 2.3233 | 1.4303 | 0.0000 | 0.0000 | 5.1630 | 110.32 | 155.48 | 256.32 | 157.80 | 0.00 | 0.00 | 569.59 | 194.90 | 151.12 | 70.40 | 64.96 | 5.16 | 3.86 | 1.83 | 14.66 | 1.46 | 2.28 | 0.06 |
| Lianshan Zhuang Yao Autonomous County | 95136 | 0.9980 | 1.1053 | 0.4920 | 0.0320 | 0.0000 | 2.6273 | 78.24 | 78.08 | 86.48 | 38.49 | 2.50 | 0.00 | 205.55 | 62.75 | 48.66 | 22.67 | 20.92 | 1.66 | 1.24 | 0.59 | 4.72 | 0.47 | 0.73 | 0.02 |
| Liannan Yao Autonomous County | 134691 | 0.4733 | 0.7887 | 0.3160 | 0.0000 | 0.0000 | 1.5780 | 108.40 | 51.31 | 85.49 | 34.26 | 0.00 | 0.00 | 171.06 | 53.90 | 41.79 | 19.47 | 17.96 | 1.43 | 1.07 | 0.51 | 4.05 | 0.40 | 0.63 | 0.02 |
| Yingde City | 941325 | 11.3667 | 7.6800 | 0.7333 | 0.0000 | 0.0000 | 19.7800 | 167.04 | 1898.70 | 1282.87 | 122.50 | 0.00 | 0.00 | 3304.07 | 743.16 | 576.25 | 268.43 | 247.70 | 19.69 | 14.71 | 6.99 | 55.89 | 5.57 | 8.70 | 0.22 |
| Lianzhou City | 377220 | 3.0533 | 0.8533 | 0.1413 | 0.0040 | 0.0013 | 4.0533 | 141.32 | 431.51 | 120.60 | 19.97 | 0.57 | 0.19 | 572.83 | 113.51 | 88.02 | 41.00 | 37.83 | 3.01 | 2.25 | 1.07 | 8.54 | 0.85 | 1.33 | 0.03 |
| **Dongguan City** | 10466625 | 55.6400 | 10.4340 | 0.0087 | 0.0000 | 0.0000 | 66.0827 | 4272.62 | 237728.30 | 44580.47 | 37.03 | 0.00 | 0.00 | 282345.79 | 49055.60 | 38037.71 | 17718.88 | 16350.23 | 1299.97 | 971.30 | 461.12 | 3688.98 | 367.92 | 573.95 | 14.72 |
| Municipal district: Dongguan City | 10466625 | 55.6400 | 10.4340 | 0.0087 | 0.0000 | 0.0000 | 66.0827 | 4272.62 | 237728.30 | 44580.47 | 37.03 | 0.00 | 0.00 | 282345.79 | 49055.60 | 38037.71 | 17718.88 | 16350.23 | 1299.97 | 971.30 | 461.12 | 3688.98 | 367.92 | 573.95 | 14.72 |
| **Zhongshan City** | 4418060 | 10.6360 | 5.0188 | 0.5955 | 0.0125 | 0.0000 | 16.2628 | 2533.15 | 26942.61 | 12713.32 | 1508.57 | 31.58 | 0.00 | 41196.08 | 8785.79 | 6812.51 | 3173.43 | 2928.31 | 232.82 | 173.96 | 82.59 | 660.69 | 65.89 | 102.79 | 2.64 |
| Municipal district: Zhongshan City | 4418060 | 10.6360 | 5.0188 | 0.5955 | 0.0125 | 0.0000 | 16.2628 | 2533.15 | 26942.61 | 12713.32 | 1508.57 | 31.58 | 0.00 | 41196.08 | 8785.79 | 6812.51 | 3173.43 | 2928.31 | 232.82 | 173.96 | 82.59 | 660.69 | 65.89 | 102.79 | 2.64 |
| **Chaozhou City** | 2568387 | 23.2567 | 13.2447 | 2.4227 | 0.0000 | 0.0000 | 38.9240 | 824.69 | 19179.64 | 10922.80 | 1997.96 | 0.00 | 0.00 | 32100.40 | 7352.56 | 5701.18 | 2655.75 | 2450.61 | 194.84 | 145.58 | 69.11 | 552.91 | 55.14 | 86.02 | 2.21 |
| Xiangqiao District | 575795 | 3.0800 | 1.5293 | 0.8093 | 0.0000 | 0.0000 | 5.4187 | 1762.73 | 5429.20 | 2695.80 | 1426.63 | 0.00 | 0.00 | 9551.63 | 2479.10 | 1922.29 | 895.45 | 826.28 | 65.70 | 49.09 | 23.30 | 186.43 | 18.59 | 29.01 | 0.74 |
| Chao'an District | 1175150 | 2.5120 | 0.0613 | 0.0547 | 0.0000 | 0.0000 | 2.6280 | 1080.80 | 2714.96 | 66.29 | 59.08 | 0.00 | 0.00 | 2840.33 | 462.58 | 358.69 | 167.08 | 154.18 | 12.26 | 9.16 | 4.35 | 34.79 | 3.47 | 5.41 | 0.14 |
| Raoping County | 817442 | 17.6647 | 11.6540 | 1.5587 | 0.0000 | 0.0000 | 30.8773 | 480.74 | 8492.03 | 5602.49 | 749.31 | 0.00 | 0.00 | 14843.82 | 3404.13 | 2639.57 | 1229.57 | 1134.60 | 90.21 | 67.40 | 32.00 | 255.99 | 25.53 | 39.83 | 1.02 |
| **Jieyang City** | 5577814 | 11.3078 | 8.2416 | 3.7090 | 0.0273 | 0.0053 | 23.2911 | 1060.81 | 11995.43 | 8742.77 | 3934.54 | 29.00 | 5.66 | 24707.40 | 6811.16 | 5281.37 | 2460.19 | 2270.16 | 180.50 | 134.86 | 64.02 | 512.20 | 51.08 | 79.69 | 2.04 |
| Rongcheng District | 931868 | 1.3253 | 0.4720 | 0.0003 | 0.0000 | 0.0000 | 1.7977 | 2759.13 | 3656.76 | 1302.31 | 0.92 | 0.00 | 0.00 | 4959.99 | 939.76 | 728.69 | 339.44 | 313.22 | 24.90 | 18.61 | 8.83 | 70.67 | 7.05 | 11.00 | 0.28 |
| Jiedong District | 931719 | 3.5040 | 3.0207 | 1.6100 | 0.0047 | 0.0053 | 8.1447 | 1316.30 | 4612.33 | 3976.11 | 2119.25 | 6.14 | 7.02 | 10720.85 | 3167.46 | 2456.05 | 1144.09 | 1055.72 | 83.94 | 62.72 | 29.77 | 238.19 | 23.76 | 37.06 | 0.95 |
| Jiexi County | 674829 | 2.3793 | 1.5200 | 0.2220 | 0.0227 | 0.0000 | 4.1440 | 500.65 | 1191.22 | 760.99 | 111.14 | 11.35 | 0.00 | 2074.70 | 482.75 | 374.32 | 174.37 | 160.90 | 12.79 | 9.56 | 4.54 | 36.30 | 3.62 | 5.65 | 0.14 |
| Huilai County | 1040779 | 0.8291 | 0.4689 | 0.0300 | 0.0000 | 0.0000 | 1.3281 | 835.43 | 692.68 | 391.76 | 25.06 | 0.00 | 0.00 | 1109.51 | 236.47 | 183.36 | 85.41 | 78.81 | 6.27 | 4.68 | 2.22 | 17.78 | 1.77 | 2.77 | 0.07 |
| Puning City | 1998619 | 3.2700 | 2.7600 | 1.8467 | 0.0000 | 0.0000 | 7.8767 | 1234.63 | 4037.24 | 3407.58 | 2279.95 | 0.00 | 0.00 | 9724.77 | 2995.83 | 2322.97 | 1082.09 | 998.51 | 79.39 | 59.32 | 28.16 | 225.29 | 22.47 | 35.05 | 0.90 |
| **Yunfu City** | 2383350 | 22.7213 | 10.8513 | 2.1373 | 0.4407 | 0.2000 | 36.3507 | 306.21 | 6957.51 | 3322.79 | 654.47 | 134.94 | 61.24 | 11130.96 | 2596.22 | 2013.11 | 937.75 | 865.32 | 68.80 | 51.41 | 24.40 | 195.24 | 19.47 | 30.38 | 0.78 |
| Yuncheng District | 408537 | 4.9700 | 2.2940 | 0.0800 | 0.0000 | 0.0000 | 7.3440 | 519.39 | 2581.37 | 1191.48 | 41.55 | 0.00 | 0.00 | 3814.40 | 769.58 | 596.73 | 277.97 | 256.50 | 20.39 | 15.24 | 7.23 | 57.87 | 5.77 | 9.00 | 0.23 |
| Yun'an District | 235390 | 1.9667 | 0.0200 | 0.0000 | 0.0000 | 0.0000 | 1.9867 | 199.42 | 392.18 | 3.99 | 0.00 | 0.00 | 0.00 | 396.17 | 60.02 | 46.54 | 21.68 | 20.01 | 1.59 | 1.19 | 0.56 | 4.51 | 0.45 | 0.70 | 0.02 |
| Xinxing County | 430831 | 7.9847 | 2.4040 | 0.2527 | 0.0407 | 0.0000 | 10.6820 | 283.01 | 2259.77 | 680.36 | 71.51 | 11.51 | 0.00 | 3023.15 | 595.19 | 461.51 | 214.98 | 198.38 | 15.77 | 11.78 | 5.59 | 44.76 | 4.46 | 6.96 | 0.18 |
| Yunan County | 371661 | 3.8667 | 4.2667 | 1.7333 | 0.4000 | 0.2000 | 10.4667 | 189.48 | 732.65 | 808.44 | 328.43 | 75.79 | 37.90 | 1983.20 | 644.23 | 499.53 | 232.69 | 214.72 | 17.07 | 12.76 | 6.06 | 48.45 | 4.83 | 7.54 | 0.19 |
| Luoding City | 936931 | 3.9333 | 1.8667 | 0.0713 | 0.0000 | 0.0000 | 5.8713 | 401.67 | 1579.89 | 749.78 | 28.65 | 0.00 | 0.00 | 2358.33 | 479.11 | 371.50 | 173.05 | 159.69 | 12.70 | 9.49 | 4.50 | 36.03 | 3.59 | 5.61 | 0.14 |
| **Guangxi Province** | 50126804 | 440.7487 | 225.3833 | 79.7328 | 23.8240 | 4.7514 | 774.4402 | 193.84 | 85436.28 | 43689.08 | 15455.68 | 4618.13 | 921.03 | 150120.21 | 39719.01 | 30798.12 | 14346.50 | 13238.34 | 1052.55 | 786.44 | 373.36 | 2986.87 | 297.89 | 464.71 | 11.92 |
| **Nanning** | 8741584 | 36.0347 | 8.6200 | 2.4453 | 0.4340 | 0.0900 | 47.6240 | 395.54 | 14253.04 | 3409.53 | 967.22 | 171.66 | 35.60 | 18837.05 | 3910.52 | 3032.21 | 1412.48 | 1303.37 | 103.63 | 77.43 | 36.76 | 294.07 | 29.33 | 45.75 | 1.17 |
| Xingning District | 615485 | 0.5067 | 0.0067 | 0.0000 | 0.0000 | 0.0000 | 0.5133 | 850.06 | 430.70 | 5.67 | 0.00 | 0.00 | 0.00 | 436.36 | 66.30 | 51.41 | 23.95 | 22.10 | 1.76 | 1.31 | 0.62 | 4.99 | 0.50 | 0.78 | 0.02 |
| Qingxiu District | 1124334 | 5.3333 | 0.6667 | 0.3333 | 0.0000 | 0.0000 | 6.3333 | 1301.16 | 6939.53 | 867.44 | 433.72 | 0.00 | 0.00 | 8240.69 | 1561.39 | 1210.71 | 563.98 | 520.41 | 41.38 | 30.92 | 14.68 | 117.42 | 11.71 | 18.27 | 0.47 |
| Gangnam District | 989823 | 0.5333 | 0.1000 | 0.0667 | 0.0000 | 0.0000 | 0.7000 | 837.63 | 446.73 | 83.76 | 55.84 | 0.00 | 0.00 | 586.34 | 125.64 | 97.42 | 45.38 | 41.88 | 3.33 | 2.49 | 1.18 | 9.45 | 0.94 | 1.47 | 0.04 |
| Xixiangtang District | 1643819 | 2.5333 | 0.5000 | 0.5000 | 0.0000 | 0.0000 | 3.5333 | 1525.30 | 3864.10 | 762.65 | 762.65 | 0.00 | 0.00 | 5389.40 | 1266.00 | 981.66 | 457.28 | 421.96 | 33.55 | 25.07 | 11.90 | 95.20 | 9.50 | 14.81 | 0.38 |
| Liangqing District | 587618 | 2.8600 | 0.2800 | 0.1000 | 0.0000 | 0.0000 | 3.2400 | 428.64 | 1225.90 | 120.02 | 42.86 | 0.00 | 0.00 | 1388.78 | 245.61 | 190.45 | 88.71 | 81.86 | 6.51 | 4.86 | 2.31 | 18.47 | 1.84 | 2.87 | 0.07 |
| Yongning District | 332280 | 3.4200 | 0.4533 | 0.0000 | 0.0000 | 0.0000 | 3.8733 | 270.52 | 925.18 | 122.64 | 0.00 | 0.00 | 0.00 | 1047.82 | 175.57 | 136.14 | 63.42 | 58.52 | 4.65 | 3.48 | 1.65 | 13.20 | 1.32 | 2.05 | 0.05 |
| Wuming District | 683826 | 2.5933 | 0.9000 | 0.3600 | 0.0000 | 0.0000 | 3.8533 | 201.85 | 523.46 | 181.66 | 72.67 | 0.00 | 0.00 | 777.79 | 176.62 | 136.95 | 63.79 | 58.87 | 4.68 | 3.50 | 1.66 | 13.28 | 1.32 | 2.07 | 0.05 |
| Long'an County | 325140 | 3.3133 | 0.3053 | 0.0133 | 0.0000 | 0.0000 | 3.6320 | 140.89 | 466.81 | 43.02 | 1.88 | 0.00 | 0.00 | 511.70 | 84.05 | 65.18 | 30.36 | 28.02 | 2.23 | 1.66 | 0.79 | 6.32 | 0.63 | 0.98 | 0.03 |
| Mashan County | 382430 | 0.1367 | 0.0233 | 0.0133 | 0.0000 | 0.0000 | 0.1733 | 163.35 | 22.33 | 3.81 | 2.18 | 0.00 | 0.00 | 28.31 | 5.80 | 4.50 | 2.09 | 1.93 | 0.15 | 0.11 | 0.05 | 0.44 | 0.04 | 0.07 | 0.00 |
| Shanglin County | 359323 | 1.4000 | 0.0567 | 0.0000 | 0.0000 | 0.0000 | 1.4567 | 192.18 | 269.06 | 10.89 | 0.00 | 0.00 | 0.00 | 279.95 | 43.63 | 33.83 | 15.76 | 14.54 | 1.16 | 0.86 | 0.41 | 3.28 | 0.33 | 0.51 | 0.01 |
| Binyang County | 801407 | 6.4047 | 4.9947 | 1.0587 | 0.4340 | 0.0900 | 12.9820 | 348.65 | 2232.99 | 1741.39 | 369.10 | 151.31 | 31.38 | 4526.17 | 1228.12 | 952.28 | 443.60 | 409.33 | 32.55 | 24.32 | 11.54 | 92.35 | 9.21 | 14.37 | 0.37 |
| Hengzhou City | 896099 | 7.0000 | 0.3333 | 0.0000 | 0.0000 | 0.0000 | 7.3333 | 259.83 | 1818.80 | 86.61 | 0.00 | 0.00 | 0.00 | 1905.41 | 298.80 | 231.69 | 107.93 | 99.59 | 7.92 | 5.92 | 2.81 | 22.47 | 2.24 | 3.50 | 0.09 |
| **Liuzhou City** | 4157934 | 26.8027 | 29.3007 | 17.1000 | 8.2760 | 2.1100 | 83.5893 | 102.41 | 2744.86 | 3000.68 | 1751.21 | 847.55 | 216.09 | 8560.38 | 3235.17 | 2508.55 | 1168.54 | 1078.28 | 85.73 | 64.06 | 30.41 | 243.28 | 24.26 | 37.85 | 0.97 |
| Urban district | 243628 | 3.0713 | 0.6927 | 0.4820 | 0.3667 | 0.0000 | 4.6127 | 3117.04 | 9573.47 | 2159.07 | 1502.41 | 1142.92 | 0.00 | 14377.88 | 3899.52 | 3023.69 | 1408.51 | 1299.71 | 103.34 | 77.21 | 36.66 | 293.24 | 29.25 | 45.62 | 1.17 |
| Yufeng District | 668961 | 3.1593 | 3.6907 | 2.8720 | 0.7413 | 0.0100 | 10.4733 | 774.70 | 2447.53 | 2859.16 | 2224.94 | 574.31 | 7.75 | 8113.69 | 3026.26 | 2346.56 | 1093.09 | 1008.65 | 80.20 | 59.92 | 28.45 | 227.57 | 22.70 | 35.41 | 0.91 |
| Liunan District | 617925 | 0.8727 | 4.1633 | 2.4213 | 0.3793 | 0.0000 | 7.8367 | 1142.51 | 997.03 | 4756.64 | 2766.39 | 433.39 | 0.00 | 8953.45 | 3583.09 | 2778.33 | 1294.21 | 1194.24 | 94.95 | 70.95 | 33.68 | 269.45 | 26.87 | 41.92 | 1.07 |
| Liubei District | 484765 | 1.3087 | 3.5780 | 1.1447 | 0.2487 | 0.0000 | 6.2800 | 1604.44 | 2099.67 | 5740.68 | 1836.55 | 398.97 | 0.00 | 10075.87 | 3458.26 | 2681.53 | 1249.12 | 1152.64 | 91.64 | 68.47 | 32.51 | 260.06 | 25.94 | 40.46 | 1.04 |
| Liujiang District | 503772 | 7.7200 | 7.6467 | 4.9133 | 3.0067 | 0.0000 | 23.2867 | 284.31 | 2194.89 | 2174.04 | 1396.92 | 854.83 | 0.00 | 6620.67 | 2503.46 | 1941.18 | 904.25 | 834.40 | 66.34 | 49.57 | 23.53 | 188.26 | 18.78 | 29.29 | 0.75 |
| Liucheng County | 314242 | 2.5333 | 5.1333 | 3.7333 | 2.8667 | 2.0667 | 16.3333 | 148.61 | 376.47 | 762.84 | 554.80 | 426.00 | 307.12 | 2427.23 | 1235.41 | 957.94 | 446.23 | 411.76 | 32.74 | 24.46 | 11.61 | 92.90 | 9.27 | 14.45 | 0.37 |
| Luzhai County | 337298 | 7.0000 | 3.6667 | 1.0000 | 0.2000 | 0.0333 | 11.9000 | 113.42 | 793.91 | 415.86 | 113.42 | 22.68 | 3.78 | 1349.65 | 333.44 | 258.55 | 120.44 | 111.14 | 8.84 | 6.60 | 3.13 | 25.07 | 2.50 | 3.90 | 0.10 |
| Rong'an County | 253360 | 0.3333 | 0.6667 | 0.5333 | 0.4667 | 0.0000 | 2.0000 | 87.35 | 29.12 | 58.24 | 46.59 | 40.76 | 0.00 | 174.71 | 82.40 | 63.90 | 29.76 | 27.47 | 2.18 | 1.63 | 0.77 | 6.20 | 0.62 | 0.96 | 0.02 |
| Rongshui Miao Autonomous County | 412445 | 0.7000 | 0.0333 | 0.0000 | 0.0000 | 0.0000 | 0.7333 | 88.91 | 62.24 | 2.96 | 0.00 | 0.00 | 0.00 | 65.20 | 10.23 | 7.93 | 3.69 | 3.41 | 0.27 | 0.20 | 0.10 | 0.77 | 0.08 | 0.12 | 0.00 |
| Sanjiang Dong Autonomous County | 321538 | 0.1040 | 0.0293 | 0.0000 | 0.0000 | 0.0000 | 0.1333 | 13.17 | 1.37 | 0.39 | 0.00 | 0.00 | 0.00 | 1.76 | 0.32 | 0.25 | 0.12 | 0.11 | 0.01 | 0.01 | 0.00 | 0.02 | 0.00 | 0.00 | 0.00 |
| **Guilin City** | 4931137 | 53.2167 | 19.8800 | 7.3267 | 0.4733 | 0.0333 | 80.9300 | 178.28 | 9487.49 | 3544.22 | 1306.20 | 84.39 | 5.94 | 14428.24 | 3342.97 | 2592.14 | 1207.48 | 1114.21 | 88.59 | 66.19 | 31.42 | 251.39 | 25.07 | 39.11 | 1.00 |
| Xiufeng District | 161066 | 0.2667 | 0.0667 | 0.0000 | 0.0000 | 0.0000 | 0.3333 | 3807.71 | 1015.39 | 253.85 | 0.00 | 0.00 | 0.00 | 1269.24 | 228.46 | 177.15 | 82.52 | 76.15 | 6.05 | 4.52 | 2.15 | 17.18 | 1.71 | 2.67 | 0.07 |
| Diecai District | 202875 | 0.0000 | 3.4667 | 1.7333 | 0.0000 | 0.0000 | 5.2000 | 4015.74 | 0.00 | 13921.22 | 6960.61 | 0.00 | 0.00 | 20881.83 | 8352.73 | 6476.71 | 3017.01 | 2783.97 | 221.35 | 165.38 | 78.52 | 628.13 | 62.65 | 97.73 | 2.51 |
| Xiangshan District | 286872 | 0.2667 | 0.0667 | 0.0000 | 0.0000 | 0.0000 | 0.3333 | 3199.91 | 853.31 | 213.33 | 0.00 | 0.00 | 0.00 | 1066.64 | 191.99 | 148.87 | 69.35 | 63.99 | 5.09 | 3.80 | 1.80 | 14.44 | 1.44 | 2.25 | 0.06 |
| Qixing District | 387304 | 0.2667 | 0.0667 | 0.0000 | 0.0000 | 0.0000 | 0.3333 | 5509.30 | 1469.15 | 367.29 | 0.00 | 0.00 | 0.00 | 1836.43 | 330.56 | 256.31 | 119.40 | 110.18 | 8.76 | 6.55 | 3.11 | 24.86 | 2.48 | 3.87 | 0.10 |
| Yanshan District | 132639 | 6.6667 | 0.0000 | 0.0000 | 0.0000 | 0.0000 | 6.6667 | 437.09 | 2913.93 | 0.00 | 0.00 | 0.00 | 0.00 | 2913.93 | 437.09 | 338.92 | 157.88 | 145.68 | 11.58 | 8.65 | 4.11 | 32.87 | 3.28 | 5.11 | 0.13 |
| Lingui District | 555109 | 2.3373 | 4.4053 | 3.7333 | 0.0000 | 0.0000 | 10.4760 | 247.06 | 577.45 | 1088.36 | 922.34 | 0.00 | 0.00 | 2588.15 | 966.53 | 749.45 | 349.11 | 322.14 | 25.61 | 19.14 | 9.09 | 72.68 | 7.25 | 11.31 | 0.29 |
| Yangshuo County | 273124 | 11.4467 | 2.3267 | 0.2400 | 0.0400 | 0.0000 | 14.0533 | 190.20 | 2177.13 | 442.53 | 45.65 | 7.61 | 0.00 | 2672.91 | 492.80 | 382.12 | 178.00 | 164.25 | 13.06 | 9.76 | 4.63 | 37.06 | 3.70 | 5.77 | 0.15 |
| Lingchuan County | 422776 | 23.3333 | 6.1107 | 0.0000 | 0.0000 | 0.0000 | 29.4440 | 183.42 | 4279.73 | 1120.80 | 0.00 | 0.00 | 0.00 | 5400.53 | 978.20 | 758.50 | 353.33 | 326.03 | 25.92 | 19.37 | 9.20 | 73.56 | 7.34 | 11.44 | 0.29 |
| Quanzhou County | 565696 | 0.0000 | 0.0000 | 0.0000 | 0.0000 | 0.0000 | 0.0000 | 142.40 | 0.00 | 0.00 | 0.00 | 0.00 | 0.00 | 0.00 | 0.00 | 0.00 | 0.00 | 0.00 | 0.00 | 0.00 | 0.00 | 0.00 | 0.00 | 0.00 | 0.00 |
| Xing'an County | 307043 | 0.6467 | 0.2667 | 0.5867 | 0.0333 | 0.0333 | 1.5667 | 131.69 | 85.16 | 35.12 | 77.26 | 4.39 | 4.39 | 206.32 | 77.13 | 59.81 | 27.86 | 25.71 | 2.04 | 1.53 | 0.73 | 5.80 | 0.58 | 0.90 | 0.02 |
| Yongfu County | 228646 | 0.3527 | 0.0107 | 0.0000 | 0.0000 | 0.0000 | 0.3633 | 81.86 | 28.87 | 0.87 | 0.00 | 0.00 | 0.00 | 29.74 | 4.59 | 3.56 | 1.66 | 1.53 | 0.12 | 0.09 | 0.04 | 0.35 | 0.03 | 0.05 | 0.00 |
| Guanyang County | 208412 | 0.0000 | 0.0000 | 0.0000 | 0.0000 | 0.0000 | 0.0000 | 113.47 | 0.00 | 0.00 | 0.00 | 0.00 | 0.00 | 0.00 | 0.00 | 0.00 | 0.00 | 0.00 | 0.00 | 0.00 | 0.00 | 0.00 | 0.00 | 0.00 | 0.00 |
| Longsheng Autonomous County | 139483 | 0.0000 | 0.0000 | 0.0000 | 0.0000 | 0.0000 | 0.0000 | 56.91 | 0.00 | 0.00 | 0.00 | 0.00 | 0.00 | 0.00 | 0.00 | 0.00 | 0.00 | 0.00 | 0.00 | 0.00 | 0.00 | 0.00 | 0.00 | 0.00 | 0.00 |
| Ziyuan County | 139212 | 0.0000 | 0.0000 | 0.0000 | 0.0000 | 0.0000 | 0.0000 | 71.69 | 0.00 | 0.00 | 0.00 | 0.00 | 0.00 | 0.00 | 0.00 | 0.00 | 0.00 | 0.00 | 0.00 | 0.00 | 0.00 | 0.00 | 0.00 | 0.00 | 0.00 |
| Pingle County | 340921 | 0.0000 | 2.6667 | 1.0000 | 0.4000 | 0.0000 | 4.0667 | 180.12 | 0.00 | 480.33 | 180.12 | 72.05 | 0.00 | 732.50 | 309.81 | 240.23 | 111.90 | 103.26 | 8.21 | 6.13 | 2.91 | 23.30 | 2.32 | 3.62 | 0.09 |
| Gongcheng Yao Autonomous County | 245432 | 6.6667 | 0.4000 | 0.0333 | 0.0000 | 0.0000 | 7.1000 | 114.62 | 764.16 | 45.85 | 3.82 | 0.00 | 0.00 | 813.83 | 130.67 | 101.32 | 47.20 | 43.55 | 3.46 | 2.59 | 1.23 | 9.83 | 0.98 | 1.53 | 0.04 |
| Lipu City | 334527 | 0.9667 | 0.0267 | 0.0000 | 0.0000 | 0.0000 | 0.9933 | 190.66 | 184.30 | 5.08 | 0.00 | 0.00 | 0.00 | 189.39 | 29.17 | 22.62 | 10.54 | 9.72 | 0.77 | 0.58 | 0.27 | 2.19 | 0.22 | 0.34 | 0.01 |
| **Wuzhou City** | 2820977 | 26.6307 | 8.6347 | 1.7480 | 0.0800 | 0.0160 | 37.1093 | 224.50 | 5978.63 | 1938.50 | 392.43 | 17.96 | 3.59 | 8331.10 | 1731.40 | 1342.53 | 625.38 | 577.08 | 45.88 | 34.28 | 16.28 | 130.20 | 12.99 | 20.26 | 0.52 |
| Wanxiu District | 271989 | 0.0000 | 0.0033 | 0.0013 | 0.0067 | 0.0080 | 0.0193 | 606.83 | 0.00 | 2.02 | 0.81 | 4.05 | 4.85 | 11.73 | 8.70 | 6.74 | 3.14 | 2.90 | 0.23 | 0.17 | 0.08 | 0.65 | 0.07 | 0.10 | 0.00 |
| Changzhou District | 299344 | 0.0000 | 0.0013 | 0.0033 | 0.0133 | 0.0067 | 0.0247 | 798.36 | 0.00 | 1.06 | 2.66 | 10.64 | 5.32 | 19.69 | 15.22 | 11.80 | 5.50 | 5.07 | 0.40 | 0.30 | 0.14 | 1.14 | 0.11 | 0.18 | 0.00 |
| Longwei District | 288482 | 0.0000 | 0.0000 | 0.0000 | 0.0033 | 0.0013 | 0.0047 | 296.78 | 0.00 | 0.00 | 0.00 | 0.99 | 0.40 | 1.38 | 1.15 | 0.89 | 0.41 | 0.38 | 0.03 | 0.02 | 0.01 | 0.09 | 0.01 | 0.01 | 0.00 |
| Cangwu County | 276766 | 2.1000 | 0.6167 | 0.1567 | 0.0567 | 0.0000 | 2.9300 | 99.59 | 209.14 | 61.42 | 15.60 | 5.64 | 0.00 | 291.80 | 63.67 | 49.37 | 23.00 | 21.22 | 1.69 | 1.26 | 0.60 | 4.79 | 0.48 | 0.74 | 0.02 |
| Teng County | 795612 | 4.1000 | 1.1000 | 0.1667 | 0.0000 | 0.0000 | 5.3667 | 201.81 | 827.42 | 221.99 | 33.63 | 0.00 | 0.00 | 1083.04 | 210.89 | 163.52 | 76.17 | 70.29 | 5.59 | 4.18 | 1.98 | 15.86 | 1.58 | 2.47 | 0.06 |
| Mengshan County | 164420 | 5.9307 | 0.0000 | 0.0000 | 0.0000 | 0.0000 | 5.9307 | 128.56 | 762.47 | 0.00 | 0.00 | 0.00 | 0.00 | 762.47 | 114.37 | 88.68 | 41.31 | 38.12 | 3.03 | 2.26 | 1.08 | 8.60 | 0.86 | 1.34 | 0.03 |
| Cenxi City | 724364 | 14.5000 | 6.9133 | 1.4200 | 0.0000 | 0.0000 | 22.8333 | 261.50 | 3791.80 | 1807.86 | 371.33 | 0.00 | 0.00 | 5970.99 | 1333.93 | 1034.33 | 481.81 | 444.60 | 35.35 | 26.41 | 12.54 | 100.31 | 10.00 | 15.61 | 0.40 |
| **Beihai City** | 1853227 | 5.1773 | 4.9853 | 0.0700 | 0.0000 | 0.0000 | 10.2327 | 543.77 | 2815.29 | 2710.89 | 38.06 | 0.00 | 0.00 | 5564.25 | 1258.40 | 975.76 | 454.53 | 419.42 | 33.35 | 24.92 | 11.83 | 94.63 | 9.44 | 14.72 | 0.38 |
| Haicheng District | 527895 | 1.6000 | 0.0000 | 0.0000 | 0.0000 | 0.0000 | 1.6000 | 4471.41 | 7154.26 | 0.00 | 0.00 | 0.00 | 0.00 | 7154.26 | 1073.14 | 832.11 | 387.62 | 357.68 | 28.44 | 21.25 | 10.09 | 80.70 | 8.05 | 12.56 | 0.32 |
| Yinhai District | 315196 | 0.8400 | 0.1080 | 0.0000 | 0.0000 | 0.0000 | 0.9480 | 685.10 | 575.49 | 73.99 | 0.00 | 0.00 | 0.00 | 649.48 | 108.52 | 84.15 | 39.20 | 36.17 | 2.88 | 2.15 | 1.02 | 8.16 | 0.81 | 1.27 | 0.03 |
| Tieshangang District | 145943 | 1.4467 | 0.6200 | 0.0000 | 0.0000 | 0.0000 | 2.0667 | 355.21 | 513.88 | 220.23 | 0.00 | 0.00 | 0.00 | 734.11 | 143.15 | 111.00 | 51.71 | 47.71 | 3.79 | 2.83 | 1.35 | 10.76 | 1.07 | 1.67 | 0.04 |
| Hepu County | 864193 | 1.2907 | 4.2573 | 0.0700 | 0.0000 | 0.0000 | 5.6180 | 357.24 | 461.07 | 1520.88 | 25.01 | 0.00 | 0.00 | 2006.96 | 540.43 | 419.05 | 195.20 | 180.12 | 14.32 | 10.70 | 5.08 | 40.64 | 4.05 | 6.32 | 0.16 |
| **Fangchenggang City** | 1046068 | 15.9287 | 4.4867 | 0.9760 | 0.1707 | 0.0000 | 21.5620 | 174.48 | 2779.20 | 782.82 | 170.29 | 29.78 | 0.00 | 3762.09 | 777.72 | 603.05 | 280.91 | 259.22 | 20.61 | 15.40 | 7.31 | 58.48 | 5.83 | 9.10 | 0.23 |
| Gangkou | 244280 | 1.6793 | 0.5660 | 0.0653 | 0.0067 | 0.0000 | 2.3173 | 787.92 | 1323.19 | 445.96 | 51.48 | 5.25 | 0.00 | 1825.88 | 367.36 | 284.85 | 132.69 | 122.44 | 9.73 | 7.27 | 3.45 | 27.63 | 2.76 | 4.30 | 0.11 |
| Fangcheng District | 390961 | 5.4967 | 1.2447 | 0.5440 | 0.1640 | 0.0000 | 7.4493 | 165.02 | 907.05 | 205.39 | 89.77 | 27.06 | 0.00 | 1229.28 | 273.19 | 211.83 | 98.68 | 91.05 | 7.24 | 5.41 | 2.57 | 20.54 | 2.05 | 3.20 | 0.08 |
| Shangsi County | 194774 | 7.4600 | 2.4267 | 0.2947 | 0.0000 | 0.0000 | 10.1813 | 69.20 | 516.26 | 167.93 | 20.39 | 0.00 | 0.00 | 704.59 | 140.05 | 108.60 | 50.59 | 46.68 | 3.71 | 2.77 | 1.32 | 10.53 | 1.05 | 1.64 | 0.04 |
| Dongxing City | 216053 | 1.2927 | 0.2493 | 0.0720 | 0.0000 | 0.0000 | 1.6140 | 430.65 | 556.69 | 107.38 | 31.01 | 0.00 | 0.00 | 695.07 | 134.32 | 104.15 | 48.52 | 44.77 | 3.56 | 2.66 | 1.26 | 10.10 | 1.01 | 1.57 | 0.04 |
| **Qinzhou City** | 3302238 | 27.6487 | 2.8447 | 0.4967 | 0.0320 | 0.0000 | 31.0220 | 308.78 | 8537.49 | 878.39 | 153.36 | 9.88 | 0.00 | 9579.12 | 1644.06 | 1274.81 | 593.84 | 547.97 | 43.57 | 32.55 | 15.45 | 123.63 | 12.33 | 19.24 | 0.49 |
| Qinnan District | 679692 | 11.8300 | 0.6847 | 0.0587 | 0.0000 | 0.0000 | 12.5733 | 284.65 | 3367.43 | 194.89 | 16.70 | 0.00 | 0.00 | 3579.02 | 573.60 | 444.77 | 207.19 | 191.18 | 15.20 | 11.36 | 5.39 | 43.13 | 4.30 | 6.71 | 0.17 |
| Qinbei District | 720442 | 5.7867 | 1.7233 | 0.3467 | 0.0000 | 0.0000 | 7.8567 | 324.67 | 1878.76 | 559.51 | 112.55 | 0.00 | 0.00 | 2550.82 | 517.20 | 401.04 | 186.81 | 172.38 | 13.71 | 10.24 | 4.86 | 38.89 | 3.88 | 6.05 | 0.16 |
| Lingshan County | 1218140 | 7.0000 | 0.2400 | 0.0000 | 0.0000 | 0.0000 | 7.2400 | 342.30 | 2396.09 | 82.15 | 0.00 | 0.00 | 0.00 | 2478.25 | 384.06 | 297.80 | 138.72 | 128.01 | 10.18 | 7.60 | 3.61 | 28.88 | 2.88 | 4.49 | 0.12 |
| Pubei County | 683964 | 3.0320 | 0.1967 | 0.0913 | 0.0320 | 0.0000 | 3.3520 | 270.47 | 820.06 | 53.19 | 24.70 | 8.66 | 0.00 | 906.61 | 160.71 | 124.62 | 58.05 | 53.57 | 4.26 | 3.18 | 1.51 | 12.09 | 1.21 | 1.88 | 0.05 |
| **Guigang City** | 4316262 | 92.0900 | 50.0140 | 4.7987 | 5.3840 | 0.7667 | 153.0533 | 407.93 | 37566.59 | 20402.38 | 1957.54 | 2196.31 | 312.75 | 62435.57 | 14968.75 | 11606.77 | 5406.71 | 4989.08 | 396.67 | 296.38 | 140.71 | 1125.65 | 112.27 | 175.13 | 4.49 |
| Kohoku District | 760633 | 2.5900 | 1.0127 | 3.2220 | 4.3600 | 0.7667 | 11.9513 | 711.00 | 1841.50 | 720.01 | 2290.86 | 3099.98 | 545.10 | 8497.46 | 4837.32 | 3750.86 | 1747.24 | 1612.28 | 128.19 | 95.78 | 45.47 | 363.77 | 36.28 | 56.60 | 1.45 |
| Konan District | 516598 | 15.9433 | 15.2640 | 0.0433 | 0.0240 | 0.0000 | 31.2747 | 469.81 | 7490.26 | 7171.11 | 20.36 | 11.28 | 0.00 | 14693.01 | 3296.11 | 2555.80 | 1190.55 | 1098.59 | 87.35 | 65.26 | 30.98 | 247.87 | 24.72 | 38.56 | 0.99 |
| Qintang District | 423747 | 50.2133 | 15.7000 | 0.0000 | 0.0000 | 0.0000 | 65.9133 | 313.49 | 15741.47 | 4921.82 | 0.00 | 0.00 | 0.00 | 20663.30 | 3837.77 | 2975.81 | 1386.20 | 1279.13 | 101.70 | 75.99 | 36.08 | 288.60 | 28.78 | 44.90 | 1.15 |
| Pingnan County | 1104273 | 10.6667 | 8.5333 | 1.5333 | 1.0000 | 0.0000 | 21.7333 | 369.63 | 3942.73 | 3154.19 | 566.77 | 369.63 | 0.00 | 8033.32 | 2173.43 | 1685.28 | 785.04 | 724.40 | 57.60 | 43.03 | 20.43 | 163.44 | 16.30 | 25.43 | 0.65 |
| Guiping City | 1511011 | 12.6767 | 9.5040 | 0.0000 | 0.0000 | 0.0000 | 22.1807 | 371.06 | 4703.74 | 3526.51 | 0.00 | 0.00 | 0.00 | 8230.25 | 1763.51 | 1367.43 | 636.98 | 587.78 | 46.73 | 34.92 | 16.58 | 132.62 | 13.23 | 20.63 | 0.53 |
| **Yulin City** | 5796766 | 44.8433 | 29.3373 | 6.1413 | 2.3320 | 0.9553 | 83.6093 | 452.04 | 20271.14 | 13261.75 | 2776.15 | 1054.17 | 431.85 | 37795.05 | 9916.88 | 7689.55 | 3581.98 | 3305.30 | 262.80 | 196.35 | 93.22 | 745.75 | 74.38 | 116.03 | 2.98 |
| Yuzhou District | 908121 | 1.2133 | 0.1500 | 0.0200 | 0.0000 | 0.0000 | 1.3833 | 2082.42 | 2526.67 | 312.36 | 41.65 | 0.00 | 0.00 | 2880.68 | 497.70 | 385.91 | 179.77 | 165.88 | 13.19 | 9.85 | 4.68 | 37.43 | 3.73 | 5.82 | 0.15 |
| Fumian District | 318709 | 0.0000 | 2.6333 | 0.0000 | 0.0000 | 0.0000 | 2.6333 | 383.81 | 0.00 | 1010.70 | 0.00 | 0.00 | 0.00 | 1010.70 | 303.21 | 235.11 | 109.52 | 101.06 | 8.04 | 6.00 | 2.85 | 22.80 | 2.27 | 3.55 | 0.09 |
| Rong County | 654916 | 3.5120 | 2.7640 | 1.0953 | 0.2920 | 0.2220 | 7.8853 | 290.45 | 1020.07 | 802.82 | 318.14 | 84.81 | 64.48 | 2290.33 | 710.63 | 551.02 | 256.68 | 236.85 | 18.83 | 14.07 | 6.68 | 53.44 | 5.33 | 8.31 | 0.21 |
| Luchuan County | 804427 | 3.9333 | 6.2667 | 3.0000 | 1.8000 | 0.7333 | 15.7333 | 517.82 | 2036.74 | 3244.98 | 1553.45 | 932.07 | 379.73 | 8146.97 | 3298.49 | 2557.65 | 1191.41 | 1099.39 | 87.41 | 65.31 | 31.01 | 248.05 | 24.74 | 38.59 | 0.99 |
| Bobai County | 1390612 | 5.4333 | 0.2333 | 0.1000 | 0.0333 | 0.0000 | 5.8000 | 363.15 | 1973.12 | 84.74 | 36.32 | 12.11 | 0.00 | 2106.27 | 352.86 | 273.61 | 127.45 | 117.61 | 9.35 | 6.99 | 3.32 | 26.54 | 2.65 | 4.13 | 0.11 |
| Xingye County | 508344 | 2.8547 | 1.4540 | 0.2493 | 0.0133 | 0.0000 | 4.5713 | 346.52 | 989.20 | 503.84 | 86.40 | 4.62 | 0.00 | 1584.06 | 355.07 | 275.32 | 128.25 | 118.34 | 9.41 | 7.03 | 3.34 | 26.70 | 2.66 | 4.15 | 0.11 |
| Beiliu City | 1211637 | 27.8967 | 15.8360 | 1.6767 | 0.1933 | 0.0000 | 45.6027 | 494.06 | 13782.68 | 7823.96 | 828.38 | 95.52 | 0.00 | 22530.53 | 4988.03 | 3867.72 | 1801.68 | 1662.51 | 132.18 | 98.76 | 46.89 | 375.10 | 37.41 | 58.36 | 1.50 |
| **Baise City** | 3571505 | 3.9767 | 5.2353 | 3.5780 | 0.0000 | 0.0001 | 12.7901 | 98.68 | 392.43 | 516.64 | 353.09 | 0.00 | 0.01 | 1262.16 | 425.71 | 330.10 | 153.77 | 141.89 | 11.28 | 8.43 | 4.00 | 32.01 | 3.19 | 4.98 | 0.13 |
| Youjiang District | 472086 | 0.0000 | 2.4087 | 1.7913 | 0.0000 | 0.0000 | 4.2000 | 127.06 | 0.00 | 306.05 | 227.61 | 0.00 | 0.00 | 533.66 | 228.38 | 177.09 | 82.49 | 76.12 | 6.05 | 4.52 | 2.15 | 17.17 | 1.71 | 2.67 | 0.07 |
| Tianyang District | 308785 | 0.6333 | 1.0000 | 1.0333 | 0.0000 | 0.0000 | 2.6667 | 130.15 | 82.43 | 130.15 | 134.48 | 0.00 | 0.00 | 347.06 | 132.10 | 102.43 | 47.71 | 44.03 | 3.50 | 2.62 | 1.24 | 9.93 | 0.99 | 1.55 | 0.04 |
| Tiandong County | 352007 | 0.6100 | 0.2933 | 0.0200 | 0.0000 | 0.0000 | 0.9233 | 125.26 | 76.41 | 36.74 | 2.51 | 0.00 | 0.00 | 115.66 | 23.99 | 18.60 | 8.66 | 8.00 | 0.64 | 0.47 | 0.23 | 1.80 | 0.18 | 0.28 | 0.01 |
| DePaul County | 269758 | 0.0000 | 0.0000 | 0.0000 | 0.0000 | 0.0000 | 0.0000 | 104.65 | 0.00 | 0.00 | 0.00 | 0.00 | 0.00 | 0.00 | 0.00 | 0.00 | 0.00 | 0.00 | 0.00 | 0.00 | 0.00 | 0.00 | 0.00 | 0.00 | 0.00 |
| Napo County | 171781 | 0.0000 | 0.0000 | 0.0000 | 0.0000 | 0.0000 | 0.0000 | 77.50 | 0.00 | 0.00 | 0.00 | 0.00 | 0.00 | 0.00 | 0.00 | 0.00 | 0.00 | 0.00 | 0.00 | 0.00 | 0.00 | 0.00 | 0.00 | 0.00 | 0.00 |
| Lingyun County | 188194 | 0.0000 | 0.0000 | 0.0000 | 0.0000 | 0.0000 | 0.0000 | 91.75 | 0.00 | 0.00 | 0.00 | 0.00 | 0.00 | 0.00 | 0.00 | 0.00 | 0.00 | 0.00 | 0.00 | 0.00 | 0.00 | 0.00 | 0.00 | 0.00 | 0.00 |
| Leye County | 146397 | 0.0000 | 0.0000 | 0.0000 | 0.0000 | 0.0000 | 0.0000 | 55.57 | 0.00 | 0.00 | 0.00 | 0.00 | 0.00 | 0.00 | 0.00 | 0.00 | 0.00 | 0.00 | 0.00 | 0.00 | 0.00 | 0.00 | 0.00 | 0.00 | 0.00 |
| Tianlin County | 224828 | 1.4667 | 0.2000 | 0.0667 | 0.0000 | 0.0000 | 1.7333 | 40.76 | 59.78 | 8.15 | 2.72 | 0.00 | 0.00 | 70.65 | 13.04 | 10.11 | 4.71 | 4.35 | 0.35 | 0.26 | 0.12 | 0.98 | 0.10 | 0.15 | 0.00 |
| Xilin County | 142098 | 0.0000 | 0.0000 | 0.0000 | 0.0000 | 0.0000 | 0.0000 | 47.39 | 0.00 | 0.00 | 0.00 | 0.00 | 0.00 | 0.00 | 0.00 | 0.00 | 0.00 | 0.00 | 0.00 | 0.00 | 0.00 | 0.00 | 0.00 | 0.00 | 0.00 |
| Longlin Autonomous County | 350765 | 0.0000 | 0.0000 | 0.0000 | 0.0000 | 0.0001 | 0.0001 | 99.66 | 0.00 | 0.00 | 0.00 | 0.00 | 0.01 | 0.01 | 0.01 | 0.00 | 0.00 | 0.00 | 0.00 | 0.00 | 0.00 | 0.00 | 0.00 | 0.00 | 0.00 |
| Jingxi City | 489163 | 1.1333 | 0.0000 | 0.0000 | 0.0000 | 0.0000 | 1.1333 | 147.14 | 166.76 | 0.00 | 0.00 | 0.00 | 0.00 | 166.76 | 25.01 | 19.40 | 9.03 | 8.34 | 0.66 | 0.50 | 0.24 | 1.88 | 0.19 | 0.29 | 0.01 |
| Pingguo City | 455643 | 0.1333 | 1.3333 | 0.6667 | 0.0000 | 0.0000 | 2.1333 | 185.51 | 24.74 | 247.35 | 123.68 | 0.00 | 0.00 | 395.76 | 152.12 | 117.96 | 54.95 | 50.70 | 4.03 | 3.01 | 1.43 | 11.44 | 1.14 | 1.78 | 0.05 |
| **Hezhou City** | 2007858 | 76.1593 | 49.2480 | 22.4220 | 3.7460 | 0.1333 | 151.7087 | 170.81 | 13008.58 | 8411.92 | 3829.84 | 639.84 | 22.77 | 25912.96 | 7305.14 | 5664.41 | 2638.62 | 2434.80 | 193.59 | 144.64 | 68.67 | 549.35 | 54.79 | 85.47 | 2.19 |
| Babu District | 655994 | 17.0667 | 23.7333 | 5.2000 | 0.5933 | 0.0000 | 46.5933 | 179.18 | 3058.08 | 4252.64 | 931.76 | 106.32 | 0.00 | 8348.80 | 2378.61 | 1844.38 | 859.15 | 792.79 | 63.03 | 47.10 | 22.36 | 178.87 | 17.84 | 27.83 | 0.71 |
| Pinggui District | 404161 | 2.0000 | 0.2200 | 0.0000 | 0.0000 | 0.0000 | 2.2200 | 218.08 | 436.15 | 47.98 | 0.00 | 0.00 | 0.00 | 484.13 | 79.82 | 61.89 | 28.83 | 26.60 | 2.12 | 1.58 | 0.75 | 6.00 | 0.60 | 0.93 | 0.02 |
| Zhaoping County | 330116 | 4.7467 | 0.2467 | 0.0467 | 0.0000 | 0.0000 | 5.0400 | 102.29 | 485.51 | 25.23 | 4.77 | 0.00 | 0.00 | 515.52 | 83.26 | 64.56 | 30.07 | 27.75 | 2.21 | 1.65 | 0.78 | 6.26 | 0.62 | 0.97 | 0.02 |
| Zhongshan County | 351057 | 11.0127 | 1.3813 | 0.1753 | 0.1060 | 0.0000 | 12.6753 | 238.55 | 2627.12 | 329.52 | 41.83 | 25.29 | 0.00 | 3023.76 | 538.25 | 417.36 | 194.42 | 179.40 | 14.26 | 10.66 | 5.06 | 40.48 | 4.04 | 6.30 | 0.16 |
| Fuchuan Yao Autonomous County | 266530 | 41.3333 | 23.6667 | 17.0000 | 3.0467 | 0.1333 | 85.1800 | 172.87 | 7145.27 | 4091.24 | 2938.78 | 526.68 | 23.05 | 14725.01 | 4504.51 | 3492.80 | 1627.03 | 1501.35 | 119.37 | 89.19 | 42.34 | 338.74 | 33.78 | 52.70 | 1.35 |
| **Hechi City** | 3417945 | 2.7487 | 3.4277 | 0.3853 | 0.0127 | 0.0000 | 6.5743 | 102.09 | 280.61 | 349.93 | 39.34 | 1.29 | 0.00 | 671.17 | 171.71 | 133.14 | 62.02 | 57.23 | 4.55 | 3.40 | 1.61 | 12.91 | 1.29 | 2.01 | 0.05 |
| Jinchengjiang District | 372521 | 0.7053 | 0.4910 | 0.1887 | 0.0127 | 0.0000 | 1.3977 | 158.78 | 112.00 | 77.96 | 29.96 | 2.01 | 0.00 | 221.93 | 59.77 | 46.35 | 21.59 | 19.92 | 1.58 | 1.18 | 0.56 | 4.49 | 0.45 | 0.70 | 0.02 |
| Yizhou District | 549359 | 1.8533 | 0.6800 | 0.0633 | 0.0000 | 0.0000 | 2.5967 | 142.54 | 264.18 | 96.93 | 9.03 | 0.00 | 0.00 | 370.14 | 74.12 | 57.47 | 26.77 | 24.70 | 1.96 | 1.47 | 0.70 | 5.57 | 0.56 | 0.87 | 0.02 |
| Nandan County | 275554 | 0.0000 | 0.0000 | 0.0000 | 0.0000 | 0.0000 | 0.0000 | 70.57 | 0.00 | 0.00 | 0.00 | 0.00 | 0.00 | 0.00 | 0.00 | 0.00 | 0.00 | 0.00 | 0.00 | 0.00 | 0.00 | 0.00 | 0.00 | 0.00 | 0.00 |
| Tian'e County | 143155 | 0.0133 | 0.0000 | 0.0000 | 0.0000 | 0.0000 | 0.0133 | 44.95 | 0.60 | 0.00 | 0.00 | 0.00 | 0.00 | 0.60 | 0.09 | 0.07 | 0.03 | 0.03 | 0.00 | 0.00 | 0.00 | 0.01 | 0.00 | 0.00 | 0.00 |
| Fengshan County | 169845 | 0.0000 | 0.0000 | 0.0000 | 0.0000 | 0.0000 | 0.0000 | 98.20 | 0.00 | 0.00 | 0.00 | 0.00 | 0.00 | 0.00 | 0.00 | 0.00 | 0.00 | 0.00 | 0.00 | 0.00 | 0.00 | 0.00 | 0.00 | 0.00 | 0.00 |
| Donglan County | 219549 | 0.0000 | 0.0000 | 0.0000 | 0.0000 | 0.0000 | 0.0000 | 90.59 | 0.00 | 0.00 | 0.00 | 0.00 | 0.00 | 0.00 | 0.00 | 0.00 | 0.00 | 0.00 | 0.00 | 0.00 | 0.00 | 0.00 | 0.00 | 0.00 | 0.00 |
| Luochengmulao Autonomous County | 272672 | 0.1667 | 0.1333 | 0.1333 | 0.0000 | 0.0000 | 0.4333 | 102.81 | 17.13 | 13.71 | 13.71 | 0.00 | 0.00 | 44.55 | 14.91 | 11.56 | 5.38 | 4.97 | 0.40 | 0.30 | 0.14 | 1.12 | 0.11 | 0.17 | 0.00 |
| Huanjiangmaonan Autonomous County | 276076 | 0.0100 | 0.0100 | 0.0000 | 0.0000 | 0.0000 | 0.0200 | 60.63 | 0.61 | 0.61 | 0.00 | 0.00 | 0.00 | 1.21 | 0.27 | 0.21 | 0.10 | 0.09 | 0.01 | 0.01 | 0.00 | 0.02 | 0.00 | 0.00 | 0.00 |
| Bamayao Autonomous County | 236152 | 0.0000 | 0.0000 | 0.0000 | 0.0000 | 0.0000 | 0.0000 | 119.34 | 0.00 | 0.00 | 0.00 | 0.00 | 0.00 | 0.00 | 0.00 | 0.00 | 0.00 | 0.00 | 0.00 | 0.00 | 0.00 | 0.00 | 0.00 | 0.00 | 0.00 |
| Du'anyao Autonomous County | 538061 | 0.0000 | 2.1000 | 0.0000 | 0.0000 | 0.0000 | 2.1000 | 132.19 | 0.00 | 277.59 | 0.00 | 0.00 | 0.00 | 277.59 | 83.28 | 64.57 | 30.08 | 27.76 | 2.21 | 1.65 | 0.78 | 6.26 | 0.62 | 0.97 | 0.02 |
| Dahuayao Autonomous County | 365001 | 0.0000 | 0.0133 | 0.0000 | 0.0000 | 0.0000 | 0.0133 | 131.19 | 0.00 | 1.75 | 0.00 | 0.00 | 0.00 | 1.75 | 0.52 | 0.41 | 0.19 | 0.17 | 0.01 | 0.01 | 0.00 | 0.04 | 0.00 | 0.01 | 0.00 |
| **Come to the city** | 2074611 | 26.5605 | 8.5027 | 12.0257 | 2.8667 | 0.6467 | 50.6022 | 154.97 | 4116.10 | 1317.66 | 1863.63 | 444.25 | 100.21 | 7841.86 | 2576.48 | 1997.80 | 930.63 | 858.74 | 68.28 | 51.01 | 24.22 | 193.75 | 19.32 | 30.14 | 0.77 |
| Xingbin District | 925377 | 5.9667 | 1.8800 | 5.3067 | 2.8667 | 0.6467 | 16.6667 | 210.08 | 1253.50 | 394.96 | 1114.84 | 602.24 | 135.85 | 3501.40 | 1579.48 | 1224.73 | 570.51 | 526.44 | 41.86 | 31.27 | 14.85 | 118.78 | 11.85 | 18.48 | 0.47 |
| Xincheng County | 297825 | 0.0480 | 0.8627 | 0.0000 | 0.0000 | 0.0000 | 0.9107 | 118.06 | 5.67 | 101.85 | 0.00 | 0.00 | 0.00 | 107.52 | 31.40 | 24.35 | 11.34 | 10.47 | 0.83 | 0.62 | 0.30 | 2.36 | 0.24 | 0.37 | 0.01 |
| Xiangzhou County | 281846 | 9.2467 | 2.5467 | 0.2400 | 0.0000 | 0.0000 | 12.0333 | 146.79 | 1357.36 | 373.84 | 35.23 | 0.00 | 0.00 | 1766.43 | 336.89 | 261.23 | 121.69 | 112.29 | 8.93 | 6.67 | 3.17 | 25.33 | 2.53 | 3.94 | 0.10 |
| Wuxuan County | 340312 | 6.7733 | 3.2000 | 6.3800 | 0.0000 | 0.0000 | 16.3533 | 199.92 | 1354.16 | 639.76 | 1275.52 | 0.00 | 0.00 | 3269.44 | 1160.36 | 899.75 | 419.12 | 386.75 | 30.75 | 22.98 | 10.91 | 87.26 | 8.70 | 13.58 | 0.35 |
| Jinxiuyao Autonomous County | 130313 | 0.1925 | 0.0133 | 0.0990 | 0.0000 | 0.0000 | 0.3048 | 52.71 | 10.15 | 0.70 | 5.22 | 0.00 | 0.00 | 16.07 | 4.86 | 3.77 | 1.76 | 1.62 | 0.13 | 0.10 | 0.05 | 0.37 | 0.04 | 0.06 | 0.00 |
| Heshan City | 98938 | 4.3333 | 0.0000 | 0.0000 | 0.0000 | 0.0000 | 4.3333 | 270.83 | 1173.61 | 0.00 | 0.00 | 0.00 | 0.00 | 1173.61 | 176.04 | 136.50 | 63.59 | 58.67 | 4.67 | 3.49 | 1.65 | 13.24 | 1.32 | 2.06 | 0.05 |
| **Chongzuo City** | 2088692 | 2.9309 | 0.8663 | 0.2191 | 0.0167 | 0.0000 | 4.0330 | 120.38 | 352.81 | 104.28 | 26.38 | 2.01 | 0.00 | 485.47 | 101.64 | 78.81 | 36.71 | 33.88 | 2.69 | 2.01 | 0.96 | 7.64 | 0.76 | 1.19 | 0.03 |
| Jiangzhou District | 435438 | 0.6533 | 0.4567 | 0.1200 | 0.0167 | 0.0000 | 1.2467 | 149.34 | 97.57 | 68.20 | 17.92 | 2.49 | 0.00 | 186.17 | 47.84 | 37.09 | 17.28 | 15.94 | 1.27 | 0.95 | 0.45 | 3.60 | 0.36 | 0.56 | 0.01 |
| Fusui County | 408921 | 1.0000 | 0.0800 | 0.0533 | 0.0000 | 0.0000 | 1.1333 | 143.88 | 143.88 | 11.51 | 7.67 | 0.00 | 0.00 | 163.06 | 29.64 | 22.98 | 10.71 | 9.88 | 0.79 | 0.59 | 0.28 | 2.23 | 0.22 | 0.35 | 0.01 |
| Ningming County | 319386 | 0.4400 | 0.0813 | 0.0120 | 0.0000 | 0.0000 | 0.5333 | 85.97 | 37.82 | 6.99 | 1.03 | 0.00 | 0.00 | 45.85 | 8.39 | 6.51 | 3.03 | 2.80 | 0.22 | 0.17 | 0.08 | 0.63 | 0.06 | 0.10 | 0.00 |
| Longzhou County | 232068 | 0.0416 | 0.0529 | 0.0325 | 0.0000 | 0.0000 | 0.1270 | 99.97 | 4.16 | 5.29 | 3.25 | 0.00 | 0.00 | 12.69 | 4.16 | 3.22 | 1.50 | 1.39 | 0.11 | 0.08 | 0.04 | 0.31 | 0.03 | 0.05 | 0.00 |
| Daxin County | 282563 | 0.1293 | 0.0620 | 0.0013 | 0.0000 | 0.0000 | 0.1927 | 102.75 | 13.29 | 6.37 | 0.14 | 0.00 | 0.00 | 19.80 | 3.99 | 3.09 | 1.44 | 1.33 | 0.11 | 0.08 | 0.04 | 0.30 | 0.03 | 0.05 | 0.00 |
| Tiandeng County | 280473 | 0.0000 | 0.0000 | 0.0000 | 0.0000 | 0.0000 | 0.0000 | 129.54 | 0.00 | 0.00 | 0.00 | 0.00 | 0.00 | 0.00 | 0.00 | 0.00 | 0.00 | 0.00 | 0.00 | 0.00 | 0.00 | 0.00 | 0.00 | 0.00 | 0.00 |
| Pingxiang City | 129843 | 0.6667 | 0.1333 | 0.0000 | 0.0000 | 0.0000 | 0.8000 | 202.27 | 134.84 | 26.97 | 0.00 | 0.00 | 0.00 | 161.81 | 28.32 | 21.96 | 10.23 | 9.44 | 0.75 | 0.56 | 0.27 | 2.13 | 0.21 | 0.33 | 0.01 |
| **Hainan** | 10081232 | 117.7021 | 47.4973 | 21.4620 | 10.6500 | 0.7747 | 198.0861 | 731.57 | 86107.78 | 34747.80 | 15701.03 | 7791.26 | 566.73 | 144914.60 | 39504.19 | 30631.55 | 14268.91 | 13166.75 | 1046.86 | 782.18 | 371.34 | 2970.72 | 296.28 | 462.20 | 11.85 |
| **Haikou City** | 2873358 | 14.2053 | 0.0000 | 0.0000 | 0.0000 | 0.0000 | 14.2053 | 1284.89 | 18252.35 | 0.00 | 0.00 | 0.00 | 0.00 | 18252.35 | 2737.85 | 2122.93 | 988.91 | 912.53 | 72.55 | 54.21 | 25.74 | 205.89 | 20.53 | 32.03 | 0.82 |
| Xiuying District | 567108 | 4.1860 | 0.0000 | 0.0000 | 0.0000 | 0.0000 | 4.1860 | 1145.19 | 4793.75 | 0.00 | 0.00 | 0.00 | 0.00 | 4793.75 | 719.06 | 557.56 | 259.73 | 239.66 | 19.06 | 14.24 | 6.76 | 54.07 | 5.39 | 8.41 | 0.22 |
| Longhua District | 797684 | 2.7213 | 0.0000 | 0.0000 | 0.0000 | 0.0000 | 2.7213 | 2656.38 | 7228.89 | 0.00 | 0.00 | 0.00 | 0.00 | 7228.89 | 1084.33 | 840.79 | 391.66 | 361.41 | 28.73 | 21.47 | 10.19 | 81.54 | 8.13 | 12.69 | 0.33 |
| Qiongshan District | 655553 | 4.5093 | 0.0000 | 0.0000 | 0.0000 | 0.0000 | 4.5093 | 704.95 | 3178.85 | 0.00 | 0.00 | 0.00 | 0.00 | 3178.85 | 476.83 | 369.73 | 172.23 | 158.93 | 12.64 | 9.44 | 4.48 | 35.86 | 3.58 | 5.58 | 0.14 |
| Meilan District | 853013 | 2.7887 | 0.0000 | 0.0000 | 0.0000 | 0.0000 | 2.7887 | 1669.86 | 4656.67 | 0.00 | 0.00 | 0.00 | 0.00 | 4656.67 | 698.50 | 541.62 | 252.30 | 232.81 | 18.51 | 13.83 | 6.57 | 52.53 | 5.24 | 8.17 | 0.21 |
| **Sanya City** | 1031396 | 4.4795 | 0.0000 | 0.0000 | 0.0000 | 0.0000 | 4.4795 | 542.03 | 2428.00 | 0.00 | 0.00 | 0.00 | 0.00 | 2428.00 | 364.20 | 282.40 | 131.55 | 121.39 | 9.65 | 7.21 | 3.42 | 27.39 | 2.73 | 4.26 | 0.11 |
| Haitang District | 113481 | 2.3795 | 0.0000 | 0.0000 | 0.0000 | 0.0000 | 2.3795 | 456.43 | 1086.08 | 0.00 | 0.00 | 0.00 | 0.00 | 1086.08 | 162.91 | 126.32 | 58.84 | 54.30 | 4.32 | 3.23 | 1.53 | 12.25 | 1.22 | 1.91 | 0.05 |
| Jiyang District | 447322 | 1.3363 | 0.0000 | 0.0000 | 0.0000 | 0.0000 | 1.3363 | 1219.66 | 1629.85 | 0.00 | 0.00 | 0.00 | 0.00 | 1629.85 | 244.48 | 189.57 | 88.31 | 81.48 | 6.48 | 4.84 | 2.30 | 18.38 | 1.83 | 2.86 | 0.07 |
| Tianya District | 353698 | 0.2717 | 0.0000 | 0.0000 | 0.0000 | 0.0000 | 0.2717 | 375.62 | 102.07 | 0.00 | 0.00 | 0.00 | 0.00 | 102.07 | 15.31 | 11.87 | 5.53 | 5.10 | 0.41 | 0.30 | 0.14 | 1.15 | 0.11 | 0.18 | 0.00 |
| Yazhou District | 116895 | 0.4919 | 0.0000 | 0.0000 | 0.0000 | 0.0000 | 0.4919 | 338.03 | 166.27 | 0.00 | 0.00 | 0.00 | 0.00 | 166.27 | 24.94 | 19.34 | 9.01 | 8.31 | 0.66 | 0.49 | 0.23 | 1.88 | 0.19 | 0.29 | 0.01 |
| **Sansha City** | 2333 | 0.0000 | 0.0000 | 0.0000 | 0.0000 | 0.0000 | 0.0000 | 0.37 | 0.00 | 0.00 | 0.00 | 0.00 | 0.00 | 0.00 | 0.00 | 0.00 | 0.00 | 0.00 | 0.00 | 0.00 | 0.00 | 0.00 | 0.00 | 0.00 | 0.00 |
| Sansha City | 2333 | 0.0000 | 0.0000 | 0.0000 | 0.0000 | 0.0000 | 0.0000 | 0.37 | 0.00 | 0.00 | 0.00 | 0.00 | 0.00 | 0.00 | 0.00 | 0.00 | 0.00 | 0.00 | 0.00 | 0.00 | 0.00 | 0.00 | 0.00 | 0.00 | 0.00 |
| **Administrative divisions at the county level directly under the provincial government** | 6174145 | 99.0173 | 47.4973 | 21.4620 | 10.6500 | 0.7747 | 179.4013 | 207.11 | 20507.92 | 9837.39 | 4445.09 | 2205.77 | 160.44 | 37156.62 | 10603.47 | 8221.93 | 3829.98 | 3534.14 | 280.99 | 209.95 | 99.67 | 797.38 | 79.53 | 124.06 | 3.18 |
| Danzhou City | 954259 | 15.7067 | 1.4733 | 1.2333 | 0.3787 | 0.0000 | 18.7920 | 292.05 | 4587.06 | 430.28 | 360.19 | 110.59 | 0.00 | 5488.12 | 1121.73 | 869.79 | 405.17 | 373.87 | 29.73 | 22.21 | 10.54 | 84.35 | 8.41 | 13.12 | 0.34 |
| Wuzhishan Market | 112269 | 0.0000 | 1.2333 | 0.0000 | 0.0000 | 0.0000 | 1.2333 | 98.15 | 0.00 | 121.06 | 0.00 | 0.00 | 0.00 | 121.06 | 36.32 | 28.16 | 13.12 | 12.10 | 0.96 | 0.72 | 0.34 | 2.73 | 0.27 | 0.42 | 0.01 |
| Qionghai City | 528238 | 6.0453 | 6.2033 | 0.9900 | 0.3933 | 0.0000 | 13.6320 | 312.99 | 1892.15 | 1941.60 | 309.86 | 123.11 | 0.00 | 4266.72 | 1150.71 | 892.26 | 415.64 | 383.53 | 30.49 | 22.78 | 10.82 | 86.53 | 8.63 | 13.46 | 0.35 |
| Wenchang City | 560894 | 20.3733 | 7.9667 | 0.9600 | 0.3800 | 0.0000 | 29.6800 | 236.97 | 4827.95 | 1887.89 | 227.50 | 90.05 | 0.00 | 7033.39 | 1499.10 | 1162.40 | 541.47 | 499.65 | 39.73 | 29.68 | 14.09 | 112.73 | 11.24 | 17.54 | 0.45 |
| Wanning City | 545992 | 13.9200 | 4.8667 | 2.3067 | 1.0000 | 0.0000 | 22.0933 | 289.70 | 4032.58 | 1409.86 | 668.23 | 289.70 | 0.00 | 6400.37 | 1660.54 | 1287.59 | 599.79 | 553.46 | 44.00 | 32.88 | 15.61 | 124.87 | 12.45 | 19.43 | 0.50 |
| Dongfang City | 444458 | 0.0000 | 0.0000 | 0.0000 | 0.0000 | 0.0000 | 0.0000 | 196.49 | 0.00 | 0.00 | 0.00 | 0.00 | 0.00 | 0.00 | 0.00 | 0.00 | 0.00 | 0.00 | 0.00 | 0.00 | 0.00 | 0.00 | 0.00 | 0.00 | 0.00 |
| Ding'an County | 284690 | 6.5667 | 1.5333 | 0.6267 | 0.0000 | 0.0000 | 8.7267 | 237.92 | 1562.31 | 364.80 | 149.09 | 0.00 | 0.00 | 2076.21 | 433.24 | 335.94 | 156.49 | 144.40 | 11.48 | 8.58 | 4.07 | 32.58 | 3.25 | 5.07 | 0.13 |
| Tunchang County | 255335 | 4.8107 | 0.8520 | 0.1900 | 0.0433 | 0.0000 | 5.8960 | 208.69 | 1003.95 | 177.81 | 39.65 | 9.04 | 0.00 | 1230.45 | 234.96 | 182.19 | 84.87 | 78.31 | 6.23 | 4.65 | 2.21 | 17.67 | 1.76 | 2.75 | 0.07 |
| Chengmai County | 497953 | 12.5040 | 4.2487 | 1.1313 | 0.4513 | 0.0947 | 18.4300 | 242.42 | 3031.21 | 1029.96 | 274.26 | 109.41 | 22.95 | 4467.78 | 1036.41 | 803.63 | 374.35 | 345.43 | 27.46 | 20.52 | 9.74 | 77.94 | 7.77 | 12.13 | 0.31 |
| Lingao County | 420594 | 11.7333 | 10.7333 | 6.8000 | 7.0000 | 0.6800 | 36.9467 | 323.09 | 3790.88 | 3467.80 | 2196.99 | 2261.61 | 219.70 | 11936.97 | 4934.18 | 3825.96 | 1782.22 | 1644.56 | 130.76 | 97.70 | 46.38 | 371.05 | 37.01 | 57.73 | 1.48 |
| Baisha Li Autonomous County | 164699 | 2.8840 | 1.5533 | 1.1773 | 0.0433 | 0.0000 | 5.6580 | 77.78 | 224.31 | 120.81 | 91.57 | 3.37 | 0.00 | 440.06 | 127.53 | 98.88 | 46.06 | 42.50 | 3.38 | 2.53 | 1.20 | 9.59 | 0.96 | 1.49 | 0.04 |
| Changjiang Li Autonomous County | 232124 | 0.1400 | 0.0667 | 0.0333 | 0.0000 | 0.0000 | 0.2400 | 145.58 | 20.38 | 9.71 | 4.85 | 0.00 | 0.00 | 34.94 | 8.88 | 6.89 | 3.21 | 2.96 | 0.24 | 0.18 | 0.08 | 0.67 | 0.07 | 0.10 | 0.00 |
| Ledong Li Autonomous County | 464435 | 0.0000 | 0.2000 | 0.0000 | 0.0000 | 0.0000 | 0.2000 | 168.19 | 0.00 | 33.64 | 0.00 | 0.00 | 0.00 | 33.64 | 10.09 | 7.83 | 3.65 | 3.36 | 0.27 | 0.20 | 0.09 | 0.76 | 0.08 | 0.12 | 0.00 |
| Lingshui Li Autonomous County | 372511 | 3.7333 | 3.0733 | 0.4800 | 0.1467 | 0.0000 | 7.4333 | 341.82 | 1276.11 | 1050.51 | 164.07 | 50.13 | 0.00 | 2540.83 | 645.12 | 500.23 | 233.02 | 215.02 | 17.10 | 12.77 | 6.06 | 48.51 | 4.84 | 7.55 | 0.19 |
| Baoting Li and Miao Autonomous County | 156108 | 0.1000 | 3.3133 | 5.3667 | 0.8133 | 0.0000 | 9.5933 | 135.39 | 13.54 | 448.60 | 726.61 | 110.12 | 0.00 | 1298.87 | 660.67 | 512.29 | 238.63 | 220.20 | 17.51 | 13.08 | 6.21 | 49.68 | 4.96 | 7.73 | 0.20 |
| Qiongzhong Li and Miao Autonomous County | 179586 | 0.5000 | 0.1800 | 0.1667 | 0.0000 | 0.0000 | 0.8467 | 66.38 | 33.19 | 11.95 | 11.06 | 0.00 | 0.00 | 56.20 | 15.20 | 11.79 | 5.49 | 5.07 | 0.40 | 0.30 | 0.14 | 1.14 | 0.11 | 0.18 | 0.00 |
| **Chongqing** | 32054159 | 5.6349 | 2.2655 | 1.9560 | 0.1413 | 0.1333 | 10.1312 | 376.56 | 2121.92 | 853.12 | 736.58 | 53.21 | 50.21 | 3815.03 | 1103.93 | 855.98 | 398.74 | 367.94 | 29.25 | 21.86 | 10.38 | 83.02 | 8.28 | 12.92 | 0.33 |
| **Municipal districts** | 25379469 | 4.9223 | 1.9942 | 1.6894 | 0.1413 | 0.1333 | 8.6273 | 586.88 | 2888.79 | 1170.36 | 991.47 | 82.93 | 78.25 | 5063.20 | 1516.08 | 1175.57 | 547.61 | 505.31 | 40.18 | 30.02 | 14.25 | 114.01 | 11.37 | 17.74 | 0.45 |
| Wanzhou District | 1564449 | 0.0000 | 0.2104 | 0.1509 | 0.0000 | 0.0000 | 0.3613 | 452.44 | 0.00 | 95.18 | 68.27 | 0.00 | 0.00 | 163.45 | 69.52 | 53.90 | 25.11 | 23.17 | 1.84 | 1.38 | 0.65 | 5.23 | 0.52 | 0.81 | 0.02 |
| Fuling District | 1115016 | 0.0000 | 0.0000 | 0.0200 | 0.0000 | 0.0000 | 0.0200 | 378.77 | 0.00 | 0.00 | 7.58 | 0.00 | 0.00 | 7.58 | 4.55 | 3.52 | 1.64 | 1.51 | 0.12 | 0.09 | 0.04 | 0.34 | 0.03 | 0.05 | 0.00 |
| Yuzhong District | 588717 | 0.0000 | 0.0000 | 0.0000 | 0.0000 | 0.0000 | 0.0000 | 24673.81 | 0.00 | 0.00 | 0.00 | 0.00 | 0.00 | 0.00 | 0.00 | 0.00 | 0.00 | 0.00 | 0.00 | 0.00 | 0.00 | 0.00 | 0.00 | 0.00 | 0.00 |
| Dadukou District | 421904 | 0.0000 | 0.0000 | 0.0000 | 0.0000 | 0.0000 | 0.0000 | 4125.80 | 0.00 | 0.00 | 0.00 | 0.00 | 0.00 | 0.00 | 0.00 | 0.00 | 0.00 | 0.00 | 0.00 | 0.00 | 0.00 | 0.00 | 0.00 | 0.00 | 0.00 |
| Jiangbei District | 925800 | 0.0067 | 0.0000 | 0.0000 | 0.0000 | 0.0000 | 0.0067 | 4187.81 | 27.92 | 0.00 | 0.00 | 0.00 | 0.00 | 27.92 | 4.19 | 3.25 | 1.51 | 1.40 | 0.11 | 0.08 | 0.04 | 0.31 | 0.03 | 0.05 | 0.00 |
| Shapingba District | 1477345 | 2.0316 | 0.0000 | 0.0000 | 0.0000 | 0.0000 | 2.0316 | 3715.00 | 7547.47 | 0.00 | 0.00 | 0.00 | 0.00 | 7547.47 | 1132.12 | 877.85 | 408.92 | 377.34 | 30.00 | 22.42 | 10.64 | 85.14 | 8.49 | 13.25 | 0.34 |
| Jiulongpo District | 1526821 | 0.1001 | 0.0033 | 0.0000 | 0.0005 | 0.0000 | 0.1040 | 3537.34 | 354.21 | 11.79 | 0.00 | 1.89 | 0.00 | 367.88 | 58.18 | 45.11 | 21.01 | 19.39 | 1.54 | 1.15 | 0.55 | 4.37 | 0.44 | 0.68 | 0.02 |
| Nan'an District | 1197639 | 0.0267 | 0.0000 | 0.0000 | 0.0000 | 0.0000 | 0.0267 | 4571.49 | 121.91 | 0.00 | 0.00 | 0.00 | 0.00 | 121.91 | 18.29 | 14.18 | 6.60 | 6.09 | 0.48 | 0.36 | 0.17 | 1.38 | 0.14 | 0.21 | 0.01 |
| Beibei District | 834887 | 0.0000 | 0.0000 | 0.0000 | 0.0000 | 0.0000 | 0.0000 | 1113.23 | 0.00 | 0.00 | 0.00 | 0.00 | 0.00 | 0.00 | 0.00 | 0.00 | 0.00 | 0.00 | 0.00 | 0.00 | 0.00 | 0.00 | 0.00 | 0.00 | 0.00 |
| Qijiang District | 1011334 | 0.0172 | 0.0893 | 0.1467 | 0.0000 | 0.0000 | 0.0000 | 367.81 | 6.33 | 32.86 | 53.95 | 0.00 | 0.00 | 0.00 | 43.17 | 33.48 | 15.59 | 14.39 | 1.14 | 0.85 | 0.41 | 3.25 | 0.32 | 0.51 | 0.01 |
| Dazu area | 834592 | 0.0007 | 0.0000 | 0.1980 | 0.0000 | 0.0000 | 0.1987 | 581.31 | 0.39 | 0.00 | 115.10 | 0.00 | 0.00 | 115.49 | 69.12 | 53.59 | 24.97 | 23.04 | 1.83 | 1.37 | 0.65 | 5.20 | 0.52 | 0.81 | 0.02 |
| Yubei District | 2191493 | 2.1057 | 0.2380 | 0.1587 | 0.0000 | 0.1333 | 2.6357 | 1503.80 | 3166.51 | 357.91 | 238.60 | 0.00 | 200.51 | 3963.53 | 905.97 | 702.49 | 327.24 | 301.96 | 24.01 | 17.94 | 8.52 | 68.13 | 6.79 | 10.60 | 0.27 |
| Banan District | 1178856 | 0.0333 | 0.0033 | 0.0027 | 0.0000 | 0.0000 | 0.0393 | 647.30 | 21.58 | 2.16 | 1.73 | 0.00 | 0.00 | 25.46 | 4.92 | 3.81 | 1.78 | 1.64 | 0.13 | 0.10 | 0.05 | 0.37 | 0.04 | 0.06 | 0.00 |
| Qianjiang District | 487281 | 0.0000 | 0.0000 | 0.0000 | 0.0000 | 0.0000 | 0.0000 | 203.80 | 0.00 | 0.00 | 0.00 | 0.00 | 0.00 | 0.00 | 0.00 | 0.00 | 0.00 | 0.00 | 0.00 | 0.00 | 0.00 | 0.00 | 0.00 | 0.00 | 0.00 |
| Longevity area | 692960 | 0.0000 | 0.0000 | 0.4667 | 0.0000 | 0.0000 | 0.4667 | 488.17 | 0.00 | 0.00 | 227.81 | 0.00 | 0.00 | 227.81 | 136.69 | 105.99 | 49.37 | 45.56 | 3.62 | 2.71 | 1.28 | 10.28 | 1.03 | 1.60 | 0.04 |
| Jiangjin District | 1359611 | 0.2459 | 1.3105 | 0.4592 | 0.1274 | 0.0000 | 2.1429 | 422.61 | 103.90 | 553.82 | 194.04 | 53.86 | 0.00 | 905.62 | 341.24 | 264.60 | 123.26 | 113.74 | 9.04 | 6.76 | 3.21 | 25.66 | 2.56 | 3.99 | 0.10 |
| Hechuan District | 1245294 | 0.1253 | 0.0167 | 0.0000 | 0.0000 | 0.0000 | 0.1420 | 530.91 | 66.54 | 8.85 | 0.00 | 0.00 | 0.00 | 75.39 | 12.64 | 9.80 | 4.56 | 4.21 | 0.33 | 0.25 | 0.12 | 0.95 | 0.09 | 0.15 | 0.00 |
| Yongchuan District | 1148896 | 0.0068 | 0.0000 | 0.0000 | 0.0000 | 0.0000 | 0.0068 | 729.97 | 4.96 | 0.00 | 0.00 | 0.00 | 0.00 | 4.96 | 0.74 | 0.58 | 0.27 | 0.25 | 0.02 | 0.01 | 0.01 | 0.06 | 0.01 | 0.01 | 0.00 |
| Nanchuan District | 572362 | 0.0000 | 0.0000 | 0.0000 | 0.0000 | 0.0000 | 0.0000 | 221.31 | 0.00 | 0.00 | 0.00 | 0.00 | 0.00 | 0.00 | 0.00 | 0.00 | 0.00 | 0.00 | 0.00 | 0.00 | 0.00 | 0.00 | 0.00 | 0.00 | 0.00 |
| Bishan District | 756022 | 0.0047 | 0.0000 | 0.0000 | 0.0000 | 0.0000 | 0.0047 | 827.23 | 3.86 | 0.00 | 0.00 | 0.00 | 0.00 | 3.86 | 0.58 | 0.45 | 0.21 | 0.19 | 0.02 | 0.01 | 0.01 | 0.04 | 0.00 | 0.01 | 0.00 |
| Tongliang District | 685729 | 0.2133 | 0.0667 | 0.0867 | 0.0133 | 0.0000 | 0.3800 | 512.16 | 109.26 | 34.14 | 44.39 | 6.83 | 0.00 | 194.62 | 58.73 | 45.54 | 21.21 | 19.57 | 1.56 | 1.16 | 0.55 | 4.42 | 0.44 | 0.69 | 0.02 |
| Tongnan District | 688115 | 0.0017 | 0.0000 | 0.0000 | 0.0000 | 0.0000 | 0.0017 | 433.51 | 0.72 | 0.00 | 0.00 | 0.00 | 0.00 | 0.72 | 0.11 | 0.08 | 0.04 | 0.04 | 0.00 | 0.00 | 0.00 | 0.01 | 0.00 | 0.00 | 0.00 |
| Rongchang District | 668977 | 0.0000 | 0.0560 | 0.0000 | 0.0000 | 0.0000 | 0.0560 | 622.94 | 0.00 | 34.88 | 0.00 | 0.00 | 0.00 | 34.88 | 10.47 | 8.11 | 3.78 | 3.49 | 0.28 | 0.21 | 0.10 | 0.79 | 0.08 | 0.12 | 0.00 |
| Kaizhou District | 1203306 | 0.0027 | 0.0000 | 0.0000 | 0.0000 | 0.0000 | 0.0027 | 303.65 | 0.81 | 0.00 | 0.00 | 0.00 | 0.00 | 0.81 | 0.12 | 0.09 | 0.04 | 0.04 | 0.00 | 0.00 | 0.00 | 0.01 | 0.00 | 0.00 | 0.00 |
| Liangping District | 645315 | 0.0000 | 0.0000 | 0.0000 | 0.0000 | 0.0000 | 0.0000 | 341.49 | 0.00 | 0.00 | 0.00 | 0.00 | 0.00 | 0.00 | 0.00 | 0.00 | 0.00 | 0.00 | 0.00 | 0.00 | 0.00 | 0.00 | 0.00 | 0.00 | 0.00 |
| Wulong District | 356748 | 0.0000 | 0.0000 | 0.0000 | 0.0000 | 0.0000 | 0.0000 | 123.41 | 0.00 | 0.00 | 0.00 | 0.00 | 0.00 | 0.00 | 0.00 | 0.00 | 0.00 | 0.00 | 0.00 | 0.00 | 0.00 | 0.00 | 0.00 | 0.00 | 0.00 |
| **County** | 6674690 | 0.7127 | 0.2713 | 0.2667 | 0.0000 | 0.0000 | 1.5039 | 159.38 | 113.59 | 43.25 | 42.50 | 0.00 | 0.00 | 239.69 | 55.51 | 43.05 | 20.05 | 18.50 | 1.47 | 1.10 | 0.52 | 4.17 | 0.42 | 0.65 | 0.02 |
| Chengkou County | 197497 | 0.0000 | 0.0000 | 0.0000 | 0.0000 | 0.0000 | 0.0000 | 60.03 | 0.00 | 0.00 | 0.00 | 0.00 | 0.00 | 0.00 | 0.00 | 0.00 | 0.00 | 0.00 | 0.00 | 0.00 | 0.00 | 0.00 | 0.00 | 0.00 | 0.00 |
| Fengdu County | 557374 | 0.0440 | 0.0000 | 0.2667 | 0.0000 | 0.0000 | 0.3107 | 191.96 | 8.45 | 0.00 | 51.19 | 0.00 | 0.00 | 59.64 | 31.98 | 24.80 | 11.55 | 10.66 | 0.85 | 0.63 | 0.30 | 2.40 | 0.24 | 0.37 | 0.01 |
| Dianjiang County | 650694 | 0.0000 | 0.0000 | 0.0000 | 0.0000 | 0.0000 | 0.0000 | 429.30 | 0.00 | 0.00 | 0.00 | 0.00 | 0.00 | 0.00 | 0.00 | 0.00 | 0.00 | 0.00 | 0.00 | 0.00 | 0.00 | 0.00 | 0.00 | 0.00 | 0.00 |
| Zhong County | 720976 | 0.0000 | 0.0000 | 0.0000 | 0.0000 | 0.0000 | 0.0000 | 330.53 | 0.00 | 0.00 | 0.00 | 0.00 | 0.00 | 0.00 | 0.00 | 0.00 | 0.00 | 0.00 | 0.00 | 0.00 | 0.00 | 0.00 | 0.00 | 0.00 | 0.00 |
| Yunyang County | 929034 | 0.0000 | 0.0000 | 0.0000 | 0.0000 | 0.0000 | 0.0000 | 255.38 | 0.00 | 0.00 | 0.00 | 0.00 | 0.00 | 0.00 | 0.00 | 0.00 | 0.00 | 0.00 | 0.00 | 0.00 | 0.00 | 0.00 | 0.00 | 0.00 | 0.00 |
| Fengjie County | 744836 | 0.0000 | 0.0000 | 0.0000 | 0.0000 | 0.0000 | 0.0000 | 181.75 | 0.00 | 0.00 | 0.00 | 0.00 | 0.00 | 0.00 | 0.00 | 0.00 | 0.00 | 0.00 | 0.00 | 0.00 | 0.00 | 0.00 | 0.00 | 0.00 | 0.00 |
| Wushan County | 462462 | 0.0000 | 0.0000 | 0.0000 | 0.0000 | 0.0000 | 0.0000 | 156.68 | 0.00 | 0.00 | 0.00 | 0.00 | 0.00 | 0.00 | 0.00 | 0.00 | 0.00 | 0.00 | 0.00 | 0.00 | 0.00 | 0.00 | 0.00 | 0.00 | 0.00 |
| Wuxi County | 388685 | 0.0000 | 0.0000 | 0.0000 | 0.0000 | 0.0000 | 0.0000 | 96.70 | 0.00 | 0.00 | 0.00 | 0.00 | 0.00 | 0.00 | 0.00 | 0.00 | 0.00 | 0.00 | 0.00 | 0.00 | 0.00 | 0.00 | 0.00 | 0.00 | 0.00 |
| Shizhu Tujia Autonomous County | 389001 | 0.6667 | 0.2713 | 0.0000 | 0.0000 | 0.0000 | 0.9380 | 129.00 | 86.00 | 35.00 | 0.00 | 0.00 | 0.00 | 121.01 | 23.40 | 18.15 | 8.45 | 7.80 | 0.62 | 0.46 | 0.22 | 1.76 | 0.18 | 0.27 | 0.01 |
| Xiushan Tujia and Miao Autonomous County | 496194 | 0.0000 | 0.0000 | 0.0000 | 0.0000 | 0.0000 | 0.0000 | 202.29 | 0.00 | 0.00 | 0.00 | 0.00 | 0.00 | 0.00 | 0.00 | 0.00 | 0.00 | 0.00 | 0.00 | 0.00 | 0.00 | 0.00 | 0.00 | 0.00 | 0.00 |
| Youyang Tujia and Miao Autonomous County | 607338 | 0.0000 | 0.0000 | 0.0000 | 0.0000 | 0.0000 | 0.0000 | 117.53 | 0.00 | 0.00 | 0.00 | 0.00 | 0.00 | 0.00 | 0.00 | 0.00 | 0.00 | 0.00 | 0.00 | 0.00 | 0.00 | 0.00 | 0.00 | 0.00 | 0.00 |
| Pengshui Miao Tujia Autonomous County | 530599 | 0.0000 | 0.0000 | 0.0000 | 0.0000 | 0.0000 | 0.0000 | 136.25 | 0.00 | 0.00 | 0.00 | 0.00 | 0.00 | 0.00 | 0.00 | 0.00 | 0.00 | 0.00 | 0.00 | 0.00 | 0.00 | 0.00 | 0.00 | 0.00 | 0.00 |
| Qijiang District (Wanshengjingkai District ) | 117.55 | 0.0020 | 0.0000 | 0.0000 | 0.0000 | 0.0000 | 0.2552 | 0.04 | 0.00 | 0.00 | 0.00 | 0.00 | 0.00 | 0.01 | 0.00 | 0.00 | 0.00 | 0.00 | 0.00 | 0.00 | 0.00 | 0.00 | 0.00 | 0.00 | 0.00 |
| **Sichuan Province** | 83674866 | 127.9042 | 66.0942 | 30.1292 | 6.8853 | 1.3780 | 232.3909 | 172.12 | 22014.63 | 11376.00 | 5185.78 | 1185.09 | 237.18 | 39998.68 | 10987.99 | 8520.09 | 3968.86 | 3662.30 | 291.18 | 217.56 | 103.29 | 826.30 | 82.41 | 128.56 | 3.30 |
| **Chengdu City** | 20937757 | 0.4680 | 0.1480 | 0.0624 | 0.0240 | 0.0000 | 0.7024 | 1460.81 | 683.66 | 216.20 | 91.15 | 35.06 | 0.00 | 1026.07 | 250.15 | 193.97 | 90.35 | 83.37 | 6.63 | 4.95 | 2.35 | 18.81 | 1.88 | 2.93 | 0.08 |
| Jinjiang District | 902933 | 0.0000 | 0.0000 | 0.0020 | 0.0000 | 0.0000 | 0.0020 | 14946.75 | 0.00 | 0.00 | 29.89 | 0.00 | 0.00 | 29.89 | 17.94 | 13.91 | 6.48 | 5.98 | 0.48 | 0.36 | 0.17 | 1.35 | 0.13 | 0.21 | 0.01 |
| Qingyang District | 955954 | 0.0000 | 0.0000 | 0.0000 | 0.0000 | 0.0000 | 0.0000 | 15047.28 | 0.00 | 0.00 | 0.00 | 0.00 | 0.00 | 0.00 | 0.00 | 0.00 | 0.00 | 0.00 | 0.00 | 0.00 | 0.00 | 0.00 | 0.00 | 0.00 | 0.00 |
| Taurus District | 1265398 | 0.0000 | 0.0000 | 0.0000 | 0.0000 | 0.0000 | 0.0000 | 11854.96 | 0.00 | 0.00 | 0.00 | 0.00 | 0.00 | 0.00 | 0.00 | 0.00 | 0.00 | 0.00 | 0.00 | 0.00 | 0.00 | 0.00 | 0.00 | 0.00 | 0.00 |
| Wuhou District | 1855186 | 0.0000 | 0.0000 | 0.0004 | 0.0000 | 0.0000 | 0.0004 | 14992.61 | 0.00 | 0.00 | 6.00 | 0.00 | 0.00 | 6.00 | 3.60 | 2.79 | 1.30 | 1.20 | 0.10 | 0.07 | 0.03 | 0.27 | 0.03 | 0.04 | 0.00 |
| Chenghua District | 1381894 | 0.0000 | 0.0000 | 0.0000 | 0.0000 | 0.0000 | 0.0000 | 12624.65 | 0.00 | 0.00 | 0.00 | 0.00 | 0.00 | 0.00 | 0.00 | 0.00 | 0.00 | 0.00 | 0.00 | 0.00 | 0.00 | 0.00 | 0.00 | 0.00 | 0.00 |
| Longquanyi District | 1346210 | 0.0000 | 0.0000 | 0.0000 | 0.0000 | 0.0000 | 0.0000 | 2413.82 | 0.00 | 0.00 | 0.00 | 0.00 | 0.00 | 0.00 | 0.00 | 0.00 | 0.00 | 0.00 | 0.00 | 0.00 | 0.00 | 0.00 | 0.00 | 0.00 | 0.00 |
| Qingbaijiang District | 490091 | 0.0000 | 0.0000 | 0.0000 | 0.0000 | 0.0000 | 0.0000 | 1295.06 | 0.00 | 0.00 | 0.00 | 0.00 | 0.00 | 0.00 | 0.00 | 0.00 | 0.00 | 0.00 | 0.00 | 0.00 | 0.00 | 0.00 | 0.00 | 0.00 | 0.00 |
| Xindu District | 1558466 | 0.0000 | 0.0000 | 0.0000 | 0.0000 | 0.0000 | 0.0000 | 3157.86 | 0.00 | 0.00 | 0.00 | 0.00 | 0.00 | 0.00 | 0.00 | 0.00 | 0.00 | 0.00 | 0.00 | 0.00 | 0.00 | 0.00 | 0.00 | 0.00 | 0.00 |
| Wenjiang District | 967868 | 0.0000 | 0.0000 | 0.0000 | 0.0000 | 0.0000 | 0.0000 | 3499.92 | 0.00 | 0.00 | 0.00 | 0.00 | 0.00 | 0.00 | 0.00 | 0.00 | 0.00 | 0.00 | 0.00 | 0.00 | 0.00 | 0.00 | 0.00 | 0.00 | 0.00 |
| Shuangliu District | 2659829 | 0.0013 | 0.0427 | 0.0593 | 0.0240 | 0.0000 | 0.1273 | 2487.45 | 3.32 | 106.13 | 147.59 | 59.70 | 0.00 | 316.74 | 168.65 | 130.77 | 60.92 | 56.21 | 4.47 | 3.34 | 1.59 | 12.68 | 1.26 | 1.97 | 0.05 |
| Pidu District | 1672025 | 0.0000 | 0.0000 | 0.0000 | 0.0000 | 0.0000 | 0.0000 | 3803.95 | 0.00 | 0.00 | 0.00 | 0.00 | 0.00 | 0.00 | 0.00 | 0.00 | 0.00 | 0.00 | 0.00 | 0.00 | 0.00 | 0.00 | 0.00 | 0.00 | 0.00 |
| Xinjin District | 363591 | 0.0000 | 0.0920 | 0.0000 | 0.0000 | 0.0000 | 0.0920 | 1103.33 | 0.00 | 101.51 | 0.00 | 0.00 | 0.00 | 101.51 | 30.45 | 23.61 | 11.00 | 10.15 | 0.81 | 0.60 | 0.29 | 2.29 | 0.23 | 0.36 | 0.01 |
| Jintang County | 800371 | 0.0000 | 0.0133 | 0.0000 | 0.0000 | 0.0000 | 0.0133 | 692.30 | 0.00 | 9.23 | 0.00 | 0.00 | 0.00 | 9.23 | 2.77 | 2.15 | 1.00 | 0.92 | 0.07 | 0.05 | 0.03 | 0.21 | 0.02 | 0.03 | 0.00 |
| Dayi County | 515962 | 0.0000 | 0.0000 | 0.0000 | 0.0000 | 0.0000 | 0.0000 | 402.25 | 0.00 | 0.00 | 0.00 | 0.00 | 0.00 | 0.00 | 0.00 | 0.00 | 0.00 | 0.00 | 0.00 | 0.00 | 0.00 | 0.00 | 0.00 | 0.00 | 0.00 |
| Pujiang County | 255563 | 0.0000 | 0.0000 | 0.0000 | 0.0000 | 0.0000 | 0.0000 | 439.15 | 0.00 | 0.00 | 0.00 | 0.00 | 0.00 | 0.00 | 0.00 | 0.00 | 0.00 | 0.00 | 0.00 | 0.00 | 0.00 | 0.00 | 0.00 | 0.00 | 0.00 |
| Dujiangyan City | 710056 | 0.0000 | 0.0000 | 0.0000 | 0.0000 | 0.0000 | 0.0000 | 588.33 | 0.00 | 0.00 | 0.00 | 0.00 | 0.00 | 0.00 | 0.00 | 0.00 | 0.00 | 0.00 | 0.00 | 0.00 | 0.00 | 0.00 | 0.00 | 0.00 | 0.00 |
| Pengzhou City | 780399 | 0.0000 | 0.0000 | 0.0000 | 0.0000 | 0.0000 | 0.0000 | 548.88 | 0.00 | 0.00 | 0.00 | 0.00 | 0.00 | 0.00 | 0.00 | 0.00 | 0.00 | 0.00 | 0.00 | 0.00 | 0.00 | 0.00 | 0.00 | 0.00 | 0.00 |
| Qionglai City | 602973 | 0.0000 | 0.0000 | 0.0000 | 0.0000 | 0.0000 | 0.0000 | 439.23 | 0.00 | 0.00 | 0.00 | 0.00 | 0.00 | 0.00 | 0.00 | 0.00 | 0.00 | 0.00 | 0.00 | 0.00 | 0.00 | 0.00 | 0.00 | 0.00 | 0.00 |
| Chongzhou City | 735723 | 0.0000 | 0.0000 | 0.0007 | 0.0000 | 0.0000 | 0.0007 | 674.85 | 0.00 | 0.00 | 0.45 | 0.00 | 0.00 | 0.45 | 0.27 | 0.21 | 0.10 | 0.09 | 0.01 | 0.01 | 0.00 | 0.02 | 0.00 | 0.00 | 0.00 |
| Jianyang City | 1117265 | 0.4667 | 0.0000 | 0.0000 | 0.0000 | 0.0000 | 0.4667 | 505.07 | 235.70 | 0.00 | 0.00 | 0.00 | 0.00 | 235.70 | 35.35 | 27.41 | 12.77 | 11.78 | 0.94 | 0.70 | 0.33 | 2.66 | 0.27 | 0.41 | 0.01 |
| **Zigong City** | 2489256 | 0.0213 | 0.0563 | 0.0000 | 0.0000 | 0.0000 | 0.0777 | 568.12 | 12.12 | 32.00 | 0.00 | 0.00 | 0.00 | 44.12 | 11.42 | 8.85 | 4.12 | 3.81 | 0.30 | 0.23 | 0.11 | 0.86 | 0.09 | 0.13 | 0.00 |
| Ziliujing District | 481981 | 0.0000 | 0.0563 | 0.0000 | 0.0000 | 0.0000 | 0.0563 | 3097.17 | 0.00 | 174.47 | 0.00 | 0.00 | 0.00 | 174.47 | 52.34 | 40.59 | 18.91 | 17.45 | 1.39 | 1.04 | 0.49 | 3.94 | 0.39 | 0.61 | 0.02 |
| Gongjing District | 226704 | 0.0000 | 0.0000 | 0.0000 | 0.0000 | 0.0000 | 0.0000 | 559.18 | 0.00 | 0.00 | 0.00 | 0.00 | 0.00 | 0.00 | 0.00 | 0.00 | 0.00 | 0.00 | 0.00 | 0.00 | 0.00 | 0.00 | 0.00 | 0.00 | 0.00 |
| Da'an District | 291645 | 0.0000 | 0.0000 | 0.0000 | 0.0000 | 0.0000 | 0.0000 | 726.28 | 0.00 | 0.00 | 0.00 | 0.00 | 0.00 | 0.00 | 0.00 | 0.00 | 0.00 | 0.00 | 0.00 | 0.00 | 0.00 | 0.00 | 0.00 | 0.00 | 0.00 |
| Yantan District | 297365 | 0.0213 | 0.0000 | 0.0000 | 0.0000 | 0.0000 | 0.0213 | 633.66 | 13.52 | 0.00 | 0.00 | 0.00 | 0.00 | 13.52 | 2.03 | 1.57 | 0.73 | 0.68 | 0.05 | 0.04 | 0.02 | 0.15 | 0.02 | 0.02 | 0.00 |
| Rong County | 469488 | 0.0000 | 0.0000 | 0.0000 | 0.0000 | 0.0000 | 0.0000 | 292.22 | 0.00 | 0.00 | 0.00 | 0.00 | 0.00 | 0.00 | 0.00 | 0.00 | 0.00 | 0.00 | 0.00 | 0.00 | 0.00 | 0.00 | 0.00 | 0.00 | 0.00 |
| Fushun County | 722073 | 0.0000 | 0.0000 | 0.0000 | 0.0000 | 0.0000 | 0.0000 | 537.62 | 0.00 | 0.00 | 0.00 | 0.00 | 0.00 | 0.00 | 0.00 | 0.00 | 0.00 | 0.00 | 0.00 | 0.00 | 0.00 | 0.00 | 0.00 | 0.00 | 0.00 |
| **Panzhihua City** | 1212203 | 34.4480 | 21.3863 | 9.5667 | 5.4653 | 1.2847 | 72.1509 | 163.82 | 5643.32 | 3503.53 | 1567.22 | 895.34 | 210.46 | 11819.87 | 3743.57 | 2902.77 | 1352.18 | 1247.73 | 99.20 | 74.12 | 35.19 | 281.52 | 28.08 | 43.80 | 1.12 |
| Dong District | 411427 | 3.4000 | 0.2507 | 0.0000 | 0.0000 | 0.0000 | 3.6507 | 2485.51 | 8450.74 | 623.04 | 0.00 | 0.00 | 0.00 | 9073.78 | 1454.52 | 1127.84 | 525.37 | 484.79 | 38.54 | 28.80 | 13.67 | 109.38 | 10.91 | 17.02 | 0.44 |
| Xi District | 129406 | 1.8000 | 0.2667 | 0.0667 | 0.0000 | 0.0000 | 2.1333 | 1066.74 | 1920.13 | 284.46 | 71.12 | 0.00 | 0.00 | 2275.71 | 416.03 | 322.59 | 150.27 | 138.66 | 11.02 | 8.24 | 3.91 | 31.29 | 3.12 | 4.87 | 0.12 |
| Renhe District | 265562 | 7.1193 | 5.9816 | 8.2173 | 5.3267 | 1.2847 | 27.9296 | 153.55 | 1093.16 | 918.47 | 1261.76 | 817.90 | 197.26 | 4288.55 | 2028.42 | 1572.84 | 732.67 | 676.07 | 53.75 | 40.16 | 19.07 | 152.54 | 15.21 | 23.73 | 0.61 |
| Miyi County | 227011 | 8.8553 | 10.0207 | 1.1900 | 0.1387 | 0.0000 | 20.2047 | 107.60 | 952.82 | 1078.21 | 128.04 | 14.92 | 0.00 | 2173.99 | 555.15 | 430.46 | 200.52 | 185.03 | 14.71 | 10.99 | 5.22 | 41.75 | 4.16 | 6.50 | 0.17 |
| Yanbian County | 178797 | 13.2733 | 4.8667 | 0.0927 | 0.0000 | 0.0000 | 18.2327 | 54.62 | 725.01 | 265.82 | 5.06 | 0.00 | 0.00 | 995.89 | 191.53 | 148.52 | 69.18 | 63.84 | 5.08 | 3.79 | 1.80 | 14.40 | 1.44 | 2.24 | 0.06 |
| **Luzhou City** | 4254149 | 0.6208 | 0.7808 | 0.0021 | 0.0000 | 0.0000 | 1.4037 | 347.73 | 215.87 | 271.50 | 0.72 | 0.00 | 0.00 | 488.09 | 114.26 | 88.60 | 41.27 | 38.08 | 3.03 | 2.26 | 1.07 | 8.59 | 0.86 | 1.34 | 0.03 |
| Jiangyang District | 761576 | 0.4615 | 0.1555 | 0.0021 | 0.0000 | 0.0000 | 0.6190 | 1174.31 | 541.91 | 182.57 | 2.43 | 0.00 | 0.00 | 726.90 | 137.51 | 106.63 | 49.67 | 45.83 | 3.64 | 2.72 | 1.29 | 10.34 | 1.03 | 1.61 | 0.04 |
| Naxi District | 354846 | 0.0000 | 0.0000 | 0.0000 | 0.0000 | 0.0000 | 0.0000 | 308.03 | 0.00 | 0.00 | 0.00 | 0.00 | 0.00 | 0.00 | 0.00 | 0.00 | 0.00 | 0.00 | 0.00 | 0.00 | 0.00 | 0.00 | 0.00 | 0.00 | 0.00 |
| Longmatan District | 479697 | 0.0000 | 0.6253 | 0.0000 | 0.0000 | 0.0000 | 0.6253 | 1436.00 | 0.00 | 897.98 | 0.00 | 0.00 | 0.00 | 897.98 | 269.39 | 208.89 | 97.31 | 89.79 | 7.14 | 5.33 | 2.53 | 20.26 | 2.02 | 3.15 | 0.08 |
| Lu County | 764362 | 0.1593 | 0.0000 | 0.0000 | 0.0000 | 0.0000 | 0.1593 | 500.27 | 79.71 | 0.00 | 0.00 | 0.00 | 0.00 | 79.71 | 11.96 | 9.27 | 4.32 | 3.99 | 0.32 | 0.24 | 0.11 | 0.90 | 0.09 | 0.14 | 0.00 |
| Hejiang County | 688731 | 0.0000 | 0.0000 | 0.0000 | 0.0000 | 0.0000 | 0.0000 | 285.11 | 0.00 | 0.00 | 0.00 | 0.00 | 0.00 | 0.00 | 0.00 | 0.00 | 0.00 | 0.00 | 0.00 | 0.00 | 0.00 | 0.00 | 0.00 | 0.00 | 0.00 |
| Xuyong County | 552979 | 0.0000 | 0.0000 | 0.0000 | 0.0000 | 0.0000 | 0.0000 | 186.06 | 0.00 | 0.00 | 0.00 | 0.00 | 0.00 | 0.00 | 0.00 | 0.00 | 0.00 | 0.00 | 0.00 | 0.00 | 0.00 | 0.00 | 0.00 | 0.00 | 0.00 |
| Gulin County | 651958 | 0.0000 | 0.0000 | 0.0000 | 0.0000 | 0.0000 | 0.0000 | 204.76 | 0.00 | 0.00 | 0.00 | 0.00 | 0.00 | 0.00 | 0.00 | 0.00 | 0.00 | 0.00 | 0.00 | 0.00 | 0.00 | 0.00 | 0.00 | 0.00 | 0.00 |
| **Deyang City** | 3456161 | 0.0000 | 0.0000 | 0.0000 | 0.0000 | 0.0000 | 0.0000 | 584.67 | 0.00 | 0.00 | 0.00 | 0.00 | 0.00 | 0.00 | 0.00 | 0.00 | 0.00 | 0.00 | 0.00 | 0.00 | 0.00 | 0.00 | 0.00 | 0.00 | 0.00 |
| Jingyang District | 828189 | 0.0000 | 0.0000 | 0.0000 | 0.0000 | 0.0000 | 0.0000 | 1280.50 | 0.00 | 0.00 | 0.00 | 0.00 | 0.00 | 0.00 | 0.00 | 0.00 | 0.00 | 0.00 | 0.00 | 0.00 | 0.00 | 0.00 | 0.00 | 0.00 | 0.00 |
| Luojiang District | 209088 | 0.0000 | 0.0000 | 0.0000 | 0.0000 | 0.0000 | 0.0000 | 465.20 | 0.00 | 0.00 | 0.00 | 0.00 | 0.00 | 0.00 | 0.00 | 0.00 | 0.00 | 0.00 | 0.00 | 0.00 | 0.00 | 0.00 | 0.00 | 0.00 | 0.00 |
| Zhongjiang County | 946019 | 0.0000 | 0.0000 | 0.0000 | 0.0000 | 0.0000 | 0.0000 | 429.81 | 0.00 | 0.00 | 0.00 | 0.00 | 0.00 | 0.00 | 0.00 | 0.00 | 0.00 | 0.00 | 0.00 | 0.00 | 0.00 | 0.00 | 0.00 | 0.00 | 0.00 |
| Guanghan City | 626132 | 0.0000 | 0.0000 | 0.0000 | 0.0000 | 0.0000 | 0.0000 | 1143.75 | 0.00 | 0.00 | 0.00 | 0.00 | 0.00 | 0.00 | 0.00 | 0.00 | 0.00 | 0.00 | 0.00 | 0.00 | 0.00 | 0.00 | 0.00 | 0.00 | 0.00 |
| Shifang Market | 406775 | 0.0000 | 0.0000 | 0.0000 | 0.0000 | 0.0000 | 0.0000 | 495.81 | 0.00 | 0.00 | 0.00 | 0.00 | 0.00 | 0.00 | 0.00 | 0.00 | 0.00 | 0.00 | 0.00 | 0.00 | 0.00 | 0.00 | 0.00 | 0.00 | 0.00 |
| Mianzhu City | 439958 | 0.0000 | 0.0000 | 0.0000 | 0.0000 | 0.0000 | 0.0000 | 353.04 | 0.00 | 0.00 | 0.00 | 0.00 | 0.00 | 0.00 | 0.00 | 0.00 | 0.00 | 0.00 | 0.00 | 0.00 | 0.00 | 0.00 | 0.00 | 0.00 | 0.00 |
| **Mianyang City** | 4868243 | 0.2077 | 0.0333 | 0.0000 | 0.6760 | 0.0933 | 1.0104 | 240.31 | 49.92 | 8.01 | 0.00 | 162.45 | 22.43 | 242.81 | 160.03 | 124.09 | 57.80 | 53.34 | 4.24 | 3.17 | 1.50 | 12.03 | 1.20 | 1.87 | 0.05 |
| Fucheng District | 1298524 | 0.2067 | 0.0333 | 0.0000 | 0.6760 | 0.0933 | 1.0093 | 2346.02 | 484.88 | 78.20 | 0.00 | 1585.91 | 218.96 | 2367.95 | 1561.99 | 1211.16 | 564.19 | 520.61 | 41.39 | 30.93 | 14.68 | 117.46 | 11.71 | 18.28 | 0.47 |
| Youxian District | 561379 | 0.0000 | 0.0000 | 0.0000 | 0.0000 | 0.0000 | 0.0000 | 551.07 | 0.00 | 0.00 | 0.00 | 0.00 | 0.00 | 0.00 | 0.00 | 0.00 | 0.00 | 0.00 | 0.00 | 0.00 | 0.00 | 0.00 | 0.00 | 0.00 | 0.00 |
| Anzhou District | 372962 | 0.0000 | 0.0000 | 0.0000 | 0.0000 | 0.0000 | 0.0000 | 315.32 | 0.00 | 0.00 | 0.00 | 0.00 | 0.00 | 0.00 | 0.00 | 0.00 | 0.00 | 0.00 | 0.00 | 0.00 | 0.00 | 0.00 | 0.00 | 0.00 | 0.00 |
| Santai County | 955811 | 0.0000 | 0.0000 | 0.0000 | 0.0000 | 0.0000 | 0.0000 | 359.49 | 0.00 | 0.00 | 0.00 | 0.00 | 0.00 | 0.00 | 0.00 | 0.00 | 0.00 | 0.00 | 0.00 | 0.00 | 0.00 | 0.00 | 0.00 | 0.00 | 0.00 |
| Yanting County | 370739 | 0.0011 | 0.0000 | 0.0000 | 0.0000 | 0.0000 | 0.0011 | 225.15 | 0.24 | 0.00 | 0.00 | 0.00 | 0.00 | 0.24 | 0.04 | 0.03 | 0.01 | 0.01 | 0.00 | 0.00 | 0.00 | 0.00 | 0.00 | 0.00 | 0.00 |
| Zitong County | 276996 | 0.0000 | 0.0000 | 0.0000 | 0.0000 | 0.0000 | 0.0000 | 191.99 | 0.00 | 0.00 | 0.00 | 0.00 | 0.00 | 0.00 | 0.00 | 0.00 | 0.00 | 0.00 | 0.00 | 0.00 | 0.00 | 0.00 | 0.00 | 0.00 | 0.00 |
| Beichuan Qiang Autonomous County | 174132 | 0.0000 | 0.0000 | 0.0000 | 0.0000 | 0.0000 | 0.0000 | 56.43 | 0.00 | 0.00 | 0.00 | 0.00 | 0.00 | 0.00 | 0.00 | 0.00 | 0.00 | 0.00 | 0.00 | 0.00 | 0.00 | 0.00 | 0.00 | 0.00 | 0.00 |
| Pingwu County | 126357 | 0.0000 | 0.0000 | 0.0000 | 0.0000 | 0.0000 | 0.0000 | 21.24 | 0.00 | 0.00 | 0.00 | 0.00 | 0.00 | 0.00 | 0.00 | 0.00 | 0.00 | 0.00 | 0.00 | 0.00 | 0.00 | 0.00 | 0.00 | 0.00 | 0.00 |
| Jiangyou City | 731343 | 0.0000 | 0.0000 | 0.0000 | 0.0000 | 0.0000 | 0.0000 | 268.73 | 0.00 | 0.00 | 0.00 | 0.00 | 0.00 | 0.00 | 0.00 | 0.00 | 0.00 | 0.00 | 0.00 | 0.00 | 0.00 | 0.00 | 0.00 | 0.00 | 0.00 |
| **Guangyuan City** | 2305657 | 0.0113 | 0.0000 | 0.0000 | 0.0000 | 0.0000 | 0.0113 | 141.29 | 1.60 | 0.00 | 0.00 | 0.00 | 0.00 | 1.60 | 0.24 | 0.19 | 0.09 | 0.08 | 0.01 | 0.00 | 0.00 | 0.02 | 0.00 | 0.00 | 0.00 |
| Lizhou District | 621978 | 0.0113 | 0.0000 | 0.0000 | 0.0000 | 0.0000 | 0.0113 | 404.88 | 4.59 | 0.00 | 0.00 | 0.00 | 0.00 | 4.59 | 0.69 | 0.53 | 0.25 | 0.23 | 0.02 | 0.01 | 0.01 | 0.05 | 0.01 | 0.01 | 0.00 |
| Zhaohua District | 134202 | 0.0000 | 0.0000 | 0.0000 | 0.0000 | 0.0000 | 0.0000 | 93.80 | 0.00 | 0.00 | 0.00 | 0.00 | 0.00 | 0.00 | 0.00 | 0.00 | 0.00 | 0.00 | 0.00 | 0.00 | 0.00 | 0.00 | 0.00 | 0.00 | 0.00 |
| Chaotian District | 126506 | 0.0000 | 0.0000 | 0.0000 | 0.0000 | 0.0000 | 0.0000 | 78.46 | 0.00 | 0.00 | 0.00 | 0.00 | 0.00 | 0.00 | 0.00 | 0.00 | 0.00 | 0.00 | 0.00 | 0.00 | 0.00 | 0.00 | 0.00 | 0.00 | 0.00 |
| Wangcang County | 330108 | 0.0000 | 0.0000 | 0.0000 | 0.0000 | 0.0000 | 0.0000 | 110.44 | 0.00 | 0.00 | 0.00 | 0.00 | 0.00 | 0.00 | 0.00 | 0.00 | 0.00 | 0.00 | 0.00 | 0.00 | 0.00 | 0.00 | 0.00 | 0.00 | 0.00 |
| Qingchuan County | 156387 | 0.0000 | 0.0000 | 0.0000 | 0.0000 | 0.0000 | 0.0000 | 48.66 | 0.00 | 0.00 | 0.00 | 0.00 | 0.00 | 0.00 | 0.00 | 0.00 | 0.00 | 0.00 | 0.00 | 0.00 | 0.00 | 0.00 | 0.00 | 0.00 | 0.00 |
| Jiange County | 423859 | 0.0000 | 0.0000 | 0.0000 | 0.0000 | 0.0000 | 0.0000 | 132.34 | 0.00 | 0.00 | 0.00 | 0.00 | 0.00 | 0.00 | 0.00 | 0.00 | 0.00 | 0.00 | 0.00 | 0.00 | 0.00 | 0.00 | 0.00 | 0.00 | 0.00 |
| Cangxi County | 512617 | 0.0000 | 0.0000 | 0.0000 | 0.0000 | 0.0000 | 0.0000 | 219.66 | 0.00 | 0.00 | 0.00 | 0.00 | 0.00 | 0.00 | 0.00 | 0.00 | 0.00 | 0.00 | 0.00 | 0.00 | 0.00 | 0.00 | 0.00 | 0.00 | 0.00 |
| **Suining City** | 2814196 | 0.0000 | 0.0000 | 0.1733 | 0.0000 | 0.0000 | 0.1733 | 528.70 | 0.00 | 0.00 | 91.64 | 0.00 | 0.00 | 91.64 | 54.99 | 42.64 | 19.86 | 18.33 | 1.46 | 1.09 | 0.52 | 4.13 | 0.41 | 0.64 | 0.02 |
| Chuanshan District | 832863 | 0.0000 | 0.0000 | 0.0000 | 0.0000 | 0.0000 | 0.0000 | 1373.98 | 0.00 | 0.00 | 0.00 | 0.00 | 0.00 | 0.00 | 0.00 | 0.00 | 0.00 | 0.00 | 0.00 | 0.00 | 0.00 | 0.00 | 0.00 | 0.00 | 0.00 |
| An'ju Area | 431310 | 0.0000 | 0.0000 | 0.0000 | 0.0000 | 0.0000 | 0.0000 | 340.26 | 0.00 | 0.00 | 0.00 | 0.00 | 0.00 | 0.00 | 0.00 | 0.00 | 0.00 | 0.00 | 0.00 | 0.00 | 0.00 | 0.00 | 0.00 | 0.00 | 0.00 |
| Pengxi County | 430344 | 0.0000 | 0.0000 | 0.0000 | 0.0000 | 0.0000 | 0.0000 | 344.30 | 0.00 | 0.00 | 0.00 | 0.00 | 0.00 | 0.00 | 0.00 | 0.00 | 0.00 | 0.00 | 0.00 | 0.00 | 0.00 | 0.00 | 0.00 | 0.00 | 0.00 |
| Daying County | 387299 | 0.0000 | 0.0000 | 0.1733 | 0.0000 | 0.0000 | 0.1733 | 552.23 | 0.00 | 0.00 | 95.72 | 0.00 | 0.00 | 95.72 | 57.43 | 44.53 | 20.74 | 19.14 | 1.52 | 1.14 | 0.54 | 4.32 | 0.43 | 0.67 | 0.02 |
| Shehong City | 732380 | 0.0000 | 0.0000 | 0.0000 | 0.0000 | 0.0000 | 0.0000 | 488.97 | 0.00 | 0.00 | 0.00 | 0.00 | 0.00 | 0.00 | 0.00 | 0.00 | 0.00 | 0.00 | 0.00 | 0.00 | 0.00 | 0.00 | 0.00 | 0.00 | 0.00 |
| **Neijiang City** | 3140678 | 0.0000 | 0.0000 | 0.0000 | 0.0000 | 0.0000 | 0.0000 | 583.40 | 0.00 | 0.00 | 0.00 | 0.00 | 0.00 | 0.00 | 0.00 | 0.00 | 0.00 | 0.00 | 0.00 | 0.00 | 0.00 | 0.00 | 0.00 | 0.00 | 0.00 |
| Shizhong District | 425020 | 0.0000 | 0.0000 | 0.0000 | 0.0000 | 0.0000 | 0.0000 | 1098.64 | 0.00 | 0.00 | 0.00 | 0.00 | 0.00 | 0.00 | 0.00 | 0.00 | 0.00 | 0.00 | 0.00 | 0.00 | 0.00 | 0.00 | 0.00 | 0.00 | 0.00 |
| Dongxing District | 754120 | 0.0000 | 0.0000 | 0.0000 | 0.0000 | 0.0000 | 0.0000 | 638.11 | 0.00 | 0.00 | 0.00 | 0.00 | 0.00 | 0.00 | 0.00 | 0.00 | 0.00 | 0.00 | 0.00 | 0.00 | 0.00 | 0.00 | 0.00 | 0.00 | 0.00 |
| Weiyuan County | 547059 | 0.0000 | 0.0000 | 0.0000 | 0.0000 | 0.0000 | 0.0000 | 424.83 | 0.00 | 0.00 | 0.00 | 0.00 | 0.00 | 0.00 | 0.00 | 0.00 | 0.00 | 0.00 | 0.00 | 0.00 | 0.00 | 0.00 | 0.00 | 0.00 | 0.00 |
| Zizhong County | 845579 | 0.0000 | 0.0000 | 0.0000 | 0.0000 | 0.0000 | 0.0000 | 488.18 | 0.00 | 0.00 | 0.00 | 0.00 | 0.00 | 0.00 | 0.00 | 0.00 | 0.00 | 0.00 | 0.00 | 0.00 | 0.00 | 0.00 | 0.00 | 0.00 | 0.00 |
| Longchang City | 568900 | 0.0000 | 0.0000 | 0.0000 | 0.0000 | 0.0000 | 0.0000 | 715.61 | 0.00 | 0.00 | 0.00 | 0.00 | 0.00 | 0.00 | 0.00 | 0.00 | 0.00 | 0.00 | 0.00 | 0.00 | 0.00 | 0.00 | 0.00 | 0.00 | 0.00 |
| **Leshan City** | 3160168 | 1.0000 | 0.3333 | 0.0000 | 0.0000 | 0.0000 | 1.3333 | 248.42 | 248.42 | 82.81 | 0.00 | 0.00 | 0.00 | 331.22 | 62.10 | 48.16 | 22.43 | 20.70 | 1.65 | 1.23 | 0.58 | 4.67 | 0.47 | 0.73 | 0.02 |
| Shizhong District | 814597 | 0.0000 | 0.0000 | 0.0000 | 0.0000 | 0.0000 | 0.0000 | 973.78 | 0.00 | 0.00 | 0.00 | 0.00 | 0.00 | 0.00 | 0.00 | 0.00 | 0.00 | 0.00 | 0.00 | 0.00 | 0.00 | 0.00 | 0.00 | 0.00 | 0.00 |
| Shawan District | 144931 | 1.0000 | 0.3333 | 0.0000 | 0.0000 | 0.0000 | 1.3333 | 239.20 | 239.20 | 79.73 | 0.00 | 0.00 | 0.00 | 318.93 | 59.80 | 46.37 | 21.60 | 19.93 | 1.58 | 1.18 | 0.56 | 4.50 | 0.45 | 0.70 | 0.02 |
| Wutongqiao District | 237933 | 0.0000 | 0.0000 | 0.0000 | 0.0000 | 0.0000 | 0.0000 | 512.04 | 0.00 | 0.00 | 0.00 | 0.00 | 0.00 | 0.00 | 0.00 | 0.00 | 0.00 | 0.00 | 0.00 | 0.00 | 0.00 | 0.00 | 0.00 | 0.00 | 0.00 |
| Jinkouhe District | 38727 | 0.0000 | 0.0000 | 0.0000 | 0.0000 | 0.0000 | 0.0000 | 64.87 | 0.00 | 0.00 | 0.00 | 0.00 | 0.00 | 0.00 | 0.00 | 0.00 | 0.00 | 0.00 | 0.00 | 0.00 | 0.00 | 0.00 | 0.00 | 0.00 | 0.00 |
| Qianwei County | 416673 | 0.0000 | 0.0000 | 0.0000 | 0.0000 | 0.0000 | 0.0000 | 304.50 | 0.00 | 0.00 | 0.00 | 0.00 | 0.00 | 0.00 | 0.00 | 0.00 | 0.00 | 0.00 | 0.00 | 0.00 | 0.00 | 0.00 | 0.00 | 0.00 | 0.00 |
| Jingyan County | 280641 | 0.0000 | 0.0000 | 0.0000 | 0.0000 | 0.0000 | 0.0000 | 332.97 | 0.00 | 0.00 | 0.00 | 0.00 | 0.00 | 0.00 | 0.00 | 0.00 | 0.00 | 0.00 | 0.00 | 0.00 | 0.00 | 0.00 | 0.00 | 0.00 | 0.00 |
| Jiajiang County | 305441 | 0.0000 | 0.0000 | 0.0000 | 0.0000 | 0.0000 | 0.0000 | 410.22 | 0.00 | 0.00 | 0.00 | 0.00 | 0.00 | 0.00 | 0.00 | 0.00 | 0.00 | 0.00 | 0.00 | 0.00 | 0.00 | 0.00 | 0.00 | 0.00 | 0.00 |
| Muchuan County | 192313 | 0.0000 | 0.0000 | 0.0000 | 0.0000 | 0.0000 | 0.0000 | 136.55 | 0.00 | 0.00 | 0.00 | 0.00 | 0.00 | 0.00 | 0.00 | 0.00 | 0.00 | 0.00 | 0.00 | 0.00 | 0.00 | 0.00 | 0.00 | 0.00 | 0.00 |
| Ebian Yi Autonomous County | 121554 | 0.0000 | 0.0000 | 0.0000 | 0.0000 | 0.0000 | 0.0000 | 51.08 | 0.00 | 0.00 | 0.00 | 0.00 | 0.00 | 0.00 | 0.00 | 0.00 | 0.00 | 0.00 | 0.00 | 0.00 | 0.00 | 0.00 | 0.00 | 0.00 | 0.00 |
| Mabian Yi Autonomous County | 188251 | 0.0000 | 0.0000 | 0.0000 | 0.0000 | 0.0000 | 0.0000 | 82.08 | 0.00 | 0.00 | 0.00 | 0.00 | 0.00 | 0.00 | 0.00 | 0.00 | 0.00 | 0.00 | 0.00 | 0.00 | 0.00 | 0.00 | 0.00 | 0.00 | 0.00 |
| E'mei Mountain City | 419107 | 0.0000 | 0.0000 | 0.0000 | 0.0000 | 0.0000 | 0.0000 | 355.30 | 0.00 | 0.00 | 0.00 | 0.00 | 0.00 | 0.00 | 0.00 | 0.00 | 0.00 | 0.00 | 0.00 | 0.00 | 0.00 | 0.00 | 0.00 | 0.00 | 0.00 |
| **Nanchong City** | 5607565 | 0.0000 | 0.0894 | 0.0207 | 0.1867 | 0.0000 | 0.2967 | 449.25 | 0.00 | 40.14 | 9.28 | 83.86 | 0.00 | 133.29 | 84.70 | 65.68 | 30.59 | 28.23 | 2.24 | 1.68 | 0.80 | 6.37 | 0.64 | 0.99 | 0.03 |
| Shunqing District | 834294 | 0.0000 | 0.0000 | 0.0000 | 0.0000 | 0.0000 | 0.0000 | 1538.61 | 0.00 | 0.00 | 0.00 | 0.00 | 0.00 | 0.00 | 0.00 | 0.00 | 0.00 | 0.00 | 0.00 | 0.00 | 0.00 | 0.00 | 0.00 | 0.00 | 0.00 |
| Gaoping District | 570415 | 0.0000 | 0.0000 | 0.0207 | 0.0000 | 0.0000 | 0.0207 | 707.26 | 0.00 | 0.00 | 14.62 | 0.00 | 0.00 | 14.62 | 8.77 | 6.80 | 3.17 | 2.92 | 0.23 | 0.17 | 0.08 | 0.66 | 0.07 | 0.10 | 0.00 |
| Jialing District | 531825 | 0.0000 | 0.0033 | 0.0000 | 0.0000 | 0.0000 | 0.0033 | 451.39 | 0.00 | 1.50 | 0.00 | 0.00 | 0.00 | 1.50 | 0.45 | 0.35 | 0.16 | 0.15 | 0.01 | 0.01 | 0.00 | 0.03 | 0.00 | 0.01 | 0.00 |
| Nanbu County | 817235 | 0.0000 | 0.0800 | 0.0000 | 0.1867 | 0.0000 | 0.2667 | 368.84 | 0.00 | 29.51 | 0.00 | 68.85 | 0.00 | 98.36 | 63.93 | 49.57 | 23.09 | 21.31 | 1.69 | 1.27 | 0.60 | 4.81 | 0.48 | 0.75 | 0.02 |
| Yingshan County | 620480 | 0.0000 | 0.0020 | 0.0000 | 0.0000 | 0.0000 | 0.0020 | 379.52 | 0.00 | 0.76 | 0.00 | 0.00 | 0.00 | 0.76 | 0.23 | 0.18 | 0.08 | 0.08 | 0.01 | 0.00 | 0.00 | 0.02 | 0.00 | 0.00 | 0.00 |
| Peng'an County | 461485 | 0.0000 | 0.0000 | 0.0000 | 0.0000 | 0.0000 | 0.0000 | 346.20 | 0.00 | 0.00 | 0.00 | 0.00 | 0.00 | 0.00 | 0.00 | 0.00 | 0.00 | 0.00 | 0.00 | 0.00 | 0.00 | 0.00 | 0.00 | 0.00 | 0.00 |
| Yilong County | 729141 | 0.0000 | <0.0001 | 0.0000 | 0.0000 | 0.0000 | <0.0001 | 407.52 | 0.00 | 0.00 | 0.00 | 0.00 | 0.00 | #VALUE! | 0.00 | 0.00 | 0.00 | 0.00 | 0.00 | 0.00 | 0.00 | 0.00 | 0.00 | 0.00 | 0.00 |
| Xichong County | 420023 | 0.0000 | 0.0040 | 0.0000 | 0.0000 | 0.0000 | 0.0040 | 379.80 | 0.00 | 1.52 | 0.00 | 0.00 | 0.00 | 1.52 | 0.46 | 0.35 | 0.16 | 0.15 | 0.01 | 0.01 | 0.00 | 0.03 | 0.00 | 0.01 | 0.00 |
| Langzhong Market | 622667 | 0.0000 | 0.0000 | 0.0000 | 0.0000 | 0.0000 | 0.0000 | 331.82 | 0.00 | 0.00 | 0.00 | 0.00 | 0.00 | 0.00 | 0.00 | 0.00 | 0.00 | 0.00 | 0.00 | 0.00 | 0.00 | 0.00 | 0.00 | 0.00 | 0.00 |
| **Meishan City** | 2955219 | 0.0000 | 0.0000 | 0.0000 | 0.0000 | 0.0000 | 0.0000 | 414.02 | 0.00 | 0.00 | 0.00 | 0.00 | 0.00 | 0.00 | 0.00 | 0.00 | 0.00 | 0.00 | 0.00 | 0.00 | 0.00 | 0.00 | 0.00 | 0.00 | 0.00 |
| Dongpo District | 904412 | 0.0000 | 0.0000 | 0.0000 | 0.0000 | 0.0000 | 0.0000 | 680.01 | 0.00 | 0.00 | 0.00 | 0.00 | 0.00 | 0.00 | 0.00 | 0.00 | 0.00 | 0.00 | 0.00 | 0.00 | 0.00 | 0.00 | 0.00 | 0.00 | 0.00 |
| Pengshan District | 328236 | 0.0000 | 0.0000 | 0.0000 | 0.0000 | 0.0000 | 0.0000 | 701.76 | 0.00 | 0.00 | 0.00 | 0.00 | 0.00 | 0.00 | 0.00 | 0.00 | 0.00 | 0.00 | 0.00 | 0.00 | 0.00 | 0.00 | 0.00 | 0.00 | 0.00 |
| Renshou County | 1110017 | 0.0000 | 0.0000 | 0.0000 | 0.0000 | 0.0000 | 0.0000 | 425.72 | 0.00 | 0.00 | 0.00 | 0.00 | 0.00 | 0.00 | 0.00 | 0.00 | 0.00 | 0.00 | 0.00 | 0.00 | 0.00 | 0.00 | 0.00 | 0.00 | 0.00 |
| Hongya County | 295744 | 0.0000 | 0.0000 | 0.0000 | 0.0000 | 0.0000 | 0.0000 | 155.88 | 0.00 | 0.00 | 0.00 | 0.00 | 0.00 | 0.00 | 0.00 | 0.00 | 0.00 | 0.00 | 0.00 | 0.00 | 0.00 | 0.00 | 0.00 | 0.00 | 0.00 |
| Danling County | 148820 | 0.0000 | 0.0000 | 0.0000 | 0.0000 | 0.0000 | 0.0000 | 331.94 | 0.00 | 0.00 | 0.00 | 0.00 | 0.00 | 0.00 | 0.00 | 0.00 | 0.00 | 0.00 | 0.00 | 0.00 | 0.00 | 0.00 | 0.00 | 0.00 | 0.00 |
| Qingshen County | 167990 | 0.0000 | 0.0000 | 0.0000 | 0.0000 | 0.0000 | 0.0000 | 433.95 | 0.00 | 0.00 | 0.00 | 0.00 | 0.00 | 0.00 | 0.00 | 0.00 | 0.00 | 0.00 | 0.00 | 0.00 | 0.00 | 0.00 | 0.00 | 0.00 | 0.00 |
| **Yibin City** | 4588804 | 0.7586 | 0.2733 | 0.0000 | 0.0000 | 0.0000 | 1.0319 | 346.07 | 262.53 | 94.59 | 0.00 | 0.00 | 0.00 | 357.12 | 67.76 | 52.54 | 24.47 | 22.58 | 1.80 | 1.34 | 0.64 | 5.10 | 0.51 | 0.79 | 0.02 |
| Cuiping District | 887359 | 0.7586 | 0.2733 | 0.0000 | 0.0000 | 0.0000 | 1.0319 | 595.46 | 451.71 | 162.76 | 0.00 | 0.00 | 0.00 | 614.47 | 116.58 | 90.40 | 42.11 | 38.86 | 3.09 | 2.31 | 1.10 | 8.77 | 0.87 | 1.36 | 0.03 |
| Nanxi District | 332796 | 0.0000 | 0.0000 | 0.0000 | 0.0000 | 0.0000 | 0.0000 | 472.64 | 0.00 | 0.00 | 0.00 | 0.00 | 0.00 | 0.00 | 0.00 | 0.00 | 0.00 | 0.00 | 0.00 | 0.00 | 0.00 | 0.00 | 0.00 | 0.00 | 0.00 |
| Xizhou District | 938157 | <0.0001 | 0.0000 | 0.0000 | 0.0000 | 0.0000 | <0.0001 | 364.49 | 0.00 | 0.00 | 0.00 | 0.00 | 0.00 | #VALUE! | 0.00 | 0.00 | 0.00 | 0.00 | 0.00 | 0.00 | 0.00 | 0.00 | 0.00 | 0.00 | 0.00 |
| Jiang'an County | 424470 | 0.0000 | 0.0000 | 0.0000 | 0.0000 | 0.0000 | 0.0000 | 474.15 | 0.00 | 0.00 | 0.00 | 0.00 | 0.00 | 0.00 | 0.00 | 0.00 | 0.00 | 0.00 | 0.00 | 0.00 | 0.00 | 0.00 | 0.00 | 0.00 | 0.00 |
| Changning County | 327904 | 0.0000 | 0.0000 | 0.0000 | 0.0000 | 0.0000 | 0.0000 | 330.58 | 0.00 | 0.00 | 0.00 | 0.00 | 0.00 | 0.00 | 0.00 | 0.00 | 0.00 | 0.00 | 0.00 | 0.00 | 0.00 | 0.00 | 0.00 | 0.00 | 0.00 |
| Gao County | 380893 | 0.0000 | 0.0000 | 0.0000 | 0.0000 | 0.0000 | 0.0000 | 287.94 | 0.00 | 0.00 | 0.00 | 0.00 | 0.00 | 0.00 | 0.00 | 0.00 | 0.00 | 0.00 | 0.00 | 0.00 | 0.00 | 0.00 | 0.00 | 0.00 | 0.00 |
| Gong County | 339200 | 0.0000 | 0.0000 | 0.0000 | 0.0000 | 0.0000 | 0.0000 | 296.48 | 0.00 | 0.00 | 0.00 | 0.00 | 0.00 | 0.00 | 0.00 | 0.00 | 0.00 | 0.00 | 0.00 | 0.00 | 0.00 | 0.00 | 0.00 | 0.00 | 0.00 |
| Junlian County | 332805 | 0.0000 | 0.0000 | 0.0000 | 0.0000 | 0.0000 | 0.0000 | 265.27 | 0.00 | 0.00 | 0.00 | 0.00 | 0.00 | 0.00 | 0.00 | 0.00 | 0.00 | 0.00 | 0.00 | 0.00 | 0.00 | 0.00 | 0.00 | 0.00 | 0.00 |
| Xingwen County | 380036 | 0.0000 | 0.0000 | 0.0000 | 0.0000 | 0.0000 | 0.0000 | 275.25 | 0.00 | 0.00 | 0.00 | 0.00 | 0.00 | 0.00 | 0.00 | 0.00 | 0.00 | 0.00 | 0.00 | 0.00 | 0.00 | 0.00 | 0.00 | 0.00 | 0.00 |
| Pingshan County | 245184 | 0.0000 | 0.0000 | 0.0000 | 0.0000 | 0.0000 | 0.0000 | 163.22 | 0.00 | 0.00 | 0.00 | 0.00 | 0.00 | 0.00 | 0.00 | 0.00 | 0.00 | 0.00 | 0.00 | 0.00 | 0.00 | 0.00 | 0.00 | 0.00 | 0.00 |
| **Guang'an City** | 3254883 | 0.1733 | 0.0000 | 0.0000 | 0.0000 | 0.0000 | 0.1733 | 513.21 | 88.96 | 0.00 | 0.00 | 0.00 | 0.00 | 88.96 | 13.34 | 10.35 | 4.82 | 4.45 | 0.35 | 0.26 | 0.13 | 1.00 | 0.10 | 0.16 | 0.00 |
| Guang'an District | 744115 | 0.0000 | 0.0000 | 0.0000 | 0.0000 | 0.0000 | 0.0000 | 725.19 | 0.00 | 0.00 | 0.00 | 0.00 | 0.00 | 0.00 | 0.00 | 0.00 | 0.00 | 0.00 | 0.00 | 0.00 | 0.00 | 0.00 | 0.00 | 0.00 | 0.00 |
| Forward area | 232255 | 0.0000 | 0.0000 | 0.0000 | 0.0000 | 0.0000 | 0.0000 | 459.38 | 0.00 | 0.00 | 0.00 | 0.00 | 0.00 | 0.00 | 0.00 | 0.00 | 0.00 | 0.00 | 0.00 | 0.00 | 0.00 | 0.00 | 0.00 | 0.00 | 0.00 |
| Yuechi County | 742747 | 0.1733 | 0.0000 | 0.0000 | 0.0000 | 0.0000 | 0.1733 | 501.75 | 86.97 | 0.00 | 0.00 | 0.00 | 0.00 | 86.97 | 13.05 | 10.12 | 4.71 | 4.35 | 0.35 | 0.26 | 0.12 | 0.98 | 0.10 | 0.15 | 0.00 |
| Wusheng County | 555897 | 0.0000 | 0.0000 | 0.0000 | 0.0000 | 0.0000 | 0.0000 | 581.10 | 0.00 | 0.00 | 0.00 | 0.00 | 0.00 | 0.00 | 0.00 | 0.00 | 0.00 | 0.00 | 0.00 | 0.00 | 0.00 | 0.00 | 0.00 | 0.00 | 0.00 |
| Linshui County | 707537 | 0.0000 | 0.0000 | 0.0000 | 0.0000 | 0.0000 | 0.0000 | 370.19 | 0.00 | 0.00 | 0.00 | 0.00 | 0.00 | 0.00 | 0.00 | 0.00 | 0.00 | 0.00 | 0.00 | 0.00 | 0.00 | 0.00 | 0.00 | 0.00 | 0.00 |
| Huaying City | 272332 | 0.0000 | 0.0000 | 0.0000 | 0.0000 | 0.0000 | 0.0000 | 589.11 | 0.00 | 0.00 | 0.00 | 0.00 | 0.00 | 0.00 | 0.00 | 0.00 | 0.00 | 0.00 | 0.00 | 0.00 | 0.00 | 0.00 | 0.00 | 0.00 | 0.00 |
| **Dazhou City** | 5385422 | 0.3387 | 0.0000 | 0.0000 | 0.0000 | 0.0000 | 0.3387 | 324.66 | 109.95 | 0.00 | 0.00 | 0.00 | 0.00 | 109.95 | 16.49 | 12.79 | 5.96 | 5.50 | 0.44 | 0.33 | 0.16 | 1.24 | 0.12 | 0.19 | 0.00 |
| Tongchuan District | 905678 | 0.0000 | 0.0000 | 0.0000 | 0.0000 | 0.0000 | 0.0000 | 1021.38 | 0.00 | 0.00 | 0.00 | 0.00 | 0.00 | 0.00 | 0.00 | 0.00 | 0.00 | 0.00 | 0.00 | 0.00 | 0.00 | 0.00 | 0.00 | 0.00 | 0.00 |
| Dachuan District | 945191 | 0.0000 | 0.0000 | 0.0000 | 0.0000 | 0.0000 | 0.0000 | 420.78 | 0.00 | 0.00 | 0.00 | 0.00 | 0.00 | 0.00 | 0.00 | 0.00 | 0.00 | 0.00 | 0.00 | 0.00 | 0.00 | 0.00 | 0.00 | 0.00 | 0.00 |
| Xuanhan County | 954090 | 0.0000 | 0.0000 | 0.0000 | 0.0000 | 0.0000 | 0.0000 | 223.32 | 0.00 | 0.00 | 0.00 | 0.00 | 0.00 | 0.00 | 0.00 | 0.00 | 0.00 | 0.00 | 0.00 | 0.00 | 0.00 | 0.00 | 0.00 | 0.00 | 0.00 |
| Kaijiang County | 414310 | 0.0000 | 0.0000 | 0.0000 | 0.0000 | 0.0000 | 0.0000 | 402.28 | 0.00 | 0.00 | 0.00 | 0.00 | 0.00 | 0.00 | 0.00 | 0.00 | 0.00 | 0.00 | 0.00 | 0.00 | 0.00 | 0.00 | 0.00 | 0.00 | 0.00 |
| Dazhu County | 841960 | 0.0000 | 0.0000 | 0.0000 | 0.0000 | 0.0000 | 0.0000 | 405.00 | 0.00 | 0.00 | 0.00 | 0.00 | 0.00 | 0.00 | 0.00 | 0.00 | 0.00 | 0.00 | 0.00 | 0.00 | 0.00 | 0.00 | 0.00 | 0.00 | 0.00 |
| Qu County | 917508 | 0.3387 | 0.0000 | 0.0000 | 0.0000 | 0.0000 | 0.3387 | 454.28 | 153.85 | 0.00 | 0.00 | 0.00 | 0.00 | 153.85 | 23.08 | 17.89 | 8.34 | 7.69 | 0.61 | 0.46 | 0.22 | 1.74 | 0.17 | 0.27 | 0.01 |
| Wanyuan City | 406685 | 0.0000 | 0.0000 | 0.0000 | 0.0000 | 0.0000 | 0.0000 | 100.31 | 0.00 | 0.00 | 0.00 | 0.00 | 0.00 | 0.00 | 0.00 | 0.00 | 0.00 | 0.00 | 0.00 | 0.00 | 0.00 | 0.00 | 0.00 | 0.00 | 0.00 |
| **Ya'an City** | 1434603 | 0.0000 | 0.0000 | 0.0000 | 0.0000 | 0.0000 | 0.0000 | 95.32 | 0.00 | 0.00 | 0.00 | 0.00 | 0.00 | 0.00 | 0.00 | 0.00 | 0.00 | 0.00 | 0.00 | 0.00 | 0.00 | 0.00 | 0.00 | 0.00 | 0.00 |
| Yucheng District | 368909 | 0.0000 | 0.0000 | 0.0000 | 0.0000 | 0.0000 | 0.0000 | 346.59 | 0.00 | 0.00 | 0.00 | 0.00 | 0.00 | 0.00 | 0.00 | 0.00 | 0.00 | 0.00 | 0.00 | 0.00 | 0.00 | 0.00 | 0.00 | 0.00 | 0.00 |
| Mingshan District | 254632 | 0.0000 | 0.0000 | 0.0000 | 0.0000 | 0.0000 | 0.0000 | 411.15 | 0.00 | 0.00 | 0.00 | 0.00 | 0.00 | 0.00 | 0.00 | 0.00 | 0.00 | 0.00 | 0.00 | 0.00 | 0.00 | 0.00 | 0.00 | 0.00 | 0.00 |
| Rongjing County | 131491 | 0.0000 | 0.0000 | 0.0000 | 0.0000 | 0.0000 | 0.0000 | 74.05 | 0.00 | 0.00 | 0.00 | 0.00 | 0.00 | 0.00 | 0.00 | 0.00 | 0.00 | 0.00 | 0.00 | 0.00 | 0.00 | 0.00 | 0.00 | 0.00 | 0.00 |
| Hanyuan County | 285558 | 0.0000 | 0.0000 | 0.0000 | 0.0000 | 0.0000 | 0.0000 | 128.83 | 0.00 | 0.00 | 0.00 | 0.00 | 0.00 | 0.00 | 0.00 | 0.00 | 0.00 | 0.00 | 0.00 | 0.00 | 0.00 | 0.00 | 0.00 | 0.00 | 0.00 |
| Asbestos County | 114116 | 0.0000 | 0.0000 | 0.0000 | 0.0000 | 0.0000 | 0.0000 | 42.62 | 0.00 | 0.00 | 0.00 | 0.00 | 0.00 | 0.00 | 0.00 | 0.00 | 0.00 | 0.00 | 0.00 | 0.00 | 0.00 | 0.00 | 0.00 | 0.00 | 0.00 |
| Tianquan County | 132033 | 0.0000 | 0.0000 | 0.0000 | 0.0000 | 0.0000 | 0.0000 | 55.21 | 0.00 | 0.00 | 0.00 | 0.00 | 0.00 | 0.00 | 0.00 | 0.00 | 0.00 | 0.00 | 0.00 | 0.00 | 0.00 | 0.00 | 0.00 | 0.00 | 0.00 |
| Lushan County | 99824 | 0.0000 | 0.0000 | 0.0000 | 0.0000 | 0.0000 | 0.0000 | 83.88 | 0.00 | 0.00 | 0.00 | 0.00 | 0.00 | 0.00 | 0.00 | 0.00 | 0.00 | 0.00 | 0.00 | 0.00 | 0.00 | 0.00 | 0.00 | 0.00 | 0.00 |
| Baoxing County | 48040 | 0.0000 | 0.0000 | 0.0000 | 0.0000 | 0.0000 | 0.0000 | 15.42 | 0.00 | 0.00 | 0.00 | 0.00 | 0.00 | 0.00 | 0.00 | 0.00 | 0.00 | 0.00 | 0.00 | 0.00 | 0.00 | 0.00 | 0.00 | 0.00 | 0.00 |
| **Bazhong City** | 2712894 | 0.1031 | 0.1269 | 0.0087 | 0.0000 | 0.0000 | 0.2387 | 220.64 | 22.75 | 27.99 | 1.92 | 0.00 | 0.00 | 52.66 | 12.96 | 10.05 | 4.68 | 4.32 | 0.34 | 0.26 | 0.12 | 0.97 | 0.10 | 0.15 | 0.00 |
| Bazhou District | 719038 | 0.0000 | 0.0000 | 0.0000 | 0.0000 | 0.0000 | 0.0000 | 511.12 | 0.00 | 0.00 | 0.00 | 0.00 | 0.00 | 0.00 | 0.00 | 0.00 | 0.00 | 0.00 | 0.00 | 0.00 | 0.00 | 0.00 | 0.00 | 0.00 | 0.00 |
| Enyang District | 345728 | 0.0000 | 0.0000 | 0.0000 | 0.0000 | 0.0000 | 0.0000 | 299.57 | 0.00 | 0.00 | 0.00 | 0.00 | 0.00 | 0.00 | 0.00 | 0.00 | 0.00 | 0.00 | 0.00 | 0.00 | 0.00 | 0.00 | 0.00 | 0.00 | 0.00 |
| Tongjiang County | 521875 | 0.1031 | 0.1269 | 0.0087 | 0.0000 | 0.0000 | 0.2387 | 126.62 | 13.05 | 16.06 | 1.10 | 0.00 | 0.00 | 30.22 | 7.44 | 5.77 | 2.69 | 2.48 | 0.20 | 0.15 | 0.07 | 0.56 | 0.06 | 0.09 | 0.00 |
| Nanjiang County | 467609 | 0.0000 | 0.0000 | 0.0000 | 0.0000 | 0.0000 | 0.0000 | 138.04 | 0.00 | 0.00 | 0.00 | 0.00 | 0.00 | 0.00 | 0.00 | 0.00 | 0.00 | 0.00 | 0.00 | 0.00 | 0.00 | 0.00 | 0.00 | 0.00 | 0.00 |
| Pingchang County | 658644 | 0.0000 | 0.0000 | 0.0000 | 0.0000 | 0.0000 | 0.0000 | 295.95 | 0.00 | 0.00 | 0.00 | 0.00 | 0.00 | 0.00 | 0.00 | 0.00 | 0.00 | 0.00 | 0.00 | 0.00 | 0.00 | 0.00 | 0.00 | 0.00 | 0.00 |
| **Ziyang City** | 2308631 | 0.0000 | 0.0000 | 0.0000 | 0.0000 | 0.0000 | 0.0000 | 401.82 | 0.00 | 0.00 | 0.00 | 0.00 | 0.00 | 0.00 | 0.00 | 0.00 | 0.00 | 0.00 | 0.00 | 0.00 | 0.00 | 0.00 | 0.00 | 0.00 | 0.00 |
| Yanjiang District | 867119 | 0.0000 | 0.0000 | 0.0000 | 0.0000 | 0.0000 | 0.0000 | 530.41 | 0.00 | 0.00 | 0.00 | 0.00 | 0.00 | 0.00 | 0.00 | 0.00 | 0.00 | 0.00 | 0.00 | 0.00 | 0.00 | 0.00 | 0.00 | 0.00 | 0.00 |
| Anyue County | 950939 | 0.0000 | 0.0000 | 0.0000 | 0.0000 | 0.0000 | 0.0000 | 353.92 | 0.00 | 0.00 | 0.00 | 0.00 | 0.00 | 0.00 | 0.00 | 0.00 | 0.00 | 0.00 | 0.00 | 0.00 | 0.00 | 0.00 | 0.00 | 0.00 | 0.00 |
| Lezhi County | 490573 | 0.0000 | 0.0000 | 0.0000 | 0.0000 | 0.0000 | 0.0000 | 344.58 | 0.00 | 0.00 | 0.00 | 0.00 | 0.00 | 0.00 | 0.00 | 0.00 | 0.00 | 0.00 | 0.00 | 0.00 | 0.00 | 0.00 | 0.00 | 0.00 | 0.00 |
| **Aba Tibetan and Qiang Autonomous Prefecture** | 822587 | 0.0000 | 0.0000 | 0.0000 | 0.0000 | 0.0000 | 0.0000 | 9.90 | 0.00 | 0.00 | 0.00 | 0.00 | 0.00 | 0.00 | 0.00 | 0.00 | 0.00 | 0.00 | 0.00 | 0.00 | 0.00 | 0.00 | 0.00 | 0.00 | 0.00 |
| Maerkang City | 58390 | 0.0000 | 0.0000 | 0.0000 | 0.0000 | 0.0000 | 0.0000 | 8.82 | 0.00 | 0.00 | 0.00 | 0.00 | 0.00 | 0.00 | 0.00 | 0.00 | 0.00 | 0.00 | 0.00 | 0.00 | 0.00 | 0.00 | 0.00 | 0.00 | 0.00 |
| Wenchuan County | 82971 | 0.0000 | 0.0000 | 0.0000 | 0.0000 | 0.0000 | 0.0000 | 20.31 | 0.00 | 0.00 | 0.00 | 0.00 | 0.00 | 0.00 | 0.00 | 0.00 | 0.00 | 0.00 | 0.00 | 0.00 | 0.00 | 0.00 | 0.00 | 0.00 | 0.00 |
| Li County | 36926 | 0.0000 | 0.0000 | 0.0000 | 0.0000 | 0.0000 | 0.0000 | 8.55 | 0.00 | 0.00 | 0.00 | 0.00 | 0.00 | 0.00 | 0.00 | 0.00 | 0.00 | 0.00 | 0.00 | 0.00 | 0.00 | 0.00 | 0.00 | 0.00 | 0.00 |
| Mao County | 95361 | 0.0000 | 0.0000 | 0.0000 | 0.0000 | 0.0000 | 0.0000 | 24.48 | 0.00 | 0.00 | 0.00 | 0.00 | 0.00 | 0.00 | 0.00 | 0.00 | 0.00 | 0.00 | 0.00 | 0.00 | 0.00 | 0.00 | 0.00 | 0.00 | 0.00 |
| Songpan County | 66937 | 0.0000 | 0.0000 | 0.0000 | 0.0000 | 0.0000 | 0.0000 | 8.02 | 0.00 | 0.00 | 0.00 | 0.00 | 0.00 | 0.00 | 0.00 | 0.00 | 0.00 | 0.00 | 0.00 | 0.00 | 0.00 | 0.00 | 0.00 | 0.00 | 0.00 |
| Jiuzhaigou County | 66055 | 0.0000 | 0.0000 | 0.0000 | 0.0000 | 0.0000 | 0.0000 | 12.49 | 0.00 | 0.00 | 0.00 | 0.00 | 0.00 | 0.00 | 0.00 | 0.00 | 0.00 | 0.00 | 0.00 | 0.00 | 0.00 | 0.00 | 0.00 | 0.00 | 0.00 |
| Jinchuan County | 58068 | 0.0000 | 0.0000 | 0.0000 | 0.0000 | 0.0000 | 0.0000 | 10.84 | 0.00 | 0.00 | 0.00 | 0.00 | 0.00 | 0.00 | 0.00 | 0.00 | 0.00 | 0.00 | 0.00 | 0.00 | 0.00 | 0.00 | 0.00 | 0.00 | 0.00 |
| Xiaojin County | 64813 | 0.0000 | 0.0000 | 0.0000 | 0.0000 | 0.0000 | 0.0000 | 11.65 | 0.00 | 0.00 | 0.00 | 0.00 | 0.00 | 0.00 | 0.00 | 0.00 | 0.00 | 0.00 | 0.00 | 0.00 | 0.00 | 0.00 | 0.00 | 0.00 | 0.00 |
| Heishui County | 44564 | 0.0000 | 0.0000 | 0.0000 | 0.0000 | 0.0000 | 0.0000 | 10.76 | 0.00 | 0.00 | 0.00 | 0.00 | 0.00 | 0.00 | 0.00 | 0.00 | 0.00 | 0.00 | 0.00 | 0.00 | 0.00 | 0.00 | 0.00 | 0.00 | 0.00 |
| Rangtang County | 44679 | 0.0000 | 0.0000 | 0.0000 | 0.0000 | 0.0000 | 0.0000 | 6.67 | 0.00 | 0.00 | 0.00 | 0.00 | 0.00 | 0.00 | 0.00 | 0.00 | 0.00 | 0.00 | 0.00 | 0.00 | 0.00 | 0.00 | 0.00 | 0.00 | 0.00 |
| A'ba County | 80467 | 0.0000 | 0.0000 | 0.0000 | 0.0000 | 0.0000 | 0.0000 | 7.95 | 0.00 | 0.00 | 0.00 | 0.00 | 0.00 | 0.00 | 0.00 | 0.00 | 0.00 | 0.00 | 0.00 | 0.00 | 0.00 | 0.00 | 0.00 | 0.00 | 0.00 |
| Ruoergai County | 76712 | 0.0000 | 0.0000 | 0.0000 | 0.0000 | 0.0000 | 0.0000 | 7.43 | 0.00 | 0.00 | 0.00 | 0.00 | 0.00 | 0.00 | 0.00 | 0.00 | 0.00 | 0.00 | 0.00 | 0.00 | 0.00 | 0.00 | 0.00 | 0.00 | 0.00 |
| Hongyuan County | 46644 | 0.0000 | 0.0000 | 0.0000 | 0.0000 | 0.0000 | 0.0000 | 5.62 | 0.00 | 0.00 | 0.00 | 0.00 | 0.00 | 0.00 | 0.00 | 0.00 | 0.00 | 0.00 | 0.00 | 0.00 | 0.00 | 0.00 | 0.00 | 0.00 | 0.00 |
| **Ganzi Tibetan Autonomous Prefecture** | 1107431 | 0.0000 | 0.0000 | 0.0000 | 0.0000 | 0.0000 | 0.0000 | 7.40 | 0.00 | 0.00 | 0.00 | 0.00 | 0.00 | 0.00 | 0.00 | 0.00 | 0.00 | 0.00 | 0.00 | 0.00 | 0.00 | 0.00 | 0.00 | 0.00 | 0.00 |
| Kangding City | 126785 | 0.0000 | 0.0000 | 0.0000 | 0.0000 | 0.0000 | 0.0000 | 10.93 | 0.00 | 0.00 | 0.00 | 0.00 | 0.00 | 0.00 | 0.00 | 0.00 | 0.00 | 0.00 | 0.00 | 0.00 | 0.00 | 0.00 | 0.00 | 0.00 | 0.00 |
| Luding County | 84204 | 0.0000 | 0.0000 | 0.0000 | 0.0000 | 0.0000 | 0.0000 | 38.89 | 0.00 | 0.00 | 0.00 | 0.00 | 0.00 | 0.00 | 0.00 | 0.00 | 0.00 | 0.00 | 0.00 | 0.00 | 0.00 | 0.00 | 0.00 | 0.00 | 0.00 |
| Danba County | 49872 | 0.0000 | 0.0000 | 0.0000 | 0.0000 | 0.0000 | 0.0000 | 11.07 | 0.00 | 0.00 | 0.00 | 0.00 | 0.00 | 0.00 | 0.00 | 0.00 | 0.00 | 0.00 | 0.00 | 0.00 | 0.00 | 0.00 | 0.00 | 0.00 | 0.00 |
| Jiulong County | 53738 | 0.0000 | 0.0000 | 0.0000 | 0.0000 | 0.0000 | 0.0000 | 7.94 | 0.00 | 0.00 | 0.00 | 0.00 | 0.00 | 0.00 | 0.00 | 0.00 | 0.00 | 0.00 | 0.00 | 0.00 | 0.00 | 0.00 | 0.00 | 0.00 | 0.00 |
| Yajiang County | 51162 | 0.0000 | 0.0000 | 0.0000 | 0.0000 | 0.0000 | 0.0000 | 6.76 | 0.00 | 0.00 | 0.00 | 0.00 | 0.00 | 0.00 | 0.00 | 0.00 | 0.00 | 0.00 | 0.00 | 0.00 | 0.00 | 0.00 | 0.00 | 0.00 | 0.00 |
| Daofu County | 53378 | 0.0000 | 0.0000 | 0.0000 | 0.0000 | 0.0000 | 0.0000 | 7.60 | 0.00 | 0.00 | 0.00 | 0.00 | 0.00 | 0.00 | 0.00 | 0.00 | 0.00 | 0.00 | 0.00 | 0.00 | 0.00 | 0.00 | 0.00 | 0.00 | 0.00 |
| Luhuo County | 47185 | 0.0000 | 0.0000 | 0.0000 | 0.0000 | 0.0000 | 0.0000 | 10.54 | 0.00 | 0.00 | 0.00 | 0.00 | 0.00 | 0.00 | 0.00 | 0.00 | 0.00 | 0.00 | 0.00 | 0.00 | 0.00 | 0.00 | 0.00 | 0.00 | 0.00 |
| Ganzi County | 72698 | 0.0000 | 0.0000 | 0.0000 | 0.0000 | 0.0000 | 0.0000 | 10.59 | 0.00 | 0.00 | 0.00 | 0.00 | 0.00 | 0.00 | 0.00 | 0.00 | 0.00 | 0.00 | 0.00 | 0.00 | 0.00 | 0.00 | 0.00 | 0.00 | 0.00 |
| Xinlong County | 45698 | 0.0000 | 0.0000 | 0.0000 | 0.0000 | 0.0000 | 0.0000 | 4.94 | 0.00 | 0.00 | 0.00 | 0.00 | 0.00 | 0.00 | 0.00 | 0.00 | 0.00 | 0.00 | 0.00 | 0.00 | 0.00 | 0.00 | 0.00 | 0.00 | 0.00 |
| Derge County | 88542 | 0.0000 | 0.0000 | 0.0000 | 0.0000 | 0.0000 | 0.0000 | 7.74 | 0.00 | 0.00 | 0.00 | 0.00 | 0.00 | 0.00 | 0.00 | 0.00 | 0.00 | 0.00 | 0.00 | 0.00 | 0.00 | 0.00 | 0.00 | 0.00 | 0.00 |
| Baiyu County | 59524 | 0.0000 | 0.0000 | 0.0000 | 0.0000 | 0.0000 | 0.0000 | 5.80 | 0.00 | 0.00 | 0.00 | 0.00 | 0.00 | 0.00 | 0.00 | 0.00 | 0.00 | 0.00 | 0.00 | 0.00 | 0.00 | 0.00 | 0.00 | 0.00 | 0.00 |
| Shiqu County | 103633 | 0.0000 | 0.0000 | 0.0000 | 0.0000 | 0.0000 | 0.0000 | 4.63 | 0.00 | 0.00 | 0.00 | 0.00 | 0.00 | 0.00 | 0.00 | 0.00 | 0.00 | 0.00 | 0.00 | 0.00 | 0.00 | 0.00 | 0.00 | 0.00 | 0.00 |
| Seda County | 64681 | 0.0000 | 0.0000 | 0.0000 | 0.0000 | 0.0000 | 0.0000 | 7.41 | 0.00 | 0.00 | 0.00 | 0.00 | 0.00 | 0.00 | 0.00 | 0.00 | 0.00 | 0.00 | 0.00 | 0.00 | 0.00 | 0.00 | 0.00 | 0.00 | 0.00 |
| Litang County | 67293 | 0.0000 | 0.0000 | 0.0000 | 0.0000 | 0.0000 | 0.0000 | 4.80 | 0.00 | 0.00 | 0.00 | 0.00 | 0.00 | 0.00 | 0.00 | 0.00 | 0.00 | 0.00 | 0.00 | 0.00 | 0.00 | 0.00 | 0.00 | 0.00 | 0.00 |
| Batang County | 49967 | 0.0000 | 0.0000 | 0.0000 | 0.0000 | 0.0000 | 0.0000 | 6.52 | 0.00 | 0.00 | 0.00 | 0.00 | 0.00 | 0.00 | 0.00 | 0.00 | 0.00 | 0.00 | 0.00 | 0.00 | 0.00 | 0.00 | 0.00 | 0.00 | 0.00 |
| Xiangcheng County | 31407 | 0.0000 | 0.0000 | 0.0000 | 0.0000 | 0.0000 | 0.0000 | 6.35 | 0.00 | 0.00 | 0.00 | 0.00 | 0.00 | 0.00 | 0.00 | 0.00 | 0.00 | 0.00 | 0.00 | 0.00 | 0.00 | 0.00 | 0.00 | 0.00 | 0.00 |
| Daocheng County | 32916 | 0.0000 | 0.0000 | 0.0000 | 0.0000 | 0.0000 | 0.0000 | 4.65 | 0.00 | 0.00 | 0.00 | 0.00 | 0.00 | 0.00 | 0.00 | 0.00 | 0.00 | 0.00 | 0.00 | 0.00 | 0.00 | 0.00 | 0.00 | 0.00 | 0.00 |
| Derong County | 24748 | 0.0000 | 0.0000 | 0.0000 | 0.0000 | 0.0000 | 0.0000 | 8.51 | 0.00 | 0.00 | 0.00 | 0.00 | 0.00 | 0.00 | 0.00 | 0.00 | 0.00 | 0.00 | 0.00 | 0.00 | 0.00 | 0.00 | 0.00 | 0.00 | 0.00 |
| **Liangshan Yi Autonomous Prefecture** | 4858359 | 89.7533 | 42.8666 | 20.2953 | 0.5333 | 0.0000 | 153.4486 | 80.60 | 7234.47 | 3455.21 | 1635.88 | 42.99 | 0.00 | 12368.55 | 3137.65 | 2432.94 | 1133.32 | 1045.78 | 83.15 | 62.13 | 29.49 | 235.95 | 23.53 | 36.71 | 0.94 |
| Xichang City | 955041 | 71.8260 | 31.0305 | 14.4593 | 0.1333 | 0.0000 | 117.4491 | 359.34 | 25809.61 | 11150.34 | 5195.75 | 47.91 | 0.00 | 42203.60 | 10372.32 | 8042.70 | 3746.48 | 3457.09 | 274.87 | 205.37 | 97.50 | 780.00 | 77.79 | 121.36 | 3.11 |
| Muli Tibetan Autonomous County | 122944 | 0.2000 | 0.0000 | 0.0000 | 0.0000 | 0.0000 | 0.2000 | 9.30 | 1.86 | 0.00 | 0.00 | 0.00 | 0.00 | 1.86 | 0.28 | 0.22 | 0.10 | 0.09 | 0.01 | 0.01 | 0.00 | 0.02 | 0.00 | 0.00 | 0.00 |
| Yanyuan County | 340898 | 0.0000 | 0.0000 | 0.0000 | 0.0000 | 0.0000 | 0.0000 | 40.55 | 0.00 | 0.00 | 0.00 | 0.00 | 0.00 | 0.00 | 0.00 | 0.00 | 0.00 | 0.00 | 0.00 | 0.00 | 0.00 | 0.00 | 0.00 | 0.00 | 0.00 |
| Dechang County | 216533 | 0.4667 | 7.7633 | 5.0667 | 0.2000 | 0.0000 | 13.4967 | 94.23 | 43.97 | 731.55 | 477.44 | 18.85 | 0.00 | 1271.80 | 527.60 | 409.10 | 190.57 | 175.85 | 13.98 | 10.45 | 4.96 | 39.68 | 3.96 | 6.17 | 0.16 |
| Huili County | 390531 | 8.3467 | 1.5867 | 0.2333 | 0.0000 | 0.0000 | 10.1667 | 86.15 | 719.07 | 136.69 | 20.10 | 0.00 | 0.00 | 875.87 | 160.93 | 124.79 | 58.13 | 53.64 | 4.26 | 3.19 | 1.51 | 12.10 | 1.21 | 1.88 | 0.05 |
| Huidong County | 346082 | 0.0000 | 0.0000 | 0.0000 | 0.0000 | 0.0000 | 0.0000 | 107.44 | 0.00 | 0.00 | 0.00 | 0.00 | 0.00 | 0.00 | 0.00 | 0.00 | 0.00 | 0.00 | 0.00 | 0.00 | 0.00 | 0.00 | 0.00 | 0.00 | 0.00 |
| Ningnan County | 184293 | 6.6667 | 2.0000 | 0.2667 | 0.2000 | 0.0000 | 9.1333 | 110.07 | 733.77 | 220.13 | 29.35 | 22.01 | 0.00 | 1005.26 | 211.32 | 163.86 | 76.33 | 70.43 | 5.60 | 4.18 | 1.99 | 15.89 | 1.58 | 2.47 | 0.06 |
| Puge County | 180052 | 0.0000 | 0.0000 | 0.0000 | 0.0000 | 0.0000 | 0.0000 | 94.49 | 0.00 | 0.00 | 0.00 | 0.00 | 0.00 | 0.00 | 0.00 | 0.00 | 0.00 | 0.00 | 0.00 | 0.00 | 0.00 | 0.00 | 0.00 | 0.00 | 0.00 |
| Butuo County | 185553 | 0.0000 | 0.0000 | 0.0000 | 0.0000 | 0.0000 | 0.0000 | 110.10 | 0.00 | 0.00 | 0.00 | 0.00 | 0.00 | 0.00 | 0.00 | 0.00 | 0.00 | 0.00 | 0.00 | 0.00 | 0.00 | 0.00 | 0.00 | 0.00 | 0.00 |
| Jinyang County | 170063 | 0.0000 | 0.0000 | 0.0000 | 0.0000 | 0.0000 | 0.0000 | 107.22 | 0.00 | 0.00 | 0.00 | 0.00 | 0.00 | 0.00 | 0.00 | 0.00 | 0.00 | 0.00 | 0.00 | 0.00 | 0.00 | 0.00 | 0.00 | 0.00 | 0.00 |
| Zhaojue County | 252435 | 0.0000 | 0.0000 | 0.0000 | 0.0000 | 0.0000 | 0.0000 | 93.49 | 0.00 | 0.00 | 0.00 | 0.00 | 0.00 | 0.00 | 0.00 | 0.00 | 0.00 | 0.00 | 0.00 | 0.00 | 0.00 | 0.00 | 0.00 | 0.00 | 0.00 |
| Xide County | 158139 | 2.2073 | 0.3927 | 0.2160 | 0.0000 | 0.0000 | 2.8160 | 71.73 | 158.34 | 28.17 | 15.49 | 0.00 | 0.00 | 202.00 | 41.50 | 32.18 | 14.99 | 13.83 | 1.10 | 0.82 | 0.39 | 3.12 | 0.31 | 0.49 | 0.01 |
| Mianning County | 369166 | 0.0400 | 0.0934 | 0.0533 | 0.0000 | 0.0000 | 0.1868 | 83.53 | 3.34 | 7.81 | 4.46 | 0.00 | 0.00 | 15.60 | 5.52 | 4.28 | 1.99 | 1.84 | 0.15 | 0.11 | 0.05 | 0.41 | 0.04 | 0.06 | 0.00 |
| Yuexi County | 301865 | 0.0000 | 0.0000 | 0.0000 | 0.0000 | 0.0000 | 0.0000 | 133.81 | 0.00 | 0.00 | 0.00 | 0.00 | 0.00 | 0.00 | 0.00 | 0.00 | 0.00 | 0.00 | 0.00 | 0.00 | 0.00 | 0.00 | 0.00 | 0.00 | 0.00 |
| Ganluo County | 205991 | 0.0000 | 0.0000 | 0.0000 | 0.0000 | 0.0000 | 0.0000 | 95.65 | 0.00 | 0.00 | 0.00 | 0.00 | 0.00 | 0.00 | 0.00 | 0.00 | 0.00 | 0.00 | 0.00 | 0.00 | 0.00 | 0.00 | 0.00 | 0.00 | 0.00 |
| Meigu County | 238624 | 0.0000 | 0.0000 | 0.0000 | 0.0000 | 0.0000 | 0.0000 | 94.98 | 0.00 | 0.00 | 0.00 | 0.00 | 0.00 | 0.00 | 0.00 | 0.00 | 0.00 | 0.00 | 0.00 | 0.00 | 0.00 | 0.00 | 0.00 | 0.00 | 0.00 |
| Leibo County | 240149 | 0.0000 | 0.0000 | 0.0000 | 0.0000 | 0.0000 | 0.0000 | 84.58 | 0.00 | 0.00 | 0.00 | 0.00 | 0.00 | 0.00 | 0.00 | 0.00 | 0.00 | 0.00 | 0.00 | 0.00 | 0.00 | 0.00 | 0.00 | 0.00 | 0.00 |
| **Guizhou Province** | 38562148 | 8.3446 | 4.0536 | 0.9549 | 0.7050 | 0.0071 | 14.0652 | 218.97 | 1827.25 | 887.62 | 209.10 | 154.38 | 1.55 | 3079.91 | 790.74 | 613.14 | 285.61 | 263.55 | 20.95 | 15.66 | 7.43 | 59.46 | 5.93 | 9.25 | 0.24 |
| **Guiyang City** | 5987018 | 0.0000 | 0.0000 | 0.0000 | 0.0000 | 0.0000 | 0.0000 | 744.33 | 0.00 | 0.00 | 0.00 | 0.00 | 0.00 | 0.00 | 0.00 | 0.00 | 0.00 | 0.00 | 0.00 | 0.00 | 0.00 | 0.00 | 0.00 | 0.00 | 0.00 |
| Nanming District | 1047792 | 0.0000 | 0.0000 | 0.0000 | 0.0000 | 0.0000 | 0.0000 | 4934.97 | 0.00 | 0.00 | 0.00 | 0.00 | 0.00 | 0.00 | 0.00 | 0.00 | 0.00 | 0.00 | 0.00 | 0.00 | 0.00 | 0.00 | 0.00 | 0.00 | 0.00 |
| Yunyan District | 1056819 | 0.0000 | 0.0000 | 0.0000 | 0.0000 | 0.0000 | 0.0000 | 11929.33 | 0.00 | 0.00 | 0.00 | 0.00 | 0.00 | 0.00 | 0.00 | 0.00 | 0.00 | 0.00 | 0.00 | 0.00 | 0.00 | 0.00 | 0.00 | 0.00 | 0.00 |
| Huaxi District | 966276 | 0.0000 | 0.0000 | 0.0000 | 0.0000 | 0.0000 | 0.0000 | 1003.11 | 0.00 | 0.00 | 0.00 | 0.00 | 0.00 | 0.00 | 0.00 | 0.00 | 0.00 | 0.00 | 0.00 | 0.00 | 0.00 | 0.00 | 0.00 | 0.00 | 0.00 |
| Wudang District | 336363 | 0.0000 | 0.0000 | 0.0000 | 0.0000 | 0.0000 | 0.0000 | 492.95 | 0.00 | 0.00 | 0.00 | 0.00 | 0.00 | 0.00 | 0.00 | 0.00 | 0.00 | 0.00 | 0.00 | 0.00 | 0.00 | 0.00 | 0.00 | 0.00 | 0.00 |
| Baiyun District | 456250 | 0.0000 | 0.0000 | 0.0000 | 0.0000 | 0.0000 | 0.0000 | 1692.95 | 0.00 | 0.00 | 0.00 | 0.00 | 0.00 | 0.00 | 0.00 | 0.00 | 0.00 | 0.00 | 0.00 | 0.00 | 0.00 | 0.00 | 0.00 | 0.00 | 0.00 |
| Guanshanhu District | 642634 | 0.0000 | 0.0000 | 0.0000 | 0.0000 | 0.0000 | 0.0000 | 2096.07 | 0.00 | 0.00 | 0.00 | 0.00 | 0.00 | 0.00 | 0.00 | 0.00 | 0.00 | 0.00 | 0.00 | 0.00 | 0.00 | 0.00 | 0.00 | 0.00 | 0.00 |
| Kaiyang County | 343871 | 0.0000 | 0.0000 | 0.0000 | 0.0000 | 0.0000 | 0.0000 | 169.89 | 0.00 | 0.00 | 0.00 | 0.00 | 0.00 | 0.00 | 0.00 | 0.00 | 0.00 | 0.00 | 0.00 | 0.00 | 0.00 | 0.00 | 0.00 | 0.00 | 0.00 |
| Xifeng County | 219835 | 0.0000 | 0.0000 | 0.0000 | 0.0000 | 0.0000 | 0.0000 | 211.85 | 0.00 | 0.00 | 0.00 | 0.00 | 0.00 | 0.00 | 0.00 | 0.00 | 0.00 | 0.00 | 0.00 | 0.00 | 0.00 | 0.00 | 0.00 | 0.00 | 0.00 |
| Xiuwen County | 288090 | 0.0000 | 0.0000 | 0.0000 | 0.0000 | 0.0000 | 0.0000 | 268.44 | 0.00 | 0.00 | 0.00 | 0.00 | 0.00 | 0.00 | 0.00 | 0.00 | 0.00 | 0.00 | 0.00 | 0.00 | 0.00 | 0.00 | 0.00 | 0.00 | 0.00 |
| Qingzhen City | 629088 | 0.0000 | 0.0000 | 0.0000 | 0.0000 | 0.0000 | 0.0000 | 453.92 | 0.00 | 0.00 | 0.00 | 0.00 | 0.00 | 0.00 | 0.00 | 0.00 | 0.00 | 0.00 | 0.00 | 0.00 | 0.00 | 0.00 | 0.00 | 0.00 | 0.00 |
| **Liupanshui City** | 3031602 | 0.2267 | 0.1867 | 0.0400 | 0.0000 | 0.0000 | 0.4533 | 305.78 | 69.31 | 57.08 | 12.23 | 0.00 | 0.00 | 138.62 | 34.86 | 27.03 | 12.59 | 11.62 | 0.92 | 0.69 | 0.33 | 2.62 | 0.26 | 0.41 | 0.01 |
| Zhongshan District | 674249 | 0.0000 | 0.0000 | 0.0000 | 0.0000 | 0.0000 | 0.0000 | 635.30 | 0.00 | 0.00 | 0.00 | 0.00 | 0.00 | 0.00 | 0.00 | 0.00 | 0.00 | 0.00 | 0.00 | 0.00 | 0.00 | 0.00 | 0.00 | 0.00 | 0.00 |
| Liuzhite District | 536873 | 0.0333 | 0.0533 | 0.0000 | 0.0000 | 0.0000 | 0.0867 | 298.73 | 9.96 | 15.93 | 0.00 | 0.00 | 0.00 | 25.89 | 6.27 | 4.86 | 2.27 | 2.09 | 0.17 | 0.12 | 0.06 | 0.47 | 0.05 | 0.07 | 0.00 |
| Shuicheng County | 746407 | 0.0133 | 0.0000 | 0.0000 | 0.0000 | 0.0000 | 0.0133 | 247.49 | 3.30 | 0.00 | 0.00 | 0.00 | 0.00 | 3.30 | 0.49 | 0.38 | 0.18 | 0.16 | 0.01 | 0.01 | 0.00 | 0.04 | 0.00 | 0.01 | 0.00 |
| Panzhou City | 1074073 | 0.1800 | 0.1333 | 0.0400 | 0.0000 | 0.0000 | 0.3533 | 265.86 | 47.85 | 35.45 | 10.63 | 0.00 | 0.00 | 93.94 | 24.19 | 18.76 | 8.74 | 8.06 | 0.64 | 0.48 | 0.23 | 1.82 | 0.18 | 0.28 | 0.01 |
| **Zunyi City** | 6606675 | 0.0007 | 0.0000 | 0.0000 | 0.0000 | 0.0000 | 0.0007 | 214.70 | 0.14 | 0.00 | 0.00 | 0.00 | 0.00 | 0.14 | 0.02 | 0.02 | 0.01 | 0.01 | 0.00 | 0.00 | 0.00 | 0.00 | 0.00 | 0.00 | 0.00 |
| Honghuagang District | 971337 | 0.0000 | 0.0000 | 0.0000 | 0.0000 | 0.0000 | 0.0000 | 694.90 | 0.00 | 0.00 | 0.00 | 0.00 | 0.00 | 0.00 | 0.00 | 0.00 | 0.00 | 0.00 | 0.00 | 0.00 | 0.00 | 0.00 | 0.00 | 0.00 | 0.00 |
| Huichuan District | 627721 | 0.0000 | 0.0000 | 0.0000 | 0.0000 | 0.0000 | 0.0000 | 413.36 | 0.00 | 0.00 | 0.00 | 0.00 | 0.00 | 0.00 | 0.00 | 0.00 | 0.00 | 0.00 | 0.00 | 0.00 | 0.00 | 0.00 | 0.00 | 0.00 | 0.00 |
| Bozhou District | 761491 | 0.0000 | 0.0000 | 0.0000 | 0.0000 | 0.0000 | 0.0000 | 306.07 | 0.00 | 0.00 | 0.00 | 0.00 | 0.00 | 0.00 | 0.00 | 0.00 | 0.00 | 0.00 | 0.00 | 0.00 | 0.00 | 0.00 | 0.00 | 0.00 | 0.00 |
| Tongzi County | 529471 | 0.0000 | 0.0000 | 0.0000 | 0.0000 | 0.0000 | 0.0000 | 165.04 | 0.00 | 0.00 | 0.00 | 0.00 | 0.00 | 0.00 | 0.00 | 0.00 | 0.00 | 0.00 | 0.00 | 0.00 | 0.00 | 0.00 | 0.00 | 0.00 | 0.00 |
| Suiyang County | 379677 | 0.0000 | 0.0000 | 0.0000 | 0.0000 | 0.0000 | 0.0000 | 149.04 | 0.00 | 0.00 | 0.00 | 0.00 | 0.00 | 0.00 | 0.00 | 0.00 | 0.00 | 0.00 | 0.00 | 0.00 | 0.00 | 0.00 | 0.00 | 0.00 | 0.00 |
| Zheng'an County | 396159 | 0.0000 | 0.0000 | 0.0000 | 0.0000 | 0.0000 | 0.0000 | 153.04 | 0.00 | 0.00 | 0.00 | 0.00 | 0.00 | 0.00 | 0.00 | 0.00 | 0.00 | 0.00 | 0.00 | 0.00 | 0.00 | 0.00 | 0.00 | 0.00 | 0.00 |
| Daozhen Qianlao and Miao Autonomous County | 243846 | 0.0000 | 0.0000 | 0.0000 | 0.0000 | 0.0000 | 0.0000 | 113.06 | 0.00 | 0.00 | 0.00 | 0.00 | 0.00 | 0.00 | 0.00 | 0.00 | 0.00 | 0.00 | 0.00 | 0.00 | 0.00 | 0.00 | 0.00 | 0.00 | 0.00 |
| Wuchuan Lao and Miao Autonomous County | 308466 | 0.0000 | 0.0000 | 0.0000 | 0.0000 | 0.0000 | 0.0000 | 111.15 | 0.00 | 0.00 | 0.00 | 0.00 | 0.00 | 0.00 | 0.00 | 0.00 | 0.00 | 0.00 | 0.00 | 0.00 | 0.00 | 0.00 | 0.00 | 0.00 | 0.00 |
| Fenggang County | 304156 | 0.0000 | 0.0000 | 0.0000 | 0.0000 | 0.0000 | 0.0000 | 161.50 | 0.00 | 0.00 | 0.00 | 0.00 | 0.00 | 0.00 | 0.00 | 0.00 | 0.00 | 0.00 | 0.00 | 0.00 | 0.00 | 0.00 | 0.00 | 0.00 | 0.00 |
| Meitan County | 372865 | 0.0000 | 0.0000 | 0.0000 | 0.0000 | 0.0000 | 0.0000 | 199.71 | 0.00 | 0.00 | 0.00 | 0.00 | 0.00 | 0.00 | 0.00 | 0.00 | 0.00 | 0.00 | 0.00 | 0.00 | 0.00 | 0.00 | 0.00 | 0.00 | 0.00 |
| Yuqing County | 223952 | 0.0000 | 0.0000 | 0.0000 | 0.0000 | 0.0000 | 0.0000 | 137.68 | 0.00 | 0.00 | 0.00 | 0.00 | 0.00 | 0.00 | 0.00 | 0.00 | 0.00 | 0.00 | 0.00 | 0.00 | 0.00 | 0.00 | 0.00 | 0.00 | 0.00 |
| Xishui County | 584947 | 0.0000 | 0.0000 | 0.0000 | 0.0000 | 0.0000 | 0.0000 | 190.26 | 0.00 | 0.00 | 0.00 | 0.00 | 0.00 | 0.00 | 0.00 | 0.00 | 0.00 | 0.00 | 0.00 | 0.00 | 0.00 | 0.00 | 0.00 | 0.00 | 0.00 |
| Chishui City | 247287 | 0.0000 | 0.0000 | 0.0000 | 0.0000 | 0.0000 | 0.0000 | 133.54 | 0.00 | 0.00 | 0.00 | 0.00 | 0.00 | 0.00 | 0.00 | 0.00 | 0.00 | 0.00 | 0.00 | 0.00 | 0.00 | 0.00 | 0.00 | 0.00 | 0.00 |
| Renhuai City | 655300 | 0.0007 | 0.0000 | 0.0000 | 0.0000 | 0.0000 | 0.0007 | 366.50 | 0.24 | 0.00 | 0.00 | 0.00 | 0.00 | 0.24 | 0.04 | 0.03 | 0.01 | 0.01 | 0.00 | 0.00 | 0.00 | 0.00 | 0.00 | 0.00 | 0.00 |
| **Anshun City** | 2470630 | 0.2533 | 0.0113 | 0.0047 | 0.0013 | 0.0000 | 0.2707 | 267.57 | 67.79 | 3.03 | 1.25 | 0.36 | 0.00 | 72.42 | 12.11 | 9.39 | 4.37 | 4.04 | 0.32 | 0.24 | 0.11 | 0.91 | 0.09 | 0.14 | 0.00 |
| Xixiu District | 870441 | 0.0567 | 0.0000 | 0.0000 | 0.0000 | 0.0000 | 0.0567 | 503.81 | 28.55 | 0.00 | 0.00 | 0.00 | 0.00 | 28.55 | 4.28 | 3.32 | 1.55 | 1.43 | 0.11 | 0.08 | 0.04 | 0.32 | 0.03 | 0.05 | 0.00 |
| Pingba District | 347060 | 0.0000 | 0.0000 | 0.0000 | 0.0000 | 0.0000 | 0.0000 | 351.32 | 0.00 | 0.00 | 0.00 | 0.00 | 0.00 | 0.00 | 0.00 | 0.00 | 0.00 | 0.00 | 0.00 | 0.00 | 0.00 | 0.00 | 0.00 | 0.00 | 0.00 |
| Puding County | 376285 | 0.0000 | 0.0000 | 0.0000 | 0.0000 | 0.0000 | 0.0000 | 347.96 | 0.00 | 0.00 | 0.00 | 0.00 | 0.00 | 0.00 | 0.00 | 0.00 | 0.00 | 0.00 | 0.00 | 0.00 | 0.00 | 0.00 | 0.00 | 0.00 | 0.00 |
| Zhenning Buyi and Miao Autonomous County | 299696 | 0.1400 | 0.0000 | 0.0000 | 0.0000 | 0.0000 | 0.1400 | 174.57 | 24.44 | 0.00 | 0.00 | 0.00 | 0.00 | 24.44 | 3.67 | 2.84 | 1.32 | 1.22 | 0.10 | 0.07 | 0.03 | 0.28 | 0.03 | 0.04 | 0.00 |
| Guanling Buyi and Miao Autonomous County | 283497 | 0.0567 | 0.0113 | 0.0047 | 0.0013 | 0.0000 | 0.0740 | 193.61 | 10.97 | 2.19 | 0.90 | 0.26 | 0.00 | 14.33 | 3.05 | 2.37 | 1.10 | 1.02 | 0.08 | 0.06 | 0.03 | 0.23 | 0.02 | 0.04 | 0.00 |
| Ziyun Miao Buyi Autonomous County | 293651 | 0.0000 | 0.0000 | 0.0000 | 0.0000 | 0.0000 | 0.0000 | 130.20 | 0.00 | 0.00 | 0.00 | 0.00 | 0.00 | 0.00 | 0.00 | 0.00 | 0.00 | 0.00 | 0.00 | 0.00 | 0.00 | 0.00 | 0.00 | 0.00 | 0.00 |
| **City** | 6899636 | 0.0000 | 0.0000 | 0.0000 | 0.0000 | 0.0000 | 0.0000 | 256.98 | 0.00 | 0.00 | 0.00 | 0.00 | 0.00 | 0.00 | 0.00 | 0.00 | 0.00 | 0.00 | 0.00 | 0.00 | 0.00 | 0.00 | 0.00 | 0.00 | 0.00 |
| Qixingguan District | 1305066 | 0.0000 | 0.0000 | 0.0000 | 0.0000 | 0.0000 | 0.0000 | 382.34 | 0.00 | 0.00 | 0.00 | 0.00 | 0.00 | 0.00 | 0.00 | 0.00 | 0.00 | 0.00 | 0.00 | 0.00 | 0.00 | 0.00 | 0.00 | 0.00 | 0.00 |
| Dafang County | 857578 | 0.0000 | 0.0000 | 0.0000 | 0.0000 | 0.0000 | 0.0000 | 245.11 | 0.00 | 0.00 | 0.00 | 0.00 | 0.00 | 0.00 | 0.00 | 0.00 | 0.00 | 0.00 | 0.00 | 0.00 | 0.00 | 0.00 | 0.00 | 0.00 | 0.00 |
| Qianxi County | 732008 | 0.0000 | 0.0000 | 0.0000 | 0.0000 | 0.0000 | 0.0000 | 286.44 | 0.00 | 0.00 | 0.00 | 0.00 | 0.00 | 0.00 | 0.00 | 0.00 | 0.00 | 0.00 | 0.00 | 0.00 | 0.00 | 0.00 | 0.00 | 0.00 | 0.00 |
| Jinsha County | 544033 | 0.0000 | 0.0000 | 0.0000 | 0.0000 | 0.0000 | 0.0000 | 215.89 | 0.00 | 0.00 | 0.00 | 0.00 | 0.00 | 0.00 | 0.00 | 0.00 | 0.00 | 0.00 | 0.00 | 0.00 | 0.00 | 0.00 | 0.00 | 0.00 | 0.00 |
| Zhijin County | 815661 | 0.0000 | 0.0000 | 0.0000 | 0.0000 | 0.0000 | 0.0000 | 284.74 | 0.00 | 0.00 | 0.00 | 0.00 | 0.00 | 0.00 | 0.00 | 0.00 | 0.00 | 0.00 | 0.00 | 0.00 | 0.00 | 0.00 | 0.00 | 0.00 | 0.00 |
| Nayong County | 716703 | 0.0000 | 0.0000 | 0.0000 | 0.0000 | 0.0000 | 0.0000 | 291.98 | 0.00 | 0.00 | 0.00 | 0.00 | 0.00 | 0.00 | 0.00 | 0.00 | 0.00 | 0.00 | 0.00 | 0.00 | 0.00 | 0.00 | 0.00 | 0.00 | 0.00 |
| Weining Yi Hui and Miao Autonomous County | 1280116 | 0.0000 | 0.0000 | 0.0000 | 0.0000 | 0.0000 | 0.0000 | 203.19 | 0.00 | 0.00 | 0.00 | 0.00 | 0.00 | 0.00 | 0.00 | 0.00 | 0.00 | 0.00 | 0.00 | 0.00 | 0.00 | 0.00 | 0.00 | 0.00 | 0.00 |
| Hezhang County | 648471 | 0.0000 | 0.0000 | 0.0000 | 0.0000 | 0.0000 | 0.0000 | 200.05 | 0.00 | 0.00 | 0.00 | 0.00 | 0.00 | 0.00 | 0.00 | 0.00 | 0.00 | 0.00 | 0.00 | 0.00 | 0.00 | 0.00 | 0.00 | 0.00 | 0.00 |
| **Tongren City** | 3298468 | 0.0880 | 0.0133 | 0.0000 | 0.0000 | 0.0000 | 0.1013 | 183.13 | 16.12 | 2.44 | 0.00 | 0.00 | 0.00 | 18.56 | 3.15 | 2.44 | 1.14 | 1.05 | 0.08 | 0.06 | 0.03 | 0.24 | 0.02 | 0.04 | 0.00 |
| Bijiang District | 442076 | 0.0813 | 0.0000 | 0.0000 | 0.0000 | 0.0000 | 0.0813 | 437.74 | 35.60 | 0.00 | 0.00 | 0.00 | 0.00 | 35.60 | 5.34 | 4.14 | 1.93 | 1.78 | 0.14 | 0.11 | 0.05 | 0.40 | 0.04 | 0.06 | 0.00 |
| Wanshan District | 160624 | 0.0000 | 0.0000 | 0.0000 | 0.0000 | 0.0000 | 0.0000 | 190.97 | 0.00 | 0.00 | 0.00 | 0.00 | 0.00 | 0.00 | 0.00 | 0.00 | 0.00 | 0.00 | 0.00 | 0.00 | 0.00 | 0.00 | 0.00 | 0.00 | 0.00 |
| Jiangkou County | 184764 | 0.0000 | 0.0000 | 0.0000 | 0.0000 | 0.0000 | 0.0000 | 98.43 | 0.00 | 0.00 | 0.00 | 0.00 | 0.00 | 0.00 | 0.00 | 0.00 | 0.00 | 0.00 | 0.00 | 0.00 | 0.00 | 0.00 | 0.00 | 0.00 | 0.00 |
| Yuping Dong Autonomous County | 150457 | 0.0000 | 0.0133 | 0.0000 | 0.0000 | 0.0000 | 0.0133 | 288.02 | 0.00 | 3.84 | 0.00 | 0.00 | 0.00 | 3.84 | 1.15 | 0.89 | 0.42 | 0.38 | 0.03 | 0.02 | 0.01 | 0.09 | 0.01 | 0.01 | 0.00 |
| Shiqian County | 297086 | 0.0000 | 0.0000 | 0.0000 | 0.0000 | 0.0000 | 0.0000 | 137.04 | 0.00 | 0.00 | 0.00 | 0.00 | 0.00 | 0.00 | 0.00 | 0.00 | 0.00 | 0.00 | 0.00 | 0.00 | 0.00 | 0.00 | 0.00 | 0.00 | 0.00 |
| Sinan County | 457745 | 0.0067 | 0.0000 | 0.0000 | 0.0000 | 0.0000 | 0.0067 | 206.70 | 1.38 | 0.00 | 0.00 | 0.00 | 0.00 | 1.38 | 0.21 | 0.16 | 0.07 | 0.07 | 0.01 | 0.00 | 0.00 | 0.02 | 0.00 | 0.00 | 0.00 |
| Yinjiang Tujia and Miao Autonomous County | 294490 | 0.0000 | 0.0000 | 0.0000 | 0.0000 | 0.0000 | 0.0000 | 149.72 | 0.00 | 0.00 | 0.00 | 0.00 | 0.00 | 0.00 | 0.00 | 0.00 | 0.00 | 0.00 | 0.00 | 0.00 | 0.00 | 0.00 | 0.00 | 0.00 | 0.00 |
| Dejiang County | 393596 | 0.0000 | 0.0000 | 0.0000 | 0.0000 | 0.0000 | 0.0000 | 189.94 | 0.00 | 0.00 | 0.00 | 0.00 | 0.00 | 0.00 | 0.00 | 0.00 | 0.00 | 0.00 | 0.00 | 0.00 | 0.00 | 0.00 | 0.00 | 0.00 | 0.00 |
| Tujia Autonomous County along the river | 429893 | 0.0000 | 0.0000 | 0.0000 | 0.0000 | 0.0000 | 0.0000 | 173.04 | 0.00 | 0.00 | 0.00 | 0.00 | 0.00 | 0.00 | 0.00 | 0.00 | 0.00 | 0.00 | 0.00 | 0.00 | 0.00 | 0.00 | 0.00 | 0.00 | 0.00 |
| Songtao Miao Autonomous County | 487737 | 0.0000 | 0.0000 | 0.0000 | 0.0000 | 0.0000 | 0.0000 | 170.83 | 0.00 | 0.00 | 0.00 | 0.00 | 0.00 | 0.00 | 0.00 | 0.00 | 0.00 | 0.00 | 0.00 | 0.00 | 0.00 | 0.00 | 0.00 | 0.00 | 0.00 |
| **Qianxinan Buyi and Miao Autonomous Prefecture** | 3015112 | 5.8720 | 3.4478 | 0.8765 | 0.6893 | 0.0020 | 10.8877 | 179.45 | 1053.70 | 618.69 | 157.29 | 123.70 | 0.36 | 1953.74 | 537.32 | 416.64 | 194.08 | 179.09 | 14.24 | 10.64 | 5.05 | 40.41 | 4.03 | 6.29 | 0.16 |
| Xingyi City | 1004132 | 3.7787 | 2.7000 | 0.5100 | 0.0000 | 0.0000 | 6.9887 | 345.70 | 1306.30 | 933.40 | 176.31 | 0.00 | 0.00 | 2416.01 | 581.75 | 451.09 | 210.13 | 193.90 | 15.42 | 11.52 | 5.47 | 43.75 | 4.36 | 6.81 | 0.17 |
| Xingren City | 425770 | 0.1667 | 0.2133 | 0.1547 | 0.0300 | 0.0020 | 0.5667 | 239.45 | 39.91 | 51.08 | 37.04 | 7.18 | 0.48 | 135.69 | 49.71 | 38.55 | 17.96 | 16.57 | 1.32 | 0.98 | 0.47 | 3.74 | 0.37 | 0.58 | 0.01 |
| Pu'an County | 242958 | 0.0000 | 0.0000 | 0.0000 | 0.0000 | 0.0000 | 0.0000 | 167.30 | 0.00 | 0.00 | 0.00 | 0.00 | 0.00 | 0.00 | 0.00 | 0.00 | 0.00 | 0.00 | 0.00 | 0.00 | 0.00 | 0.00 | 0.00 | 0.00 | 0.00 |
| Qinglong County | 234162 | 0.0000 | 0.0000 | 0.0000 | 0.0000 | 0.0000 | 0.0000 | 178.89 | 0.00 | 0.00 | 0.00 | 0.00 | 0.00 | 0.00 | 0.00 | 0.00 | 0.00 | 0.00 | 0.00 | 0.00 | 0.00 | 0.00 | 0.00 | 0.00 | 0.00 |
| Zhenfeng County | 307313 | 0.0000 | 0.0000 | 0.0000 | 0.6593 | 0.0000 | 0.6593 | 203.67 | 0.00 | 0.00 | 0.00 | 134.28 | 0.00 | 134.28 | 107.43 | 83.30 | 38.80 | 35.81 | 2.85 | 2.13 | 1.01 | 8.08 | 0.81 | 1.26 | 0.03 |
| Wangmo County | 235243 | 0.0267 | 0.0000 | 0.0033 | 0.0000 | 0.0000 | 0.0300 | 77.91 | 2.08 | 0.00 | 0.26 | 0.00 | 0.00 | 2.34 | 0.47 | 0.36 | 0.17 | 0.16 | 0.01 | 0.01 | 0.00 | 0.04 | 0.00 | 0.01 | 0.00 |
| Ceheng County | 189709 | 0.0000 | 0.2411 | 0.0019 | 0.0000 | 0.0000 | 0.2430 | 73.07 | 0.00 | 17.62 | 0.14 | 0.00 | 0.00 | 17.76 | 5.37 | 4.16 | 1.94 | 1.79 | 0.14 | 0.11 | 0.05 | 0.40 | 0.04 | 0.06 | 0.00 |
| Anlong County | 375825 | 1.9000 | 0.2933 | 0.2067 | 0.0000 | 0.0000 | 2.4000 | 168.24 | 319.65 | 49.35 | 34.77 | 0.00 | 0.00 | 403.77 | 83.61 | 64.83 | 30.20 | 27.87 | 2.22 | 1.66 | 0.79 | 6.29 | 0.63 | 0.98 | 0.03 |
| **Qiandongnan Miao and Dong Autonomous Prefecture** | 3758622 | 1.6300 | 0.3253 | 0.0000 | 0.0000 | 0.0000 | 1.9553 | 124.10 | 202.28 | 40.37 | 0.00 | 0.00 | 0.00 | 242.65 | 42.45 | 32.92 | 15.33 | 14.15 | 1.13 | 0.84 | 0.40 | 3.19 | 0.32 | 0.50 | 0.01 |
| Kaili City | 709057 | 0.3373 | 0.2820 | 0.0000 | 0.0000 | 0.0000 | 0.6193 | 451.43 | 152.28 | 127.30 | 0.00 | 0.00 | 0.00 | 279.58 | 61.03 | 47.32 | 22.05 | 20.34 | 1.62 | 1.21 | 0.57 | 4.59 | 0.46 | 0.71 | 0.02 |
| Huangping County | 244125 | 0.0000 | 0.0000 | 0.0000 | 0.0000 | 0.0000 | 0.0000 | 146.31 | 0.00 | 0.00 | 0.00 | 0.00 | 0.00 | 0.00 | 0.00 | 0.00 | 0.00 | 0.00 | 0.00 | 0.00 | 0.00 | 0.00 | 0.00 | 0.00 | 0.00 |
| Shibing County | 125518 | 0.0000 | 0.0000 | 0.0000 | 0.0000 | 0.0000 | 0.0000 | 82.31 | 0.00 | 0.00 | 0.00 | 0.00 | 0.00 | 0.00 | 0.00 | 0.00 | 0.00 | 0.00 | 0.00 | 0.00 | 0.00 | 0.00 | 0.00 | 0.00 | 0.00 |
| Sansui County | 162798 | 0.0000 | 0.0000 | 0.0000 | 0.0000 | 0.0000 | 0.0000 | 158.06 | 0.00 | 0.00 | 0.00 | 0.00 | 0.00 | 0.00 | 0.00 | 0.00 | 0.00 | 0.00 | 0.00 | 0.00 | 0.00 | 0.00 | 0.00 | 0.00 | 0.00 |
| Zhenyuan County | 189715 | 0.0000 | 0.0000 | 0.0000 | 0.0000 | 0.0000 | 0.0000 | 100.39 | 0.00 | 0.00 | 0.00 | 0.00 | 0.00 | 0.00 | 0.00 | 0.00 | 0.00 | 0.00 | 0.00 | 0.00 | 0.00 | 0.00 | 0.00 | 0.00 | 0.00 |
| Cengong County | 168441 | 0.0000 | 0.0000 | 0.0000 | 0.0000 | 0.0000 | 0.0000 | 113.03 | 0.00 | 0.00 | 0.00 | 0.00 | 0.00 | 0.00 | 0.00 | 0.00 | 0.00 | 0.00 | 0.00 | 0.00 | 0.00 | 0.00 | 0.00 | 0.00 | 0.00 |
| Tianzhu County | 273588 | 0.0000 | 0.0000 | 0.0000 | 0.0000 | 0.0000 | 0.0000 | 125.32 | 0.00 | 0.00 | 0.00 | 0.00 | 0.00 | 0.00 | 0.00 | 0.00 | 0.00 | 0.00 | 0.00 | 0.00 | 0.00 | 0.00 | 0.00 | 0.00 | 0.00 |
| Jinping County | 155182 | 0.0000 | 0.0000 | 0.0000 | 0.0000 | 0.0000 | 0.0000 | 95.98 | 0.00 | 0.00 | 0.00 | 0.00 | 0.00 | 0.00 | 0.00 | 0.00 | 0.00 | 0.00 | 0.00 | 0.00 | 0.00 | 0.00 | 0.00 | 0.00 | 0.00 |
| Jianhe County | 188507 | 0.0067 | 0.0000 | 0.0000 | 0.0000 | 0.0000 | 0.0067 | 86.18 | 0.57 | 0.00 | 0.00 | 0.00 | 0.00 | 0.57 | 0.09 | 0.07 | 0.03 | 0.03 | 0.00 | 0.00 | 0.00 | 0.01 | 0.00 | 0.00 | 0.00 |
| Taijiang County | 122861 | 0.0000 | 0.0000 | 0.0000 | 0.0000 | 0.0000 | 0.0000 | 114.09 | 0.00 | 0.00 | 0.00 | 0.00 | 0.00 | 0.00 | 0.00 | 0.00 | 0.00 | 0.00 | 0.00 | 0.00 | 0.00 | 0.00 | 0.00 | 0.00 | 0.00 |
| Liping County | 412813 | 0.0000 | 0.0433 | 0.0000 | 0.0000 | 0.0000 | 0.0433 | 93.44 | 0.00 | 4.05 | 0.00 | 0.00 | 0.00 | 4.05 | 1.21 | 0.94 | 0.44 | 0.40 | 0.03 | 0.02 | 0.01 | 0.09 | 0.01 | 0.01 | 0.00 |
| Rongjiang County | 297572 | 0.6753 | 0.0000 | 0.0000 | 0.0000 | 0.0000 | 0.6753 | 90.21 | 60.92 | 0.00 | 0.00 | 0.00 | 0.00 | 60.92 | 9.14 | 7.09 | 3.30 | 3.05 | 0.24 | 0.18 | 0.09 | 0.69 | 0.07 | 0.11 | 0.00 |
| Congjiang County | 313887 | 0.6107 | 0.0000 | 0.0000 | 0.0000 | 0.0000 | 0.6107 | 97.34 | 59.44 | 0.00 | 0.00 | 0.00 | 0.00 | 59.44 | 8.92 | 6.91 | 3.22 | 2.97 | 0.24 | 0.18 | 0.08 | 0.67 | 0.07 | 0.10 | 0.00 |
| Leishan County | 124835 | 0.0000 | 0.0000 | 0.0000 | 0.0000 | 0.0000 | 0.0000 | 103.49 | 0.00 | 0.00 | 0.00 | 0.00 | 0.00 | 0.00 | 0.00 | 0.00 | 0.00 | 0.00 | 0.00 | 0.00 | 0.00 | 0.00 | 0.00 | 0.00 | 0.00 |
| Majiang County | 131081 | 0.0000 | 0.0000 | 0.0000 | 0.0000 | 0.0000 | 0.0000 | 136.71 | 0.00 | 0.00 | 0.00 | 0.00 | 0.00 | 0.00 | 0.00 | 0.00 | 0.00 | 0.00 | 0.00 | 0.00 | 0.00 | 0.00 | 0.00 | 0.00 | 0.00 |
| Danzhai County | 138642 | 0.0000 | 0.0000 | 0.0000 | 0.0000 | 0.0000 | 0.0000 | 147.04 | 0.00 | 0.00 | 0.00 | 0.00 | 0.00 | 0.00 | 0.00 | 0.00 | 0.00 | 0.00 | 0.00 | 0.00 | 0.00 | 0.00 | 0.00 | 0.00 | 0.00 |
| **Qiannan Buyi and Miao Autonomous Prefecture** | 3494385 | 0.2740 | 0.0691 | 0.0337 | 0.0144 | 0.0051 | 0.3962 | 133.42 | 36.55 | 9.22 | 4.50 | 1.92 | 0.68 | 52.87 | 13.09 | 10.15 | 4.73 | 4.36 | 0.35 | 0.26 | 0.12 | 0.98 | 0.10 | 0.15 | 0.00 |
| Duyun City | 529688 | 0.0062 | 0.0000 | 0.0000 | 0.0000 | 0.0000 | 0.0062 | 231.61 | 1.44 | 0.00 | 0.00 | 0.00 | 0.00 | 1.44 | 0.22 | 0.17 | 0.08 | 0.07 | 0.01 | 0.00 | 0.00 | 0.02 | 0.00 | 0.00 | 0.00 |
| Fuquan City | 297899 | 0.0021 | 0.0000 | 0.0000 | 0.0000 | 0.0000 | 0.0021 | 176.10 | 0.38 | 0.00 | 0.00 | 0.00 | 0.00 | 0.38 | 0.06 | 0.04 | 0.02 | 0.02 | 0.00 | 0.00 | 0.00 | 0.00 | 0.00 | 0.00 | 0.00 |
| Libo County | 154896 | 0.0660 | 0.0307 | 0.0120 | 0.0080 | 0.0033 | 0.1200 | 64.14 | 4.23 | 1.97 | 0.77 | 0.51 | 0.21 | 7.70 | 2.29 | 1.78 | 0.83 | 0.76 | 0.06 | 0.05 | 0.02 | 0.17 | 0.02 | 0.03 | 0.00 |
| Guiding County | 250146 | 0.0000 | 0.0000 | 0.0000 | 0.0000 | 0.0000 | 0.0000 | 153.62 | 0.00 | 0.00 | 0.00 | 0.00 | 0.00 | 0.00 | 0.00 | 0.00 | 0.00 | 0.00 | 0.00 | 0.00 | 0.00 | 0.00 | 0.00 | 0.00 | 0.00 |
| Weng'an County | 395536 | 0.0000 | 0.0000 | 0.0000 | 0.0000 | 0.0000 | 0.0000 | 201.33 | 0.00 | 0.00 | 0.00 | 0.00 | 0.00 | 0.00 | 0.00 | 0.00 | 0.00 | 0.00 | 0.00 | 0.00 | 0.00 | 0.00 | 0.00 | 0.00 | 0.00 |
| Dushan County | 264266 | 0.0007 | 0.0000 | 0.0000 | 0.0000 | 0.0000 | 0.0007 | 106.64 | 0.07 | 0.00 | 0.00 | 0.00 | 0.00 | 0.07 | 0.01 | 0.01 | 0.00 | 0.00 | 0.00 | 0.00 | 0.00 | 0.00 | 0.00 | 0.00 | 0.00 |
| Pingtang County | 234417 | 0.1540 | 0.0000 | 0.0000 | 0.0000 | 0.0000 | 0.1540 | 83.58 | 12.87 | 0.00 | 0.00 | 0.00 | 0.00 | 12.87 | 1.93 | 1.50 | 0.70 | 0.64 | 0.05 | 0.04 | 0.02 | 0.15 | 0.01 | 0.02 | 0.00 |
| Luodian County | 257551 | 0.0068 | 0.0061 | 0.0058 | 0.0044 | 0.0004 | 0.0235 | 85.54 | 0.59 | 0.52 | 0.49 | 0.37 | 0.04 | 2.01 | 0.87 | 0.68 | 0.31 | 0.29 | 0.02 | 0.02 | 0.01 | 0.07 | 0.01 | 0.01 | 0.00 |
| Changshun County | 201540 | 0.0287 | 0.0180 | 0.0126 | 0.0000 | 0.0000 | 0.0593 | 129.94 | 3.73 | 2.34 | 1.64 | 0.00 | 0.00 | 7.70 | 2.24 | 1.74 | 0.81 | 0.75 | 0.06 | 0.04 | 0.02 | 0.17 | 0.02 | 0.03 | 0.00 |
| Longli County | 236221 | 0.0000 | 0.0000 | 0.0000 | 0.0000 | 0.0000 | 0.0000 | 155.47 | 0.00 | 0.00 | 0.00 | 0.00 | 0.00 | 0.00 | 0.00 | 0.00 | 0.00 | 0.00 | 0.00 | 0.00 | 0.00 | 0.00 | 0.00 | 0.00 | 0.00 |
| Huishui County | 395878 | 0.0067 | 0.0067 | 0.0033 | 0.0020 | 0.0013 | 0.0200 | 160.42 | 1.07 | 1.07 | 0.53 | 0.32 | 0.21 | 3.21 | 1.25 | 0.97 | 0.45 | 0.42 | 0.03 | 0.02 | 0.01 | 0.09 | 0.01 | 0.01 | 0.00 |
| Sandu Aquarium Autonomous County | 276347 | 0.0028 | 0.0077 | 0.0000 | 0.0000 | 0.0000 | 0.0105 | 116.44 | 0.33 | 0.89 | 0.00 | 0.00 | 0.00 | 1.22 | 0.32 | 0.25 | 0.11 | 0.11 | 0.01 | 0.01 | 0.00 | 0.02 | 0.00 | 0.00 | 0.00 |
| **Yunnan Province** | 47209277 | 147.4220 | 89.3300 | 33.0420 | 11.9940 | 2.8033 | 284.5913 | 123.19 | 18161.48 | 11004.91 | 4070.57 | 1477.59 | 345.35 | 35059.90 | 9960.92 | 7723.70 | 3597.89 | 3319.98 | 263.96 | 197.23 | 93.63 | 749.06 | 74.71 | 116.54 | 2.99 |
| **Kunming city** | 8460088 | 9.8193 | 4.6660 | 1.2773 | 0.0667 | 0.0000 | 15.8293 | 402.64 | 3953.61 | 1878.70 | 514.30 | 26.84 | 0.00 | 6373.45 | 1486.70 | 1152.79 | 537.00 | 495.52 | 39.40 | 29.44 | 13.98 | 111.80 | 11.15 | 17.39 | 0.45 |
| Wuhua District | 1143085 | 0.0000 | 0.0000 | 0.0000 | 0.0000 | 0.0000 | 0.0000 | 2985.10 | 0.00 | 0.00 | 0.00 | 0.00 | 0.00 | 0.00 | 0.00 | 0.00 | 0.00 | 0.00 | 0.00 | 0.00 | 0.00 | 0.00 | 0.00 | 0.00 | 0.00 |
| Panlong District | 987955 | 0.0000 | 0.0000 | 0.0000 | 0.0000 | 0.0000 | 0.0000 | 1147.00 | 0.00 | 0.00 | 0.00 | 0.00 | 0.00 | 0.00 | 0.00 | 0.00 | 0.00 | 0.00 | 0.00 | 0.00 | 0.00 | 0.00 | 0.00 | 0.00 | 0.00 |
| Guandu District | 1602279 | 0.0000 | 0.0000 | 0.0000 | 0.0000 | 0.0000 | 0.0000 | 2528.13 | 0.00 | 0.00 | 0.00 | 0.00 | 0.00 | 0.00 | 0.00 | 0.00 | 0.00 | 0.00 | 0.00 | 0.00 | 0.00 | 0.00 | 0.00 | 0.00 | 0.00 |
| Xishan District | 960746 | 0.5700 | 0.0000 | 0.0000 | 0.0000 | 0.0000 | 0.5700 | 1093.71 | 623.41 | 0.00 | 0.00 | 0.00 | 0.00 | 623.41 | 93.51 | 72.51 | 33.78 | 31.17 | 2.48 | 1.85 | 0.88 | 7.03 | 0.70 | 1.09 | 0.03 |
| Dongchuan District | 260744 | 5.9307 | 4.0480 | 1.2613 | 0.0667 | 0.0000 | 11.3067 | 139.45 | 827.03 | 564.49 | 175.89 | 9.30 | 0.00 | 1576.72 | 406.38 | 315.10 | 146.78 | 135.45 | 10.77 | 8.05 | 3.82 | 30.56 | 3.05 | 4.75 | 0.12 |
| Chenggong District | 649501 | 0.6740 | 0.3307 | 0.0160 | 0.0000 | 0.0000 | 1.0207 | 1275.28 | 859.54 | 421.69 | 20.40 | 0.00 | 0.00 | 1301.64 | 267.68 | 207.56 | 96.69 | 89.22 | 7.09 | 5.30 | 2.52 | 20.13 | 2.01 | 3.13 | 0.08 |
| Jinning District | 346268 | 0.0493 | 0.0000 | 0.0000 | 0.0000 | 0.0000 | 0.0493 | 259.53 | 12.80 | 0.00 | 0.00 | 0.00 | 0.00 | 12.80 | 1.92 | 1.49 | 0.69 | 0.64 | 0.05 | 0.04 | 0.02 | 0.14 | 0.01 | 0.02 | 0.00 |
| Fumin County | 149506 | 1.1867 | 0.2800 | 0.0000 | 0.0000 | 0.0000 | 1.4667 | 150.05 | 178.06 | 42.02 | 0.00 | 0.00 | 0.00 | 220.08 | 39.31 | 30.48 | 14.20 | 13.10 | 1.04 | 0.78 | 0.37 | 2.96 | 0.29 | 0.46 | 0.01 |
| Yiliang County | 384875 | 0.0520 | 0.0000 | 0.0000 | 0.0000 | 0.0000 | 0.0520 | 201.32 | 10.47 | 0.00 | 0.00 | 0.00 | 0.00 | 10.47 | 1.57 | 1.22 | 0.57 | 0.52 | 0.04 | 0.03 | 0.01 | 0.12 | 0.01 | 0.02 | 0.00 |
| Shilin Yi Autonomous County | 240827 | 0.8000 | 0.0000 | 0.0000 | 0.0000 | 0.0000 | 0.8000 | 143.32 | 114.66 | 0.00 | 0.00 | 0.00 | 0.00 | 114.66 | 17.20 | 13.34 | 6.21 | 5.73 | 0.46 | 0.34 | 0.16 | 1.29 | 0.13 | 0.20 | 0.01 |
| Songming County | 410929 | 0.5293 | 0.0000 | 0.0000 | 0.0000 | 0.0000 | 0.5293 | 495.12 | 262.08 | 0.00 | 0.00 | 0.00 | 0.00 | 262.08 | 39.31 | 30.48 | 14.20 | 13.10 | 1.04 | 0.78 | 0.37 | 2.96 | 0.29 | 0.46 | 0.01 |
| Luquan Yi and Miao Autonomous County | 378881 | 0.0133 | 0.0067 | 0.0000 | 0.0000 | 0.0000 | 0.0200 | 89.47 | 1.19 | 0.60 | 0.00 | 0.00 | 0.00 | 1.79 | 0.36 | 0.28 | 0.13 | 0.12 | 0.01 | 0.01 | 0.00 | 0.03 | 0.00 | 0.00 | 0.00 |
| Xundian Hui Yi Autonomous County | 460739 | 0.0000 | 0.0000 | 0.0000 | 0.0000 | 0.0000 | 0.0000 | 128.50 | 0.00 | 0.00 | 0.00 | 0.00 | 0.00 | 0.00 | 0.00 | 0.00 | 0.00 | 0.00 | 0.00 | 0.00 | 0.00 | 0.00 | 0.00 | 0.00 | 0.00 |
| Anning City | 483753 | 0.0140 | 0.0007 | 0.0000 | 0.0000 | 0.0000 | 0.0147 | 371.15 | 5.20 | 0.25 | 0.00 | 0.00 | 0.00 | 5.44 | 0.85 | 0.66 | 0.31 | 0.28 | 0.02 | 0.02 | 0.01 | 0.06 | 0.01 | 0.01 | 0.00 |
| **Qujing City** | 5765775 | 2.8613 | 1.3373 | 0.4880 | 0.0000 | 0.0000 | 4.6867 | 199.34 | 570.37 | 266.58 | 97.28 | 0.00 | 0.00 | 934.23 | 223.90 | 173.61 | 80.87 | 74.62 | 5.93 | 4.43 | 2.10 | 16.84 | 1.68 | 2.62 | 0.07 |
| Qilin District | 996279 | 1.2413 | 0.9707 | 0.3547 | 0.0000 | 0.0000 | 2.5667 | 645.34 | 801.08 | 626.41 | 228.88 | 0.00 | 0.00 | 1656.38 | 445.42 | 345.37 | 160.88 | 148.46 | 11.80 | 8.82 | 4.19 | 33.50 | 3.34 | 5.21 | 0.13 |
| Zhan Yi District | 405305 | 1.6200 | 0.3667 | 0.1333 | 0.0000 | 0.0000 | 2.1200 | 143.91 | 233.13 | 52.77 | 19.19 | 0.00 | 0.00 | 305.09 | 62.31 | 48.32 | 22.51 | 20.77 | 1.65 | 1.23 | 0.59 | 4.69 | 0.47 | 0.73 | 0.02 |
| Malone District | 193137 | 0.0000 | 0.0000 | 0.0000 | 0.0000 | 0.0000 | 0.0000 | 120.70 | 0.00 | 0.00 | 0.00 | 0.00 | 0.00 | 0.00 | 0.00 | 0.00 | 0.00 | 0.00 | 0.00 | 0.00 | 0.00 | 0.00 | 0.00 | 0.00 | 0.00 |
| Luliang County | 599266 | 0.0000 | 0.0000 | 0.0000 | 0.0000 | 0.0000 | 0.0000 | 301.00 | 0.00 | 0.00 | 0.00 | 0.00 | 0.00 | 0.00 | 0.00 | 0.00 | 0.00 | 0.00 | 0.00 | 0.00 | 0.00 | 0.00 | 0.00 | 0.00 | 0.00 |
| Shizong County | 376902 | 0.0000 | 0.0000 | 0.0000 | 0.0000 | 0.0000 | 0.0000 | 136.13 | 0.00 | 0.00 | 0.00 | 0.00 | 0.00 | 0.00 | 0.00 | 0.00 | 0.00 | 0.00 | 0.00 | 0.00 | 0.00 | 0.00 | 0.00 | 0.00 | 0.00 |
| Luoping County | 535565 | 0.0000 | 0.0000 | 0.0000 | 0.0000 | 0.0000 | 0.0000 | 177.81 | 0.00 | 0.00 | 0.00 | 0.00 | 0.00 | 0.00 | 0.00 | 0.00 | 0.00 | 0.00 | 0.00 | 0.00 | 0.00 | 0.00 | 0.00 | 0.00 | 0.00 |
| Fuyuan County | 675229 | 0.0000 | 0.0000 | 0.0000 | 0.0000 | 0.0000 | 0.0000 | 207.21 | 0.00 | 0.00 | 0.00 | 0.00 | 0.00 | 0.00 | 0.00 | 0.00 | 0.00 | 0.00 | 0.00 | 0.00 | 0.00 | 0.00 | 0.00 | 0.00 | 0.00 |
| Huize County | 794279 | 0.0000 | 0.0000 | 0.0000 | 0.0000 | 0.0000 | 0.0000 | 134.99 | 0.00 | 0.00 | 0.00 | 0.00 | 0.00 | 0.00 | 0.00 | 0.00 | 0.00 | 0.00 | 0.00 | 0.00 | 0.00 | 0.00 | 0.00 | 0.00 | 0.00 |
| Xuanwei City | 1189813 | 0.0000 | 0.0000 | 0.0000 | 0.0000 | 0.0000 | 0.0000 | 196.67 | 0.00 | 0.00 | 0.00 | 0.00 | 0.00 | 0.00 | 0.00 | 0.00 | 0.00 | 0.00 | 0.00 | 0.00 | 0.00 | 0.00 | 0.00 | 0.00 | 0.00 |
| **Yuxi City** | 2249502 | 10.1520 | 7.6073 | 3.2220 | 0.5527 | 0.0393 | 21.5733 | 150.53 | 1528.23 | 1145.17 | 485.02 | 83.20 | 5.92 | 3247.53 | 935.68 | 725.53 | 337.97 | 311.86 | 24.80 | 18.53 | 8.80 | 70.36 | 7.02 | 10.95 | 0.28 |
| Hongta District | 588738 | 0.9233 | 3.3707 | 0.2920 | 0.0013 | 0.0000 | 4.5873 | 620.81 | 573.21 | 2092.54 | 181.28 | 0.83 | 0.00 | 2847.86 | 823.17 | 638.29 | 297.33 | 274.36 | 21.81 | 16.30 | 7.74 | 61.90 | 6.17 | 9.63 | 0.25 |
| Jiangchuan District | 253295 | 2.8407 | 0.3580 | 0.0153 | 0.0000 | 0.0000 | 3.2140 | 312.92 | 888.91 | 112.03 | 4.80 | 0.00 | 0.00 | 1005.73 | 169.82 | 131.68 | 61.34 | 56.60 | 4.50 | 3.36 | 1.60 | 12.77 | 1.27 | 1.99 | 0.05 |
| Tonghai County | 289891 | 0.0000 | 0.0000 | 0.0000 | 0.0000 | 0.0000 | 0.0000 | 391.90 | 0.00 | 0.00 | 0.00 | 0.00 | 0.00 | 0.00 | 0.00 | 0.00 | 0.00 | 0.00 | 0.00 | 0.00 | 0.00 | 0.00 | 0.00 | 0.00 | 0.00 |
| Huaning County | 190425 | 2.1800 | 0.9767 | 0.5780 | 0.1920 | 0.0100 | 3.9367 | 152.55 | 332.55 | 148.99 | 88.17 | 29.29 | 1.53 | 600.53 | 172.29 | 133.59 | 62.23 | 57.42 | 4.57 | 3.41 | 1.62 | 12.96 | 1.29 | 2.02 | 0.05 |
| Yimen County | 151671 | 0.6500 | 0.0000 | 0.0000 | 0.0000 | 0.0000 | 0.6500 | 99.27 | 64.53 | 0.00 | 0.00 | 0.00 | 0.00 | 64.53 | 9.68 | 7.51 | 3.50 | 3.23 | 0.26 | 0.19 | 0.09 | 0.73 | 0.07 | 0.11 | 0.00 |
| Eshan Yi Autonomous County | 143903 | 2.3233 | 0.0000 | 0.0000 | 0.0000 | 0.0000 | 2.3233 | 74.58 | 173.28 | 0.00 | 0.00 | 0.00 | 0.00 | 173.28 | 25.99 | 20.15 | 9.39 | 8.66 | 0.69 | 0.51 | 0.24 | 1.95 | 0.19 | 0.30 | 0.01 |
| Xinping Yi Dai Autonomous County | 262771 | 0.1907 | 0.2160 | 0.0000 | 0.0000 | 0.0000 | 0.4067 | 61.60 | 11.75 | 13.31 | 0.00 | 0.00 | 0.00 | 25.05 | 5.75 | 4.46 | 2.08 | 1.92 | 0.15 | 0.11 | 0.05 | 0.43 | 0.04 | 0.07 | 0.00 |
| Yuanjiang Hani Yi Dai Autonomous County | 195647 | 0.1613 | 0.7893 | 0.5280 | 0.0913 | 0.0040 | 1.5740 | 71.97 | 11.61 | 56.81 | 38.00 | 6.57 | 0.29 | 113.28 | 47.10 | 36.52 | 17.01 | 15.70 | 1.25 | 0.93 | 0.44 | 3.54 | 0.35 | 0.55 | 0.01 |
| Chengjiang City | 173161 | 0.8827 | 1.8967 | 1.8087 | 0.2680 | 0.0253 | 4.8813 | 228.98 | 202.11 | 434.29 | 414.14 | 61.37 | 5.80 | 1117.71 | 463.40 | 359.32 | 167.38 | 154.45 | 12.28 | 9.18 | 4.36 | 34.85 | 3.48 | 5.42 | 0.14 |
| **Baoshan City** | 2431211 | 4.1600 | 2.8007 | 0.9840 | 0.1533 | 0.0020 | 8.1000 | 127.46 | 530.23 | 356.97 | 125.42 | 19.54 | 0.25 | 1032.42 | 277.74 | 215.36 | 100.32 | 92.57 | 7.36 | 5.50 | 2.61 | 20.89 | 2.08 | 3.25 | 0.08 |
| Longyang District | 903081 | 1.4367 | 0.1633 | 0.0440 | 0.0187 | 0.0020 | 1.6647 | 186.42 | 267.82 | 30.45 | 8.20 | 3.48 | 0.37 | 310.32 | 57.35 | 44.47 | 20.71 | 19.11 | 1.52 | 1.14 | 0.54 | 4.31 | 0.43 | 0.67 | 0.02 |
| Shidian County | 293022 | 1.8367 | 0.4240 | 0.0867 | 0.0193 | 0.0000 | 2.3667 | 149.81 | 275.16 | 63.52 | 12.98 | 2.90 | 0.00 | 354.56 | 70.44 | 54.62 | 25.44 | 23.48 | 1.87 | 1.39 | 0.66 | 5.30 | 0.53 | 0.82 | 0.02 |
| Longling County | 272769 | 0.0000 | 0.0000 | 0.0000 | 0.0000 | 0.0000 | 0.0000 | 97.57 | 0.00 | 0.00 | 0.00 | 0.00 | 0.00 | 0.00 | 0.00 | 0.00 | 0.00 | 0.00 | 0.00 | 0.00 | 0.00 | 0.00 | 0.00 | 0.00 | 0.00 |
| Changning County | 319858 | 0.0200 | 0.0133 | 0.0000 | 0.0000 | 0.0000 | 0.0333 | 84.91 | 1.70 | 1.13 | 0.00 | 0.00 | 0.00 | 2.83 | 0.59 | 0.46 | 0.21 | 0.20 | 0.02 | 0.01 | 0.01 | 0.04 | 0.00 | 0.01 | 0.00 |
| Tengchong City | 642481 | 0.8667 | 2.2000 | 0.8533 | 0.1153 | 0.0000 | 4.0353 | 112.49 | 97.50 | 247.49 | 96.00 | 12.97 | 0.00 | 453.95 | 156.85 | 121.62 | 56.65 | 52.28 | 4.16 | 3.11 | 1.47 | 11.79 | 1.18 | 1.84 | 0.05 |
| **Zhaotong City** | 5092611 | 0.2413 | 0.0000 | 0.0000 | 0.0000 | 0.0000 | 0.2413 | 226.89 | 54.76 | 0.00 | 0.00 | 0.00 | 0.00 | 54.76 | 8.21 | 6.37 | 2.97 | 2.74 | 0.22 | 0.16 | 0.08 | 0.62 | 0.06 | 0.10 | 0.00 |
| Zhaoyang District | 911766 | 0.0000 | 0.0000 | 0.0000 | 0.0000 | 0.0000 | 0.0000 | 421.39 | 0.00 | 0.00 | 0.00 | 0.00 | 0.00 | 0.00 | 0.00 | 0.00 | 0.00 | 0.00 | 0.00 | 0.00 | 0.00 | 0.00 | 0.00 | 0.00 | 0.00 |
| Ludian County | 398447 | 0.0000 | 0.0000 | 0.0000 | 0.0000 | 0.0000 | 0.0000 | 268.53 | 0.00 | 0.00 | 0.00 | 0.00 | 0.00 | 0.00 | 0.00 | 0.00 | 0.00 | 0.00 | 0.00 | 0.00 | 0.00 | 0.00 | 0.00 | 0.00 | 0.00 |
| Qiaojia County | 462173 | 0.0947 | 0.0000 | 0.0000 | 0.0000 | 0.0000 | 0.0947 | 144.53 | 13.68 | 0.00 | 0.00 | 0.00 | 0.00 | 13.68 | 2.05 | 1.59 | 0.74 | 0.68 | 0.05 | 0.04 | 0.02 | 0.15 | 0.02 | 0.02 | 0.00 |
| Yanjin County | 317463 | 0.0000 | 0.0000 | 0.0000 | 0.0000 | 0.0000 | 0.0000 | 156.59 | 0.00 | 0.00 | 0.00 | 0.00 | 0.00 | 0.00 | 0.00 | 0.00 | 0.00 | 0.00 | 0.00 | 0.00 | 0.00 | 0.00 | 0.00 | 0.00 | 0.00 |
| Daguan County | 209116 | 0.0000 | 0.0000 | 0.0000 | 0.0000 | 0.0000 | 0.0000 | 121.50 | 0.00 | 0.00 | 0.00 | 0.00 | 0.00 | 0.00 | 0.00 | 0.00 | 0.00 | 0.00 | 0.00 | 0.00 | 0.00 | 0.00 | 0.00 | 0.00 | 0.00 |
| Yongshan County | 349157 | 0.0000 | 0.0000 | 0.0000 | 0.0000 | 0.0000 | 0.0000 | 125.74 | 0.00 | 0.00 | 0.00 | 0.00 | 0.00 | 0.00 | 0.00 | 0.00 | 0.00 | 0.00 | 0.00 | 0.00 | 0.00 | 0.00 | 0.00 | 0.00 | 0.00 |
| Suijiang County | 135468 | 0.0000 | 0.0000 | 0.0000 | 0.0000 | 0.0000 | 0.0000 | 180.92 | 0.00 | 0.00 | 0.00 | 0.00 | 0.00 | 0.00 | 0.00 | 0.00 | 0.00 | 0.00 | 0.00 | 0.00 | 0.00 | 0.00 | 0.00 | 0.00 | 0.00 |
| Zhenxiong County | 1349795 | 0.0000 | 0.0000 | 0.0000 | 0.0000 | 0.0000 | 0.0000 | 365.21 | 0.00 | 0.00 | 0.00 | 0.00 | 0.00 | 0.00 | 0.00 | 0.00 | 0.00 | 0.00 | 0.00 | 0.00 | 0.00 | 0.00 | 0.00 | 0.00 | 0.00 |
| Yiliang County | 503376 | 0.1467 | 0.0000 | 0.0000 | 0.0000 | 0.0000 | 0.1467 | 179.91 | 26.39 | 0.00 | 0.00 | 0.00 | 0.00 | 26.39 | 3.96 | 3.07 | 1.43 | 1.32 | 0.10 | 0.08 | 0.04 | 0.30 | 0.03 | 0.05 | 0.00 |
| Wilson County | 352318 | 0.0000 | 0.0000 | 0.0000 | 0.0000 | 0.0000 | 0.0000 | 252.85 | 0.00 | 0.00 | 0.00 | 0.00 | 0.00 | 0.00 | 0.00 | 0.00 | 0.00 | 0.00 | 0.00 | 0.00 | 0.00 | 0.00 | 0.00 | 0.00 | 0.00 |
| Mizutomi City | 103532 | 0.0000 | 0.0000 | 0.0000 | 0.0000 | 0.0000 | 0.0000 | 236.17 | 0.00 | 0.00 | 0.00 | 0.00 | 0.00 | 0.00 | 0.00 | 0.00 | 0.00 | 0.00 | 0.00 | 0.00 | 0.00 | 0.00 | 0.00 | 0.00 | 0.00 |
| **Lijiang City** | 1253878 | 2.1173 | 0.2487 | 0.0000 | 0.0000 | 0.0000 | 2.3660 | 60.99 | 129.13 | 15.17 | 0.00 | 0.00 | 0.00 | 144.30 | 23.92 | 18.55 | 8.64 | 7.97 | 0.63 | 0.47 | 0.22 | 1.80 | 0.18 | 0.28 | 0.01 |
| Gucheng City | 288787 | 0.0000 | 0.0000 | 0.0000 | 0.0000 | 0.0000 | 0.0000 | 228.24 | 0.00 | 0.00 | 0.00 | 0.00 | 0.00 | 0.00 | 0.00 | 0.00 | 0.00 | 0.00 | 0.00 | 0.00 | 0.00 | 0.00 | 0.00 | 0.00 | 0.00 |
| Yulong Naxi Autonomous County | 224039 | 0.9440 | 0.1553 | 0.0000 | 0.0000 | 0.0000 | 1.0993 | 36.14 | 34.12 | 5.61 | 0.00 | 0.00 | 0.00 | 39.73 | 6.80 | 5.27 | 2.46 | 2.27 | 0.18 | 0.13 | 0.06 | 0.51 | 0.05 | 0.08 | 0.00 |
| Yongsheng County | 336832 | 0.8000 | 0.0000 | 0.0000 | 0.0000 | 0.0000 | 0.8000 | 68.36 | 54.68 | 0.00 | 0.00 | 0.00 | 0.00 | 54.68 | 8.20 | 6.36 | 2.96 | 2.73 | 0.22 | 0.16 | 0.08 | 0.62 | 0.06 | 0.10 | 0.00 |
| Huaping County | 159695 | 0.0533 | 0.0933 | 0.0000 | 0.0000 | 0.0000 | 0.1467 | 74.57 | 3.98 | 6.96 | 0.00 | 0.00 | 0.00 | 10.94 | 2.68 | 2.08 | 0.97 | 0.89 | 0.07 | 0.05 | 0.03 | 0.20 | 0.02 | 0.03 | 0.00 |
| Ninglang Yi Autonomous County | 244525 | 0.3200 | 0.0000 | 0.0000 | 0.0000 | 0.0000 | 0.3200 | 40.58 | 12.99 | 0.00 | 0.00 | 0.00 | 0.00 | 12.99 | 1.95 | 1.51 | 0.70 | 0.65 | 0.05 | 0.04 | 0.02 | 0.15 | 0.01 | 0.02 | 0.00 |
| **Pu'er City** | 2404954 | 21.9720 | 3.0480 | 0.7420 | 0.1547 | 0.0000 | 25.9167 | 54.32 | 1193.43 | 165.55 | 40.30 | 8.40 | 0.00 | 1407.68 | 259.58 | 201.28 | 93.76 | 86.52 | 6.88 | 5.14 | 2.44 | 19.52 | 1.95 | 3.04 | 0.08 |
| Simao District | 416188 | 11.3333 | 0.5333 | 0.0667 | 0.0000 | 0.0000 | 11.9333 | 107.44 | 1217.68 | 57.30 | 7.16 | 0.00 | 0.00 | 1282.14 | 204.14 | 158.29 | 73.74 | 68.04 | 5.41 | 4.04 | 1.92 | 15.35 | 1.53 | 2.39 | 0.06 |
| Ning'er Hani Yi Autonomous County | 162711 | 2.0000 | 0.0000 | 0.0000 | 0.0000 | 0.0000 | 2.0000 | 44.35 | 88.70 | 0.00 | 0.00 | 0.00 | 0.00 | 88.70 | 13.30 | 10.32 | 4.81 | 4.43 | 0.35 | 0.26 | 0.13 | 1.00 | 0.10 | 0.16 | 0.00 |
| Mojiang Hani Autonomous County | 281554 | 0.1320 | 0.0000 | 0.0000 | 0.0000 | 0.0000 | 0.1320 | 53.22 | 7.03 | 0.00 | 0.00 | 0.00 | 0.00 | 7.03 | 1.05 | 0.82 | 0.38 | 0.35 | 0.03 | 0.02 | 0.01 | 0.08 | 0.01 | 0.01 | 0.00 |
| Jingdong Yi Autonomous County | 303109 | 1.5867 | 0.6800 | 0.0000 | 0.0000 | 0.0000 | 2.2667 | 68.02 | 107.92 | 46.25 | 0.00 | 0.00 | 0.00 | 154.17 | 30.06 | 23.31 | 10.86 | 10.02 | 0.80 | 0.60 | 0.28 | 2.26 | 0.23 | 0.35 | 0.01 |
| Jinggu Dai Yi Autonomous County | 277417 | 0.7000 | 0.0000 | 0.0000 | 0.0000 | 0.0000 | 0.7000 | 36.89 | 25.82 | 0.00 | 0.00 | 0.00 | 0.00 | 25.82 | 3.87 | 3.00 | 1.40 | 1.29 | 0.10 | 0.08 | 0.04 | 0.29 | 0.03 | 0.05 | 0.00 |
| Zhenyuan Yi Hani Lahu Autonomous County | 179503 | 0.6667 | 0.4333 | 0.2333 | 0.0000 | 0.0000 | 1.3333 | 43.30 | 28.87 | 18.76 | 10.10 | 0.00 | 0.00 | 57.74 | 16.02 | 12.42 | 5.79 | 5.34 | 0.42 | 0.32 | 0.15 | 1.20 | 0.12 | 0.19 | 0.00 |
| Jiangcheng Hani Yi Autonomous County | 111033 | 3.3800 | 0.3533 | 0.0000 | 0.0000 | 0.0000 | 3.7333 | 32.42 | 109.57 | 11.45 | 0.00 | 0.00 | 0.00 | 121.02 | 19.87 | 15.41 | 7.18 | 6.62 | 0.53 | 0.39 | 0.19 | 1.49 | 0.15 | 0.23 | 0.01 |
| Menglian Dai Lahu Wa Autonomous County | 144693 | 0.6133 | 0.2400 | 0.1000 | 0.0000 | 0.0000 | 0.9533 | 76.16 | 46.71 | 18.28 | 7.62 | 0.00 | 0.00 | 72.61 | 17.06 | 13.23 | 6.16 | 5.69 | 0.45 | 0.34 | 0.16 | 1.28 | 0.13 | 0.20 | 0.01 |
| Lancang Lahu Autonomous County | 441455 | 1.5600 | 0.8080 | 0.3420 | 0.1547 | 0.0000 | 2.8647 | 50.51 | 78.79 | 40.81 | 17.27 | 7.81 | 0.00 | 144.69 | 40.67 | 31.54 | 14.69 | 13.56 | 1.08 | 0.81 | 0.38 | 3.06 | 0.31 | 0.48 | 0.01 |
| Ximeng Wa Autonomous County | 87291 | 0.0000 | 0.0000 | 0.0000 | 0.0000 | 0.0000 | 0.0000 | 69.42 | 0.00 | 0.00 | 0.00 | 0.00 | 0.00 | 0.00 | 0.00 | 0.00 | 0.00 | 0.00 | 0.00 | 0.00 | 0.00 | 0.00 | 0.00 | 0.00 | 0.00 |
| **Lincang City** | 2257991 | 9.8213 | 6.0473 | 1.9253 | 1.2000 | 0.5333 | 19.5273 | 95.36 | 936.59 | 576.69 | 183.60 | 114.43 | 50.86 | 1862.17 | 560.98 | 434.98 | 202.63 | 186.97 | 14.87 | 11.11 | 5.27 | 42.19 | 4.21 | 6.56 | 0.17 |
| Linxiang District | 370947 | 1.1900 | 0.1340 | 0.0987 | 0.0000 | 0.0000 | 1.4227 | 144.97 | 172.52 | 19.43 | 14.30 | 0.00 | 0.00 | 206.25 | 40.29 | 31.24 | 14.55 | 13.43 | 1.07 | 0.80 | 0.38 | 3.03 | 0.30 | 0.47 | 0.01 |
| Fengqing County | 385420 | 0.3080 | 0.1167 | 0.0000 | 0.0000 | 0.0000 | 0.4247 | 115.95 | 35.71 | 13.53 | 0.00 | 0.00 | 0.00 | 49.24 | 9.42 | 7.30 | 3.40 | 3.14 | 0.25 | 0.19 | 0.09 | 0.71 | 0.07 | 0.11 | 0.00 |
| Cloud County | 389180 | 5.6967 | 0.9667 | 0.0267 | 0.0000 | 0.0000 | 6.6900 | 106.37 | 605.94 | 102.82 | 2.84 | 0.00 | 0.00 | 711.60 | 123.44 | 95.72 | 44.59 | 41.14 | 3.27 | 2.44 | 1.16 | 9.28 | 0.93 | 1.44 | 0.04 |
| Yongde County | 328864 | 0.0000 | 0.2000 | 0.0000 | 0.0000 | 0.0000 | 0.2000 | 102.11 | 0.00 | 20.42 | 0.00 | 0.00 | 0.00 | 20.42 | 6.13 | 4.75 | 2.21 | 2.04 | 0.16 | 0.12 | 0.06 | 0.46 | 0.05 | 0.07 | 0.00 |
| Zhenkang County | 172879 | 0.0267 | 0.0133 | 0.0000 | 0.0000 | 0.0000 | 0.0400 | 67.99 | 1.81 | 0.91 | 0.00 | 0.00 | 0.00 | 2.72 | 0.54 | 0.42 | 0.20 | 0.18 | 0.01 | 0.01 | 0.01 | 0.04 | 0.00 | 0.01 | 0.00 |
| Shuangjiang Lahu, Wa, Bulang, Dai Autonomous County | 164756 | 1.4000 | 2.2000 | 1.7333 | 1.2000 | 0.5333 | 7.0667 | 76.25 | 106.75 | 167.74 | 132.16 | 91.50 | 40.67 | 538.82 | 255.43 | 198.06 | 92.26 | 85.13 | 6.77 | 5.06 | 2.40 | 19.21 | 1.92 | 2.99 | 0.08 |
| Gengma Dai Wa Autonomous County | 285683 | 0.0000 | 2.1500 | 0.0000 | 0.0000 | 0.0000 | 2.1500 | 76.41 | 0.00 | 164.29 | 0.00 | 0.00 | 0.00 | 164.29 | 49.29 | 38.22 | 17.80 | 16.43 | 1.31 | 0.98 | 0.46 | 3.71 | 0.37 | 0.58 | 0.01 |
| Cangyuan Wa Autonomous County | 160262 | 1.2000 | 0.2667 | 0.0667 | 0.0000 | 0.0000 | 1.5333 | 64.79 | 77.74 | 17.28 | 4.32 | 0.00 | 0.00 | 99.34 | 19.44 | 15.07 | 7.02 | 6.48 | 0.52 | 0.38 | 0.18 | 1.46 | 0.15 | 0.23 | 0.01 |
| **Chuxiong Yi Autonomous Prefecture** | 2416747 | 16.4700 | 2.0667 | 0.5747 | 0.0000 | 0.0000 | 19.1113 | 84.98 | 1399.68 | 175.63 | 48.84 | 0.00 | 0.00 | 1624.15 | 291.94 | 226.37 | 105.45 | 97.31 | 7.74 | 5.78 | 2.74 | 21.95 | 2.19 | 3.42 | 0.09 |
| Chuxiong City | 631530 | 1.3333 | 0.6667 | 0.0667 | 0.0000 | 0.0000 | 2.0667 | 142.43 | 189.91 | 94.95 | 9.50 | 0.00 | 0.00 | 294.35 | 62.67 | 48.59 | 22.64 | 20.89 | 1.66 | 1.24 | 0.59 | 4.71 | 0.47 | 0.73 | 0.02 |
| Shuangbai County | 133884 | 0.0000 | 0.0000 | 0.0000 | 0.0000 | 0.0000 | 0.0000 | 34.40 | 0.00 | 0.00 | 0.00 | 0.00 | 0.00 | 0.00 | 0.00 | 0.00 | 0.00 | 0.00 | 0.00 | 0.00 | 0.00 | 0.00 | 0.00 | 0.00 | 0.00 |
| Muding County | 149437 | 5.0667 | 0.9333 | 0.3213 | 0.0000 | 0.0000 | 6.3213 | 102.88 | 521.27 | 96.02 | 33.06 | 0.00 | 0.00 | 650.36 | 126.83 | 98.35 | 45.81 | 42.27 | 3.36 | 2.51 | 1.19 | 9.54 | 0.95 | 1.48 | 0.04 |
| Nanhua County | 203704 | 0.0000 | 0.0000 | 0.0000 | 0.0000 | 0.0000 | 0.0000 | 89.92 | 0.00 | 0.00 | 0.00 | 0.00 | 0.00 | 0.00 | 0.00 | 0.00 | 0.00 | 0.00 | 0.00 | 0.00 | 0.00 | 0.00 | 0.00 | 0.00 | 0.00 |
| Yao'an County | 164165 | 0.0000 | 0.0000 | 0.0000 | 0.0000 | 0.0000 | 0.0000 | 96.61 | 0.00 | 0.00 | 0.00 | 0.00 | 0.00 | 0.00 | 0.00 | 0.00 | 0.00 | 0.00 | 0.00 | 0.00 | 0.00 | 0.00 | 0.00 | 0.00 | 0.00 |
| Dayao County | 228961 | 0.0000 | 0.0000 | 0.0000 | 0.0000 | 0.0000 | 0.0000 | 56.85 | 0.00 | 0.00 | 0.00 | 0.00 | 0.00 | 0.00 | 0.00 | 0.00 | 0.00 | 0.00 | 0.00 | 0.00 | 0.00 | 0.00 | 0.00 | 0.00 | 0.00 |
| Yongren County | 97985 | 0.8667 | 0.3467 | 0.1867 | 0.0000 | 0.0000 | 1.4000 | 45.55 | 39.48 | 15.79 | 8.50 | 0.00 | 0.00 | 63.77 | 15.76 | 12.22 | 5.69 | 5.25 | 0.42 | 0.31 | 0.15 | 1.19 | 0.12 | 0.18 | 0.00 |
| Yuanmou County | 201510 | 6.6667 | 0.0000 | 0.0000 | 0.0000 | 0.0000 | 6.6667 | 99.58 | 663.87 | 0.00 | 0.00 | 0.00 | 0.00 | 663.87 | 99.58 | 77.21 | 35.97 | 33.19 | 2.64 | 1.97 | 0.94 | 7.49 | 0.75 | 1.17 | 0.03 |
| Wuding County | 239059 | 1.3333 | 0.0000 | 0.0000 | 0.0000 | 0.0000 | 1.3333 | 81.12 | 108.16 | 0.00 | 0.00 | 0.00 | 0.00 | 108.16 | 16.22 | 12.58 | 5.86 | 5.41 | 0.43 | 0.32 | 0.15 | 1.22 | 0.12 | 0.19 | 0.00 |
| Lufeng County | 366512 | 1.2033 | 0.1200 | 0.0000 | 0.0000 | 0.0000 | 1.3233 | 103.38 | 124.40 | 12.41 | 0.00 | 0.00 | 0.00 | 136.81 | 22.38 | 17.36 | 8.08 | 7.46 | 0.59 | 0.44 | 0.21 | 1.68 | 0.17 | 0.26 | 0.01 |
| **Honghe Hani Yi Autonomous Prefecture** | 4478422 | 27.2633 | 8.7687 | 2.9600 | 0.4467 | 0.0800 | 39.5187 | 139.38 | 3799.87 | 1222.15 | 412.55 | 62.25 | 11.15 | 5507.98 | 1244.00 | 964.60 | 449.33 | 414.62 | 32.97 | 24.63 | 11.69 | 93.55 | 9.33 | 14.55 | 0.37 |
| Gejiu City | 419314 | 3.7333 | 0.4200 | 0.2467 | 0.0133 | 0.0000 | 4.4133 | 269.12 | 1004.71 | 113.03 | 66.38 | 3.59 | 0.00 | 1187.71 | 227.32 | 176.26 | 82.11 | 75.76 | 6.02 | 4.50 | 2.14 | 17.09 | 1.70 | 2.66 | 0.07 |
| Kaiyuan City | 323031 | 3.9800 | 1.7667 | 0.5733 | 0.1800 | 0.0800 | 6.5800 | 166.51 | 662.71 | 294.17 | 95.47 | 29.97 | 13.32 | 1095.64 | 280.90 | 217.81 | 101.46 | 93.63 | 7.44 | 5.56 | 2.64 | 21.12 | 2.11 | 3.29 | 0.08 |
| Mengzi Ctiy | 585976 | 3.0333 | 1.1467 | 0.5667 | 0.2200 | 0.0000 | 4.9667 | 269.70 | 818.09 | 309.26 | 152.83 | 59.33 | 0.00 | 1339.51 | 354.65 | 275.00 | 128.10 | 118.21 | 9.40 | 7.02 | 3.33 | 26.67 | 2.66 | 4.15 | 0.11 |
| Mile City | 538083 | 7.9700 | 3.4867 | 0.0000 | 0.0000 | 0.0000 | 11.4567 | 137.55 | 1096.25 | 479.58 | 0.00 | 0.00 | 0.00 | 1575.83 | 308.31 | 239.06 | 111.36 | 102.76 | 8.17 | 6.10 | 2.90 | 23.18 | 2.31 | 3.61 | 0.09 |
| Pingbian Miao Autonomous County | 129448 | 0.0000 | 0.0000 | 0.0000 | 0.0000 | 0.0000 | 0.0000 | 70.23 | 0.00 | 0.00 | 0.00 | 0.00 | 0.00 | 0.00 | 0.00 | 0.00 | 0.00 | 0.00 | 0.00 | 0.00 | 0.00 | 0.00 | 0.00 | 0.00 | 0.00 |
| Jianshui County | 534205 | 6.6800 | 1.8667 | 1.5667 | 0.0333 | 0.0000 | 10.1467 | 141.27 | 943.67 | 263.70 | 221.32 | 4.71 | 0.00 | 1433.40 | 357.22 | 276.99 | 129.03 | 119.06 | 9.47 | 7.07 | 3.36 | 26.86 | 2.68 | 4.18 | 0.11 |
| Shiping County | 271951 | 1.6333 | 0.0333 | 0.0000 | 0.0000 | 0.0000 | 1.6667 | 89.41 | 146.03 | 2.98 | 0.00 | 0.00 | 0.00 | 149.01 | 22.80 | 17.68 | 8.23 | 7.60 | 0.60 | 0.45 | 0.21 | 1.71 | 0.17 | 0.27 | 0.01 |
| Luxi County | 389138 | 0.0000 | 0.0000 | 0.0000 | 0.0000 | 0.0000 | 0.0000 | 236.50 | 0.00 | 0.00 | 0.00 | 0.00 | 0.00 | 0.00 | 0.00 | 0.00 | 0.00 | 0.00 | 0.00 | 0.00 | 0.00 | 0.00 | 0.00 | 0.00 | 0.00 |
| Yuanyang County | 359155 | 0.2333 | 0.0487 | 0.0067 | 0.0000 | 0.0000 | 0.2887 | 162.33 | 37.88 | 7.90 | 1.08 | 0.00 | 0.00 | 46.86 | 8.70 | 6.75 | 3.14 | 2.90 | 0.23 | 0.17 | 0.08 | 0.65 | 0.07 | 0.10 | 0.00 |
| Honghe County | 284607 | 0.0000 | 0.0000 | 0.0000 | 0.0000 | 0.0000 | 0.0000 | 140.30 | 0.00 | 0.00 | 0.00 | 0.00 | 0.00 | 0.00 | 0.00 | 0.00 | 0.00 | 0.00 | 0.00 | 0.00 | 0.00 | 0.00 | 0.00 | 0.00 | 0.00 |
| Jinping Miao, Yao and Dai Autonomous County | 331377 | 0.0000 | 0.0000 | 0.0000 | 0.0000 | 0.0000 | 0.0000 | 92.72 | 0.00 | 0.00 | 0.00 | 0.00 | 0.00 | 0.00 | 0.00 | 0.00 | 0.00 | 0.00 | 0.00 | 0.00 | 0.00 | 0.00 | 0.00 | 0.00 | 0.00 |
| Luchun County | 210166 | 0.0000 | 0.0000 | 0.0000 | 0.0000 | 0.0000 | 0.0000 | 67.89 | 0.00 | 0.00 | 0.00 | 0.00 | 0.00 | 0.00 | 0.00 | 0.00 | 0.00 | 0.00 | 0.00 | 0.00 | 0.00 | 0.00 | 0.00 | 0.00 | 0.00 |
| Hekou Yao Autonomous County | 101971 | 0.0000 | 0.0000 | 0.0000 | 0.0000 | 0.0000 | 0.0000 | 76.86 | 0.00 | 0.00 | 0.00 | 0.00 | 0.00 | 0.00 | 0.00 | 0.00 | 0.00 | 0.00 | 0.00 | 0.00 | 0.00 | 0.00 | 0.00 | 0.00 | 0.00 |
| **Wenshan Zhuang and Miao Autonomous Prefecture** | 3503218 | 5.4620 | 14.0640 | 7.0647 | 5.6533 | 0.0953 | 32.3393 | 111.53 | 609.16 | 1568.53 | 787.91 | 630.50 | 10.63 | 3606.73 | 1548.65 | 1200.82 | 559.37 | 516.16 | 41.04 | 30.66 | 14.56 | 116.46 | 11.61 | 18.12 | 0.46 |
| Wenshan City | 623772 | 1.6840 | 4.4067 | 3.2840 | 2.6060 | 0.0140 | 11.9947 | 210.21 | 353.99 | 926.32 | 690.32 | 547.80 | 2.94 | 2521.38 | 1186.08 | 919.69 | 428.41 | 395.32 | 31.43 | 23.48 | 11.15 | 89.19 | 8.90 | 13.88 | 0.36 |
| Yanshan County | 476587 | 1.8100 | 2.8920 | 2.1287 | 1.4660 | 0.0100 | 8.3067 | 123.20 | 222.99 | 356.29 | 262.25 | 180.61 | 1.23 | 1023.36 | 443.27 | 343.72 | 160.11 | 147.74 | 11.75 | 8.78 | 4.17 | 33.33 | 3.32 | 5.19 | 0.13 |
| Xichou County | 203630 | 0.1100 | 0.1467 | 0.0000 | 0.0000 | 0.0000 | 0.2567 | 136.55 | 15.02 | 20.03 | 0.00 | 0.00 | 0.00 | 35.05 | 8.26 | 6.41 | 2.98 | 2.75 | 0.22 | 0.16 | 0.08 | 0.62 | 0.06 | 0.10 | 0.00 |
| Malipo County | 243587 | 0.0000 | 0.0000 | 0.0053 | 0.0000 | 0.0000 | 0.0053 | 103.88 | 0.00 | 0.00 | 0.55 | 0.00 | 0.00 | 0.55 | 0.33 | 0.26 | 0.12 | 0.11 | 0.01 | 0.01 | 0.00 | 0.02 | 0.00 | 0.00 | 0.00 |
| Maguan County | 318704 | 0.0000 | 0.0000 | 0.0000 | 0.0000 | 0.0000 | 0.0000 | 119.70 | 0.00 | 0.00 | 0.00 | 0.00 | 0.00 | 0.00 | 0.00 | 0.00 | 0.00 | 0.00 | 0.00 | 0.00 | 0.00 | 0.00 | 0.00 | 0.00 | 0.00 |
| Qiu Bei County | 468172 | 0.6733 | 2.7473 | 0.6187 | 1.4427 | 0.0700 | 5.5520 | 92.63 | 62.37 | 254.48 | 57.31 | 133.63 | 6.48 | 514.26 | 232.82 | 180.53 | 84.09 | 77.60 | 6.17 | 4.61 | 2.19 | 17.51 | 1.75 | 2.72 | 0.07 |
| Guangnan County | 771948 | 1.0360 | 3.8220 | 0.9273 | 0.1373 | 0.0000 | 5.9227 | 99.87 | 103.46 | 381.70 | 92.61 | 13.72 | 0.00 | 591.49 | 196.57 | 152.42 | 71.00 | 65.52 | 5.21 | 3.89 | 1.85 | 14.78 | 1.47 | 2.30 | 0.06 |
| Funing County | 396818 | 0.1487 | 0.0493 | 0.1007 | 0.0013 | 0.0013 | 0.3013 | 74.97 | 11.15 | 3.70 | 7.55 | 0.10 | 0.10 | 22.59 | 7.48 | 5.80 | 2.70 | 2.49 | 0.20 | 0.15 | 0.07 | 0.56 | 0.06 | 0.09 | 0.00 |
| **Xishuangbanna Dai Autonomous Prefecture** | 1301407 | 14.1667 | 6.9333 | 3.3333 | 0.6667 | 0.1867 | 25.2867 | 68.34 | 968.10 | 473.80 | 227.79 | 45.56 | 12.76 | 1727.99 | 471.95 | 365.95 | 170.47 | 157.30 | 12.51 | 9.34 | 4.44 | 35.49 | 3.54 | 5.52 | 0.14 |
| Jinghong City | 642737 | 9.3333 | 6.9333 | 3.3333 | 0.6667 | 0.1867 | 20.4533 | 93.79 | 875.39 | 650.29 | 312.64 | 62.53 | 17.51 | 1918.36 | 579.76 | 449.55 | 209.41 | 193.23 | 15.36 | 11.48 | 5.45 | 43.60 | 4.35 | 6.78 | 0.17 |
| Menghai County | 353720 | 2.4333 | 0.0000 | 0.0000 | 0.0000 | 0.0000 | 2.4333 | 65.88 | 160.31 | 0.00 | 0.00 | 0.00 | 0.00 | 160.31 | 24.05 | 18.65 | 8.69 | 8.01 | 0.64 | 0.48 | 0.23 | 1.81 | 0.18 | 0.28 | 0.01 |
| Mengla County | 304950 | 2.4000 | 0.0000 | 0.0000 | 0.0000 | 0.0000 | 2.4000 | 44.70 | 107.28 | 0.00 | 0.00 | 0.00 | 0.00 | 107.28 | 16.09 | 12.48 | 5.81 | 5.36 | 0.43 | 0.32 | 0.15 | 1.21 | 0.12 | 0.19 | 0.00 |
| **Dali Bai Autonomous Prefecture** | 3337559 | 3.4053 | 0.0000 | 0.0000 | 0.0000 | 0.0000 | 3.4053 | 117.94 | 401.62 | 0.00 | 0.00 | 0.00 | 0.00 | 401.62 | 60.24 | 46.71 | 21.76 | 20.08 | 1.60 | 1.19 | 0.57 | 4.53 | 0.45 | 0.70 | 0.02 |
| Dali City | 771128 | 1.7553 | 0.0000 | 0.0000 | 0.0000 | 0.0000 | 1.7553 | 443.56 | 778.59 | 0.00 | 0.00 | 0.00 | 0.00 | 778.59 | 116.79 | 90.56 | 42.18 | 38.93 | 3.09 | 2.31 | 1.10 | 8.78 | 0.88 | 1.37 | 0.04 |
| Yangbi Yi Autonomous County | 97610 | 0.0000 | 0.0000 | 0.0000 | 0.0000 | 0.0000 | 0.0000 | 52.51 | 0.00 | 0.00 | 0.00 | 0.00 | 0.00 | 0.00 | 0.00 | 0.00 | 0.00 | 0.00 | 0.00 | 0.00 | 0.00 | 0.00 | 0.00 | 0.00 | 0.00 |
| Xiangyun County | 406642 | 0.0033 | 0.0000 | 0.0000 | 0.0000 | 0.0000 | 0.0033 | 167.32 | 0.56 | 0.00 | 0.00 | 0.00 | 0.00 | 0.56 | 0.08 | 0.06 | 0.03 | 0.03 | 0.00 | 0.00 | 0.00 | 0.01 | 0.00 | 0.00 | 0.00 |
| Binchuan County | 341319 | 0.0000 | 0.0000 | 0.0000 | 0.0000 | 0.0000 | 0.0000 | 134.77 | 0.00 | 0.00 | 0.00 | 0.00 | 0.00 | 0.00 | 0.00 | 0.00 | 0.00 | 0.00 | 0.00 | 0.00 | 0.00 | 0.00 | 0.00 | 0.00 | 0.00 |
| Midu County | 261205 | 1.6467 | 0.0000 | 0.0000 | 0.0000 | 0.0000 | 1.6467 | 170.85 | 281.32 | 0.00 | 0.00 | 0.00 | 0.00 | 281.32 | 42.20 | 32.72 | 15.24 | 14.06 | 1.12 | 0.84 | 0.40 | 3.17 | 0.32 | 0.49 | 0.01 |
| Nanjian Yi Autonomous County | 192942 | 0.0000 | 0.0000 | 0.0000 | 0.0000 | 0.0000 | 0.0000 | 110.86 | 0.00 | 0.00 | 0.00 | 0.00 | 0.00 | 0.00 | 0.00 | 0.00 | 0.00 | 0.00 | 0.00 | 0.00 | 0.00 | 0.00 | 0.00 | 0.00 | 0.00 |
| Weishan Yi Hui Autonomous County | 267474 | 0.0000 | 0.0000 | 0.0000 | 0.0000 | 0.0000 | 0.0000 | 122.85 | 0.00 | 0.00 | 0.00 | 0.00 | 0.00 | 0.00 | 0.00 | 0.00 | 0.00 | 0.00 | 0.00 | 0.00 | 0.00 | 0.00 | 0.00 | 0.00 | 0.00 |
| Yongping County | 164613 | 0.0000 | 0.0000 | 0.0000 | 0.0000 | 0.0000 | 0.0000 | 58.98 | 0.00 | 0.00 | 0.00 | 0.00 | 0.00 | 0.00 | 0.00 | 0.00 | 0.00 | 0.00 | 0.00 | 0.00 | 0.00 | 0.00 | 0.00 | 0.00 | 0.00 |
| Yunlong County | 182977 | 0.0000 | 0.0000 | 0.0000 | 0.0000 | 0.0000 | 0.0000 | 41.86 | 0.00 | 0.00 | 0.00 | 0.00 | 0.00 | 0.00 | 0.00 | 0.00 | 0.00 | 0.00 | 0.00 | 0.00 | 0.00 | 0.00 | 0.00 | 0.00 | 0.00 |
| Eryuan County | 248147 | 0.0000 | 0.0000 | 0.0000 | 0.0000 | 0.0000 | 0.0000 | 98.05 | 0.00 | 0.00 | 0.00 | 0.00 | 0.00 | 0.00 | 0.00 | 0.00 | 0.00 | 0.00 | 0.00 | 0.00 | 0.00 | 0.00 | 0.00 | 0.00 | 0.00 |
| Jianchuan County | 160471 | 0.0000 | 0.0000 | 0.0000 | 0.0000 | 0.0000 | 0.0000 | 71.76 | 0.00 | 0.00 | 0.00 | 0.00 | 0.00 | 0.00 | 0.00 | 0.00 | 0.00 | 0.00 | 0.00 | 0.00 | 0.00 | 0.00 | 0.00 | 0.00 | 0.00 |
| Heqing County | 243031 | 0.0000 | 0.0000 | 0.0000 | 0.0000 | 0.0000 | 0.0000 | 102.83 | 0.00 | 0.00 | 0.00 | 0.00 | 0.00 | 0.00 | 0.00 | 0.00 | 0.00 | 0.00 | 0.00 | 0.00 | 0.00 | 0.00 | 0.00 | 0.00 | 0.00 |
| **Dehong Dai Jingpo Autonomous Prefecture** | 1315709 | 19.5100 | 31.7420 | 10.4707 | 3.1000 | 1.8667 | 66.6893 | 117.74 | 2297.20 | 3737.45 | 1232.86 | 365.01 | 219.79 | 7852.31 | 2695.35 | 2089.98 | 973.56 | 898.36 | 71.43 | 53.37 | 25.34 | 202.69 | 20.22 | 31.54 | 0.81 |
| Ruili City | 267638 | 15.3333 | 1.8000 | 0.0667 | 0.0000 | 0.0000 | 17.2000 | 284.40 | 4360.81 | 511.92 | 18.96 | 0.00 | 0.00 | 4891.69 | 819.07 | 635.11 | 295.85 | 273.00 | 21.71 | 16.22 | 7.70 | 61.59 | 6.14 | 9.58 | 0.25 |
| Mang City | 439931 | 0.0000 | 25.9220 | 7.5587 | 2.4000 | 1.5333 | 37.4140 | 151.79 | 0.00 | 3934.68 | 1147.32 | 364.29 | 232.74 | 5679.05 | 2369.70 | 1837.47 | 855.94 | 789.82 | 62.80 | 46.92 | 22.28 | 178.20 | 17.77 | 27.73 | 0.71 |
| Lianghe County | 134268 | 0.3333 | 2.0000 | 1.3333 | 0.6667 | 0.3333 | 4.6667 | 117.93 | 39.31 | 235.87 | 157.25 | 78.62 | 39.31 | 550.36 | 269.28 | 208.80 | 97.26 | 89.75 | 7.14 | 5.33 | 2.53 | 20.25 | 2.02 | 3.15 | 0.08 |
| Yingjiang County | 292508 | 3.1733 | 0.9667 | 0.4587 | 0.0333 | 0.0000 | 4.6320 | 67.58 | 214.44 | 65.32 | 30.99 | 2.25 | 0.00 | 313.01 | 72.16 | 55.95 | 26.06 | 24.05 | 1.91 | 1.43 | 0.68 | 5.43 | 0.54 | 0.84 | 0.02 |
| Longchuan County | 181364 | 0.6700 | 1.0533 | 1.0533 | 0.0000 | 0.0000 | 2.7767 | 97.10 | 65.06 | 102.28 | 102.28 | 0.00 | 0.00 | 269.62 | 101.81 | 78.94 | 36.77 | 33.93 | 2.70 | 2.02 | 0.96 | 7.66 | 0.76 | 1.19 | 0.03 |
| **Nujiang Lisu Autonomous Prefecture** | 552694 | 0.0000 | 0.0000 | 0.0000 | 0.0000 | 0.0000 | 0.0000 | 37.85 | 0.00 | 0.00 | 0.00 | 0.00 | 0.00 | 0.00 | 0.00 | 0.00 | 0.00 | 0.00 | 0.00 | 0.00 | 0.00 | 0.00 | 0.00 | 0.00 | 0.00 |
| Lushui City | 203977 | 0.0000 | 0.0000 | 0.0000 | 0.0000 | 0.0000 | 0.0000 | 65.83 | 0.00 | 0.00 | 0.00 | 0.00 | 0.00 | 0.00 | 0.00 | 0.00 | 0.00 | 0.00 | 0.00 | 0.00 | 0.00 | 0.00 | 0.00 | 0.00 | 0.00 |
| Fugong County | 114372 | 0.0000 | 0.0000 | 0.0000 | 0.0000 | 0.0000 | 0.0000 | 41.67 | 0.00 | 0.00 | 0.00 | 0.00 | 0.00 | 0.00 | 0.00 | 0.00 | 0.00 | 0.00 | 0.00 | 0.00 | 0.00 | 0.00 | 0.00 | 0.00 | 0.00 |
| Gongshan Dulong Nu Autonomous County | 38471 | 0.0000 | 0.0000 | 0.0000 | 0.0000 | 0.0000 | 0.0000 | 8.77 | 0.00 | 0.00 | 0.00 | 0.00 | 0.00 | 0.00 | 0.00 | 0.00 | 0.00 | 0.00 | 0.00 | 0.00 | 0.00 | 0.00 | 0.00 | 0.00 | 0.00 |
| Lanping Bai Pumi Autonomous County | 195874 | 0.0000 | 0.0000 | 0.0000 | 0.0000 | 0.0000 | 0.0000 | 44.79 | 0.00 | 0.00 | 0.00 | 0.00 | 0.00 | 0.00 | 0.00 | 0.00 | 0.00 | 0.00 | 0.00 | 0.00 | 0.00 | 0.00 | 0.00 | 0.00 | 0.00 |
| **Diqing Tibetan Autonomous Prefecture** | 387511 | 0.0000 | 0.0000 | 0.0000 | 0.0000 | 0.0000 | 0.0000 | 16.71 | 0.00 | 0.00 | 0.00 | 0.00 | 0.00 | 0.00 | 0.00 | 0.00 | 0.00 | 0.00 | 0.00 | 0.00 | 0.00 | 0.00 | 0.00 | 0.00 | 0.00 |
| Xianggelila City | 186412 | 0.0000 | 0.0000 | 0.0000 | 0.0000 | 0.0000 | 0.0000 | 16.32 | 0.00 | 0.00 | 0.00 | 0.00 | 0.00 | 0.00 | 0.00 | 0.00 | 0.00 | 0.00 | 0.00 | 0.00 | 0.00 | 0.00 | 0.00 | 0.00 | 0.00 |
| Deqin County | 54736 | 0.0000 | 0.0000 | 0.0000 | 0.0000 | 0.0000 | 0.0000 | 7.50 | 0.00 | 0.00 | 0.00 | 0.00 | 0.00 | 0.00 | 0.00 | 0.00 | 0.00 | 0.00 | 0.00 | 0.00 | 0.00 | 0.00 | 0.00 | 0.00 | 0.00 |
| Weixi Lisu Autonomous County | 146363 | 0.0000 | 0.0000 | 0.0000 | 0.0000 | 0.0000 | 0.0000 | 32.72 | 0.00 | 0.00 | 0.00 | 0.00 | 0.00 | 0.00 | 0.00 | 0.00 | 0.00 | 0.00 | 0.00 | 0.00 | 0.00 | 0.00 | 0.00 | 0.00 | 0.00 |
