# Supplementary material for: Analysis of the human health threat caused by the red imported fire ant in mainland China
Source: PLoS One. 2026 Jun 16;21(6):e0350501. doi: 10.1371/journal.pone.0350501 (PMC13271487; doi:10.1371/journal.pone.0350501)

**URL and content of news reports related to red imported fire ant stings.**

N1：<https://www.thepaper.cn/newsDetail_forward_12381597>


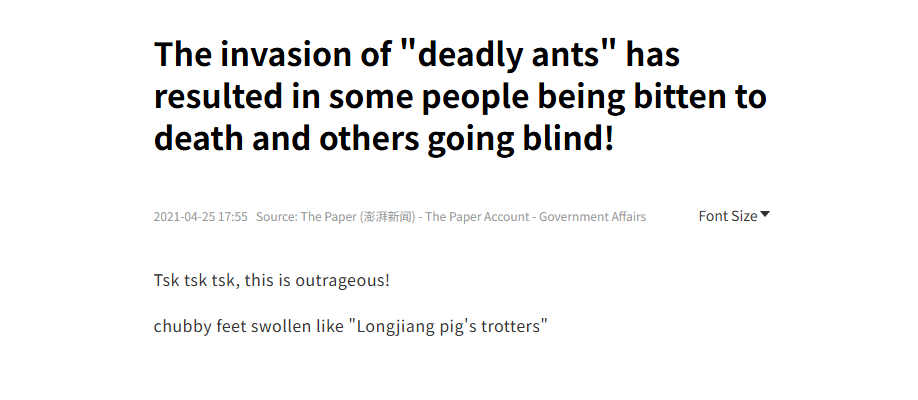


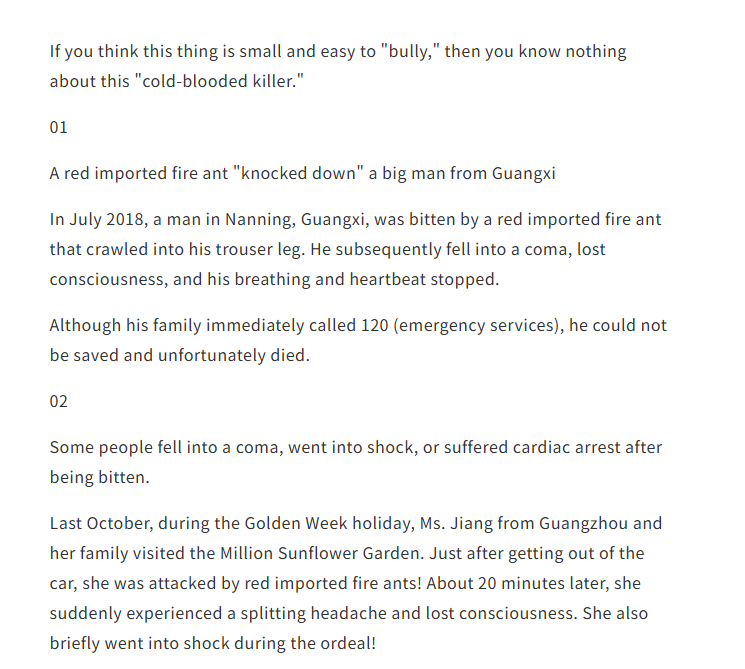


N2：<https://www.thepaper.cn/newsDetail_forward_27191127>


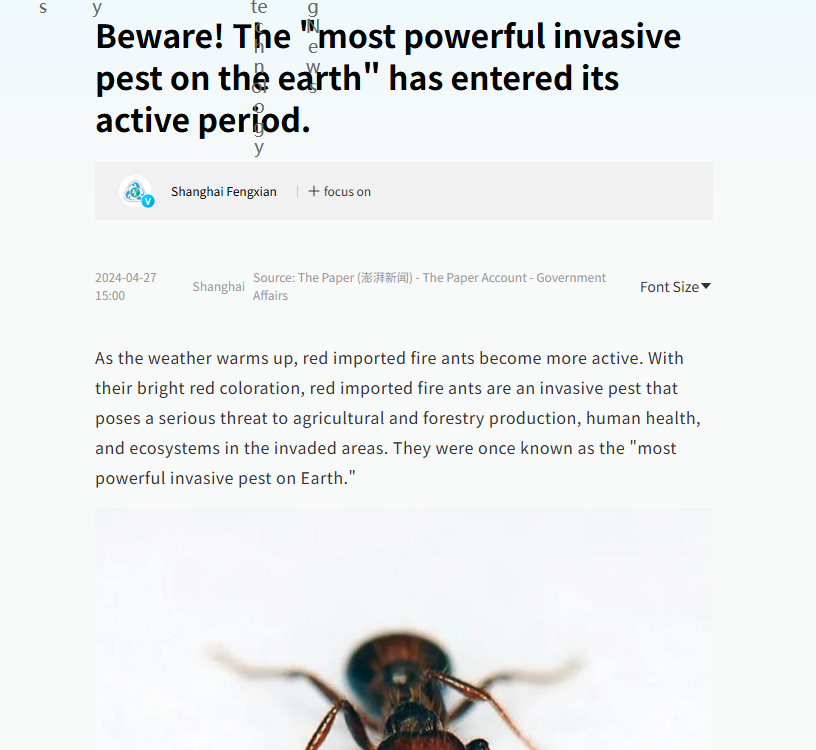


N3: <http://www.jksb.com.cn/html/xinwen/2021/0103/168522.html>


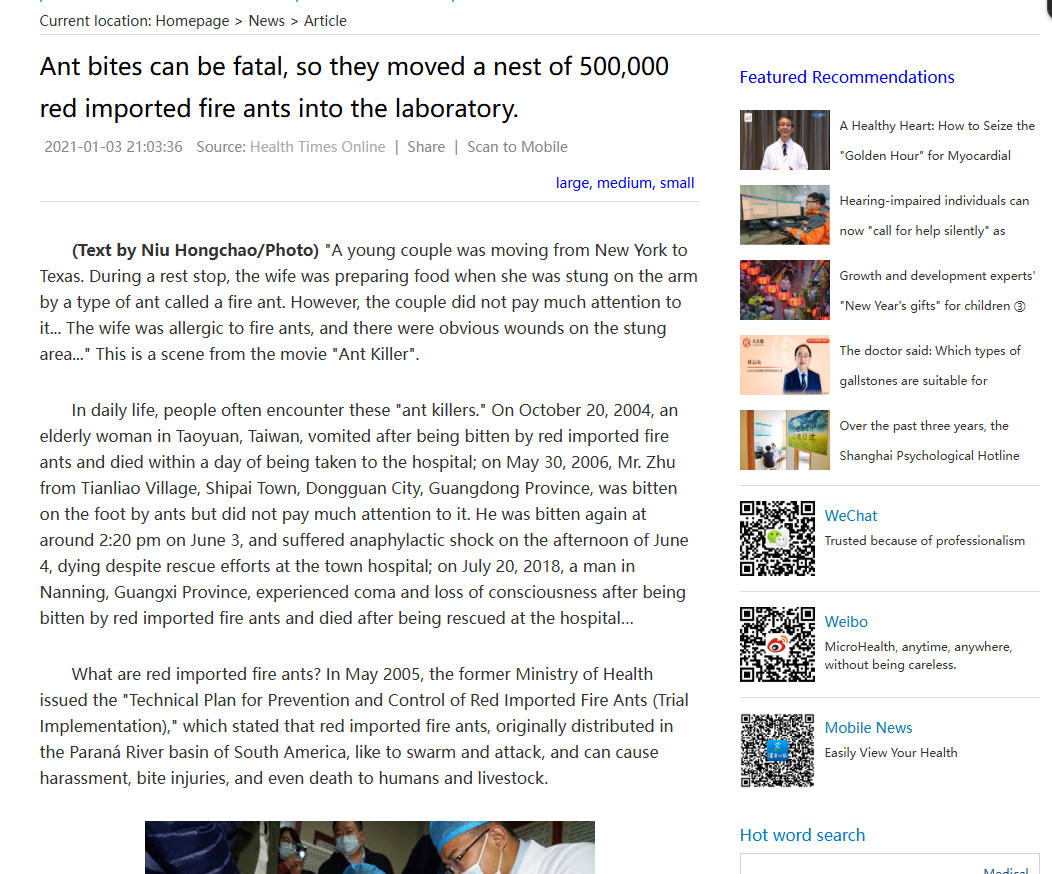


N4：<https://m.thepaper.cn/baijiahao_13867738>


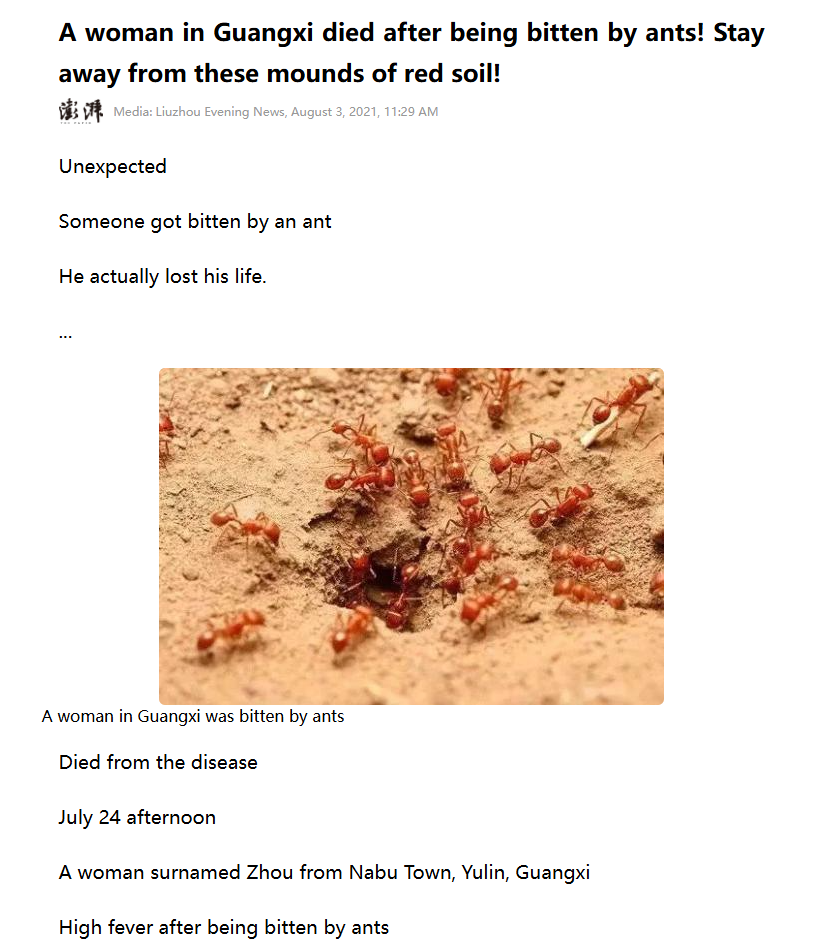


N5: <http://fj.people.com.cn/n2/2023/0729/c181466-40512152.html>


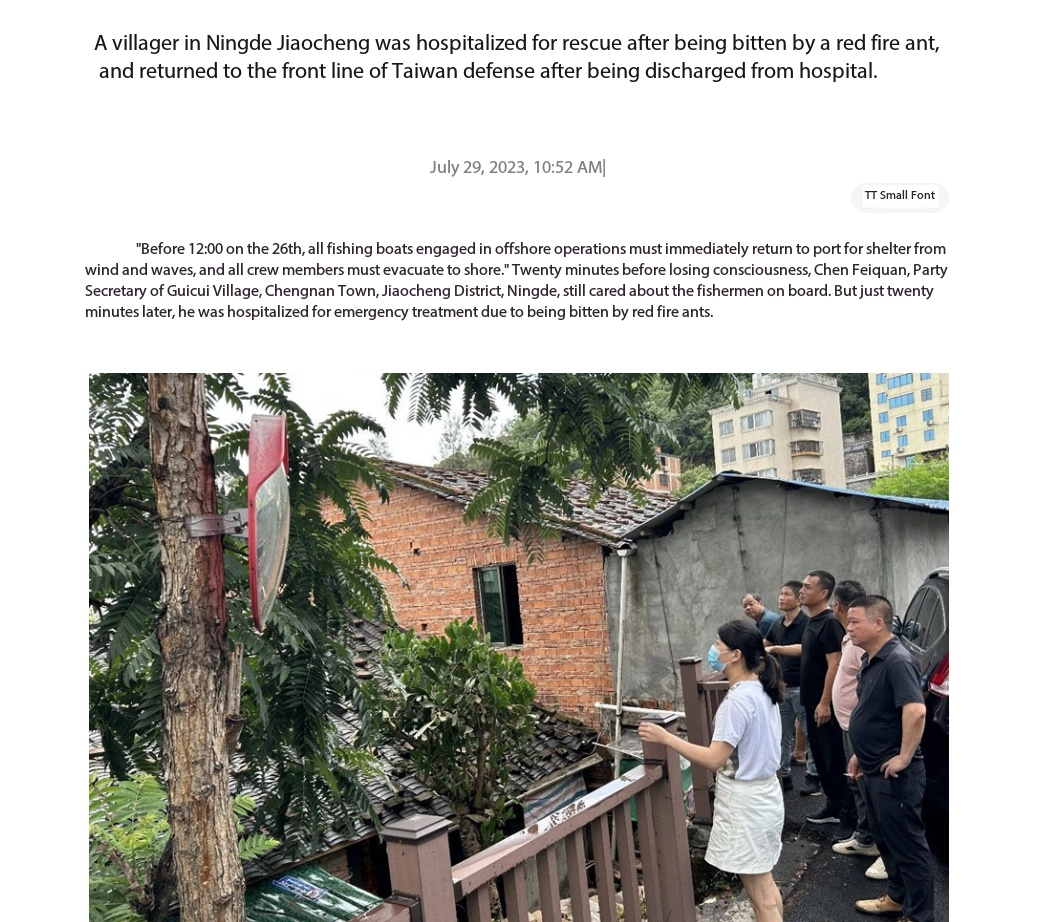


N6: <https://www.360kuai.com/pc/99d50548e678e64e1?cota=3&kuai_so=1&sign=360_57c3bbd1&refer_scene=so_1++https%3A%2F%2Fwww.163.com%2Fdy%2Farticle%2FGCSDK4IE0514R9KQ.html>


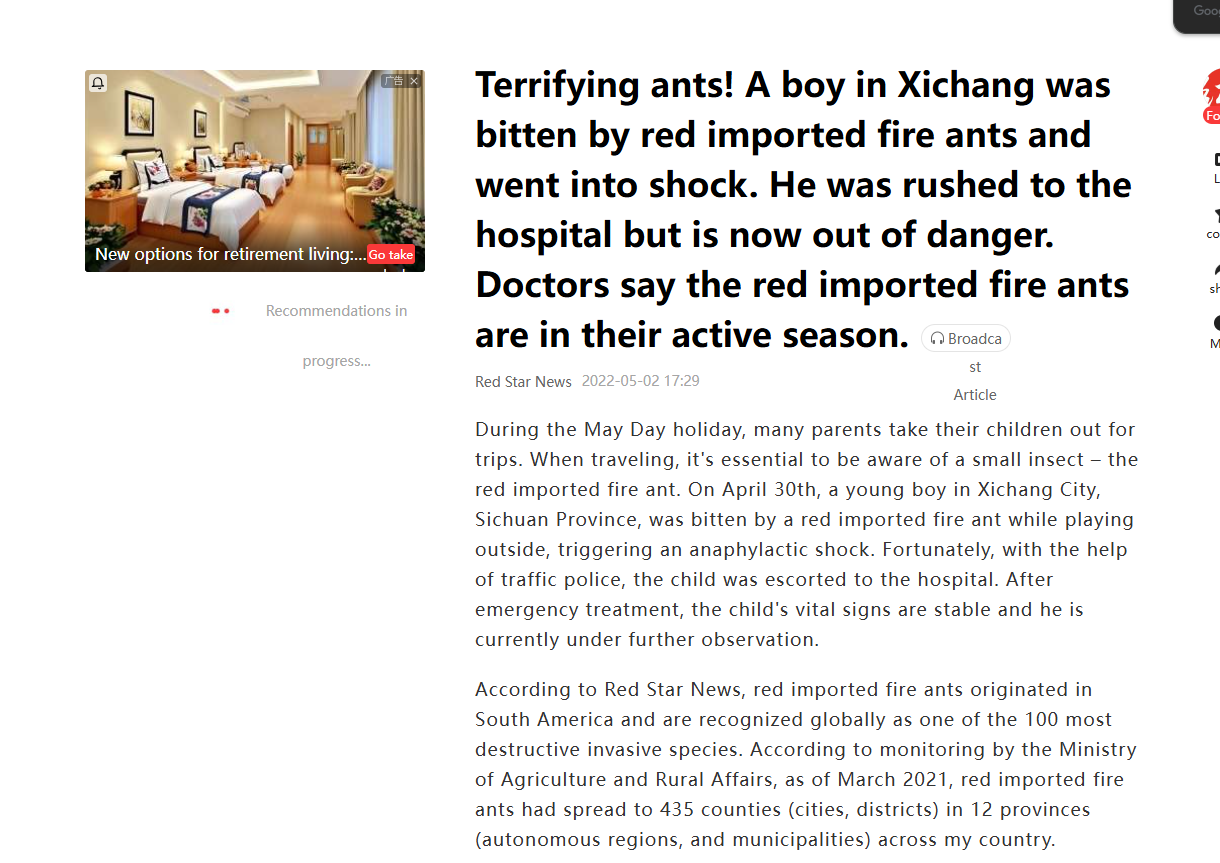


N7: <https://www.sohu.com/a/396431100_255783>


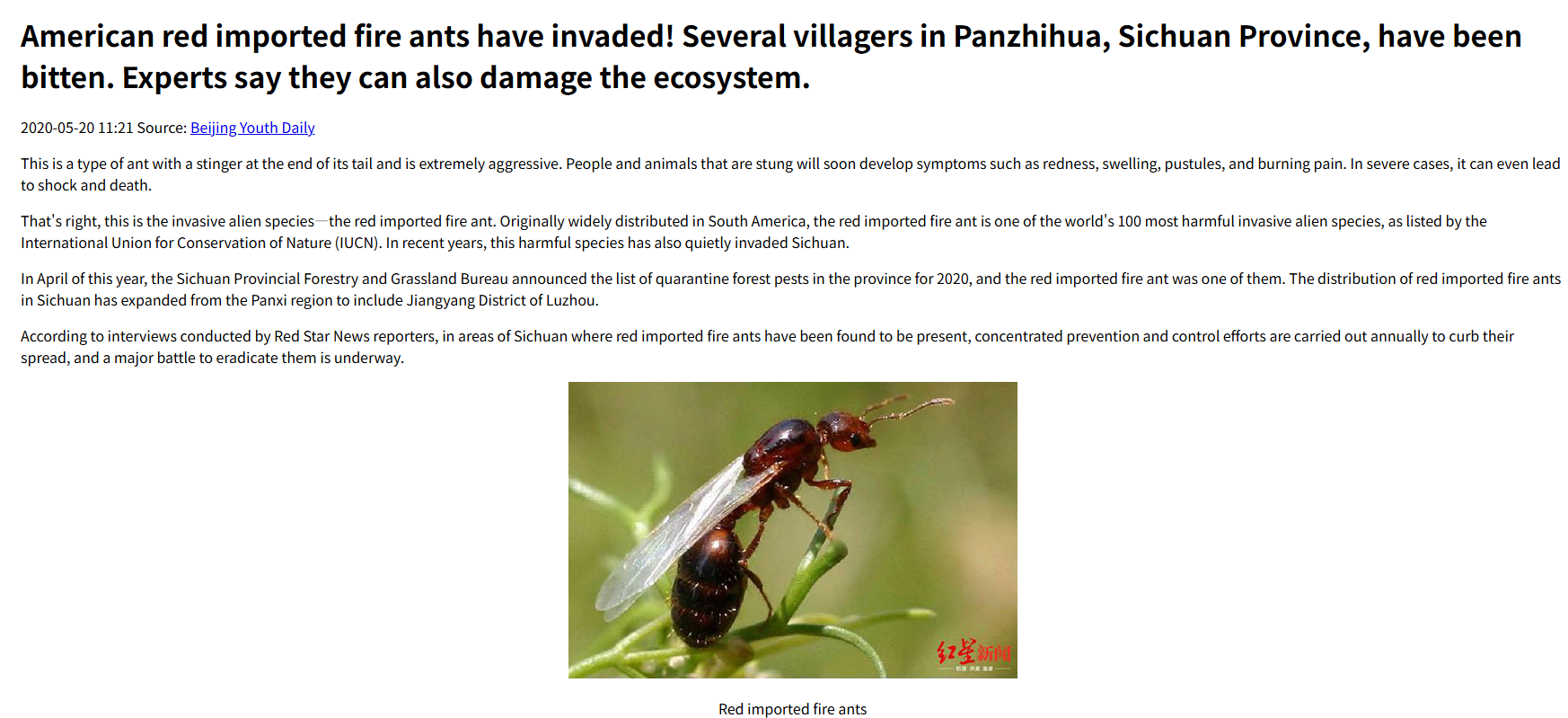


N8: [https://www.chuannan.net/wanxiang/22677.html](%20https:/www.chuannan.net/wanxiang/22677.html)


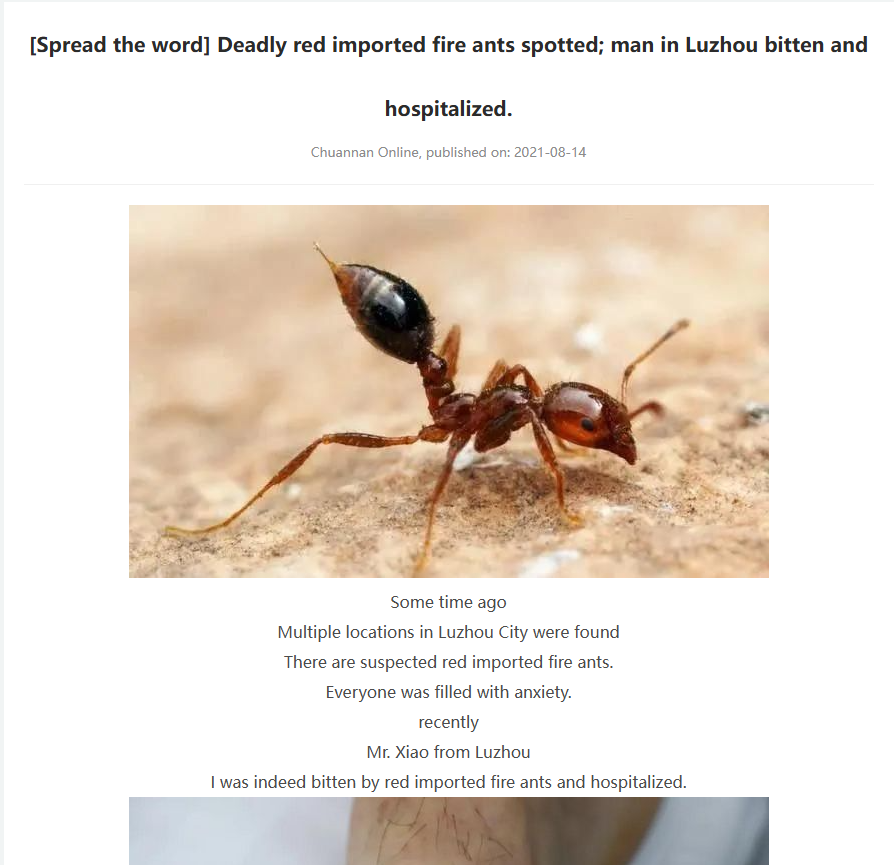


N9: <http://hainan.sina.com.cn/news/s/2015-07-13/detail-ifxewnia9125973.shtml>


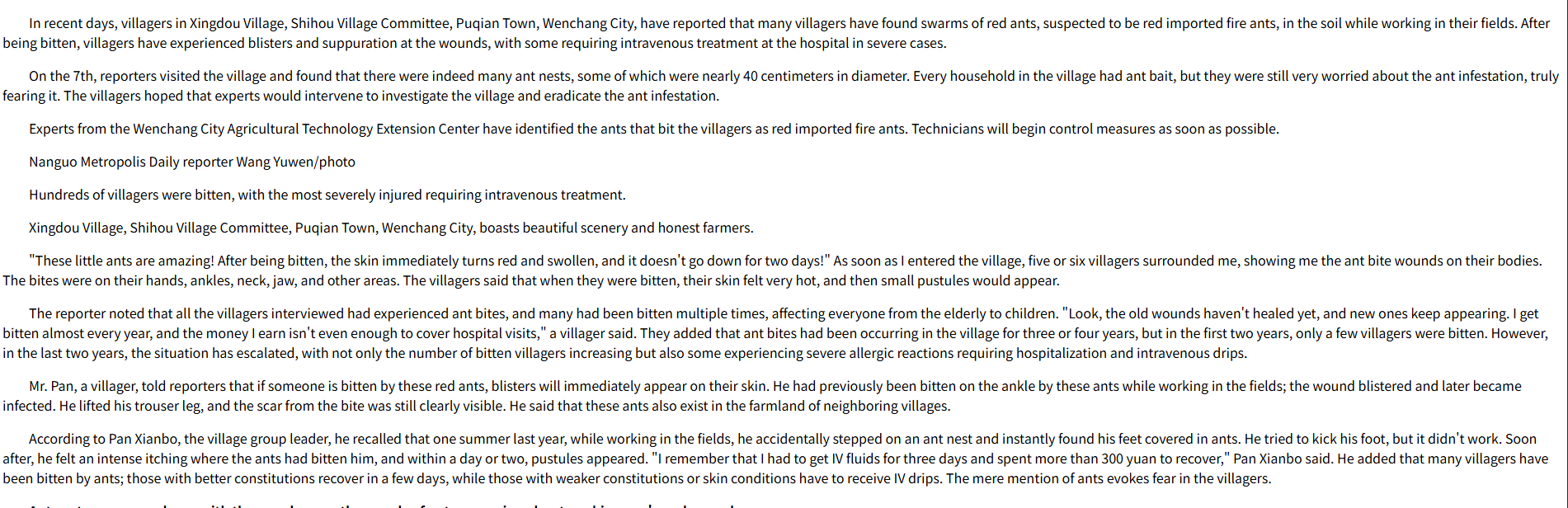


N10: <https://www.sohu.com/a/195077687_781899>


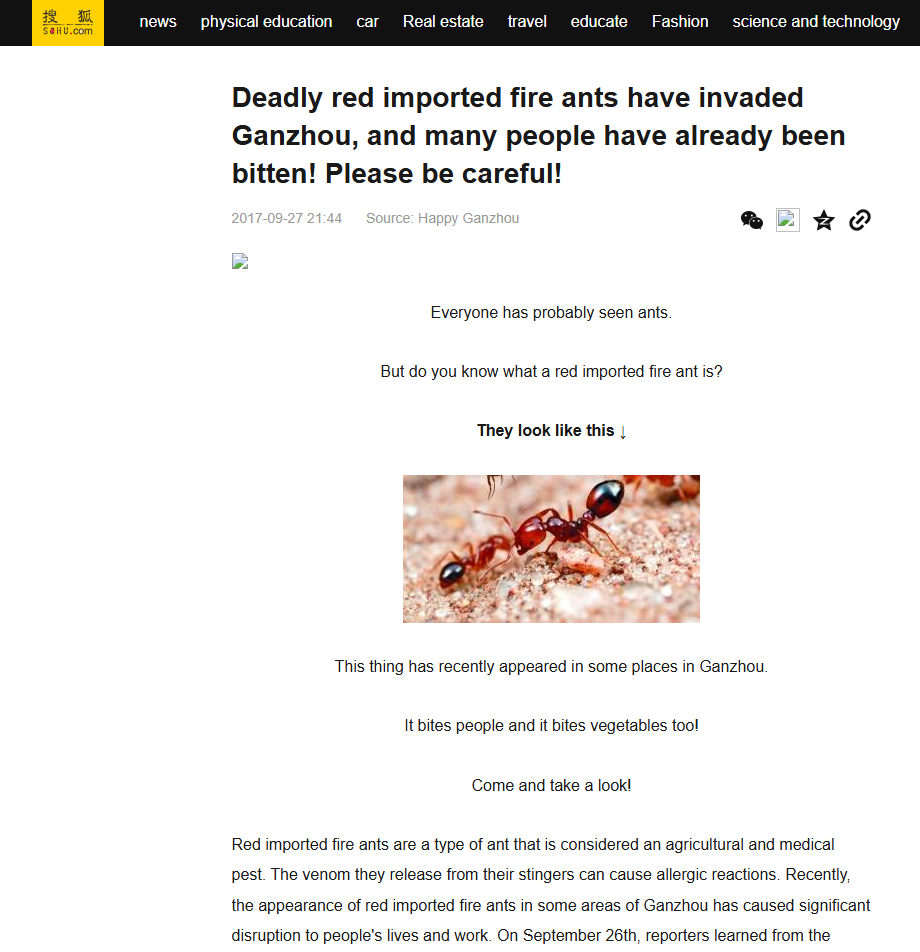


N11: <http://news.cnhubei.com/content/2021-03/31/content_13707689.html>


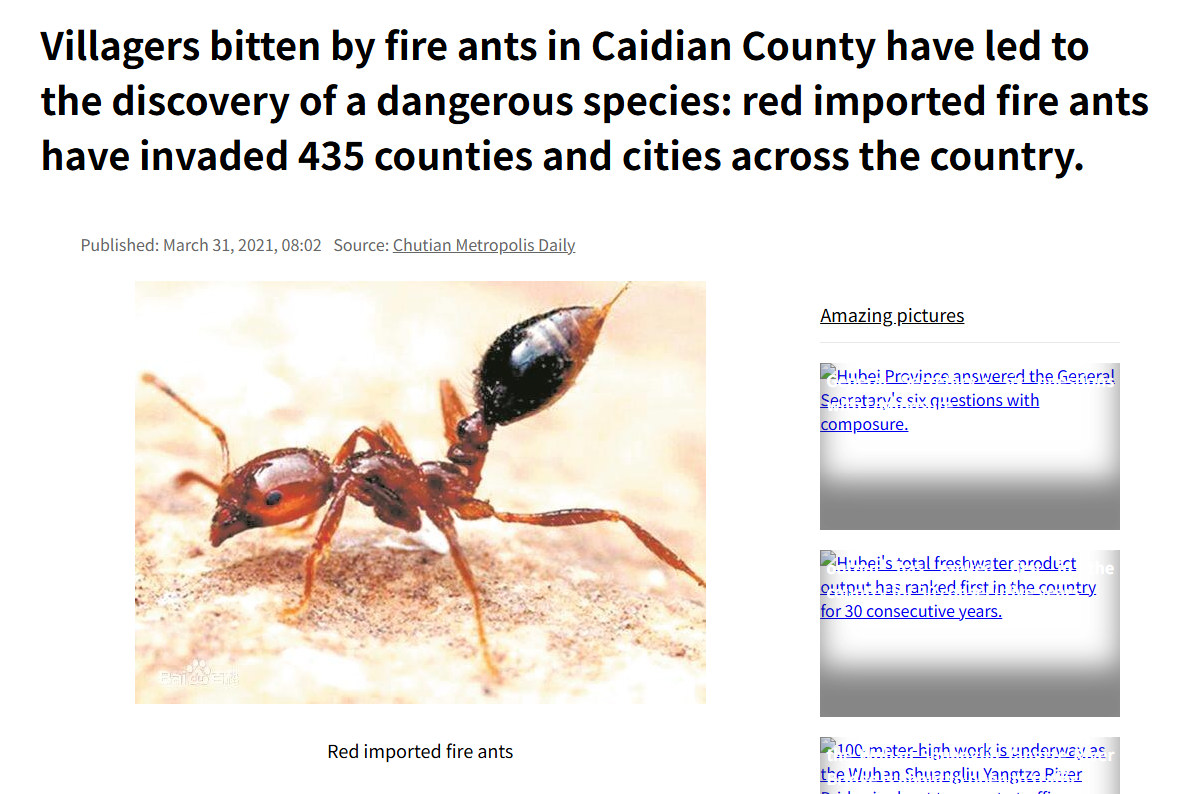


N12: <https://baijiahao.baidu.com/s?id=1697711335287703109&wfr=spider&for=pc>


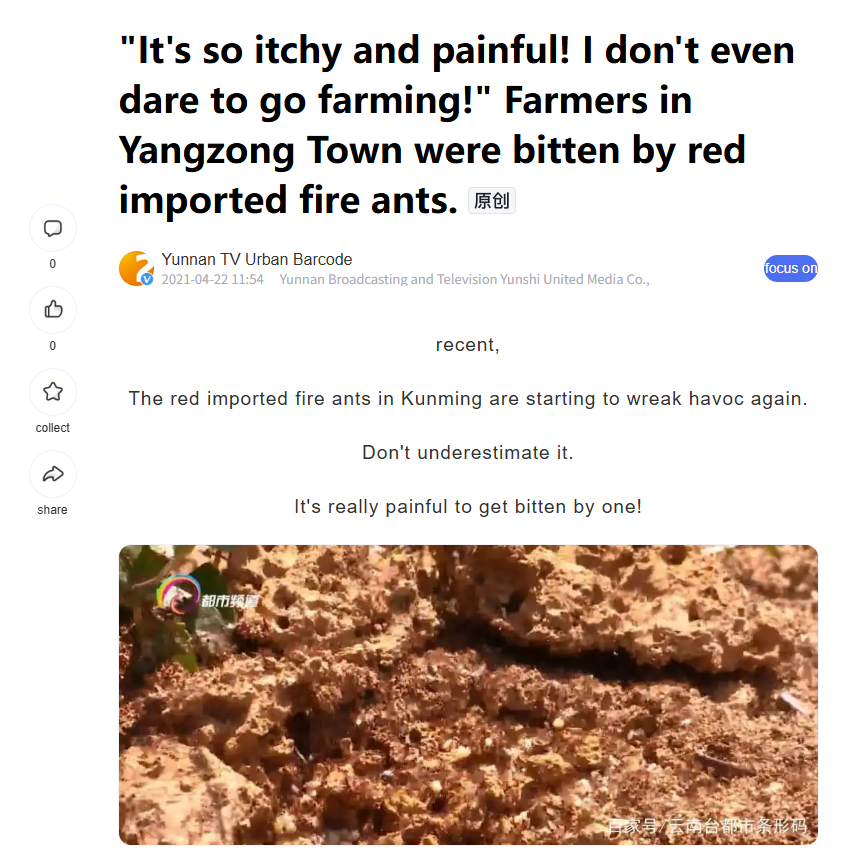


N13: <https://e.cdsb.com/html/2014-10/07/content_491791.htm>


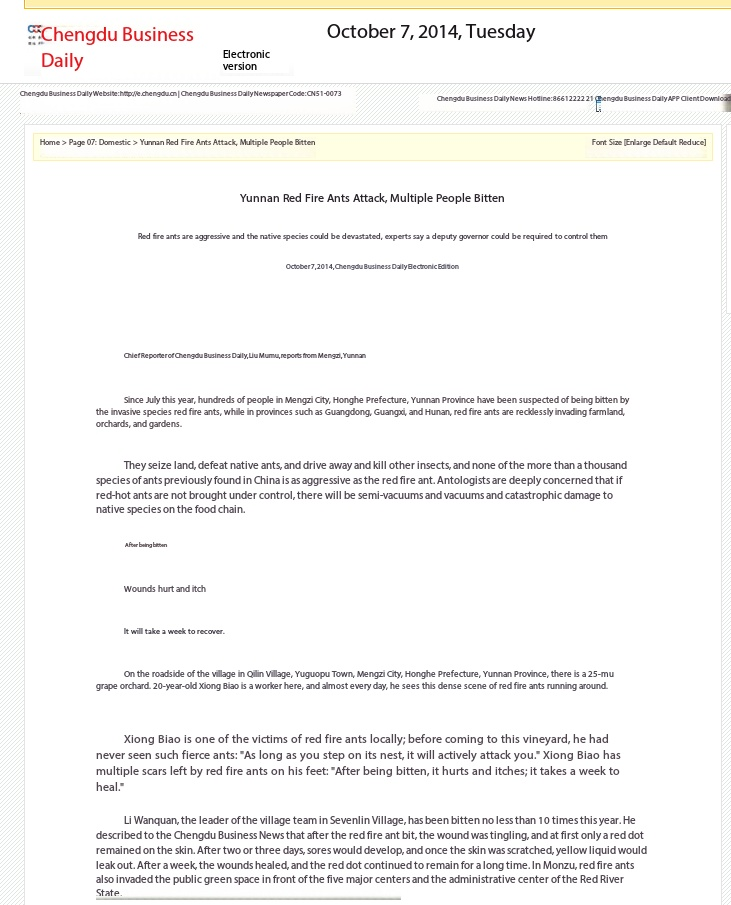

Supplement: S1 Text — (DOCX) [file pone.0350501.s002.docx]
